# Aquatic Insects from the Caatinga: checklists and diversity assessments of Ubajara (Ceará State) and Sete Cidades (Piauí State) National Parks, Northeastern Brazil

**DOI:** 10.3897/BDJ.4.e8354

**Published:** 2016-08-05

**Authors:** Daniela Maeda Takiya, Allan Paulo Moreira Santos, Ângelo Parise Pinto, Ana Lucia Henriques-Oliveira, Alcimar do Lago Carvalho, Brunno Henrique Lanzellotti Sampaio, Bruno Clarkson, Felipe Ferraz Figueiredo Moreira, Fernanda Avelino-Capistrano, Inês Corrêa Gonçalves, Isabelle da Rocha Silva Cordeiro, Josenir Teixeira Câmara, Julianna Freires Barbosa, W. Rafael Maciel de Souza, José Albertino Rafael

**Affiliations:** ‡ Laboratório de Entomologia, Departamento de Zoologia, Instituto de Biologia, Universidade Federal do Rio de Janeiro, Rio de Janeiro, Brazil; § Departamento de Zoologia, Universidade Federal do Estado do Rio de Janeiro, Rio de Janeiro, Brazil; | Laboratório de Biologia e Sistemática de Odonata (LABIOSIS), Departamento de Entomologia, Museu Nacional, Universidade Federal do Rio de Janeiro, Rio de Janeiro, Brazil; ¶ Laboratório Nacional e Internacional de Referência em Taxonomia de Triatomíneos, Instituto Oswaldo Cruz, Fundação Oswaldo Cruz, Rio de Janeiro, Brazil; # Coordenação de Biodiversidade, Instituto Nacional de Pesquisas da Amazônia, Manaus, Brazil

**Keywords:** Species richness, Amazonia, Cerrado, Atlantic forest, Freshwater macroinvertebrates

## Abstract

**Background:**

Diversity and distribution of Neotropical aquatic insects is still poorly known, with many species to be recorded and many others to be described, due to the small number of taxonomists and sparse faunistic studies. This knowledge is especially poor in the Caatinga Domain in Northeastern Brazil, even though, this region may have played an important historical role in the spatial evolution of faunas of forested areas in northern South America.

**New information:**

Aquatic insect checklists of 96 species from Parque Nacional de Ubajara (Ceará State, Brazil) and 112 species from Parque Nacional de Sete Cidades (Piauí State, Brazil) are presented, representing the following taxa: Elmidae, Epimetopidae, Hydrophilidae, and Torridincolidae (Coleoptera), Hemerodromiinae (Diptera: Empididae), Ephemeroptera, Gerromorpha and Nepomorpha (Hemiptera), Odonata, Plecoptera, and Trichoptera. Because of the scarce number of biological inventories in Northeastern Brazil, several new distributional records (of species, genera, and families) for Brazil, Northeastern Brazil, and Ceará and Piauí states are provided. In addition, several undescribed species were detected, being 26 from Ubajara and 20 from Sete Cidades. Results represent a significant increase to the known fauna of these states, ranging from 13%-70% increase for Ceará and 41% to 91% increase for Piauí. Although both parks are relatively close to each other and within the Caatinga domain, their aquatic fauna display a very high complementarity (89% species), possibly due to structural differences of water bodies sampled in each park. Rarefaction curves based on quantitative light trap samples suggest a much higher expected species richness of aquatic insects at Sete Cidades than at Ubajara National Park. Discussion on biogeographical affinities of this sample of the Caatinga fauna is provided.

## Introduction

### Aquatic insects

Insects constitute the most diverse animal group and represent one of the earliest lineages occupying the terrestrial habitat (Grimaldi and Engel 2005). Although they are mostly terrestrial, insects usually dominate inland waterbodies in terms of species number and biomass. Many terrestrial insects are resilient to eventual submersion in water (Chapman 1998), but true aquatic insects inhabit freshwater or marine environments at least for one life stage and have several morphological, physiological, and behavioural adaptations. Aquatic insects are usually abundant and important components of energy flow in freshwater habitats, and thus have been the focus of many ecological studies, as well as, the most frequent macroinvertebrate group used in biomonitoring (Rosenberg and Resh 1993, Merritt and Cummins 1996).

The diversity of aquatic insects is a result of several independent invasions of aquatic habitats by terrestrial lineages (more than 50 separate invasions, according to Dijkstra et al. (2014). Marine insects are relatively rare, most of them living in coastal areas and only very few species in the open sea (Cheng 1976). On the other hand, insects inhabiting freshwater are close to 100,000 described species, in twelve insect orders, five of them primarily aquatic: Ephemeroptera, Odonata, Plecoptera, Megaloptera, and Trichoptera (Dijkstra et al. 2014).

Regional knowledge of the aquatic insect fauna is essential to ecological surveys or biomonitoring. In the Neotropics, diversity and distribution of aquatic insects is still poorly known, with many species to be recorded and many others to be described, especially because of the small number of taxonomists and sparse faunistic studies, even near large urban areas. Currently, there are approximately 7,000 described species of aquatic insects in Brazil (Boeger et al. 2016), representing approximately 8% of known species in the world. Amongst these groups, only Odonata and Psychodomorpha reach numbers higher than 14% of the world’s fauna (Table 1). The number of species of some aquatic taxa recorded from Brazil, *e.g.*, Trichoptera, is far below the average of 8% of the world fauna, very likely due to the many undescribed species waiting to be formally named or recorded from the country. Undersampling is one of the major biases for richness evaluations studies and conservation actions such as recently demonstrated for Brazilian dragonflies (Vianna and De Marco Júnior 2012). Therefore, local inventories should remain a priority, particularly in those poorly sampled areas, such as Northeastern Brazil.

### The Caatinga Domain in Northeastern Brazil

**Figure 1.**
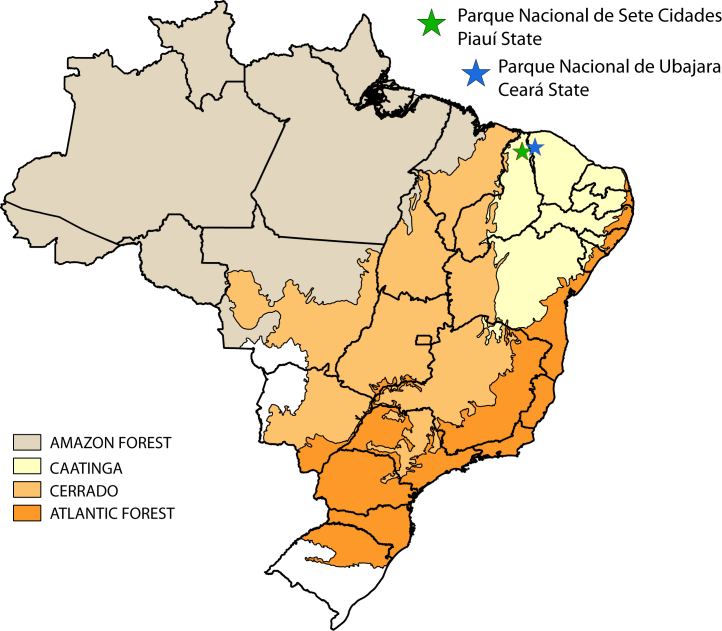
Map of Brazilian states colored by four major phytogeographical domains: Amazon forest, Cerrado, Atlantic forest, and Caatinga; the last one including Ubajara (blue star) and Sete Cidades (green star) National Parks.

The Caatinga Domain (Coutinho 2006) extends for an area of approximately 800,000 km2 in Northeastern Brazil (Fig. 1), including parts of the Brazilian states Piauí (PI), Ceará (CE), Rio Grande do Norte (RN), Paraíba (PB), Pernambuco (PE), Alagoas (AL), Sergipe (SE), Bahia (BA), and Minas Gerais (MG). It is a mosaic of different forest types, characterized by short trees and shrubs with xerophytic characteristics, influenced by climatic extremes when compared to other Brazilian formations: highest solar radiation, highest annual temperature mean, lowest rates of relative humidity, and lowest precipitation, which is limited to a very short period of the year (Prado 2003). It is estimated that over 28% of the original Caatinga vegetation has been altered by human activities, such as, slash-and-burn agriculture, harvesting of firewood, hunting, and herding (Castelletti et al. 2003). Currently, only less than 2% of its natural vegetation is enclosed under federal protected areas.

Low diversity and low numbers of endemic species is traditionally assigned to the Caatinga, although recent studies suggest otherwise, highlighting it as an important component of Brazilian biodiversity (Leal et al. 2005). The Caatinga lies in between two of the world’s biodiversity hotspots (Myers et al. 2000, Mittermeier et al. 2011), the Atlantic Forest and the Cerrado, and shares many biotic components with these domains. Current assessments of the Caatinga’s biodiversity is certainly undere﻿stimated because few biological inventories have focused this region, *e.g.*, for woody plants, 41% of its area has never been surveyed by scientists and 80% of what has been was surveyed poorly (Tabarelli and Vicente 2004). This scenario is certainly worse when it comes to general insect inventories.

### Previous records of aquatic insects in Northeastern Brazil

**Figure 2.**
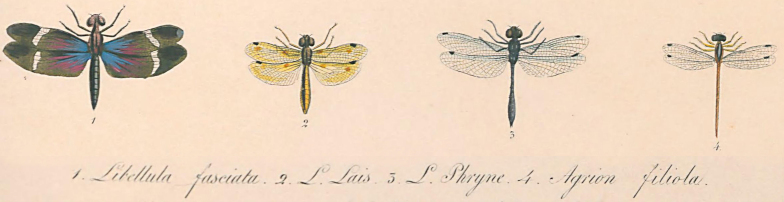
Detail of plate 25 modified from Joseph Anton Maximilian Perty’s work from 1834, with a water color illustration of the holotype of the small skimmer dragonfly *Libellula phryne* (Fig. 3, Odonata: Libellulidae; currently *Nephepeltia phryne*) described by him from "Provincia Piauhiensi", Brazil.

Due to the lack of collecting efforts in the Caatinga and Northeastern Brazil, and consequent scarcity of available voucher material from Ceará and Piauí states in insect collections, any information on insect diversity from inventories in these regions will probably constitute new records from these Brazilian states. An up to date list of Coleoptera (Hydrophilidae), Ephemeroptera, Hemiptera (Gerromorpha and Nepomorpha), Odonata, and Trichoptera recorded from Brazilian states of Ceará (CE) and Piauí (PI) are given in Table 2. This table includes species records previously published based on material collected during this project. Previous to this study, there were no records of Elmidae, Epimetopidae, and Torridincolidae (Coleoptera), Leptophlebiidae (Ephemeroptera), Gelastocoridae, Naucoridae, and Pleidae (Hemiptera), and Ecnomidae, Polycentropodidae, and Xyphocentronidae (Trichoptera) for these Brazilian states. Part of distributional data were initially compiled based on available information at Moreira et al. (2011), Moreira (2016), Salles et al. (2016), and Santos et al. (2016). Curiously, the first record of an aquatic insect from either state was of *Nephepeltia phryne* from Piauí described by Perty (1834) (Fig. 2) and, amazingly, a second dragonfly species was only recorded to this state after 160 years in 1994.

In this paper, we provide a preliminary checklist of selected taxa of aquatic insects from Ubajara and Sete Cidades National Parks in Northeastern Brazil (Fig. 1), an undersampled region in terms of biological inventories. Additionally, species richness was compared between the two National parks. As far as we know, this is the first published aquatic insect inventory of the selected National Parks.

## Materials and methods

### Focal taxa of aquatic insects

Identification of aquatic insects depended on the availability of specialists, therefore we focused on Ephemeroptera; suborders Nepomorpha and Gerromorpha of Hemiptera; families Elmidae, Epimetopidae, Hydrophilidae, and Torridincolidae of Coleoptera; Hemerodromiinae (aquatic Empididae) of Diptera; Odonata; Plecoptera; and Trichoptera. These focal taxa were identified at least in genus level, given that in some cases species-level identification was not possible because collected individuals were not adult males (immatures, subimagoes, or adult females), and, in other cases, because of the lack of comprehensive taxonomic revisions for particular genera. Higher-level classification followed the "Catálogo Taxonômico da Fauna do Brasil" (Lecci and Froehlich 2016, Monné et al. 2016, Pinto 2016, Rafael and Câmara 2016, Salles and Boldrini 2016, Santos et al. 2016).

### Study areas

Material examined in this paper were obtained during two collecting expeditions to the study areas, from April 18^th^ to 21^st^ of 2012 and February 7^th^ to 13^th^ of 2013 at Parque Nacional de Sete Cidades (PNSC, Piracuruca municipality, PI) and from Abril 21^st^ to 25^th^ of 2012 and February 13^th^ to 20^th^ of 2013 at Parque Nacional de Ubajara (PNU, Ubajara municipality, CE). Although both parks are included in the Caatinga Domain (Fig. 1), areas sampled within these parks seem to represent islands dominated by Cerrado (PNSC) or montane humid forest (PNU). The two parks are distant by approximately 110 km.

**Figure 3.**
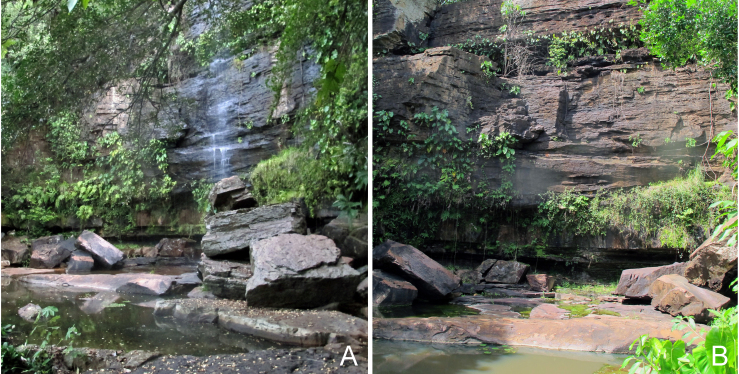
Cachoeira do Riachão (PNSC-02) at Parque Nacional de Sete Cidades, PI, Brazil. (A) April 2012. (B) February 2013.

**Figure 4.**
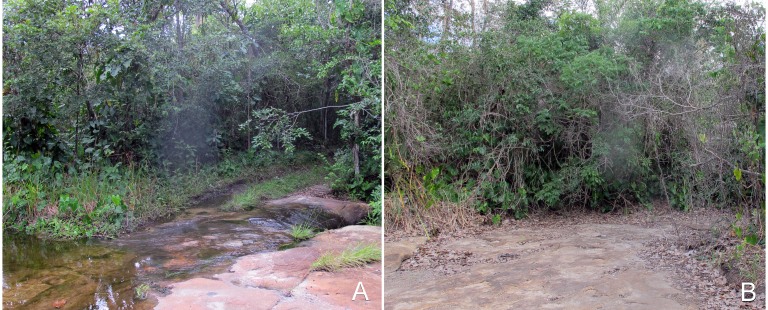
Riacho da Piedade (PNSC-04) at Parque Nacional de Sete Cidades, PI, Brazil. (A) April 2012. (B) February 2013.

Sampling localities within these parks were in or very close (less than 100 m) to bodies of water and are listed in Table 3. Bodies of water ranged from lentic and lotic systems and some streams sampled during the 2012 expedition to PNSC were completely dry in 2013 (see Figs 3, 4).

### Sampling methods

**Figure 5.**
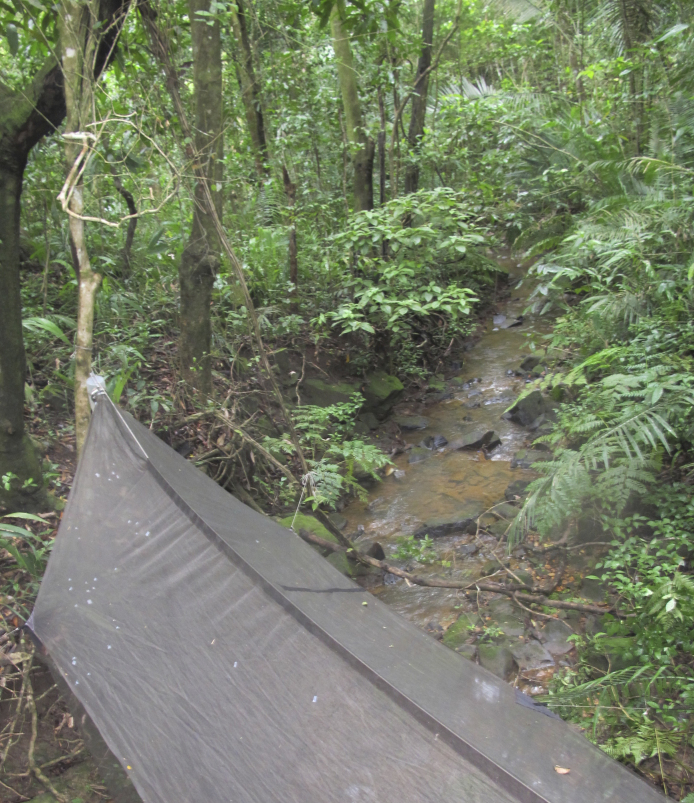
6-meter Malaise flight intercept trap over Rio Gameleira crossing Trilha Samambaia (PNU-01) at Parque Nacional de Ubajara, CE, Brazil.

**Figure 10.**
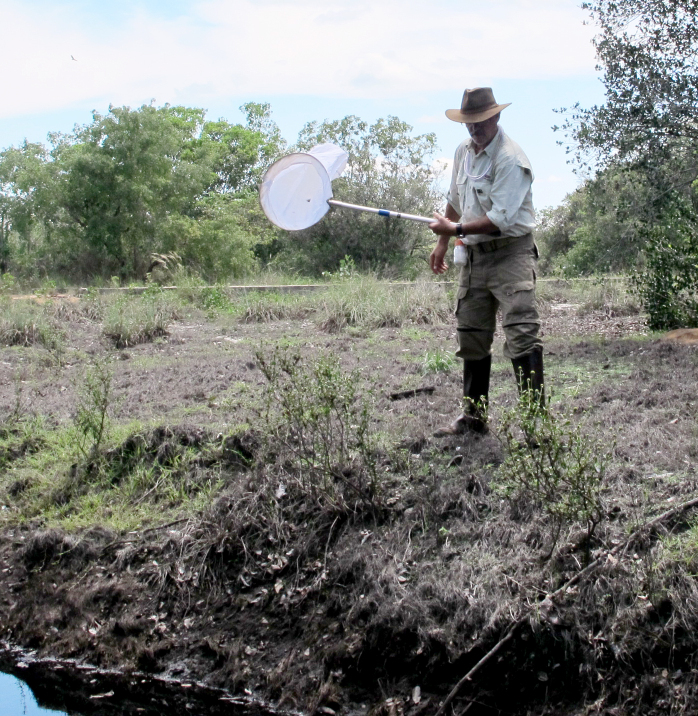
Manual collecting with entomological net near border of Riacho do Bananeira (PNSC-01), Parque Nacional de Sete Cidades, PI, Brazil.

**Figure 11.**
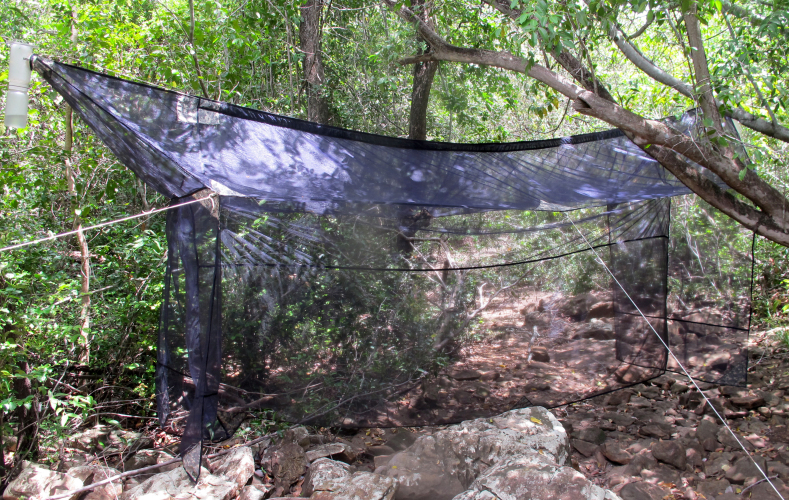
6-meter Malaise flight intercept trap over seasonally dried stream below Cachoeira do Riachão (PNSC-02) at Parque Nacional de Sete Cidades, PI, Brazil.

**Figure 12.**
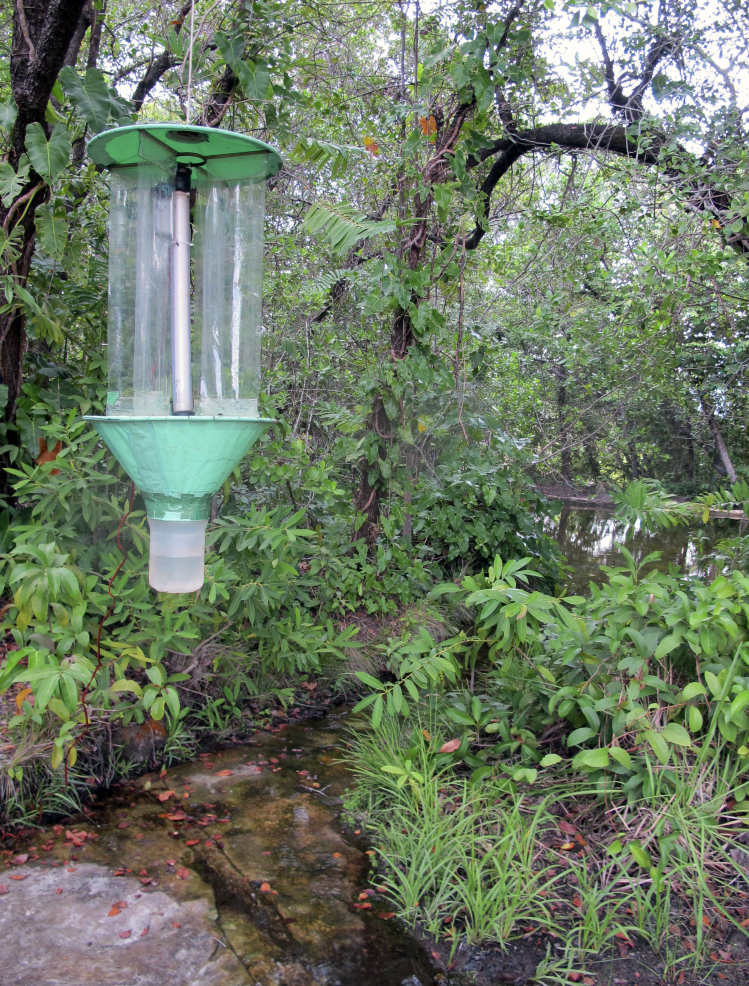
Pensylvania light trap near Olho d'água dos Milagres (PNSC-07) at Parque Nacional de Sete Cidades, PI, Brazil.

**Figure 14.**
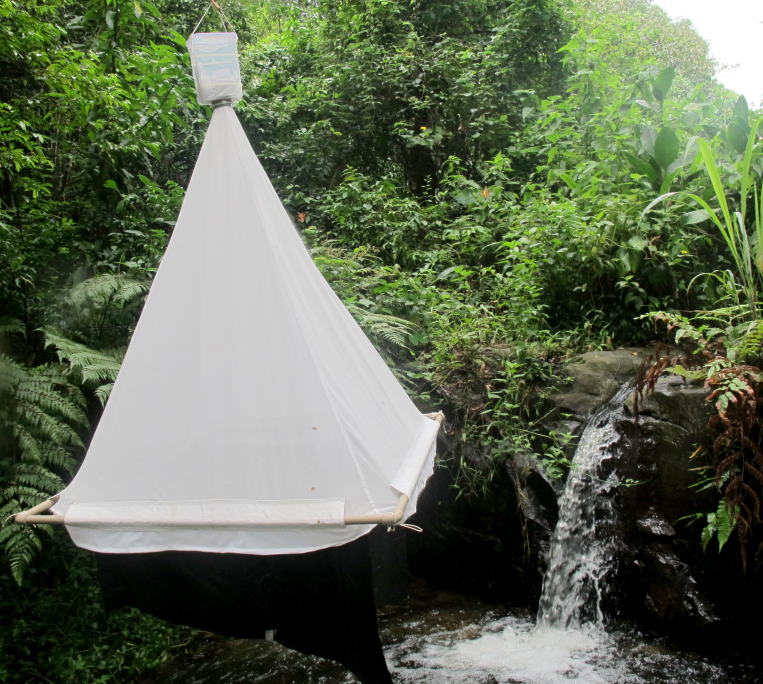
Suspended intercept trap over stream at Parque Nacional de Ubajara, CE, Brazil.

**Figure 15.**
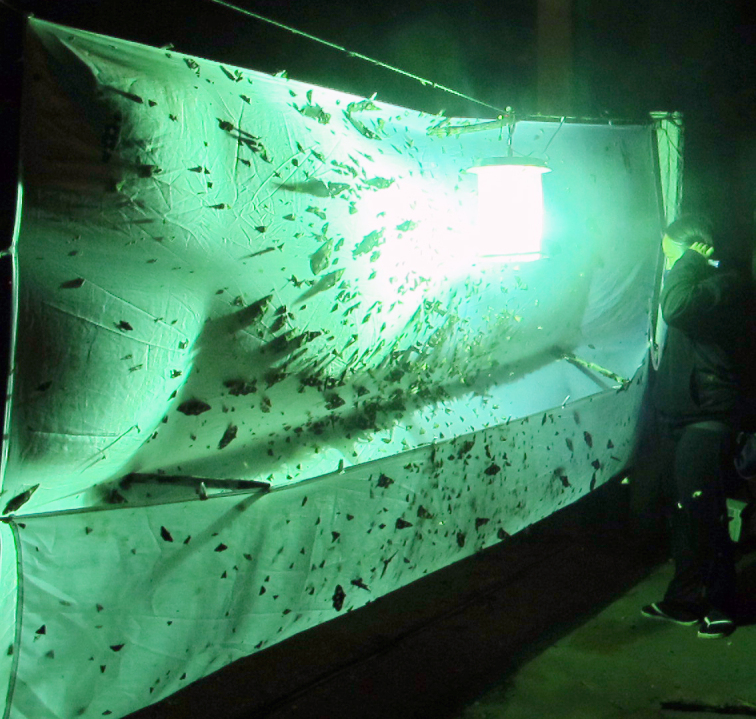
Manual collecting using white-sheet light trap at Parque Nacional de Ubajara, CE, Brazil.

**Figure 16.**
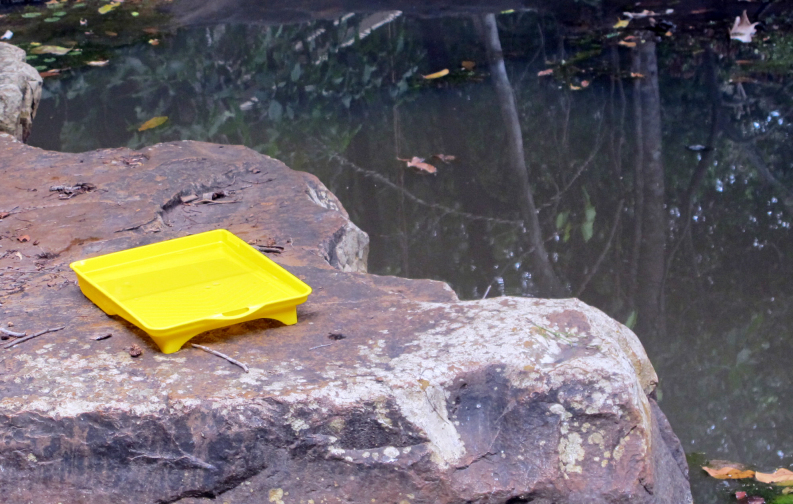
Yellow pan trap (YPT) near border of stream at Parque Nacional de Sete Cidades, PI, Brazil.

The following insects traps were used: 6-meter intercept Malaise traps (Figs 5, 11, Gressitt and Gressitt 1962), suspended intercept traps (Fig. 14, Rafael and Gorayeb 1982), light traps using a white sheet (Fig. 15) and Pennsylvania traps (Fig. 12, Frost 1957), yellow pan traps (Fig. 16), in addition to manual collecting using forceps or entomological nets (Fig. 10). Besides individuals collected during both expeditions, material was also collected by Malaise traps that were left in both parks with samples removed every month between expeditions. Material collected was divided and deposited in the following institutions: Coleção Entomológica Prof. José Alfredo Pinheiro Dutra, Departamento de Zoologia, Universidade Federal do Rio de Janeiro, Rio de Janeiro (DZRJ); Coleção Zoológica do Maranhão, Universidade Estadual do Maranhão, Caxias (CZMA); and Coleção de Invertebrados, Instituto Nacional de Pesquisas da Amazônia, Manaus (INPA).

### Data analyses

Species complementarity index for the two National parks were calculated with Cjk = Ujk/Sjk, where Ujk is the number of unique species in both sites and Sjk total richness in both sites combined (Colwell and Coddington 1994).

During the expeditions 27 samples (14 at PNSC and 13 at PNU) of Pennsylvania light traps placed over streams were collected. Each sample was the result of an approximately 6-8 hour effort of 15W fluorescent or UV light turned on at dusk. All individuals of Ephemeroptera, Plecoptera, Trichoptera, and Hydrophilidae (Coleoptera) were identified or morphotyped and counted. Adult females or subimagoes impossible to be identified into species level were treated as conspecific as males identified of the same genus on the sample (6 morphotypes from PNSC and 8 morphotypes from PNU of Ephemeroptera and Trichoptera). However, in cases where more than one species of the same genus was identified from the same sample (*Helicopsyche* and *Oxyethira* from PNU and *Chimarra*, *Neotrichia*, *Smicridea*, and *Oxyethira* from PNSC), the number of female individuals were equally divided among different species.

Quantitative samples of Pennsylvania light traps totalled 227 individuals of 23 species from PNU and 511 individuals of 54 species from PNSC of Ephemeroptera, Plecoptera, Trichoptera and Hydrophilidae (Coleoptera). Quantitative data served for comparisons of species richness between the two National Parks based on a rarefaction curve by individuals calculated in PAST 3.0 (Hammer et al. 2001).

## Data resources

### Data for species checklists of Parque Nacional de Ubajara (PNU) e Parque Nacional de Sete Cidades (PNSC)

**Figure 17.**
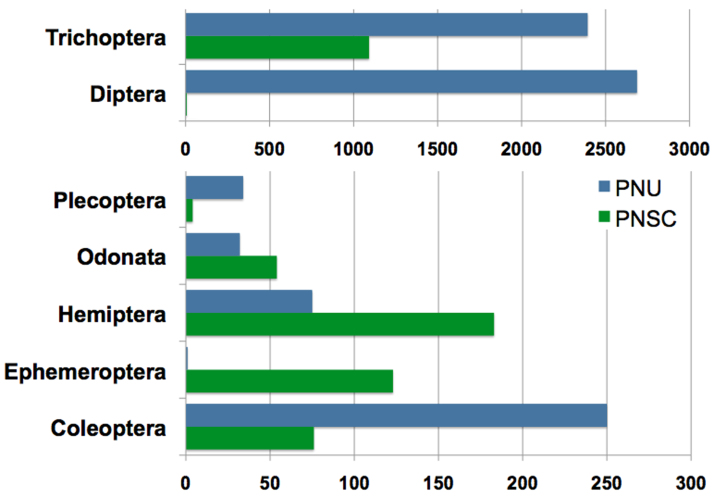
Number of individuals of aquatic insects identified from Ubajara (PNU, blue) and Sete Cidades (PNSC, green) National Parks.

Approximately 7,000 individuals of focal taxa were identified, being 5,472 from PNU and 1,539 from PNSC (Fig. 17).

**Figure 18.**
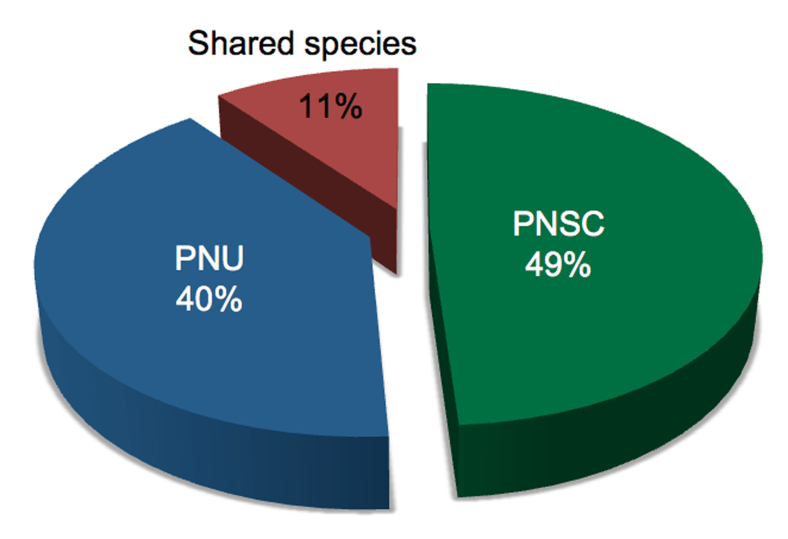
Percentage of total species of aquatic insects found only at Ubajara (PNU, blue), only at Sete Cidades (PNSC, green), and in both National Parks (red).

These individuals were identified into 96 species from PNU and 112 species from PNSC, while 20 of these species were found in both parks (Fig. 18). Species complementarity between both parks was of 89%.

The number of species per insect order in PNU and PNSC, respectively were: Coleoptera, 16 and 20; Diptera 10 and 1; Ephemeroptera, 1 and 9; Hemiptera, 9 and 20; Odonata, 21 and 21; Plecoptera, 1 and 1; and Trichoptera, 38 and 40. Species checklists for both National Parks are given below. Species marked with an asterisk (*) were found in both National Parks and distributional records (country or state) marked with and exclamation mark (!) are new records published herein. Species described or first taxa records based on material collected during this project are cited under Notes.

## Checklists

### Aquatic insects from Parque Nacional de Ubajara (PNU)

#### Coleoptera

#### Elmidae

##### Notes

New family record for CE.

#### Cylloepus

Erichson, 1847

##### Notes

New genus record for CE.

#### Cylloepus sp. 1

##### Materials

- **Type status:** Other material. **Occurrence:** recordedBy: Santos, A.P.M. | Takiya, D.M.; individualCount: 1; lifeStage: adult; **Location:** country: Brazil; stateProvince: Ceará; municipality: Ubajara; locality: Parque Nacional de Ubajara, Trilha Araticum, Rio das Minas; maximumElevationInMeters: 524; verbatimCoordinates: 3°50'3"S, 40°54'18"W; **Identification:** identifiedBy: Brunno H. L. Sampaio; **Event:** samplingProtocol: Manual; verbatimEventDate: 18.ii.13; **Record Level:** institutionCode: DZRJ; basisOfRecord: PreservedSpecimen

#### Macrelmis

Motschulsky, 1859

##### Notes

New genus record for CE.

#### Macrelmis sp. 1

##### Materials

- **Type status:** Other material. **Occurrence:** recordedBy: Santos, A.P.M. | Takiya, D.M.; individualCount: 2; lifeStage: immature; **Location:** country: Brazil; stateProvince: Ceará; municipality: Ubajara; locality: Parque Nacional de Ubajara, Trilha Araticum, Rio das Minas; maximumElevationInMeters: 524; verbatimCoordinates: 3°50'3"S, 40°54'18"W; **Identification:** identifiedBy: Brunno H. L. Sampaio; **Event:** samplingProtocol: Manual; verbatimEventDate: 18.ii.13; **Record Level:** institutionCode: DZRJ; basisOfRecord: PreservedSpecimen

#### Microcylloepus

Hinton, 1935

##### Notes

New genus record for CE.

#### Microcylloepus sp. 1

##### Materials

- **Type status:** Other material. **Occurrence:** recordedBy: Santos, A.P.M. | Takiya, D.M.; individualCount: 1; lifeStage: immature; **Location:** country: Brazil; stateProvince: Ceará; municipality: Ubajara; locality: Parque Nacional de Ubajara, Trilha Araticum, Rio das Minas; maximumElevationInMeters: 524; verbatimCoordinates: 3°50'3"S, 40°54'18"W; **Identification:** identifiedBy: Brunno H. L. Sampaio; **Event:** samplingProtocol: Manual; verbatimEventDate: 17.ii.13; **Record Level:** institutionCode: DZRJ; basisOfRecord: PreservedSpecimen
- **Type status:** Other material. **Occurrence:** recordedBy: Santos, A.P.M. | Takiya, D.M.; individualCount: 5; lifeStage: adult; **Location:** country: Brazil; stateProvince: Ceará; municipality: Ubajara; locality: Parque Nacional de Ubajara, Trilha Araticum, Rio das Minas; maximumElevationInMeters: 524; verbatimCoordinates: 3°50'3"S, 40°54'18"W; **Identification:** identifiedBy: Brunno H. L. Sampaio; **Event:** samplingProtocol: Manual; verbatimEventDate: 18.ii.13; **Record Level:** institutionCode: DZRJ; basisOfRecord: PreservedSpecimen
- **Type status:** Other material. **Occurrence:** recordedBy: Takiya, D.M.; individualCount: 1; lifeStage: immature; **Location:** country: Brazil; stateProvince: Ceará; municipality: Ubajara; locality: Parque Nacional de Ubajara, Trilha Araticum, Rio das Minas; maximumElevationInMeters: 524; verbatimCoordinates: 3°50'3"S, 40°54'18"W; **Identification:** identifiedBy: Brunno H. L. Sampaio; **Event:** samplingProtocol: Manual; verbatimEventDate: 23.iv.12; **Record Level:** institutionCode: DZRJ; basisOfRecord: PreservedSpecimen
- **Type status:** Other material. **Occurrence:** recordedBy: Takiya, D.M.; individualCount: 2; lifeStage: adult; **Location:** country: Brazil; stateProvince: Ceará; municipality: Ubajara; locality: Parque Nacional de Ubajara, Trilha Araticum, Rio das Minas; maximumElevationInMeters: 524; verbatimCoordinates: 3°50'3"S, 40°54'18"W; **Identification:** identifiedBy: Brunno H. L. Sampaio; **Event:** samplingProtocol: Manual; verbatimEventDate: 23.iv.12; **Record Level:** institutionCode: DZRJ; basisOfRecord: PreservedSpecimen

#### Epimetopidae

##### Notes

New family record for CE.

#### Epimetopus

Lacordaire, 1854

##### Notes

New genus record for CE.

#### Epimetopus sp. 1

##### Materials

- **Type status:** Other material. **Occurrence:** recordedBy: Santos, A.P.M. | Takiya, D.M.; individualCount: 1; sex: male; lifeStage: adult; **Location:** country: Brazil; stateProvince: Ceará; municipality: Ubajara; locality: Parque Nacional de Ubajara, Cachoeira do Cafundó; maximumElevationInMeters: 783; verbatimCoordinates: 3°50'12"S, 40°54'35"W; **Identification:** identifiedBy: Bruno Clarkson; **Event:** samplingProtocol: Pennsylvania light trap; verbatimEventDate: 15.ii.13; **Record Level:** institutionCode: DZRJ; basisOfRecord: PreservedSpecimen

##### Notes

Undescribed species in the lanceolatus group.

#### Hydrophilidae

#### Australocyon

Hansen, 1990

##### Notes

New genus record for CE.

#### Australocyon sp. 1

##### Materials

- **Type status:** Other material. **Occurrence:** recordedBy: Takiya, D.M.; individualCount: 1; sex: male; lifeStage: adult; **Location:** country: Brazil; stateProvince: Ceará; municipality: Ubajara; locality: Parque Nacional de Ubajara, Cachoeira do Cafundó; maximumElevationInMeters: 783; verbatimCoordinates: 3°50'12"S, 40°54'35"W; **Identification:** identifiedBy: Bruno Clarkson; **Event:** samplingProtocol: Pennsylvania light trap; verbatimEventDate: 24.iv.12; **Record Level:** institutionCode: DZRJ; basisOfRecord: PreservedSpecimen

##### Notes

Undescribed species.

#### Chasmogenus

Sharp, 1882

##### Notes

New genus record for CE.

#### Chasmogenus (Chasmogenus) sp. 1*

##### Materials

- **Type status:** Other material. **Occurrence:** recordedBy: Santos, A.P.M. | Takiya, D.M.; individualCount: 4; lifeStage: adult; **Location:** country: Brazil; stateProvince: Ceará; municipality: Ubajara; locality: Parque Nacional de Ubajara, Trilha Araticum, Rio das Minas na altura da trilha do teleférico; maximumElevationInMeters: 420; verbatimCoordinates: 3°49'58"S, 40°53'53"W; **Identification:** identifiedBy: Bruno Clarkson; **Event:** samplingProtocol: Malaise intercept trap; verbatimEventDate: 14.ii.13; **Record Level:** institutionCode: DZRJ; basisOfRecord: PreservedSpecimen

#### Enochrus

Thomson, 1859

##### Notes

New genus record for CE.

#### Enochrus (Methydrus) melanthus

Orchymont, 1943

##### Materials

- **Type status:** Other material. **Occurrence:** recordedBy: Santos, A.P.M. | Takiya, D.M.; individualCount: 9; lifeStage: adult; **Location:** country: Brazil; stateProvince: Ceará; municipality: Ubajara; locality: Parque Nacional de Ubajara, Trilha Araticum, Rio das Minas na altura da trilha do teleférico; maximumElevationInMeters: 420; verbatimCoordinates: 3°49'58"S, 40°53'53"W; **Identification:** identifiedBy: Bruno Clarkson; **Event:** samplingProtocol: Malaise intercept trap; verbatimEventDate: 14.ii.13; **Record Level:** institutionCode: DZRJ; basisOfRecord: PreservedSpecimen
- **Type status:** Other material. **Occurrence:** recordedBy: Santos, A.P.M. | Takiya, D.M.; individualCount: 1; sex: female; lifeStage: adult; **Location:** country: Brazil; stateProvince: Ceará; municipality: Ubajara; locality: Parque Nacional de Ubajara, Portão Neblina; maximumElevationInMeters: 849; verbatimCoordinates: 3°50'18"S, 40°53'54"W; **Identification:** identifiedBy: Bruno Clarkson; **Event:** samplingProtocol: Malaise intercept trap; verbatimEventDate: 14.ii.13; **Record Level:** institutionCode: DZRJ; basisOfRecord: PreservedSpecimen
- **Type status:** Other material. **Occurrence:** recordedBy: Santos, A.P.M. | Takiya, D.M.; individualCount: 1; sex: male; lifeStage: adult; **Location:** country: Brazil; stateProvince: Ceará; municipality: Ubajara; locality: Parque Nacional de Ubajara, Rio Cafundó, pouco acima da cachoeira; maximumElevationInMeters: 795; verbatimCoordinates: 3°50'13"S, 40°54'35"W; **Identification:** identifiedBy: Bruno Clarkson; **Event:** samplingProtocol: Malaise intercept trap; verbatimEventDate: 13.ii.13; **Record Level:** institutionCode: DZRJ; basisOfRecord: PreservedSpecimen

##### Distribution

Brazil: CE!, PE, MS, SP.

##### Notes

New species record for CE.

#### Helochares

Mulsant, 1844

##### Notes

New genus record for CE.

#### Helochares (Helochares) sp. 1*

##### Materials

- **Type status:** Other material. **Occurrence:** recordedBy: Santos, A.P.M. | Takiya, D.M.; individualCount: 1; lifeStage: immature; **Location:** country: Brazil; stateProvince: Ceará; municipality: Ubajara; locality: Parque Nacional de Ubajara, Trilha Araticum, Rio das Minas na altura da trilha do teleférico; maximumElevationInMeters: 420; verbatimCoordinates: 3°49'58"S, 40°53'53"W; **Identification:** identifiedBy: Bruno Clarkson; **Event:** samplingProtocol: Manual; verbatimEventDate: 19.ii.13; **Record Level:** institutionCode: DZRJ; basisOfRecord: PreservedSpecimen

#### Hemiosus

Sharp, 1882

#### Hemiosus varidius

Orchymont, 1940

##### Materials

- **Type status:** Other material. **Occurrence:** recordedBy: Santos, A.P.M. | Takiya, D.M.; individualCount: 1; sex: female; lifeStage: adult; **Location:** country: Brazil; stateProvince: Ceará; municipality: Ubajara; locality: Parque Nacional de Ubajara, Trilha Araticum, Rio da Minas abaixo do teleférico; maximumElevationInMeters: 395; verbatimCoordinates: 3°49'43.3"S, 40°53'51.5"W; **Identification:** identifiedBy: Bruno Clarkson; **Event:** samplingProtocol: Manual; verbatimEventDate: 14.ii.13; **Record Level:** institutionCode: DZRJ; basisOfRecord: PreservedSpecimen

##### Distribution

Brazil: CE!, PE.

##### Notes

New species record for CE.

#### Oocyclus

Sharp, 1882

##### Notes

New genus record for CE.

#### Oocyclus schubarti

Orchymont, 1940

##### Materials

- **Type status:** Other material. **Occurrence:** recordedBy: Santos, A.P.M. | Takiya, D.M.; individualCount: 2; sex: female; lifeStage: adult; **Location:** country: Brazil; stateProvince: Ceará; municipality: Ubajara; locality: Parque Nacional de Ubajara, Trilha Araticum, Rio das Minas; maximumElevationInMeters: 524; verbatimCoordinates: 3°50'3"S, 40°54'18"W; **Identification:** identifiedBy: Bruno Clarkson; **Event:** samplingProtocol: Manual; verbatimEventDate: 18.ii.13; **Record Level:** institutionCode: DZRJ; basisOfRecord: PreservedSpecimen
- **Type status:** Other material. **Occurrence:** recordedBy: Santos, A.P.M. | Takiya, D.M.; individualCount: 13; sex: male; lifeStage: adult; **Location:** country: Brazil; stateProvince: Ceará; municipality: Ubajara; locality: Parque Nacional de Ubajara, Trilha Araticum, Rio das Minas; maximumElevationInMeters: 524; verbatimCoordinates: 3°50'3"S, 40°54'18"W; **Identification:** identifiedBy: Bruno Clarkson; **Event:** samplingProtocol: Manual; verbatimEventDate: 18.ii.13; **Record Level:** institutionCode: DZRJ; basisOfRecord: PreservedSpecimen
- **Type status:** Other material. **Occurrence:** recordedBy: Santos, A.P.M. | Takiya, D.M.; individualCount: 15; sex: female; lifeStage: adult; **Location:** country: Brazil; stateProvince: Ceará; municipality: Ubajara; locality: Parque Nacional de Ubajara, Trilha Araticum, Rio das Minas; maximumElevationInMeters: 524; verbatimCoordinates: 3°50'3"S, 40°54'18"W; **Identification:** identifiedBy: Bruno Clarkson; **Event:** samplingProtocol: Manual; verbatimEventDate: 18.ii.13; **Record Level:** institutionCode: DZRJ; basisOfRecord: PreservedSpecimen
- **Type status:** Other material. **Occurrence:** recordedBy: Santos, A.P.M. | Takiya, D.M.; individualCount: 3; lifeStage: immature; **Location:** country: Brazil; stateProvince: Ceará; municipality: Ubajara; locality: Parque Nacional de Ubajara, Trilha Araticum, Rio das Minas; maximumElevationInMeters: 524; verbatimCoordinates: 3°50'3"S, 40°54'18"W; **Identification:** identifiedBy: Bruno Clarkson; **Event:** samplingProtocol: Manual; verbatimEventDate: 18.ii.13; **Record Level:** institutionCode: DZRJ; basisOfRecord: PreservedSpecimen

##### Distribution

Brazil: PI!, CE!, AL. Argentina?

##### Notes

New species record for CE.

#### Paracymus

Thomson, 1867

#### Paracymus rufocinctus

Bruch, 1915

##### Materials

- **Type status:** Other material. **Occurrence:** recordedBy: Rafael, J.A. | Limeira-de-Oliveira, F. | Takiya, D.M. | Santos, A.P.M. | et al.; individualCount: 1; sex: male; lifeStage: adult; **Location:** country: Brazil; stateProvince: Ceará; municipality: Ubajara; locality: Parque Nacional de Ubajara, Rio Cafundó, pouco acima da cachoeira; maximumElevationInMeters: 795; verbatimCoordinates: 3°50'13"S, 40°54'35"W; **Identification:** identifiedBy: Bruno Clarkson; **Event:** samplingProtocol: Malaise intercept trap; verbatimEventDate: 13.ii.13; **Record Level:** institutionCode: DZRJ; basisOfRecord: PreservedSpecimen

##### Distribution

Widespread in South America. Brazil: PI, CE, PE. Argentina.

#### Phaenostoma

Orchymont, 1937

##### Notes

New genus record for CE.

#### Phaenostoma sp. 1

##### Materials

- **Type status:** Other material. **Occurrence:** recordedBy: Santos, A.P.M. | Takiya, D.M.; individualCount: 1; sex: male; lifeStage: adult; **Location:** country: Brazil; stateProvince: Ceará; municipality: Ubajara; locality: Parque Nacional de Ubajara, Ponte sobre Rio Miranda; maximumElevationInMeters: 792; verbatimCoordinates: 3°50'7.4"S, 40°54'47.5"W; **Identification:** identifiedBy: Bruno Clarkson; **Event:** samplingProtocol: Pennsylvania light trap; verbatimEventDate: 15.ii.13; **Record Level:** institutionCode: DZRJ; basisOfRecord: PreservedSpecimen

##### Notes

Undescribed species.

#### Phaenonotum

Sharp, 1882

#### Phaenonotum sp. 1

##### Materials

- **Type status:** Other material. **Occurrence:** recordedBy: Takiya, D.M. | Santos, A.P.M.; individualCount: 1; sex: female; lifeStage: adult; **Location:** country: Brazil; stateProvince: Ceará; municipality: Ubajara; locality: Parque Nacional de Ubajara, Rio das Minas, próximo ao Portão Araticum; maximumElevationInMeters: 328; verbatimCoordinates: 3°49'32.6"S, 40°53'32.8"W; **Identification:** identifiedBy: Bruno Clarkson; **Event:** samplingProtocol: Malaise intercept trap; verbatimEventDate: 14.ii.13; **Record Level:** institutionCode: DZRJ; basisOfRecord: PreservedSpecimen

#### Tropisternus

Solier, 1834

#### Tropisternus (Pristoternus) flavipalpis

Sharp, 1883

##### Materials

- **Type status:** Other material. **Occurrence:** recordedBy: Santos, A.P.M. | Takiya, D.M.; individualCount: 1; sex: female; lifeStage: adult; **Location:** country: Brazil; stateProvince: Ceará; municipality: Ubajara; locality: Parque Nacional de Ubajara, Ponte sobre Rio Miranda; maximumElevationInMeters: 792; verbatimCoordinates: 3°50'7.4"S, 40°54'47.5"W; **Identification:** identifiedBy: Bruno Clarkson; **Event:** samplingProtocol: Pennsylvania light trap; verbatimEventDate: 15.ii.13; **Record Level:** institutionCode: DZRJ; basisOfRecord: PreservedSpecimen

##### Distribution

Mexico. Brazil: CE!, RJ.

##### Notes

New species record for Northeastern Brazil.

#### Torridincolidae

##### Notes

New family record for CE.

#### Claudiella

Reichardt & Vanin, 1976

##### Notes

New genus record for CE.

#### Claudiella sp. 1

##### Materials

- **Type status:** Other material. **Occurrence:** recordedBy: Santos, A.P.M. | Takiya, D.M.; individualCount: 21; lifeStage: adult; **Location:** country: Brazil; stateProvince: Ceará; municipality: Ubajara; locality: Parque Nacional de Ubajara, Trilha Araticum, Rio Cafundó; maximumElevationInMeters: 753; verbatimCoordinates: 3°50'12"S, 40°54'31"W; **Identification:** identifiedBy: Brunno H. L. Sampaio; **Event:** samplingProtocol: Manual; verbatimEventDate: 17.ii.13; **Record Level:** institutionCode: DZRJ; basisOfRecord: PreservedSpecimen
- **Type status:** Other material. **Occurrence:** recordedBy: Santos, A.P.M. | Takiya, D.M.; individualCount: 69; lifeStage: adult; **Location:** country: Brazil; stateProvince: Ceará; municipality: Ubajara; locality: Parque Nacional de Ubajara, Trilha Araticum, Rio das Minas; maximumElevationInMeters: 524; verbatimCoordinates: 3°50'3"S, 40°54'18"W; **Identification:** identifiedBy: Brunno H. L. Sampaio; **Event:** samplingProtocol: Manual; verbatimEventDate: 18.ii.13; **Record Level:** institutionCode: DZRJ; basisOfRecord: PreservedSpecimen
- **Type status:** Other material. **Occurrence:** recordedBy: Takiya, D.M.; individualCount: 1; lifeStage: adult; **Location:** country: Brazil; stateProvince: Ceará; municipality: Ubajara; locality: Parque Nacional de Ubajara, Trilha Araticum, Rio das Minas; maximumElevationInMeters: 524; verbatimCoordinates: 3°50'3"S, 40°54'18"W; **Identification:** identifiedBy: Brunno H. L. Sampaio; **Event:** samplingProtocol: Manual; verbatimEventDate: 22.iv.12; **Record Level:** institutionCode: DZRJ; basisOfRecord: PreservedSpecimen
- **Type status:** Other material. **Occurrence:** recordedBy: Takiya, D.M.; individualCount: 5; lifeStage: adult; **Location:** country: Brazil; stateProvince: Ceará; municipality: Ubajara; locality: Parque Nacional de Ubajara, Trilha Araticum, Rio das Minas; maximumElevationInMeters: 524; verbatimCoordinates: 3°50'3"S, 40°54'18"W; **Identification:** identifiedBy: Brunno H. L. Sampaio; **Event:** samplingProtocol: Manual; verbatimEventDate: 23.iv.12; **Record Level:** institutionCode: DZRJ; basisOfRecord: PreservedSpecimen
- **Type status:** Other material. **Occurrence:** recordedBy: Takiya, D.M.; individualCount: 3; lifeStage: immature; **Location:** country: Brazil; stateProvince: Ceará; municipality: Ubajara; locality: Parque Nacional de Ubajara, Rio Cafundó, pouco acima da cachoeira; maximumElevationInMeters: 795; verbatimCoordinates: 3°50'13"S, 40°54'35"W; **Identification:** identifiedBy: Brunno H. L. Sampaio; **Event:** samplingProtocol: Manual; verbatimEventDate: 23.iv.12; **Record Level:** institutionCode: DZRJ; basisOfRecord: PreservedSpecimen
- **Type status:** Other material. **Occurrence:** recordedBy: Takiya, D.M.; individualCount: 19; lifeStage: adult; **Location:** country: Brazil; stateProvince: Ceará; municipality: Ubajara; locality: Parque Nacional de Ubajara, Rio Cafundó, pouco acima da cachoeira; maximumElevationInMeters: 795; verbatimCoordinates: 3°50'13"S, 40°54'35"W; **Identification:** identifiedBy: Brunno H. L. Sampaio; **Event:** samplingProtocol: Manual; verbatimEventDate: 23.iv.12; **Record Level:** institutionCode: DZRJ; basisOfRecord: PreservedSpecimen

##### Notes

**Figure 19.**
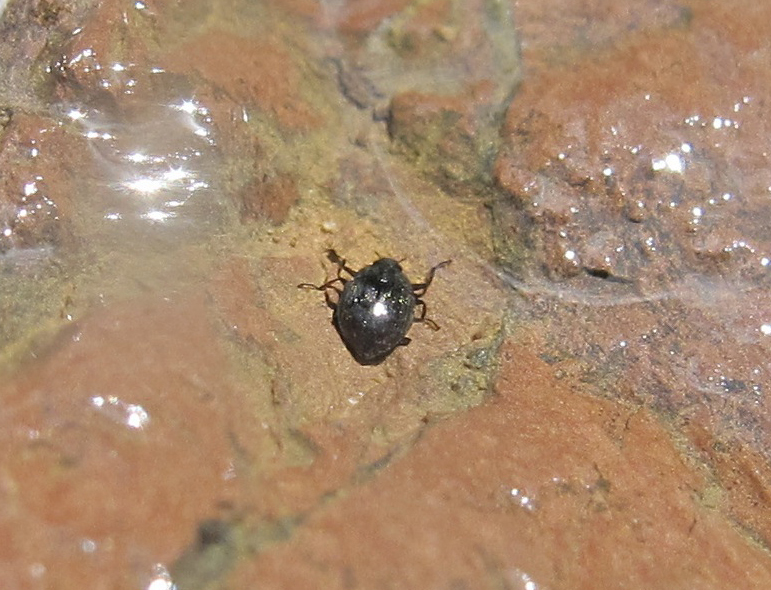
Specimen of *Claudiella* sp. 1 collected at PNU-02, an undescribed species representing the first record of Torridincolidae for Ceará.

Undescribed species. See Fig. 19.

#### Ytu

Reichardt, 1973

##### Notes

New genus record for CE.

#### Ytu sp. 1

##### Materials

- **Type status:** Other material. **Occurrence:** recordedBy: Santos, A.P.M. | Takiya, D.M.; individualCount: 64; lifeStage: adult; **Location:** country: Brazil; stateProvince: Ceará; municipality: Ubajara; locality: Parque Nacional de Ubajara, Trilha Araticum, Rio das Minas; maximumElevationInMeters: 524; verbatimCoordinates: 3°50'3"S, 40°54'18"W; **Identification:** identifiedBy: Brunno H. L. Sampaio; **Event:** samplingProtocol: Manual; verbatimEventDate: 18.ii.13; **Record Level:** institutionCode: DZRJ; basisOfRecord: PreservedSpecimen

##### Notes

Undescribed species.

#### Diptera

#### Empididae

#### Hemerodromiinae

##### Notes

Subfamily firstly recorded from CE in Câmara et al. 2015.

#### Hemerodromia

Meigen, 1823

##### Notes

Genus firstly recorded from CE in Câmara et al. 2015​.

#### Hemerodromia brevicercata

Câmara, Takiya, Plant & Rafael, 2015

##### Materials

- **Type status:** Other material. **Occurrence:** recordedBy: Rafael, J.A. | Limeira-de-Oliveira, F. | Takiya, D.M. | Santos, A.P.M. | et al.; individualCount: 258; lifeStage: adult; **Location:** country: Brazil; stateProvince: Ceará; municipality: Ubajara; locality: Parque Nacional de Ubajara, Rio Cafundó, pouco acima da cachoeira; maximumElevationInMeters: 795; verbatimCoordinates: 3°50'13"S, 40°54'35"W; **Identification:** identifiedBy: Josenir Teixeira Câmara; **Event:** samplingProtocol: Malaise intercept trap; verbatimEventDate: 13.ii.13; **Record Level:** institutionCode: DZRJ; basisOfRecord: PreservedSpecimen

##### Distribution

Brazil: CE.

##### Notes

**Figure 20.**
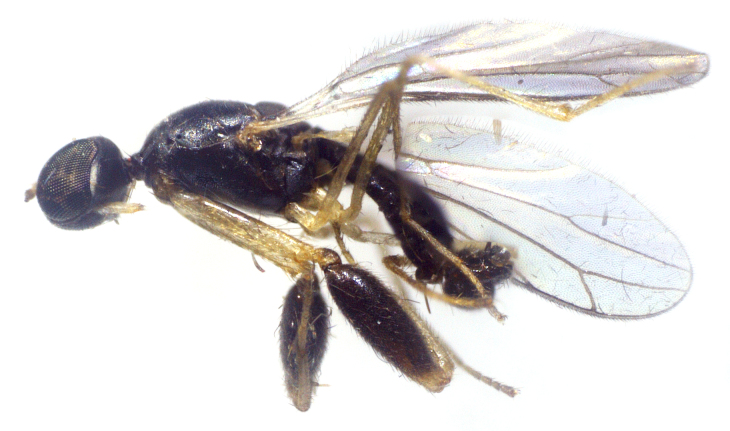
Lateral habitus of male holotype of *Hemerodromia brevicercata* collected at PNU-07 (CZMA), species described based on material collected during this project.

Species described in Câmara et al. 2015. See Fig. 20.

#### Hemerodromia membranosa

Câmara, Takiya, Plant & Rafael, 2015

##### Materials

- **Type status:** Other material. **Occurrence:** recordedBy: Rafael, J.A. | Limeira-de-Oliveira, F. | Takiya, D.M. | Santos, A.P.M. | et al.; individualCount: 33; lifeStage: adult; **Location:** country: Brazil; stateProvince: Ceará; municipality: Ubajara; locality: Parque Nacional de Ubajara, Rio Cafundó, pouco acima da cachoeira; maximumElevationInMeters: 795; verbatimCoordinates: 3°50'13"S, 40°54'35"W; **Identification:** identifiedBy: Josenir Teixeira Câmara; **Event:** samplingProtocol: Malaise intercept trap; verbatimEventDate: 14.ii.13; **Record Level:** institutionCode: DZRJ; basisOfRecord: PreservedSpecimen

##### Distribution

Brazil: CE.

##### Notes

Species described in Câmara et al. 2015.

#### Hemerodromia mesomelaena

Bezzi, 1909

##### Materials

- **Type status:** Other material. **Occurrence:** recordedBy: Rafael, J.A. | Limeira-de-Oliveira, F. | Takiya, D.M. | Santos, A.P.M. | et al.; individualCount: 15; lifeStage: adult; **Location:** country: Brazil; stateProvince: Ceará; municipality: Ubajara; locality: Parque Nacional de Ubajara, Rio Cafundó, pouco acima da cachoeira; maximumElevationInMeters: 795; verbatimCoordinates: 3°50'13"S, 40°54'35"W; **Identification:** identifiedBy: Josenir Teixeira Câmara; **Event:** samplingProtocol: Malaise intercept trap; verbatimEventDate: 14.ii.13; **Record Level:** institutionCode: DZRJ; basisOfRecord: PreservedSpecimen

##### Distribution

Brazil: PI!, CE!, PR. Peru. Argentina.

##### Notes

New species record for Northeastern Brazil.

#### Hemerodromia mourai

Câmara, Takiya, Plant & Rafael, 2015

##### Materials

- **Type status:** Other material. **Occurrence:** recordedBy: Rafael, J.A. | Limeira-de-Oliveira, F. | Takiya, D.M. | Santos, A.P.M. | et al.; individualCount: 18; lifeStage: adult; **Location:** country: Brazil; stateProvince: Ceará; municipality: Ubajara; locality: Parque Nacional de Ubajara, Rio Cafundó, pouco acima da cachoeira; maximumElevationInMeters: 795; verbatimCoordinates: 3°50'13"S, 40°54'35"W; **Identification:** identifiedBy: Josenir Teixeira Câmara; **Event:** samplingProtocol: Malaise intercept trap; verbatimEventDate: 14.ii.13; **Record Level:** institutionCode: DZRJ; basisOfRecord: PreservedSpecimen

##### Distribution

Brazil: CE.

##### Notes

**Figure 21.**
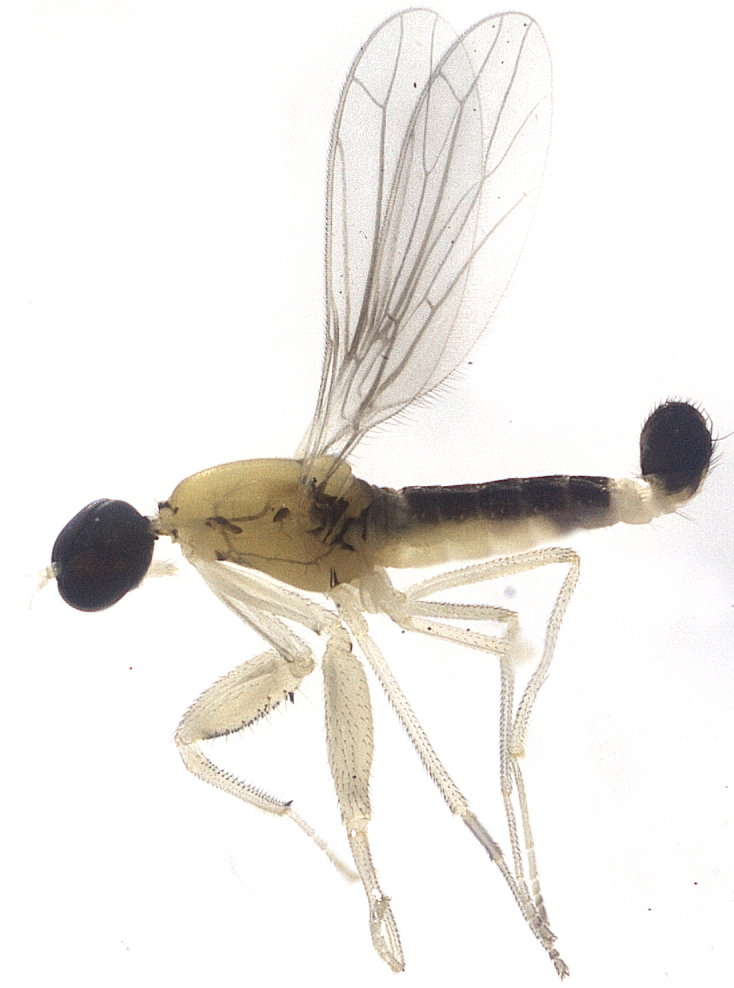
Lateral habitus of male holotype of *Hemerodromia mourai* collected at PNU-07 (CZMA), species described based on material collected during this project.

Species described in Câmara et al. 2015. See Fig. 21.

#### Hemerodromia ubajaraensis

Câmara, Takiya, Plant & Rafael, 2015

##### Materials

- **Type status:** Other material. **Occurrence:** recordedBy: Rafael, J.A. | Limeira-de-Oliveira, F. | Takiya, D.M. | Santos, A.P.M. | et al.; individualCount: 5; lifeStage: adult; **Location:** country: Brazil; stateProvince: Ceará; municipality: Ubajara; locality: Parque Nacional de Ubajara, Rio Cafundó, pouco acima da cachoeira; maximumElevationInMeters: 795; verbatimCoordinates: 3°50'13"S, 40°54'35"W; **Identification:** identifiedBy: Josenir Teixeira Câmara; **Event:** samplingProtocol: Malaise intercept trap; verbatimEventDate: 18.ii.13; **Record Level:** institutionCode: DZRJ; basisOfRecord: PreservedSpecimen

##### Distribution

Brazil: CE.

##### Notes

**Figure 22.**
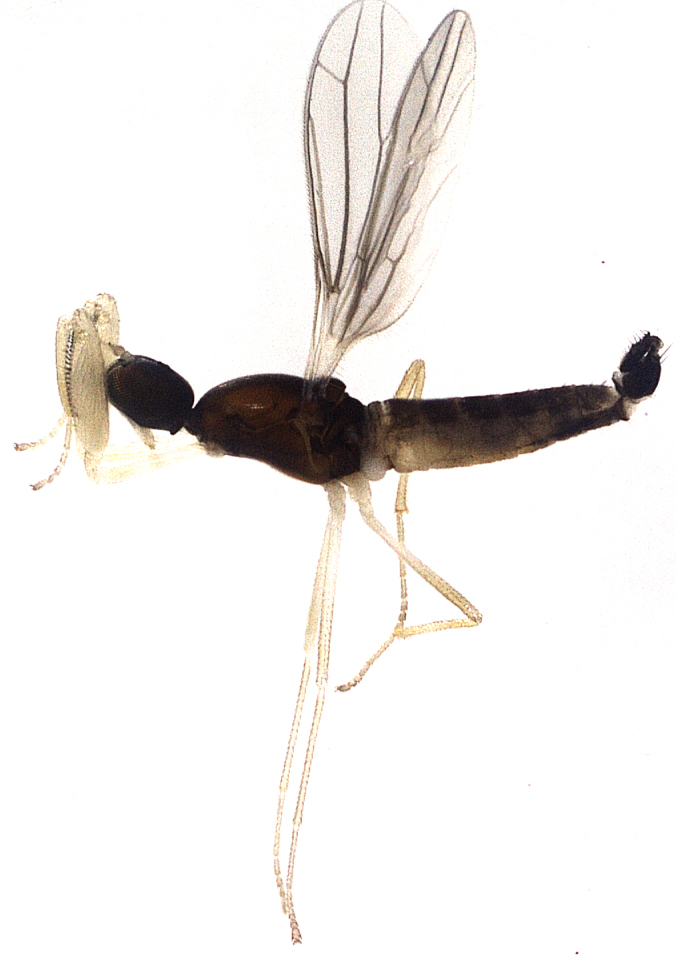
Lateral habitus of male holotype of *Hemerodromia ubajaraensis* collected at PNU-07 (CZMA), species described based on material collected during this project.

Species described in Câmara et al. 2015. See Fig. 22.

#### Hemerodromia sp. 1

##### Materials

- **Type status:** Other material. **Occurrence:** recordedBy: Rafael, J.A. | Limeira-de-Oliveira, F. | Takiya, D.M. | Santos, A.P.M. | et al.; individualCount: 3; lifeStage: adult; **Location:** country: Brazil; stateProvince: Ceará; municipality: Ubajara; locality: Parque Nacional de Ubajara, Rio Cafundó, pouco acima da cachoeira; maximumElevationInMeters: 795; verbatimCoordinates: 3°50'13"S, 40°54'35"W; **Identification:** identifiedBy: Josenir Teixeira Câmara; **Event:** samplingProtocol: Malaise intercept trap; verbatimEventDate: 14.ii.13; **Record Level:** institutionCode: DZRJ; basisOfRecord: PreservedSpecimen

##### Notes

Undescribed species.

#### Metachela

Coquillett, 1903

##### Notes

New genus record for CE.

#### Metachela sp. 1

##### Materials

- **Type status:** Other material. **Occurrence:** recordedBy: Rafael, J.A. | Limeira-de-Oliveira, F. | Takiya, D.M. | et al.; individualCount: 28; lifeStage: adult; **Location:** country: Brazil; stateProvince: Ceará; municipality: Ubajara; locality: Parque Nacional de Ubajara, Rio Cafundó, pouco acima da cachoeira; maximumElevationInMeters: 795; verbatimCoordinates: 3°50'13"S, 40°54'35"W; **Identification:** identifiedBy: Josenir Teixeira Câmara; **Event:** samplingProtocol: Malaise intercept trap; verbatimEventDate: 24.iv.12; **Record Level:** institutionCode: DZRJ; basisOfRecord: PreservedSpecimen

##### Notes

Undescribed species.

#### Metachela sp. 2

##### Materials

- **Type status:** Other material. **Occurrence:** recordedBy: Rafael, J.A. | Limeira-de-Oliveira, F. | Takiya, D.M. | Santos, A.P.M. | et al.; individualCount: 15; lifeStage: adult; **Location:** country: Brazil; stateProvince: Ceará; municipality: Ubajara; locality: Parque Nacional de Ubajara, Rio Cafundó, pouco acima da cachoeira; maximumElevationInMeters: 795; verbatimCoordinates: 3°50'13"S, 40°54'35"W; **Identification:** identifiedBy: Josenir Teixeira Câmara; **Event:** samplingProtocol: Malaise intercept trap; verbatimEventDate: 14.ii.13; **Record Level:** institutionCode: DZRJ; basisOfRecord: PreservedSpecimen

##### Notes

Undescribed species.

#### Neoplasta

Coquillett, 1895

##### Notes

New genus record for CE.

#### Neoplasta sp. 1

##### Materials

- **Type status:** Other material. **Occurrence:** recordedBy: Rafael, J.A. | Limeira-de-Oliveira, F. | Takiya, D.M. | Santos, A.P.M. | et al.; individualCount: 1986; lifeStage: adult; **Location:** country: Brazil; stateProvince: Ceará; municipality: Ubajara; locality: Parque Nacional de Ubajara, Rio Cafundó, pouco acima da cachoeira; maximumElevationInMeters: 795; verbatimCoordinates: 3°50'13"S, 40°54'35"W; **Identification:** identifiedBy: Josenir Teixeira Câmara; **Event:** samplingProtocol: Malaise intercept trap; verbatimEventDate: 18.ii.13; **Record Level:** institutionCode: DZRJ; basisOfRecord: PreservedSpecimen

##### Notes

Undescribed species.

#### Neoplasta sp. 2

##### Materials

- **Type status:** Other material. **Occurrence:** recordedBy: Rafael, J.A. | Limeira-de-Oliveira, F. | Takiya, D.M. | et al.; individualCount: 326; lifeStage: adult; **Location:** country: Brazil; stateProvince: Ceará; municipality: Ubajara; locality: Parque Nacional de Ubajara, Rio Cafundó, pouco acima da cachoeira; maximumElevationInMeters: 795; verbatimCoordinates: 3°50'13"S, 40°54'35"W; **Identification:** identifiedBy: Josenir Teixeira Câmara; **Event:** samplingProtocol: Malaise intercept trap; verbatimEventDate: 24.iv.12; **Record Level:** institutionCode: DZRJ; basisOfRecord: PreservedSpecimen

##### Notes

Undescribed species.

#### Ephemeroptera

#### Leptophlebiidae

##### Notes

New family record for CE.

#### Farrodes

Peters, 1971

##### Notes

New genus record for CE.

#### Farrodes tepui

Domínguez, Molineri & Peters, 1996

##### Materials

- **Type status:** Other material. **Occurrence:** recordedBy: Santos, A.P.M. | Takiya, D.M.; individualCount: 1; sex: male; lifeStage: immature; **Location:** country: Brazil; stateProvince: Ceará; municipality: Ubajara; locality: Parque Nacional de Ubajara, Trilha Araticum, Rio das Minas na altura da trilha do teleférico; maximumElevationInMeters: 420; verbatimCoordinates: 3°49'58"S, 40°53'53"W; **Identification:** identifiedBy: Inês Corrêa Gonçalves; **Event:** samplingProtocol: Pennsylvania light trap; verbatimEventDate: 14.ii.13; **Record Level:** institutionCode: DZRJ; basisOfRecord: PreservedSpecimen

##### Distribution

Venezuela. Brazil: PI!, CE!, PE.

##### Notes

New species record for CE.

#### Hemiptera

#### Gerromorpha

#### Gerridae

#### Limnogonus

Stål, 1868

##### Notes

Genus firstly recorded from CE in Cordeiro and Moreira 2015.

#### Limnogonus profugus

Drake & Harris, 1930

##### Materials

- **Type status:** Other material. **Occurrence:** recordedBy: Santos, A.P.M. | Takiya, D.M.; individualCount: 1; sex: female; lifeStage: adult; **Location:** country: Brazil; stateProvince: Ceará; municipality: Ubajara; locality: Parque Nacional de Ubajara, Trilha Araticum, Rio das Minas; maximumElevationInMeters: 524; verbatimCoordinates: 3°50'3"S, 40°54'18"W; **Identification:** identifiedBy: Felipe F. F. Moreira; **Event:** samplingProtocol: Manual; verbatimEventDate: 14.ii.13; **Record Level:** institutionCode: DZRJ; basisOfRecord: PreservedSpecimen

##### Distribution

Brazil: CE, PB, PE, MT, GO, MG, MS, SP, RJ. Peru. Paraguay. Argentina.

##### Notes

Species firstly recorded from CE in Cordeiro and Moreira 2015​.

#### Veliidae

#### Rhagovelia

Mayr, 1865

##### Notes

Genus firstly recorded from CE in Cordeiro and Moreira 2015.

#### Rhagovelia whitei

(Breddin, 1898)

##### Materials

- **Type status:** Other material. **Occurrence:** recordedBy: Takiya, D.M.; individualCount: 1; sex: male; lifeStage: adult; **Location:** country: Brazil; stateProvince: Ceará; municipality: Ubajara; locality: Parque Nacional de Ubajara, Trilha Araticum, Rio das Minas; maximumElevationInMeters: 524; verbatimCoordinates: 3°50'3"S, 40°54'18"W; **Identification:** identifiedBy: Felipe F. F. Moreira; **Event:** samplingProtocol: Manual; verbatimEventDate: 23.iv.12; **Record Level:** institutionCode: DZRJ; basisOfRecord: PreservedSpecimen
- **Type status:** Other material. **Occurrence:** recordedBy: Takiya, D.M.; individualCount: 1; sex: female; lifeStage: adult; **Location:** country: Brazil; stateProvince: Ceará; municipality: Ubajara; locality: Parque Nacional de Ubajara, Trilha Araticum, Rio das Minas; maximumElevationInMeters: 524; verbatimCoordinates: 3°50'3"S, 40°54'18"W; **Identification:** identifiedBy: Felipe F. F. Moreira; **Event:** samplingProtocol: Manual; verbatimEventDate: 23.iv.12; **Record Level:** institutionCode: DZRJ; basisOfRecord: PreservedSpecimen

##### Distribution

Brazil: PA, CE, MA, MT, GO, MG, MS, SP. Paraguay.

##### Notes

Species firstly recorded from CE in Cordeiro and Moreira 2015.

#### Rhagovelia sp. 1

##### Materials

- **Type status:** Other material. **Occurrence:** recordedBy: Takiya, D.M.; individualCount: 12; lifeStage: immature; **Location:** country: Brazil; stateProvince: Ceará; municipality: Ubajara; locality: Parque Nacional de Ubajara, Trilha Araticum, Rio das Minas; maximumElevationInMeters: 524; verbatimCoordinates: 3°50'3"S, 40°54'18"W; **Identification:** identifiedBy: Felipe F. F. Moreira; **Event:** samplingProtocol: Manual; verbatimEventDate: 17.ii.13; **Record Level:** institutionCode: DZRJ; basisOfRecord: PreservedSpecimen
- **Type status:** Other material. **Occurrence:** recordedBy: Takiya, D.M.; individualCount: 1; sex: female; lifeStage: adult; **Location:** country: Brazil; stateProvince: Ceará; municipality: Ubajara; locality: Parque Nacional de Ubajara, Trilha Samambaia, Rio Gameleira; maximumElevationInMeters: 874; verbatimCoordinates: 3°50'25"S, 40°54'19"W; **Identification:** identifiedBy: Felipe F. F. Moreira; **Event:** samplingProtocol: Manual; verbatimEventDate: 24.iv.12; **Record Level:** institutionCode: DZRJ; basisOfRecord: PreservedSpecimen

#### Nepomorpha

#### Belostomatidae

#### Belostoma

Latreille, 1807

#### Belostoma sp. 1*

##### Materials

- **Type status:** Other material. **Occurrence:** recordedBy: Takiya, D.M.; individualCount: 4; lifeStage: immature; **Location:** country: Brazil; stateProvince: Ceará; municipality: Ubajara; locality: Parque Nacional de Ubajara, Trilha Samambaia, Rio Gameleira; maximumElevationInMeters: 874; verbatimCoordinates: 3°50'25"S, 40°54'19"W; **Identification:** identifiedBy: Julianna Freires Barbosa; **Event:** samplingProtocol: Manual; verbatimEventDate: 21.iv.12; **Record Level:** institutionCode: DZRJ; basisOfRecord: PreservedSpecimen

#### Corixidae

#### Tenagobia

Bergroth, 1899

#### Tenagobia sp. 1

##### Materials

- **Type status:** Other material. **Occurrence:** recordedBy: Takiya, D.M. | Câmara, J.T.; individualCount: 2; sex: male; lifeStage: adult; **Location:** country: Brazil; stateProvince: Ceará; municipality: Ubajara; locality: Parque Nacional de Ubajara, Trilha Samambaia, Rio Gameleira; maximumElevationInMeters: 874; verbatimCoordinates: 3°50'25"S, 40°54'19"W; **Identification:** identifiedBy: Julianna Freires Barbosa; **Event:** samplingProtocol: Pennsylvania light trap; verbatimEventDate: 21.iv.12; **Record Level:** institutionCode: DZRJ; basisOfRecord: PreservedSpecimen
- **Type status:** Other material. **Occurrence:** recordedBy: Takiya, D.M. | Câmara, J.T.; individualCount: 7; sex: female; lifeStage: adult; **Location:** country: Brazil; stateProvince: Ceará; municipality: Ubajara; locality: Parque Nacional de Ubajara, Trilha Samambaia, Rio Gameleira; maximumElevationInMeters: 874; verbatimCoordinates: 3°50'25"S, 40°54'19"W; **Identification:** identifiedBy: Julianna Freires Barbosa; **Event:** samplingProtocol: Pennsylvania light trap; verbatimEventDate: 21.iv.12; **Record Level:** institutionCode: DZRJ; basisOfRecord: PreservedSpecimen

#### Gelastocoridae

##### Notes

New family record for CE.

#### Gelastocoris

Kirkaldy, 1897

##### Notes

New genus record for CE.

#### Gelastocoris angulatus

(Melin, 1929)

##### Materials

- **Type status:** Other material. **Occurrence:** recordedBy: Santos, A.P.M. | Takiya, D.M.; individualCount: 1; sex: male; lifeStage: adult; **Location:** country: Brazil; stateProvince: Ceará; municipality: Ubajara; locality: Parque Nacional de Ubajara, Cachoeira do Cafundó; maximumElevationInMeters: 783; verbatimCoordinates: 3°50'12"S, 40°54'35"W; **Identification:** identifiedBy: Julianna Freires Barbosa; **Event:** samplingProtocol: Manual; verbatimEventDate: 16.ii.13; **Record Level:** institutionCode: DZRJ; basisOfRecord: PreservedSpecimen
- **Type status:** Other material. **Occurrence:** recordedBy: Câmara, J.T.; individualCount: 1; sex: male; lifeStage: adult; **Location:** country: Brazil; stateProvince: Ceará; municipality: Ubajara; locality: Parque Nacional de Ubajara, Trilha Araticum, Rio das Minas; maximumElevationInMeters: 524; verbatimCoordinates: 3°50'3"S, 40°54'18"W; **Identification:** identifiedBy: Julianna Freires Barbosa; **Event:** samplingProtocol: YPT; verbatimEventDate: 22.iv.12; **Record Level:** institutionCode: DZRJ; basisOfRecord: PreservedSpecimen

##### Distribution

Guyana. Suriname. Brazil: PA, AM, CE!, MT, BA, GO, MG, SP, RJ. Bolivia. Paraguay. Argentina.

##### Notes

New species record for CE.

#### Gelastocoris sp.

##### Materials

- **Type status:** Other material. **Occurrence:** recordedBy: Takiya, D.M.; individualCount: 1; lifeStage: immature; **Location:** country: Brazil; stateProvince: Ceará; municipality: Ubajara; locality: Parque Nacional de Ubajara, Trilha Araticum, Rio das Minas; maximumElevationInMeters: 524; verbatimCoordinates: 3°50'3"S, 40°54'18"W; **Identification:** identifiedBy: Julianna Freires Barbosa; **Event:** samplingProtocol: Manual; verbatimEventDate: 22.iv.12; **Record Level:** institutionCode: DZRJ; basisOfRecord: PreservedSpecimen
- **Type status:** Other material. **Occurrence:** recordedBy: Câmara, J.T.; individualCount: 1; sex: male; lifeStage: adult; **Location:** country: Brazil; stateProvince: Ceará; municipality: Ubajara; locality: Parque Nacional de Ubajara, Trilha Araticum, Rio das Minas; maximumElevationInMeters: 524; verbatimCoordinates: 3°50'3"S, 40°54'18"W; **Identification:** identifiedBy: Julianna Freires Barbosa; **Event:** samplingProtocol: YPT; verbatimEventDate: 22.iv.12; **Record Level:** institutionCode: DZRJ; basisOfRecord: PreservedSpecimen

#### Naucoridae

##### Notes

New family record for CE.

#### Limnocoris

Stål, 1858

##### Notes

New genus record for CE.

#### Limnocoris sp. 1

##### Materials

- **Type status:** Other material. **Occurrence:** recordedBy: Santos, A.P.M. | Takiya, D.M.; individualCount: 3; sex: female; lifeStage: adult; **Location:** country: Brazil; stateProvince: Ceará; municipality: Ubajara; locality: Parque Nacional de Ubajara, Trilha Araticum, Rio das Minas; maximumElevationInMeters: 524; verbatimCoordinates: 3°50'3"S, 40°54'18"W; **Identification:** identifiedBy: Julianna Freires Barbosa; **Event:** samplingProtocol: Manual; verbatimEventDate: 14.ii.13; **Record Level:** institutionCode: DZRJ; basisOfRecord: PreservedSpecimen

#### Notonectidae

#### Martarega

White, 1879

#### Martarega bentoi

Truxal, 1949

##### Materials

- **Type status:** Other material. **Occurrence:** recordedBy: Santos, A.P.M. | Takiya, D.M.; individualCount: 9; sex: male; lifeStage: adult; **Location:** country: Brazil; stateProvince: Ceará; municipality: Ubajara; locality: Parque Nacional de Ubajara, Cachoeira do Cafundó; maximumElevationInMeters: 783; verbatimCoordinates: 3°50'12"S, 40°54'35"W; **Identification:** identifiedBy: Julianna Freires Barbosa; **Event:** samplingProtocol: Manual; verbatimEventDate: 16.ii.13; **Record Level:** institutionCode: DZRJ; basisOfRecord: PreservedSpecimen
- **Type status:** Other material. **Occurrence:** recordedBy: Santos, A.P.M. | Takiya, D.M.; individualCount: 3; sex: female; lifeStage: adult; **Location:** country: Brazil; stateProvince: Ceará; municipality: Ubajara; locality: Parque Nacional de Ubajara, Cachoeira do Cafundó; maximumElevationInMeters: 783; verbatimCoordinates: 3°50'12"S, 40°54'35"W; **Identification:** identifiedBy: Julianna Freires Barbosa; **Event:** samplingProtocol: Manual; verbatimEventDate: 16.ii.13; **Record Level:** institutionCode: DZRJ; basisOfRecord: PreservedSpecimen
- **Type status:** Other material. **Occurrence:** recordedBy: Santos, A.P.M. | Takiya, D.M.; individualCount: 28; lifeStage: immature; **Location:** country: Brazil; stateProvince: Ceará; municipality: Ubajara; locality: Parque Nacional de Ubajara, Cachoeira do Cafundó; maximumElevationInMeters: 783; verbatimCoordinates: 3°50'12"S, 40°54'35"W; **Identification:** identifiedBy: Julianna Freires Barbosa; **Event:** samplingProtocol: Manual; verbatimEventDate: 16.ii.13; **Record Level:** institutionCode: DZRJ; basisOfRecord: PreservedSpecimen

##### Distribution

Brazil: PI, CE!, PE, MT, MG, RJ. Argentina.

##### Notes

New species record for CE.

#### Odonata

#### Anisoptera

#### Aeshnidae

#### Castoraeschna

Calvert, 1952

#### Castoraeschna corbeti

Carvalho, Pinto & Ferreira-Jr, 2009

##### Materials

- **Type status:** Other material. **Occurrence:** recordedBy: Rafael, J.A. | Limeira-de-Oliveira, F. | Takiya, D.M. | Santos, A.P.M. | et al.; individualCount: 1; sex: male; lifeStage: adult; **Location:** country: Brazil; stateProvince: Ceará; municipality: Ubajara; locality: Parque Nacional de Ubajara, Rio das Minas, próximo ao Portão Araticum; maximumElevationInMeters: 328; verbatimCoordinates: 3°49'32.6"S, 40°53'32.8"W; **Identification:** identifiedBy: Alcimar do Lago Carvalho | Ângelo Parise Pinto; **Event:** samplingProtocol: Malaise intercept trap; verbatimEventDate: 16.ii.13; **Record Level:** institutionCode: DZRJ; basisOfRecord: PreservedSpecimen
- **Type status:** Other material. **Occurrence:** recordedBy: Santos, A.P.M. | Takiya, D.M.; individualCount: 1; sex: male; lifeStage: adult; **Location:** country: Brazil; stateProvince: Ceará; municipality: Ubajara; locality: Parque Nacional de Ubajara, Trilha Araticum, Rio das Minas; maximumElevationInMeters: 524; verbatimCoordinates: 3°50'3"S, 40°54'18"W; **Identification:** identifiedBy: Alcimar do Lago Carvalho | Ângelo Parise Pinto; **Event:** samplingProtocol: Manual; verbatimEventDate: 18.ii.13; **Record Level:** institutionCode: DZRJ; basisOfRecord: PreservedSpecimen

##### Distribution

Brazil: PA, CE!.

##### Notes

New species record for CE.

#### Gynacantha

Rambur, 1842

#### Gynacantha nervosa

Rambur, 1842

##### Materials

- **Type status:** Other material. **Occurrence:** recordedBy: Santos, A.P.M. | Takiya, D.M.; individualCount: 1; sex: female; lifeStage: adult; **Location:** country: Brazil; stateProvince: Ceará; municipality: Ubajara; locality: Parque Nacional de Ubajara, Portão Neblina; maximumElevationInMeters: 849; verbatimCoordinates: 3°50'18"S, 40°53'54"W; **Identification:** identifiedBy: Ângelo Parise Pinto; **Event:** samplingProtocol: Manual; verbatimEventDate: 17.ii.13; **Record Level:** institutionCode: DZRJ; basisOfRecord: PreservedSpecimen

##### Distribution

USA south to Panama. Trinidad and Tobago. Colombia. Venezuela. Guyana. Suriname. French Guiana. Brazil: RR, PI!, CE, PE, MT, MG, MS, ES, RJ. Ecuador. Peru. Bolivia.

#### Gomphidae

#### Progomphus

Selys, 1854

#### Progomphus complicatus

Selys, 1854

##### Materials

- **Type status:** Other material. **Occurrence:** recordedBy: Rafael, J.A. | Limeira-de-Oliveira, F. | Takiya, D.M. | Santos, A.P.M. | et al.; individualCount: 1; sex: male; lifeStage: adult; **Location:** country: Brazil; stateProvince: Ceará; municipality: Ubajara; locality: Parque Nacional de Ubajara, Trilha Araticum, Rio das Minas na altura da trilha do teleférico; maximumElevationInMeters: 420; verbatimCoordinates: 3°49'58"S, 40°53'53"W; **Identification:** identifiedBy: Ângelo Parise Pinto | Marcus Vinicius O. de Almeida; **Event:** samplingProtocol: Malaise intercept trap; verbatimEventDate: 14.ii.13; **Record Level:** institutionCode: CZMA; basisOfRecord: PreservedSpecimen
- **Type status:** Other material. **Occurrence:** recordedBy: Rafael, J.A. | Limeira-de-Oliveira, F. | Takiya, D.M. | Santos, A.P.M. | et al.; individualCount: 1; sex: male; lifeStage: adult; **Location:** country: Brazil; stateProvince: Ceará; municipality: Ubajara; locality: Parque Nacional de Ubajara, Trilha Araticum, Rio das Minas na altura da trilha do teleférico; maximumElevationInMeters: 420; verbatimCoordinates: 3°49'58"S, 40°53'53"W; **Identification:** identifiedBy: Ângelo Parise Pinto | Marcus Vinicius O. de Almeida; **Event:** samplingProtocol: Malaise intercept trap; verbatimEventDate: 14.ii.13; **Record Level:** institutionCode: DZRJ; basisOfRecord: PreservedSpecimen
- **Type status:** Other material. **Occurrence:** recordedBy: Rafael, J.A. | Limeira-de-Oliveira, F. | Takiya, D.M. | Santos, A.P.M. | et al.; individualCount: 1; sex: female; lifeStage: adult; **Location:** country: Brazil; stateProvince: Ceará; municipality: Ubajara; locality: Parque Nacional de Ubajara, Rio das Minas, próximo ao Portão Araticum; maximumElevationInMeters: 328; verbatimCoordinates: 3°49'32.6"S, 40°53'32.8"W; **Identification:** identifiedBy: Ângelo Parise Pinto | Marcus Vinicius O. de Almeida; **Event:** samplingProtocol: Malaise intercept trap; verbatimEventDate: 14.ii.13; **Record Level:** institutionCode: DZRJ; basisOfRecord: PreservedSpecimen

##### Distribution

Brazil: CE, BA, MG, ES, SP, RJ, SC, RS. Paraguay. Argentina.

##### Notes

Species firstly recorded from CE in De Almeida et al. 2013.

#### Progomphus dorsopallidus

Byers, 1934

##### Materials

- **Type status:** Other material. **Occurrence:** recordedBy: Rafael, J.A. | Limeira-de-Oliveira, F. | Takiya, D.M. | Santos, A.P.M. | et al.; individualCount: 1; sex: female; lifeStage: adult; **Location:** country: Brazil; stateProvince: Ceará; municipality: Ubajara; locality: Parque Nacional de Ubajara, Trilha Araticum, Rio das Minas na altura da trilha do teleférico; maximumElevationInMeters: 420; verbatimCoordinates: 3°49'58"S, 40°53'53"W; **Identification:** identifiedBy: Ângelo Parise Pinto | Marcus Vinicius O. de Almeida; **Event:** samplingProtocol: Malaise intercept trap; verbatimEventDate: 14.ii.13; **Record Level:** institutionCode: CZMA; basisOfRecord: PreservedSpecimen
- **Type status:** Other material. **Occurrence:** recordedBy: Rafael, J.A. | Limeira-de-Oliveira, F. | Takiya, D.M. | Santos, A.P.M. | et al.; individualCount: 1; sex: female; lifeStage: adult; **Location:** country: Brazil; stateProvince: Ceará; municipality: Ubajara; locality: Parque Nacional de Ubajara, Trilha Araticum, Rio das Minas na altura da trilha do teleférico; maximumElevationInMeters: 420; verbatimCoordinates: 3°49'58"S, 40°53'53"W; **Identification:** identifiedBy: Ângelo Parise Pinto | Marcus Vinicius O. de Almeida; **Event:** samplingProtocol: Malaise intercept trap; verbatimEventDate: 14.ii.13; **Record Level:** institutionCode: DZRJ; basisOfRecord: PreservedSpecimen
- **Type status:** Other material. **Occurrence:** recordedBy: Rafael, J.A. | Limeira-de-Oliveira, F. | Takiya, D.M. | Santos, A.P.M. | et al.; individualCount: 1; sex: female; lifeStage: adult; **Location:** country: Brazil; stateProvince: Ceará; municipality: Ubajara; locality: Parque Nacional de Ubajara, Rio das Minas, próximo ao Portão Araticum; maximumElevationInMeters: 328; verbatimCoordinates: 3°49'32.6"S, 40°53'32.8"W; **Identification:** identifiedBy: Ângelo Parise Pinto | Marcus Vinicius O. de Almeida; **Event:** samplingProtocol: Malaise intercept trap; verbatimEventDate: 14.ii.13; **Record Level:** institutionCode: DZRJ; basisOfRecord: PreservedSpecimen

##### Distribution

Trinidad and Tobago. Venezuela. Guyana. Brazil: CE, ES.

#### Libellulidae

#### Brechmorhoga

Kirby, 1889

#### Brechmorhoga nubecula

(Rambur, 1842)

##### Materials

- **Type status:** Other material. **Occurrence:** recordedBy: Santos, A.P.M. | Takiya, D.M.; individualCount: 1; sex: male; lifeStage: adult; **Location:** country: Brazil; stateProvince: Ceará; municipality: Ubajara; locality: Parque Nacional de Ubajara, Trilha Araticum, Rio das Minas; maximumElevationInMeters: 524; verbatimCoordinates: 3°50'3"S, 40°54'18"W; **Identification:** identifiedBy: Ângelo Parise Pinto; **Event:** samplingProtocol: Manual; verbatimEventDate: 18.ii.13; **Record Level:** institutionCode: DZRJ; basisOfRecord: PreservedSpecimen

##### Distribution

Mexico. Belize. Costa Rica. Panama. Trinidad and Tobago. Colombia. Venezuela. Brazil: CE!, MT, BA, MG, ES, SP, RJ, SC. Ecuador. Peru. Paraguay. Argentina

##### Notes

New species record for CE.

#### Dasythemis

Karsch, 1889

#### Dasythemis esmeralda

Ris, 1910

##### Materials

- **Type status:** Other material. **Occurrence:** recordedBy: Takiya, D.M. | Cavichioli, R.R.; individualCount: 1; sex: male; lifeStage: adult; **Location:** country: Brazil; stateProvince: Ceará; municipality: Ubajara; locality: Parque Nacional de Ubajara, Trilha Samambaia, Rio Gameleira; maximumElevationInMeters: 874; verbatimCoordinates: 3°50'25"S, 40°54'19"W; **Identification:** identifiedBy: Alcimar do Lago Carvalho | Ângelo Parise Pinto; **Event:** samplingProtocol: Manual; verbatimEventDate: 24.iv.12; **Record Level:** institutionCode: DZRJ; basisOfRecord: PreservedSpecimen

##### Distribution

Trinidad and Tobago. Colombia. Venezuela. Suriname. French Guiana. Brazil: AM, CE, PE, MT, RO, GO, MS, SP, RJ. Ecuador. Peru. Bolivia.

#### Dythemis

Hagen, 1861

#### Dythemis nigra

Martin, 1897

##### Materials

- **Type status:** Other material. **Occurrence:** recordedBy: Rafael, J.A. | Limeira-de-Oliveira, F. | Takiya, D.M. | Santos, A.P.M. | et al.; individualCount: 1; sex: female; lifeStage: adult; **Location:** country: Brazil; stateProvince: Ceará; municipality: Ubajara; locality: Parque Nacional de Ubajara, Rio das Minas, próximo ao Portão Araticum; maximumElevationInMeters: 328; verbatimCoordinates: 3°49'32.6"S, 40°53'32.8"W; **Identification:** identifiedBy: Ângelo Parise Pinto | Marcus Vinicius O. de Almeida; **Event:** samplingProtocol: Malaise intercept trap; verbatimEventDate: 14.ii.13; **Record Level:** institutionCode: CZMA; basisOfRecord: PreservedSpecimen
- **Type status:** Other material. **Occurrence:** recordedBy: Santos, A.P.M. | Takiya, D.M.; individualCount: 1; sex: male; lifeStage: adult; **Location:** country: Brazil; stateProvince: Ceará; municipality: Ubajara; locality: Parque Nacional de Ubajara, Trilha Araticum, Rio das Minas; maximumElevationInMeters: 524; verbatimCoordinates: 3°50'3"S, 40°54'18"W; **Identification:** identifiedBy: Ângelo Parise Pinto | Marcus Vinicius O. de Almeida; **Event:** samplingProtocol: Manual; verbatimEventDate: 18.ii.13; **Record Level:** institutionCode: DZRJ; basisOfRecord: PreservedSpecimen

##### Distribution

Mexico south to Panama. Trinidad and Tobago. Colombia. Venezuela. Guyana. Suriname. French Guiana. Brazil: RR, PA, AM, CE, PE, MT, RO, BA, GO, MG, MS, ES, SP, RJ, SC. Ecuador. Peru. Paraguay. Argentina.

#### Erythrodiplax

Brauer, 1868

#### Erythrodiplax basalis

(Kirby, 1897)

##### Materials

- **Type status:** Other material. **Occurrence:** recordedBy: Takiya, D.M. | Cavichioli, R.R.; individualCount: 1; sex: male; lifeStage: adult; **Location:** country: Brazil; stateProvince: Ceará; municipality: Ubajara; locality: Parque Nacional de Ubajara, Trilha Samambaia, Rio Gameleira; maximumElevationInMeters: 874; verbatimCoordinates: 3°50'25"S, 40°54'19"W; **Identification:** identifiedBy: Ângelo Parise Pinto; **Event:** samplingProtocol: Manual; verbatimEventDate: 24.iv.12; **Record Level:** institutionCode: DZRJ; basisOfRecord: PreservedSpecimen

##### Distribution

Trinidad and Tobago. Colombia. Venezuela. Guyana. Suriname. French Guiana. Brazil: RR, PA, AM, MA, PI!, CE, PE, MT, RO, BA, MG, MS, ES, SP, RJ, RS. Ecuador. Peru. Bolivia. Paraguay. Argentina. Uruguay.

#### Erythrodiplax castanea

(Burmeister, 1839)

##### Materials

- **Type status:** Other material. **Occurrence:** recordedBy: Takiya, D.M. | Cavichioli, R.R.; individualCount: 1; sex: male; lifeStage: adult; **Location:** country: Brazil; stateProvince: Ceará; municipality: Ubajara; locality: Parque Nacional de Ubajara, Trilha Samambaia, Rio Gameleira; maximumElevationInMeters: 874; verbatimCoordinates: 3°50'25"S, 40°54'19"W; **Identification:** identifiedBy: Ângelo Parise Pinto; **Event:** samplingProtocol: Manual; verbatimEventDate: 24.iv.12; **Record Level:** institutionCode: DZRJ; basisOfRecord: PreservedSpecimen

##### Distribution

Belize. Guatemala. Costa Rica. Trinidad and Tobago. Colombia. Venezuela. Guyana. Suriname. French Guiana. Brazil: PA, AM, CE!, PE, MT, RO, BA, GO, MS, ES, SP, RJ, SC. Ecuador. Peru. Bolivia. Paraguay. Argentina.

##### Notes

New species record for CE.

#### Erythrodiplax fusca

(Rambur, 1842)

##### Materials

- **Type status:** Other material. **Occurrence:** recordedBy: Santos, A.P.M. | Takiya, D.M.; individualCount: 1; sex: male; lifeStage: adult; **Location:** country: Brazil; stateProvince: Ceará; municipality: Ubajara; locality: Parque Nacional de Ubajara, Trilha Araticum, Rio das Minas; maximumElevationInMeters: 524; verbatimCoordinates: 3°50'3"S, 40°54'18"W; **Identification:** identifiedBy: Ângelo Parise Pinto; **Event:** samplingProtocol: Manual; verbatimEventDate: 18.ii.13; **Record Level:** institutionCode: DZRJ; basisOfRecord: PreservedSpecimen

##### Distribution

USA south to Panama. Trinidad and Tobago. Colombia. Venezuela. Guyana. Suriname. French Guiana. Brazil: PA, AM, MA, PI, CE, RN, PE, MT, RO, BA, GO, MG, MS, ES, SP, RJ, SC, RS. Ecuador. Peru. Bolivia. Paraguay. Argentina. Uruguay.

#### Erythrodiplax paraguayensis

(Förster, 1904)

##### Materials

- **Type status:** Other material. **Occurrence:** recordedBy: Câmara, J.T.; individualCount: 1; sex: female; lifeStage: adult; **Location:** country: Brazil; stateProvince: Ceará; municipality: Ubajara; locality: Parque Nacional de Ubajara, Rio Cafundó, pouco acima da cachoeira; maximumElevationInMeters: 795; verbatimCoordinates: 3°50'13"S, 40°54'35"W; **Identification:** identifiedBy: Ângelo Parise Pinto; **Event:** samplingProtocol: Suspended intercept trap; verbatimEventDate: 10.ii.13; **Record Level:** institutionCode: DZRJ; basisOfRecord: PreservedSpecimen

##### Distribution

Colombia. Venezuela. Guyana. Suriname. Brazil: RR, MA, CE, MT, MG, MS, SP, RJ, RS. Ecuador. Bolivia. Paraguay. Argentina.

#### Macrothemis

Hagen, 1868

#### Macrothemis rupicola

Rácenis, 1957

##### Materials

- **Type status:** Other material. **Occurrence:** recordedBy: Santos, A.P.M. | Takiya, D.M.; individualCount: 1; sex: male; lifeStage: adult; **Location:** country: Brazil; stateProvince: Ceará; municipality: Ubajara; locality: Parque Nacional de Ubajara, Trilha Araticum, Rio das Minas; maximumElevationInMeters: 524; verbatimCoordinates: 3°50'3"S, 40°54'18"W; **Identification:** identifiedBy: Ângelo Parise Pinto; **Event:** samplingProtocol: Manual; verbatimEventDate: 18.ii.13; **Record Level:** institutionCode: DZRJ; basisOfRecord: PreservedSpecimen

##### Distribution

Venezuela. French Guiana. Brazil: CE!, MG, RJ.

##### Notes

New species record for Northeastern Brazil.

#### incertae sedis

#### Neocordulia

Selys, 1882

##### Notes

New genus record for CE.

#### Neocordulia setifera

(Hagen in Selys, 1871)

##### Materials

- **Type status:** Other material. **Occurrence:** recordedBy: Santos, A.P.M. | Takiya, D.M.; individualCount: 1; sex: male; lifeStage: adult; **Location:** country: Brazil; stateProvince: Ceará; municipality: Ubajara; locality: Parque Nacional de Ubajara, Trilha Araticum, Rio das Minas; maximumElevationInMeters: 524; verbatimCoordinates: 3°50'3"S, 40°54'18"W; **Identification:** identifiedBy: Ângelo Parise Pinto; **Event:** samplingProtocol: Manual; verbatimEventDate: 18.ii.13; **Record Level:** institutionCode: DZRJ; basisOfRecord: PreservedSpecimen

##### Distribution

Brazil: CE!, SP, RJ, PR. Argentina.

##### Notes

**Figure 23.**
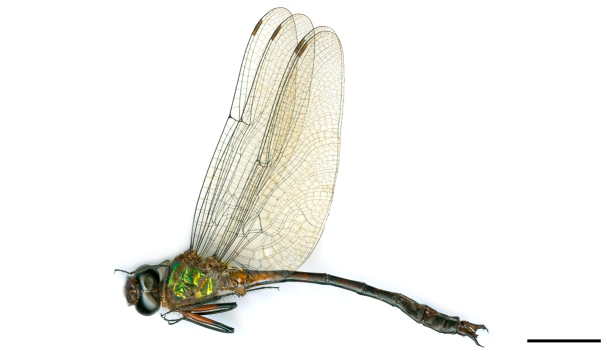
Lateral habitus of a male of *Neocordulia setifera* collected at PNU-02 (DZRJ), representing its first record for Northeastern Brazil. Scale bar = 10 mm.

New species record for Northeastern Brazil. See Fig. 23.

#### Zygoptera

#### Calopterygidae

#### Hetaerina

Hagen in Selys, 1853

#### Hetaerina indeprensa

Garrison, 1990

##### Materials

- **Type status:** Other material. **Occurrence:** recordedBy: Rafael, J.A. | Limeira-de-Oliveira, F. | Takiya, D.M. | Santos, A.P.M. | et al.; individualCount: 1; sex: male; lifeStage: adult; **Location:** country: Brazil; stateProvince: Ceará; municipality: Ubajara; locality: Parque Nacional de Ubajara, Trilha Araticum, Rio das Minas na altura da trilha do teleférico; maximumElevationInMeters: 420; verbatimCoordinates: 3°49'58"S, 40°53'53"W; **Identification:** identifiedBy: Ângelo Parise Pinto; **Event:** samplingProtocol: Malaise intercept trap; verbatimEventDate: 14.ii.13; **Record Level:** institutionCode: DZRJ; basisOfRecord: PreservedSpecimen
- **Type status:** Other material. **Occurrence:** recordedBy: Rafael, J.A. | Limeira-de-Oliveira, F. | Takiya, D.M. | Santos, A.P.M. | et al.; individualCount: 1; sex: male; lifeStage: adult; **Location:** country: Brazil; stateProvince: Ceará; municipality: Ubajara; locality: Parque Nacional de Ubajara, Rio das Minas, próximo ao Portão Araticum; maximumElevationInMeters: 328; verbatimCoordinates: 3°49'32.6"S, 40°53'32.8"W; **Identification:** identifiedBy: Ângelo Parise Pinto; **Event:** samplingProtocol: Malaise intercept trap; verbatimEventDate: 16.ii.13; **Record Level:** institutionCode: DZRJ; basisOfRecord: PreservedSpecimen

##### Distribution

Brazil: PA, CE!.

##### Notes

New species record for Northeastern Brazil.

#### Mnesarete

Cowley, 1934

#### Mnesarete sp. 1

##### Materials

- **Type status:** Other material. **Occurrence:** recordedBy: Santos, A.P.M. | Takiya, D.M.; individualCount: 1; sex: female; lifeStage: adult; **Location:** country: Brazil; stateProvince: Ceará; municipality: Ubajara; locality: Parque Nacional de Ubajara, Trilha Araticum, Rio das Minas; maximumElevationInMeters: 524; verbatimCoordinates: 3°50'3"S, 40°54'18"W; **Identification:** identifiedBy: Ângelo Parise Pinto; **Event:** samplingProtocol: Manual; verbatimEventDate: 18.ii.13; **Record Level:** institutionCode: DZRJ; basisOfRecord: PreservedSpecimen

#### Coenagrionidae

#### Acanthagrion

Selys, 1876

#### Acanthagrion quadratum

Selys, 1876

##### Materials

- **Type status:** Other material. **Occurrence:** recordedBy: Limeira-de-Oliveira, F. | Marques, D.W.A.; individualCount: 1; sex: female; lifeStage: adult; **Location:** country: Brazil; stateProvince: Ceará; municipality: Ubajara; locality: Parque Nacional de Ubajara, Cachoeira do Cafundó; maximumElevationInMeters: 783; verbatimCoordinates: 3°50'12"S, 40°54'35"W; **Identification:** identifiedBy: Ângelo Parise Pinto; **Event:** samplingProtocol: Malaise intercept trap; verbatimEventDate: 18.xi.12; **Record Level:** institutionCode: DZRJ; basisOfRecord: PreservedSpecimen

#### Argia

Rambur, 1842

#### Argia lilacina

Selys, 1865

##### Materials

- **Type status:** Other material. **Occurrence:** recordedBy: Limeira-de-Oliveira, F. | Marques, D.W.A.; individualCount: 1; sex: male; lifeStage: adult; **Location:** country: Brazil; stateProvince: Ceará; municipality: Ubajara; locality: Parque Nacional de Ubajara, Cachoeira do Cafundó; maximumElevationInMeters: 783; verbatimCoordinates: 3°50'12"S, 40°54'35"W; **Identification:** identifiedBy: Ângelo Parise Pinto; **Event:** samplingProtocol: Manual; verbatimEventDate: 13.xi.12; **Record Level:** institutionCode: DZRJ; basisOfRecord: PreservedSpecimen

##### Distribution

Brazil: CE!, TO, MT, GO, MG, MS, ES, SP, RJ. Bolivia. Paraguay. Argentina.

##### Notes

New species record for Northeastern Brazil.

#### Argia modesta

Selys, 1865

##### Materials

- **Type status:** Other material. **Occurrence:** recordedBy: Limeira-de-Oliveira, F. | Marques, D.W.A.; individualCount: 1; sex: male; lifeStage: adult; **Location:** country: Brazil; stateProvince: Ceará; municipality: Ubajara; locality: Parque Nacional de Ubajara, Cachoeira do Cafundó; maximumElevationInMeters: 783; verbatimCoordinates: 3°50'12"S, 40°54'35"W; **Identification:** identifiedBy: Rosser W. Garrison; **Event:** samplingProtocol: Manual; verbatimEventDate: 13.xi.12; **Record Level:** institutionCode: DZRJ; basisOfRecord: PreservedSpecimen
- **Type status:** Other material. **Occurrence:** recordedBy: Limeira-de-Oliveira, F. | Pinto Júnior, J.S.; individualCount: 1; sex: male; lifeStage: adult; **Location:** country: Brazil; stateProvince: Ceará; municipality: Ubajara; locality: Parque Nacional de Ubajara, Cachoeira do Cafundó; maximumElevationInMeters: 783; verbatimCoordinates: 3°50'12"S, 40°54'35"W; **Identification:** identifiedBy: Rosser W. Garrison; **Event:** samplingProtocol: Suspended intercept trap; verbatimEventDate: 16.xii.12; **Record Level:** institutionCode: DZRJ; basisOfRecord: PreservedSpecimen
- **Type status:** Other material. **Occurrence:** recordedBy: Limeira-de-Oliveira, F. | Pinto Júnior, J.S.; individualCount: 1; sex: female; lifeStage: adult; **Location:** country: Brazil; stateProvince: Ceará; municipality: Ubajara; locality: Parque Nacional de Ubajara, Cachoeira do Cafundó; maximumElevationInMeters: 783; verbatimCoordinates: 3°50'12"S, 40°54'35"W; **Identification:** identifiedBy: Rosser W. Garrison; **Event:** samplingProtocol: Suspended intercept trap; verbatimEventDate: 16.xii.12; **Record Level:** institutionCode: DZRJ; basisOfRecord: PreservedSpecimen
- **Type status:** Other material. **Occurrence:** recordedBy: Limeira-de-Oliveira, F. | Marques, D.W.A.; individualCount: 1; sex: male; lifeStage: adult; **Location:** country: Brazil; stateProvince: Ceará; municipality: Ubajara; locality: Parque Nacional de Ubajara, Cachoeira do Cafundó; maximumElevationInMeters: 783; verbatimCoordinates: 3°50'12"S, 40°54'35"W; **Identification:** identifiedBy: Rosser W. Garrison; **Event:** samplingProtocol: Malaise intercept trap; verbatimEventDate: 18.xi.12; **Record Level:** institutionCode: DZRJ; basisOfRecord: PreservedSpecimen

##### Distribution

Brazil: CE, MG, ES, SP, RJ.

#### Argia tinctipennis

Selys, 1865

##### Materials

- **Type status:** Other material. **Occurrence:** recordedBy: Santos, A.P.M. | Takiya, D.M.; individualCount: 1; sex: male; lifeStage: adult; **Location:** country: Brazil; stateProvince: Ceará; municipality: Ubajara; locality: Parque Nacional de Ubajara, Trilha Araticum, Rio das Minas; maximumElevationInMeters: 524; verbatimCoordinates: 3°50'3"S, 40°54'18"W; **Identification:** identifiedBy: Ângelo Parise Pinto; **Event:** samplingProtocol: Manual; verbatimEventDate: 18.ii.13; **Record Level:** institutionCode: DZRJ; basisOfRecord: PreservedSpecimen
- **Type status:** Other material. **Occurrence:** recordedBy: Limeira-de-Oliveira, F. | Pinto Júnior, J.S.; individualCount: 1; sex: female; lifeStage: adult; **Location:** country: Brazil; stateProvince: Ceará; municipality: Ubajara; locality: Parque Nacional de Ubajara, Cachoeira do Cafundó; maximumElevationInMeters: 783; verbatimCoordinates: 3°50'12"S, 40°54'35"W; **Identification:** identifiedBy: Rosser W. Garrison; **Event:** samplingProtocol: Suspended intercept trap; verbatimEventDate: 1.xii.12; **Record Level:** institutionCode: DZRJ; basisOfRecord: PreservedSpecimen

##### Distribution

Brazil: AM, PI!, CE!, MT, GO, MS. Peru.

##### Notes

New species record for Northeastern Brazil.

#### Argia sp. 1

##### Materials

- **Type status:** Other material. **Occurrence:** recordedBy: Limeira-de-Oliveira, F. | Pinto Júnior, J.S.; individualCount: 1; sex: male; lifeStage: adult; **Location:** country: Brazil; stateProvince: Ceará; municipality: Ubajara; locality: Parque Nacional de Ubajara, Cachoeira do Cafundó; maximumElevationInMeters: 783; verbatimCoordinates: 3°50'12"S, 40°54'35"W; **Identification:** identifiedBy: Rosser W. Garrison; **Event:** samplingProtocol: Suspended intercept trap; verbatimEventDate: 1.xii.12; **Record Level:** institutionCode: DZRJ; basisOfRecord: PreservedSpecimen
- **Type status:** Other material. **Occurrence:** recordedBy: Takiya, D.M. | Cavichioli, R.R.; individualCount: 1; sex: female; lifeStage: adult; **Location:** country: Brazil; stateProvince: Ceará; municipality: Ubajara; locality: Parque Nacional de Ubajara, Trilha Samambaia, Rio Gameleira; maximumElevationInMeters: 874; verbatimCoordinates: 3°50'25"S, 40°54'19"W; **Identification:** identifiedBy: Alcimar Carvalho; **Event:** samplingProtocol: Manual; verbatimEventDate: 24.iv.12; **Record Level:** institutionCode: DZRJ; basisOfRecord: PreservedSpecimen

#### Oxyagrion

Selys, 1876

#### Oxyagrion chapadense

Costa, 1978

##### Materials

- **Type status:** Other material. **Occurrence:** recordedBy: Santos, A.P.M. | Takiya, D.M.; individualCount: 1; sex: male; lifeStage: adult; **Location:** country: Brazil; stateProvince: Ceará; municipality: Ubajara; locality: Parque Nacional de Ubajara, Trilha Araticum, Rio das Minas; maximumElevationInMeters: 524; verbatimCoordinates: 3°50'3"S, 40°54'18"W; **Identification:** identifiedBy: Ângelo Parise Pinto; **Event:** samplingProtocol: Manual; verbatimEventDate: 18.ii.13; **Record Level:** institutionCode: DZRJ; basisOfRecord: PreservedSpecimen

##### Distribution

Brazil: CE, MT, BA, GO, MG, MS, SP, PR. Bolivia. Paraguay. Argentina. Uruguay.

##### Notes

See Fig. 24.

#### Plecoptera

#### Perlidae

#### Anacroneuria

Klapálek, 1909

#### Anacroneuria calori

Duarte & Lecci, 2016

##### Materials

- **Type status:** Other material. **Occurrence:** recordedBy: Rafael, J.A. | Limeira-de-Oliveira, F. | Takiya, D.M. | Santos, A.P.M | et al.; individualCount: 2; sex: male; lifeStage: adult; **Location:** country: Brazil; stateProvince: Ceará; municipality: Ubajara; locality: Parque Nacional de Ubajara, Trilha Araticum, Rio da Minas abaixo do teleférico; maximumElevationInMeters: 395; verbatimCoordinates: 3°49'43.3"S, 40°53'51.5"W; **Identification:** identifiedBy: Fernanda Avelino-Capistrano; **Event:** samplingProtocol: Malaise intercept trap; verbatimEventDate: 1.ii.13; **Record Level:** institutionCode: DZRJ; basisOfRecord: PreservedSpecimen
- **Type status:** Other material. **Occurrence:** recordedBy: Rafael, J.A. | Limeira-de-Oliveira, F. | Takiya, D.M. | Santos, A.P.M | et al.; individualCount: 2; sex: female; lifeStage: adult; **Location:** country: Brazil; stateProvince: Ceará; municipality: Ubajara; locality: Parque Nacional de Ubajara, Trilha Araticum, Rio da Minas abaixo do teleférico; maximumElevationInMeters: 395; verbatimCoordinates: 3°49'43.3"S, 40°53'51.5"W; **Identification:** identifiedBy: Fernanda Avelino-Capistrano; **Event:** samplingProtocol: Malaise intercept trap; verbatimEventDate: 1.ii.13; **Record Level:** institutionCode: DZRJ; basisOfRecord: PreservedSpecimen
- **Type status:** Other material. **Occurrence:** recordedBy: Santos, A.P.M. | Takiya, D.M.; individualCount: 2; sex: male; lifeStage: adult; **Location:** country: Brazil; stateProvince: Ceará; municipality: Ubajara; locality: Parque Nacional de Ubajara, Trilha Samambaia, Rio Gameleira; maximumElevationInMeters: 874; verbatimCoordinates: 3°50'25"S, 40°54'19"W; **Identification:** identifiedBy: Fernanda Avelino-Capistrano; **Event:** samplingProtocol: Pennsylvania light trap; verbatimEventDate: 14.ii.13; **Record Level:** institutionCode: DZRJ; basisOfRecord: PreservedSpecimen
- **Type status:** Other material. **Occurrence:** recordedBy: Santos, A.P.M. | Takiya, D.M.; individualCount: 3; sex: male; lifeStage: adult; **Location:** country: Brazil; stateProvince: Ceará; municipality: Ubajara; locality: Parque Nacional de Ubajara, Trilha Araticum, Rio das Minas; maximumElevationInMeters: 524; verbatimCoordinates: 3°50'3"S, 40°54'18"W; **Identification:** identifiedBy: Fernanda Avelino-Capistrano; **Event:** samplingProtocol: Pennsylvania light trap; verbatimEventDate: 14.ii.13; **Record Level:** institutionCode: DZRJ; basisOfRecord: PreservedSpecimen
- **Type status:** Other material. **Occurrence:** recordedBy: Rafael, J.A. | Limeira-de-Oliveira, F. | Takiya, D.M. | Santos, A.P.M | et al.; individualCount: 1; sex: male; lifeStage: adult; **Location:** country: Brazil; stateProvince: Ceará; municipality: Ubajara; locality: Parque Nacional de Ubajara, Trilha Araticum, Rio das Minas; maximumElevationInMeters: 524; verbatimCoordinates: 3°50'3"S, 40°54'18"W; **Identification:** identifiedBy: Fernanda Avelino-Capistrano; **Event:** samplingProtocol: Malaise intercept trap; verbatimEventDate: 16.ii.13; **Record Level:** institutionCode: DZRJ; basisOfRecord: PreservedSpecimen
- **Type status:** Other material. **Occurrence:** recordedBy: Santos, A.P.M. | Takiya, D.M.; individualCount: 4; lifeStage: immature; **Location:** country: Brazil; stateProvince: Ceará; municipality: Ubajara; locality: Parque Nacional de Ubajara, Cachoeira do Cafundó; maximumElevationInMeters: 783; verbatimCoordinates: 3°50'12"S, 40°54'35"W; **Identification:** identifiedBy: Fernanda Avelino-Capistrano; **Event:** samplingProtocol: Manual; verbatimEventDate: 16.ii.13; **Record Level:** institutionCode: DZRJ; basisOfRecord: PreservedSpecimen
- **Type status:** Other material. **Occurrence:** recordedBy: Santos, A.P.M. | Takiya, D.M.; individualCount: 1; sex: male; lifeStage: adult; **Location:** country: Brazil; stateProvince: Ceará; municipality: Ubajara; locality: Parque Nacional de Ubajara, Portão Neblina; maximumElevationInMeters: 849; verbatimCoordinates: 3°50'18"S, 40°53'54"W; **Identification:** identifiedBy: Fernanda Avelino-Capistrano; **Event:** samplingProtocol: Pennsylvania light trap; verbatimEventDate: 17.ii.13; **Record Level:** institutionCode: DZRJ; basisOfRecord: PreservedSpecimen
- **Type status:** Other material. **Occurrence:** recordedBy: Santos, A.P.M. | Takiya, D.M.; individualCount: 1; lifeStage: immature; **Location:** country: Brazil; stateProvince: Ceará; municipality: Ubajara; locality: Parque Nacional de Ubajara, Trilha Araticum, Rio das Minas; maximumElevationInMeters: 524; verbatimCoordinates: 3°50'3"S, 40°54'18"W; **Identification:** identifiedBy: Fernanda Avelino-Capistrano; **Event:** samplingProtocol: Manual; verbatimEventDate: 19.ii.13; **Record Level:** institutionCode: DZRJ; basisOfRecord: PreservedSpecimen
- **Type status:** Other material. **Occurrence:** recordedBy: Rafael, J.A. | Limeira-de-Oliveira, F. | Takiya, D.M. | et al.; individualCount: 7; sex: male; lifeStage: adult; **Location:** country: Brazil; stateProvince: Ceará; municipality: Ubajara; locality: Parque Nacional de Ubajara, Trilha Araticum, Rio das Minas na altura da trilha do teleférico; maximumElevationInMeters: 420; verbatimCoordinates: 3°49'58"S, 40°53'53"W; **Identification:** identifiedBy: Fernanda Avelino-Capistrano; **Event:** samplingProtocol: Malaise intercept trap; verbatimEventDate: 20.iv.12; **Record Level:** institutionCode: DZRJ; basisOfRecord: PreservedSpecimen
- **Type status:** Other material. **Occurrence:** recordedBy: Takiya, D.M. | Câmara, J.T.; individualCount: 1; sex: female; lifeStage: adult; **Location:** country: Brazil; stateProvince: Ceará; municipality: Ubajara; locality: Parque Nacional de Ubajara, Trilha Samambaia, Rio Gameleira; maximumElevationInMeters: 874; verbatimCoordinates: 3°50'25"S, 40°54'19"W; **Identification:** identifiedBy: Fernanda Avelino-Capistrano; **Event:** samplingProtocol: Pennsylvania light trap; verbatimEventDate: 21.iv.12; **Record Level:** institutionCode: DZRJ; basisOfRecord: PreservedSpecimen
- **Type status:** Other material. **Occurrence:** recordedBy: Takiya, D.M. | Somavilla, A.; individualCount: 1; sex: female; lifeStage: adult; **Location:** country: Brazil; stateProvince: Ceará; municipality: Ubajara; locality: Parque Nacional de Ubajara, Trilha Araticum, Rio das Minas; maximumElevationInMeters: 524; verbatimCoordinates: 3°50'3"S, 40°54'18"W; **Identification:** identifiedBy: Fernanda Avelino-Capistrano; **Event:** samplingProtocol: Pennsylvania light trap; verbatimEventDate: 22.iv.12; **Record Level:** institutionCode: DZRJ; basisOfRecord: PreservedSpecimen
- **Type status:** Other material. **Occurrence:** recordedBy: Takiya, D.M.; individualCount: 1; lifeStage: immature; **Location:** country: Brazil; stateProvince: Ceará; municipality: Ubajara; locality: Parque Nacional de Ubajara, Trilha Araticum, Rio Cafundó; maximumElevationInMeters: 753; verbatimCoordinates: 3°50'12"S, 40°54'31"W; **Identification:** identifiedBy: Fernanda Avelino-Capistrano; **Event:** samplingProtocol: Manual; verbatimEventDate: 23.iv.12; **Record Level:** institutionCode: DZRJ; basisOfRecord: PreservedSpecimen
- **Type status:** Other material. **Occurrence:** recordedBy: Rafael, J.A. | Limeira-de-Oliveira, F. | Takiya, D.M. | et al.; individualCount: 7; sex: female; lifeStage: adult; **Location:** country: Brazil; stateProvince: Ceará; municipality: Ubajara; locality: Parque Nacional de Ubajara, Trilha Araticum, Rio das Minas na altura da trilha do teleférico; maximumElevationInMeters: 420; verbatimCoordinates: 3°49'58"S, 40°53'53"W; **Identification:** identifiedBy: Fernanda Avelino-Capistrano; **Event:** samplingProtocol: Malaise intercept trap; verbatimEventDate: 23.iv.12; **Record Level:** institutionCode: DZRJ; basisOfRecord: PreservedSpecimen
- **Type status:** Other material. **Occurrence:** recordedBy: Takiya, D.M. | Rafael, J.A.; individualCount: 1; sex: male; lifeStage: adult; **Location:** country: Brazil; stateProvince: Ceará; municipality: Ubajara; locality: Parque Nacional de Ubajara, Cachoeira do Cafundó; maximumElevationInMeters: 783; verbatimCoordinates: 3°50'12"S, 40°54'35"W; **Identification:** identifiedBy: Fernanda Avelino-Capistrano; **Event:** samplingProtocol: Pennsylvania light trap; verbatimEventDate: 24.iv.12; **Record Level:** institutionCode: DZRJ; basisOfRecord: PreservedSpecimen

##### Distribution

Brazil: PI!, CE.

##### Notes

See Fig. 25.

#### Trichoptera

#### Calamoceratidae

#### Phylloicus

Müller, 1880

#### Phylloicus obliquus

Navás, 1931

##### Materials

- **Type status:** Other material. **Occurrence:** recordedBy: Limeira-de-Oliveira | et al.; individualCount: 3; sex: male; lifeStage: adult; **Location:** country: Brazil; stateProvince: Ceará; municipality: Ubajara; locality: Parque Nacional de Ubajara, Rio Cafundó, pouco acima da cachoeira; maximumElevationInMeters: 795; verbatimCoordinates: 3°50'13"S, 40°54'35"W; **Identification:** identifiedBy: Allan Paulo Moreira dos Santos; **Event:** samplingProtocol: Malaise intercept trap; verbatimEventDate: 1.xii.12; **Record Level:** institutionCode: DZRJ; basisOfRecord: PreservedSpecimen
- **Type status:** Other material. **Occurrence:** recordedBy: Limeira-de-Oliveira | et al.; individualCount: 1; sex: male; lifeStage: adult; **Location:** country: Brazil; stateProvince: Ceará; municipality: Ubajara; locality: Parque Nacional de Ubajara, Rio Cafundó, pouco acima da cachoeira; maximumElevationInMeters: 795; verbatimCoordinates: 3°50'13"S, 40°54'35"W; **Identification:** identifiedBy: Allan Paulo Moreira dos Santos; **Event:** samplingProtocol: Malaise intercept trap; verbatimEventDate: 13.xi.12; **Record Level:** institutionCode: DZRJ; basisOfRecord: PreservedSpecimen
- **Type status:** Other material. **Occurrence:** recordedBy: Limeira-de-Oliveira | et al.; individualCount: 1; sex: female; lifeStage: adult; **Location:** country: Brazil; stateProvince: Ceará; municipality: Ubajara; locality: Parque Nacional de Ubajara, Rio Cafundó, pouco acima da cachoeira; maximumElevationInMeters: 795; verbatimCoordinates: 3°50'13"S, 40°54'35"W; **Identification:** identifiedBy: Allan Paulo Moreira dos Santos; **Event:** samplingProtocol: Malaise intercept trap; verbatimEventDate: 13.xi.12; **Record Level:** institutionCode: DZRJ; basisOfRecord: PreservedSpecimen
- **Type status:** Other material. **Occurrence:** recordedBy: Limeira-de-Oliveira | et al.; individualCount: 2; sex: male; lifeStage: adult; **Location:** country: Brazil; stateProvince: Ceará; municipality: Ubajara; locality: Parque Nacional de Ubajara, Rio Cafundó, pouco acima da cachoeira; maximumElevationInMeters: 795; verbatimCoordinates: 3°50'13"S, 40°54'35"W; **Identification:** identifiedBy: Allan Paulo Moreira dos Santos; **Event:** samplingProtocol: Malaise intercept trap; verbatimEventDate: 13.xi.12; **Record Level:** institutionCode: DZRJ; basisOfRecord: PreservedSpecimen
- **Type status:** Other material. **Occurrence:** recordedBy: Limeira-de-Oliveira | et al.; individualCount: 1; sex: female; lifeStage: adult; **Location:** country: Brazil; stateProvince: Ceará; municipality: Ubajara; locality: Parque Nacional de Ubajara, Rio Cafundó, pouco acima da cachoeira; maximumElevationInMeters: 795; verbatimCoordinates: 3°50'13"S, 40°54'35"W; **Identification:** identifiedBy: Allan Paulo Moreira dos Santos; **Event:** samplingProtocol: Malaise intercept trap; verbatimEventDate: 13.xi.12; **Record Level:** institutionCode: DZRJ; basisOfRecord: PreservedSpecimen
- **Type status:** Other material. **Occurrence:** recordedBy: Rafael, J.A. | Limeira-de-Oliveira, F. | Takiya, D.M. | Santos, A.P.M. | et al.; individualCount: 1; sex: male; lifeStage: adult; **Location:** country: Brazil; stateProvince: Ceará; municipality: Ubajara; locality: Parque Nacional de Ubajara, Trilha Araticum, Rio das Minas na altura da trilha do teleférico; maximumElevationInMeters: 420; verbatimCoordinates: 3°49'58"S, 40°53'53"W; **Identification:** identifiedBy: Allan Paulo Moreira dos Santos; **Event:** samplingProtocol: Malaise intercept trap; verbatimEventDate: 14.ii.13; **Record Level:** institutionCode: DZRJ; basisOfRecord: PreservedSpecimen
- **Type status:** Other material. **Occurrence:** recordedBy: Rafael, J.A. | Limeira-de-Oliveira, F. | Takiya, D.M. | Santos, A.P.M. | et al.; individualCount: 1; sex: female; lifeStage: adult; **Location:** country: Brazil; stateProvince: Ceará; municipality: Ubajara; locality: Parque Nacional de Ubajara, Trilha Araticum, Rio das Minas na altura da trilha do teleférico; maximumElevationInMeters: 420; verbatimCoordinates: 3°49'58"S, 40°53'53"W; **Identification:** identifiedBy: Allan Paulo Moreira dos Santos; **Event:** samplingProtocol: Malaise intercept trap; verbatimEventDate: 14.ii.13; **Record Level:** institutionCode: DZRJ; basisOfRecord: PreservedSpecimen
- **Type status:** Other material. **Occurrence:** recordedBy: Rafael, J.A. | Limeira-de-Oliveira, F. | Takiya, D.M. | et al.; individualCount: 13; sex: male; lifeStage: adult; **Location:** country: Brazil; stateProvince: Ceará; municipality: Ubajara; locality: Parque Nacional de Ubajara, Trilha Samambaia, Rio Gameleira; maximumElevationInMeters: 874; verbatimCoordinates: 3°50'25"S, 40°54'19"W; **Identification:** identifiedBy: Allan Paulo Moreira dos Santos; **Event:** samplingProtocol: Malaise intercept trap; verbatimEventDate: 20.iv.12; **Record Level:** institutionCode: DZRJ; basisOfRecord: PreservedSpecimen
- **Type status:** Other material. **Occurrence:** recordedBy: Rafael, J.A. | Limeira-de-Oliveira, F. | Takiya, D.M. | et al.; individualCount: 1; sex: female; lifeStage: adult; **Location:** country: Brazil; stateProvince: Ceará; municipality: Ubajara; locality: Parque Nacional de Ubajara, Trilha Samambaia, Rio Gameleira; maximumElevationInMeters: 874; verbatimCoordinates: 3°50'25"S, 40°54'19"W; **Identification:** identifiedBy: Allan Paulo Moreira dos Santos; **Event:** samplingProtocol: Malaise intercept trap; verbatimEventDate: 20.iv.12; **Record Level:** institutionCode: DZRJ; basisOfRecord: PreservedSpecimen
- **Type status:** Other material. **Occurrence:** recordedBy: Rafael, J.A. | Limeira-de-Oliveira, F. | Takiya, D.M. | et al.; individualCount: 6; sex: male; lifeStage: adult; **Location:** country: Brazil; stateProvince: Ceará; municipality: Ubajara; locality: Parque Nacional de Ubajara, Rio Cafundó, pouco acima da cachoeira; maximumElevationInMeters: 795; verbatimCoordinates: 3°50'13"S, 40°54'35"W; **Identification:** identifiedBy: Allan Paulo Moreira dos Santos; **Event:** samplingProtocol: Malaise intercept trap; verbatimEventDate: 21.iv.12; **Record Level:** institutionCode: DZRJ; basisOfRecord: PreservedSpecimen
- **Type status:** Other material. **Occurrence:** recordedBy: Takiya, D.M. | Rafael, J.A.; individualCount: 1; sex: male; lifeStage: adult; **Location:** country: Brazil; stateProvince: Ceará; municipality: Ubajara; locality: Parque Nacional de Ubajara, Rio Cafundó, pouco acima da cachoeira; maximumElevationInMeters: 795; verbatimCoordinates: 3°50'13"S, 40°54'35"W; **Identification:** identifiedBy: Allan Paulo Moreira dos Santos; **Event:** samplingProtocol: Pennsylvania light trap; verbatimEventDate: 24.iv.12; **Record Level:** institutionCode: DZRJ; basisOfRecord: PreservedSpecimen

##### Distribution

Brazil: CE, BA, MG, RJ, SC.

#### Phylloicus pirapo

Prather, 2003

##### Materials

- **Type status:** Other material. **Occurrence:** recordedBy: Limeira-de-Oliveira | Rafael, J.A. | et al.; individualCount: 1; sex: male; lifeStage: adult; **Location:** country: Brazil; stateProvince: Ceará; municipality: Ubajara; locality: Parque Nacional de Ubajara, Rio Cafundó, pouco acima da cachoeira; maximumElevationInMeters: 795; verbatimCoordinates: 3°50'13"S, 40°54'35"W; **Identification:** identifiedBy: Allan Paulo Moreira dos Santos; **Event:** samplingProtocol: Suspended intercept trap; verbatimEventDate: 1.ii.13; **Record Level:** institutionCode: DZRJ; basisOfRecord: PreservedSpecimen
- **Type status:** Other material. **Occurrence:** recordedBy: Limeira-de-Oliveira | Rafael, J.A. | et al.; individualCount: 2; sex: female; lifeStage: adult; **Location:** country: Brazil; stateProvince: Ceará; municipality: Ubajara; locality: Parque Nacional de Ubajara, Rio Cafundó, pouco acima da cachoeira; maximumElevationInMeters: 795; verbatimCoordinates: 3°50'13"S, 40°54'35"W; **Identification:** identifiedBy: Allan Paulo Moreira dos Santos; **Event:** samplingProtocol: Suspended intercept trap; verbatimEventDate: 1.ii.13; **Record Level:** institutionCode: DZRJ; basisOfRecord: PreservedSpecimen
- **Type status:** Other material. **Occurrence:** recordedBy: Limeira-de-Oliveira | et al.; individualCount: 2; sex: male; lifeStage: adult; **Location:** country: Brazil; stateProvince: Ceará; municipality: Ubajara; locality: Parque Nacional de Ubajara, Rio Cafundó, pouco acima da cachoeira; maximumElevationInMeters: 795; verbatimCoordinates: 3°50'13"S, 40°54'35"W; **Identification:** identifiedBy: Allan Paulo Moreira dos Santos; **Event:** samplingProtocol: Malaise intercept trap; verbatimEventDate: 1.ii.13; **Record Level:** institutionCode: DZRJ; basisOfRecord: PreservedSpecimen
- **Type status:** Other material. **Occurrence:** recordedBy: Limeira-de-Oliveira | et al.; individualCount: 3; sex: female; lifeStage: adult; **Location:** country: Brazil; stateProvince: Ceará; municipality: Ubajara; locality: Parque Nacional de Ubajara, Rio Cafundó, pouco acima da cachoeira; maximumElevationInMeters: 795; verbatimCoordinates: 3°50'13"S, 40°54'35"W; **Identification:** identifiedBy: Allan Paulo Moreira dos Santos; **Event:** samplingProtocol: Malaise intercept trap; verbatimEventDate: 1.ii.13; **Record Level:** institutionCode: DZRJ; basisOfRecord: PreservedSpecimen
- **Type status:** Other material. **Occurrence:** recordedBy: Limeira-de-Oliveira | et al.; individualCount: 1; sex: female; lifeStage: adult; **Location:** country: Brazil; stateProvince: Ceará; municipality: Ubajara; locality: Parque Nacional de Ubajara, Rio Cafundó, pouco acima da cachoeira; maximumElevationInMeters: 795; verbatimCoordinates: 3°50'13"S, 40°54'35"W; **Identification:** identifiedBy: Allan Paulo Moreira dos Santos; **Event:** samplingProtocol: Malaise intercept trap; verbatimEventDate: 1.xii.12; **Record Level:** institutionCode: DZRJ; basisOfRecord: PreservedSpecimen
- **Type status:** Other material. **Occurrence:** recordedBy: Rafael, J.A. | Limeira-de-Oliveira, F. | Takiya, D.M. | Santos, A.P.M. | et al.; individualCount: 1; sex: female; lifeStage: adult; **Location:** country: Brazil; stateProvince: Ceará; municipality: Ubajara; locality: Parque Nacional de Ubajara, Trilha Samambaia, Rio Gameleira; maximumElevationInMeters: 874; verbatimCoordinates: 3°50'25"S, 40°54'19"W; **Identification:** identifiedBy: Allan Paulo Moreira dos Santos; **Event:** samplingProtocol: Malaise intercept trap; verbatimEventDate: 14.ii.13; **Record Level:** institutionCode: DZRJ; basisOfRecord: PreservedSpecimen
- **Type status:** Other material. **Occurrence:** recordedBy: Rafael, J.A. | Limeira-de-Oliveira, F. | Takiya, D.M. | Santos, A.P.M. | et al.; individualCount: 1; sex: male; lifeStage: adult; **Location:** country: Brazil; stateProvince: Ceará; municipality: Ubajara; locality: Parque Nacional de Ubajara, Trilha Araticum, Rio das Minas na altura da trilha do teleférico; maximumElevationInMeters: 420; verbatimCoordinates: 3°49'58"S, 40°53'53"W; **Identification:** identifiedBy: Allan Paulo Moreira dos Santos; **Event:** samplingProtocol: Malaise intercept trap; verbatimEventDate: 14.ii.13; **Record Level:** institutionCode: DZRJ; basisOfRecord: PreservedSpecimen
- **Type status:** Other material. **Occurrence:** recordedBy: Rafael, J.A. | Limeira-de-Oliveira, F. | Takiya, D.M. | Santos, A.P.M. | et al.; individualCount: 1; sex: female; lifeStage: adult; **Location:** country: Brazil; stateProvince: Ceará; municipality: Ubajara; locality: Parque Nacional de Ubajara, Trilha Araticum, Rio das Minas na altura da trilha do teleférico; maximumElevationInMeters: 420; verbatimCoordinates: 3°49'58"S, 40°53'53"W; **Identification:** identifiedBy: Allan Paulo Moreira dos Santos; **Event:** samplingProtocol: Malaise intercept trap; verbatimEventDate: 14.ii.13; **Record Level:** institutionCode: DZRJ; basisOfRecord: PreservedSpecimen
- **Type status:** Other material. **Occurrence:** recordedBy: Rafael, J.A. | Limeira-de-Oliveira, F. | Takiya, D.M. | Santos, A.P.M. | et al.; individualCount: 12; sex: male; lifeStage: adult; **Location:** country: Brazil; stateProvince: Ceará; municipality: Ubajara; locality: Parque Nacional de Ubajara, Trilha Araticum, Rio das Minas na altura da trilha do teleférico; maximumElevationInMeters: 420; verbatimCoordinates: 3°49'58"S, 40°53'53"W; **Identification:** identifiedBy: Allan Paulo Moreira dos Santos; **Event:** samplingProtocol: Malaise intercept trap; verbatimEventDate: 14.ii.13; **Record Level:** institutionCode: DZRJ; basisOfRecord: PreservedSpecimen
- **Type status:** Other material. **Occurrence:** recordedBy: Rafael, J.A. | Limeira-de-Oliveira, F. | Takiya, D.M. | Santos, A.P.M. | et al.; individualCount: 1; sex: female; lifeStage: adult; **Location:** country: Brazil; stateProvince: Ceará; municipality: Ubajara; locality: Parque Nacional de Ubajara, Trilha Araticum, Rio das Minas na altura da trilha do teleférico; maximumElevationInMeters: 420; verbatimCoordinates: 3°49'58"S, 40°53'53"W; **Identification:** identifiedBy: Allan Paulo Moreira dos Santos; **Event:** samplingProtocol: Malaise intercept trap; verbatimEventDate: 14.ii.13; **Record Level:** institutionCode: DZRJ; basisOfRecord: PreservedSpecimen
- **Type status:** Other material. **Occurrence:** recordedBy: Rafael, J.A. | Limeira-de-Oliveira, F. | Takiya, D.M. | Santos, A.P.M. | et al.; individualCount: 15; sex: male; lifeStage: adult; **Location:** country: Brazil; stateProvince: Ceará; municipality: Ubajara; locality: Parque Nacional de Ubajara, Trilha Araticum, Rio das Minas na altura da trilha do teleférico; maximumElevationInMeters: 420; verbatimCoordinates: 3°49'58"S, 40°53'53"W; **Identification:** identifiedBy: Allan Paulo Moreira dos Santos; **Event:** samplingProtocol: Malaise intercept trap; verbatimEventDate: 14.ii.13; **Record Level:** institutionCode: DZRJ; basisOfRecord: PreservedSpecimen
- **Type status:** Other material. **Occurrence:** recordedBy: Rafael, J.A. | Limeira-de-Oliveira, F. | Takiya, D.M. | Santos, A.P.M. | et al.; individualCount: 12; sex: female; lifeStage: adult; **Location:** country: Brazil; stateProvince: Ceará; municipality: Ubajara; locality: Parque Nacional de Ubajara, Trilha Araticum, Rio das Minas na altura da trilha do teleférico; maximumElevationInMeters: 420; verbatimCoordinates: 3°49'58"S, 40°53'53"W; **Identification:** identifiedBy: Allan Paulo Moreira dos Santos; **Event:** samplingProtocol: Malaise intercept trap; verbatimEventDate: 14.ii.13; **Record Level:** institutionCode: DZRJ; basisOfRecord: PreservedSpecimen
- **Type status:** Other material. **Occurrence:** recordedBy: Rafael, J.A. | Limeira-de-Oliveira, F. | Takiya, D.M. | Santos, A.P.M. | et al.; individualCount: 1; sex: male; lifeStage: adult; **Location:** country: Brazil; stateProvince: Ceará; municipality: Ubajara; locality: Parque Nacional de Ubajara, Rio das Minas, próximo ao Portão Araticum; maximumElevationInMeters: 328; verbatimCoordinates: 3°49'32.6"S, 40°53'32.8"W; **Identification:** identifiedBy: Allan Paulo Moreira dos Santos; **Event:** samplingProtocol: Malaise intercept trap; verbatimEventDate: 14.ii.13; **Record Level:** institutionCode: DZRJ; basisOfRecord: PreservedSpecimen
- **Type status:** Other material. **Occurrence:** recordedBy: Rafael, J.A. | Limeira-de-Oliveira, F. | Takiya, D.M. | Santos, A.P.M. | et al.; individualCount: 2; sex: female; lifeStage: adult; **Location:** country: Brazil; stateProvince: Ceará; municipality: Ubajara; locality: Parque Nacional de Ubajara, Rio das Minas, próximo ao Portão Araticum; maximumElevationInMeters: 328; verbatimCoordinates: 3°49'32.6"S, 40°53'32.8"W; **Identification:** identifiedBy: Allan Paulo Moreira dos Santos; **Event:** samplingProtocol: Malaise intercept trap; verbatimEventDate: 14.ii.13; **Record Level:** institutionCode: DZRJ; basisOfRecord: PreservedSpecimen
- **Type status:** Other material. **Occurrence:** recordedBy: Rafael, J.A. | Limeira-de-Oliveira, F. | Takiya, D.M. | Santos, A.P.M. | et al.; individualCount: 5; sex: male; lifeStage: adult; **Location:** country: Brazil; stateProvince: Ceará; municipality: Ubajara; locality: Parque Nacional de Ubajara, Rio das Minas, próximo ao Portão Araticum; maximumElevationInMeters: 328; verbatimCoordinates: 3°49'32.6"S, 40°53'32.8"W; **Identification:** identifiedBy: Allan Paulo Moreira dos Santos; **Event:** samplingProtocol: Malaise intercept trap; verbatimEventDate: 14.ii.13; **Record Level:** institutionCode: DZRJ; basisOfRecord: PreservedSpecimen
- **Type status:** Other material. **Occurrence:** recordedBy: Rafael, J.A. | Limeira-de-Oliveira, F. | Takiya, D.M. | Santos, A.P.M. | et al.; individualCount: 6; sex: female; lifeStage: adult; **Location:** country: Brazil; stateProvince: Ceará; municipality: Ubajara; locality: Parque Nacional de Ubajara, Rio das Minas, próximo ao Portão Araticum; maximumElevationInMeters: 328; verbatimCoordinates: 3°49'32.6"S, 40°53'32.8"W; **Identification:** identifiedBy: Allan Paulo Moreira dos Santos; **Event:** samplingProtocol: Malaise intercept trap; verbatimEventDate: 14.ii.13; **Record Level:** institutionCode: DZRJ; basisOfRecord: PreservedSpecimen
- **Type status:** Other material. **Occurrence:** recordedBy: Rafael, J.A. | Limeira-de-Oliveira, F. | Takiya, D.M. | Santos, A.P.M. | et al.; individualCount: 6; sex: male; lifeStage: adult; **Location:** country: Brazil; stateProvince: Ceará; municipality: Ubajara; locality: Parque Nacional de Ubajara, Rio das Minas, próximo ao Portão Araticum; maximumElevationInMeters: 328; verbatimCoordinates: 3°49'32.6"S, 40°53'32.8"W; **Identification:** identifiedBy: Allan Paulo Moreira dos Santos; **Event:** samplingProtocol: Malaise intercept trap; verbatimEventDate: 14.ii.13; **Record Level:** institutionCode: DZRJ; basisOfRecord: PreservedSpecimen
- **Type status:** Other material. **Occurrence:** recordedBy: Rafael, J.A. | Limeira-de-Oliveira, F. | Takiya, D.M. | Santos, A.P.M. | et al.; individualCount: 14; sex: female; lifeStage: adult; **Location:** country: Brazil; stateProvince: Ceará; municipality: Ubajara; locality: Parque Nacional de Ubajara, Rio das Minas, próximo ao Portão Araticum; maximumElevationInMeters: 328; verbatimCoordinates: 3°49'32.6"S, 40°53'32.8"W; **Identification:** identifiedBy: Allan Paulo Moreira dos Santos; **Event:** samplingProtocol: Malaise intercept trap; verbatimEventDate: 14.ii.13; **Record Level:** institutionCode: DZRJ; basisOfRecord: PreservedSpecimen
- **Type status:** Other material. **Occurrence:** recordedBy: Rafael, J.A. | Limeira-de-Oliveira, F. | Takiya, D.M. | Santos, A.P.M. | et al.; individualCount: 2; sex: male; lifeStage: adult; **Location:** country: Brazil; stateProvince: Ceará; municipality: Ubajara; locality: Parque Nacional de Ubajara, Trilha Samambaia, Rio Gameleira; maximumElevationInMeters: 874; verbatimCoordinates: 3°50'25"S, 40°54'19"W; **Identification:** identifiedBy: Allan Paulo Moreira dos Santos; **Event:** samplingProtocol: Malaise intercept trap; verbatimEventDate: 17.ii.13; **Record Level:** institutionCode: DZRJ; basisOfRecord: PreservedSpecimen
- **Type status:** Other material. **Occurrence:** recordedBy: Rafael, J.A. | Limeira-de-Oliveira, F. | Takiya, D.M. | Santos, A.P.M. | et al.; individualCount: 2; sex: female; lifeStage: adult; **Location:** country: Brazil; stateProvince: Ceará; municipality: Ubajara; locality: Parque Nacional de Ubajara, Trilha Samambaia, Rio Gameleira; maximumElevationInMeters: 874; verbatimCoordinates: 3°50'25"S, 40°54'19"W; **Identification:** identifiedBy: Allan Paulo Moreira dos Santos; **Event:** samplingProtocol: Malaise intercept trap; verbatimEventDate: 17.ii.13; **Record Level:** institutionCode: DZRJ; basisOfRecord: PreservedSpecimen
- **Type status:** Other material. **Occurrence:** recordedBy: Rafael, J.A. | Limeira-de-Oliveira, F. | Takiya, D.M. | Santos, A.P.M. | et al.; individualCount: 1; sex: male; lifeStage: adult; **Location:** country: Brazil; stateProvince: Ceará; municipality: Ubajara; locality: Parque Nacional de Ubajara, Trilha Araticum, Rio das Minas; maximumElevationInMeters: 524; verbatimCoordinates: 3°50'3"S, 40°54'18"W; **Identification:** identifiedBy: Allan Paulo Moreira dos Santos; **Event:** samplingProtocol: Malaise intercept trap; verbatimEventDate: 17.ii.13; **Record Level:** institutionCode: DZRJ; basisOfRecord: PreservedSpecimen
- **Type status:** Other material. **Occurrence:** recordedBy: Rafael, J.A. | Limeira-de-Oliveira, F. | Takiya, D.M. | Santos, A.P.M. | et al.; individualCount: 1; sex: female; lifeStage: adult; **Location:** country: Brazil; stateProvince: Ceará; municipality: Ubajara; locality: Parque Nacional de Ubajara, Trilha Araticum, Rio das Minas; maximumElevationInMeters: 524; verbatimCoordinates: 3°50'3"S, 40°54'18"W; **Identification:** identifiedBy: Allan Paulo Moreira dos Santos; **Event:** samplingProtocol: Malaise intercept trap; verbatimEventDate: 17.ii.13; **Record Level:** institutionCode: DZRJ; basisOfRecord: PreservedSpecimen
- **Type status:** Other material. **Occurrence:** recordedBy: Rafael, J.A. | Limeira-de-Oliveira, F. | Takiya, D.M. | Santos, A.P.M. | et al.; individualCount: 3; sex: male; lifeStage: adult; **Location:** country: Brazil; stateProvince: Ceará; municipality: Ubajara; locality: Parque Nacional de Ubajara, Rio das Minas, próximo ao Portão Araticum; maximumElevationInMeters: 328; verbatimCoordinates: 3°49'32.6"S, 40°53'32.8"W; **Identification:** identifiedBy: Allan Paulo Moreira dos Santos; **Event:** samplingProtocol: Malaise intercept trap; verbatimEventDate: 17.ii.13; **Record Level:** institutionCode: DZRJ; basisOfRecord: PreservedSpecimen
- **Type status:** Other material. **Occurrence:** recordedBy: Rafael, J.A. | Limeira-de-Oliveira, F. | et al.; individualCount: 1; sex: male; lifeStage: adult; **Location:** country: Brazil; stateProvince: Ceará; municipality: Ubajara; locality: Parque Nacional de Ubajara, Rio Cafundó, pouco acima da cachoeira; maximumElevationInMeters: 795; verbatimCoordinates: 3°50'13"S, 40°54'35"W; **Identification:** identifiedBy: Allan Paulo Moreira dos Santos; **Event:** samplingProtocol: Suspended intercept trap; verbatimEventDate: 19.i.13; **Record Level:** institutionCode: DZRJ; basisOfRecord: PreservedSpecimen
- **Type status:** Other material. **Occurrence:** recordedBy: Rafael, J.A. | Limeira-de-Oliveira, F. | et al.; individualCount: 1; sex: male; lifeStage: adult; **Location:** country: Brazil; stateProvince: Ceará; municipality: Ubajara; locality: Parque Nacional de Ubajara, Rio Cafundó, pouco acima da cachoeira; maximumElevationInMeters: 795; verbatimCoordinates: 3°50'13"S, 40°54'35"W; **Identification:** identifiedBy: Allan Paulo Moreira dos Santos; **Event:** samplingProtocol: Suspended intercept trap; verbatimEventDate: 19.i.13; **Record Level:** institutionCode: DZRJ; basisOfRecord: PreservedSpecimen
- **Type status:** Other material. **Occurrence:** recordedBy: Rafael, J.A. | Limeira-de-Oliveira, F. | Takiya, D.M. | et al.; individualCount: 2; sex: female; lifeStage: adult; **Location:** country: Brazil; stateProvince: Ceará; municipality: Ubajara; locality: Parque Nacional de Ubajara, Trilha Samambaia, Rio Gameleira; maximumElevationInMeters: 874; verbatimCoordinates: 3°50'25"S, 40°54'19"W; **Identification:** identifiedBy: Allan Paulo Moreira dos Santos; **Event:** samplingProtocol: Malaise intercept trap; verbatimEventDate: 20.iv.12; **Record Level:** institutionCode: DZRJ; basisOfRecord: PreservedSpecimen
- **Type status:** Other material. **Occurrence:** recordedBy: Rafael, J.A. | Limeira-de-Oliveira, F. | Takiya, D.M. | et al.; individualCount: 5; sex: male; lifeStage: adult; **Location:** country: Brazil; stateProvince: Ceará; municipality: Ubajara; locality: Parque Nacional de Ubajara, Trilha Araticum, Rio das Minas na altura da trilha do teleférico; maximumElevationInMeters: 420; verbatimCoordinates: 3°49'58"S, 40°53'53"W; **Identification:** identifiedBy: Allan Paulo Moreira dos Santos; **Event:** samplingProtocol: Malaise intercept trap; verbatimEventDate: 20.iv.12; **Record Level:** institutionCode: DZRJ; basisOfRecord: PreservedSpecimen
- **Type status:** Other material. **Occurrence:** recordedBy: Rafael, J.A. | Limeira-de-Oliveira, F. | Takiya, D.M. | et al.; individualCount: 14; sex: female; lifeStage: adult; **Location:** country: Brazil; stateProvince: Ceará; municipality: Ubajara; locality: Parque Nacional de Ubajara, Trilha Araticum, Rio das Minas na altura da trilha do teleférico; maximumElevationInMeters: 420; verbatimCoordinates: 3°49'58"S, 40°53'53"W; **Identification:** identifiedBy: Allan Paulo Moreira dos Santos; **Event:** samplingProtocol: Malaise intercept trap; verbatimEventDate: 20.iv.12; **Record Level:** institutionCode: DZRJ; basisOfRecord: PreservedSpecimen
- **Type status:** Other material. **Occurrence:** recordedBy: Rafael, J.A. | Limeira-de-Oliveira, F. | Takiya, D.M. | et al.; individualCount: 1; sex: male; lifeStage: adult; **Location:** country: Brazil; stateProvince: Ceará; municipality: Ubajara; locality: Parque Nacional de Ubajara, Rio Cafundó, pouco acima da cachoeira; maximumElevationInMeters: 795; verbatimCoordinates: 3°50'13"S, 40°54'35"W; **Identification:** identifiedBy: Allan Paulo Moreira dos Santos; **Event:** samplingProtocol: Malaise intercept trap; verbatimEventDate: 21.iv.12; **Record Level:** institutionCode: DZRJ; basisOfRecord: PreservedSpecimen
- **Type status:** Other material. **Occurrence:** recordedBy: Limeira-de-Oliveira | et al.; individualCount: 1; sex: male; lifeStage: adult; **Location:** country: Brazil; stateProvince: Ceará; municipality: Ubajara; locality: Parque Nacional de Ubajara, Trilha Araticum, Rio das Minas na altura da trilha do teleférico; maximumElevationInMeters: 420; verbatimCoordinates: 3°49'58"S, 40°53'53"W; **Identification:** identifiedBy: Allan Paulo Moreira dos Santos; **Event:** samplingProtocol: Malaise intercept trap; verbatimEventDate: 21.v.12; **Record Level:** institutionCode: DZRJ; basisOfRecord: PreservedSpecimen
- **Type status:** Other material. **Occurrence:** recordedBy: Limeira-de-Oliveira | et al.; individualCount: 3; sex: female; lifeStage: adult; **Location:** country: Brazil; stateProvince: Ceará; municipality: Ubajara; locality: Parque Nacional de Ubajara, Trilha Araticum, Rio das Minas na altura da trilha do teleférico; maximumElevationInMeters: 420; verbatimCoordinates: 3°49'58"S, 40°53'53"W; **Identification:** identifiedBy: Allan Paulo Moreira dos Santos; **Event:** samplingProtocol: Malaise intercept trap; verbatimEventDate: 21.v.12; **Record Level:** institutionCode: DZRJ; basisOfRecord: PreservedSpecimen

##### Distribution

Brazil!: CE!. Paraguay. Argentina.

##### Notes

New species record for Brazil.

#### Ecnomidae

##### Notes

New family record for CE.

#### Austrotinodes

Schmid, 1955

##### Notes

New genus record for CE.

#### Austrotinodes paraguayensis

Flint, 1983

##### Materials

- **Type status:** Other material. **Occurrence:** recordedBy: Limeira-de-Oliveira | et al.; individualCount: 1; sex: male; lifeStage: adult; **Location:** country: Brazil; stateProvince: Ceará; municipality: Ubajara; locality: Parque Nacional de Ubajara, Rio Cafundó, pouco acima da cachoeira; maximumElevationInMeters: 795; verbatimCoordinates: 3°50'13"S, 40°54'35"W; **Identification:** identifiedBy: Wagner Rafael Maciel de Souza; **Event:** samplingProtocol: Malaise intercept trap; verbatimEventDate: 1.ii.13; **Record Level:** institutionCode: DZRJ; basisOfRecord: PreservedSpecimen
- **Type status:** Other material. **Occurrence:** recordedBy: Santos, A.P.M. | Takiya, D.M.; individualCount: 1; sex: male; lifeStage: adult; **Location:** country: Brazil; stateProvince: Ceará; municipality: Ubajara; locality: Parque Nacional de Ubajara, Trilha Samambaia, Mirante da cachoeira do Gameleira; maximumElevationInMeters: 880; verbatimCoordinates: 3°50'21"S, 40°54'23"W; **Identification:** identifiedBy: Wagner Rafael Maciel de Souza; **Event:** samplingProtocol: Pennsylvania light trap; verbatimEventDate: 13.ii.13; **Record Level:** institutionCode: DZRJ; basisOfRecord: PreservedSpecimen
- **Type status:** Other material. **Occurrence:** recordedBy: Limeira-de-Oliveira | et al.; individualCount: 3; sex: male; lifeStage: adult; **Location:** country: Brazil; stateProvince: Ceará; municipality: Ubajara; locality: Parque Nacional de Ubajara, Rio Cafundó, pouco acima da cachoeira; maximumElevationInMeters: 795; verbatimCoordinates: 3°50'13"S, 40°54'35"W; **Identification:** identifiedBy: Wagner Rafael Maciel de Souza; **Event:** samplingProtocol: Malaise intercept trap; verbatimEventDate: 13.xi.12; **Record Level:** institutionCode: DZRJ; basisOfRecord: PreservedSpecimen
- **Type status:** Other material. **Occurrence:** recordedBy: Santos, A.P.M. | Takiya, D.M.; individualCount: 2; sex: female; lifeStage: adult; **Location:** country: Brazil; stateProvince: Ceará; municipality: Ubajara; locality: Parque Nacional de Ubajara, Trilha Samambaia, Rio Gameleira; maximumElevationInMeters: 874; verbatimCoordinates: 3°50'25"S, 40°54'19"W; **Identification:** identifiedBy: Wagner Rafael Maciel de Souza; **Event:** samplingProtocol: Pennsylvania light trap; verbatimEventDate: 14.ii.13; **Record Level:** institutionCode: DZRJ; basisOfRecord: PreservedSpecimen
- **Type status:** Other material. **Occurrence:** recordedBy: Rafael, J.A. | Limeira-de-Oliveira, F. | Takiya, D.M. | Santos, A.P.M. | et al.; individualCount: 4; sex: male; lifeStage: adult; **Location:** country: Brazil; stateProvince: Ceará; municipality: Ubajara; locality: Parque Nacional de Ubajara, Trilha Araticum, Rio das Minas; maximumElevationInMeters: 524; verbatimCoordinates: 3°50'3"S, 40°54'18"W; **Identification:** identifiedBy: Wagner Rafael Maciel de Souza; **Event:** samplingProtocol: Malaise intercept trap; verbatimEventDate: 14.ii.13; **Record Level:** institutionCode: DZRJ; basisOfRecord: PreservedSpecimen
- **Type status:** Other material. **Occurrence:** recordedBy: Rafael, J.A. | Limeira-de-Oliveira, F. | Takiya, D.M. | Santos, A.P.M. | et al.; individualCount: 2; sex: female; lifeStage: adult; **Location:** country: Brazil; stateProvince: Ceará; municipality: Ubajara; locality: Parque Nacional de Ubajara, Trilha Araticum, Rio das Minas; maximumElevationInMeters: 524; verbatimCoordinates: 3°50'3"S, 40°54'18"W; **Identification:** identifiedBy: Wagner Rafael Maciel de Souza; **Event:** samplingProtocol: Malaise intercept trap; verbatimEventDate: 14.ii.13; **Record Level:** institutionCode: DZRJ; basisOfRecord: PreservedSpecimen
- **Type status:** Other material. **Occurrence:** recordedBy: Santos, A.P.M. | Takiya, D.M.; individualCount: 2; sex: male; lifeStage: adult; **Location:** country: Brazil; stateProvince: Ceará; municipality: Ubajara; locality: Parque Nacional de Ubajara, Cachoeira do Cafundó; maximumElevationInMeters: 783; verbatimCoordinates: 3°50'12"S, 40°54'35"W; **Identification:** identifiedBy: Wagner Rafael Maciel de Souza; **Event:** samplingProtocol: Pennsylvania light trap; verbatimEventDate: 15.ii.13; **Record Level:** institutionCode: DZRJ; basisOfRecord: PreservedSpecimen
- **Type status:** Other material. **Occurrence:** recordedBy: Takiya, D.M.; individualCount: 1; sex: female; lifeStage: adult; **Location:** country: Brazil; stateProvince: Ceará; municipality: Ubajara; locality: Parque Nacional de Ubajara, Trilha Samambaia, Rio Gameleira; maximumElevationInMeters: 874; verbatimCoordinates: 3°50'25"S, 40°54'19"W; **Identification:** identifiedBy: Wagner Rafael Maciel de Souza; **Event:** samplingProtocol: Pennsylvania light trap; verbatimEventDate: 18.iv.12; **Record Level:** institutionCode: DZRJ; basisOfRecord: PreservedSpecimen
- **Type status:** Other material. **Occurrence:** recordedBy: Rafael, J.A. | Limeira-de-Oliveira, F. | Takiya, D.M. | et al.; individualCount: 1; sex: male; lifeStage: adult; **Location:** country: Brazil; stateProvince: Ceará; municipality: Ubajara; locality: Parque Nacional de Ubajara, Trilha Samambaia, Rio Gameleira; maximumElevationInMeters: 874; verbatimCoordinates: 3°50'25"S, 40°54'19"W; **Identification:** identifiedBy: Wagner Rafael Maciel de Souza; **Event:** samplingProtocol: Malaise intercept trap; verbatimEventDate: 20.iv.12; **Record Level:** institutionCode: DZRJ; basisOfRecord: PreservedSpecimen
- **Type status:** Other material. **Occurrence:** recordedBy: Rafael, J.A. | Limeira-de-Oliveira, F. | Takiya, D.M. | et al.; individualCount: 1; sex: female; lifeStage: adult; **Location:** country: Brazil; stateProvince: Ceará; municipality: Ubajara; locality: Parque Nacional de Ubajara, Trilha Samambaia, Rio Gameleira; maximumElevationInMeters: 874; verbatimCoordinates: 3°50'25"S, 40°54'19"W; **Identification:** identifiedBy: Wagner Rafael Maciel de Souza; **Event:** samplingProtocol: Malaise intercept trap; verbatimEventDate: 20.iv.12; **Record Level:** institutionCode: DZRJ; basisOfRecord: PreservedSpecimen
- **Type status:** Other material. **Occurrence:** recordedBy: Rafael, J.A. | Limeira-de-Oliveira, F. | Takiya, D.M. | et al.; individualCount: 3; sex: male; lifeStage: adult; **Location:** country: Brazil; stateProvince: Ceará; municipality: Ubajara; locality: Parque Nacional de Ubajara, Trilha Samambaia, Rio Gameleira; maximumElevationInMeters: 874; verbatimCoordinates: 3°50'25"S, 40°54'19"W; **Identification:** identifiedBy: Wagner Rafael Maciel de Souza; **Event:** samplingProtocol: Malaise intercept trap; verbatimEventDate: 20.iv.12; **Record Level:** institutionCode: DZRJ; basisOfRecord: PreservedSpecimen
- **Type status:** Other material. **Occurrence:** recordedBy: Rafael, J.A. | Limeira-de-Oliveira, F. | Takiya, D.M. | et al.; individualCount: 3; sex: female; lifeStage: adult; **Location:** country: Brazil; stateProvince: Ceará; municipality: Ubajara; locality: Parque Nacional de Ubajara, Trilha Samambaia, Rio Gameleira; maximumElevationInMeters: 874; verbatimCoordinates: 3°50'25"S, 40°54'19"W; **Identification:** identifiedBy: Wagner Rafael Maciel de Souza; **Event:** samplingProtocol: Malaise intercept trap; verbatimEventDate: 20.iv.12; **Record Level:** institutionCode: DZRJ; basisOfRecord: PreservedSpecimen
- **Type status:** Other material. **Occurrence:** recordedBy: Rafael, J.A. | Limeira-de-Oliveira, F. | Takiya, D.M. | et al.; individualCount: 1; sex: male; lifeStage: adult; **Location:** country: Brazil; stateProvince: Ceará; municipality: Ubajara; locality: Parque Nacional de Ubajara, Trilha Araticum, Rio das Minas na altura da trilha do teleférico; maximumElevationInMeters: 420; verbatimCoordinates: 3°49'58"S, 40°53'53"W; **Identification:** identifiedBy: Wagner Rafael Maciel de Souza; **Event:** samplingProtocol: Malaise intercept trap; verbatimEventDate: 20.iv.12; **Record Level:** institutionCode: DZRJ; basisOfRecord: PreservedSpecimen
- **Type status:** Other material. **Occurrence:** recordedBy: Rafael, J.A. | Limeira-de-Oliveira, F. | Takiya, D.M. | et al.; individualCount: 1; sex: male; lifeStage: adult; **Location:** country: Brazil; stateProvince: Ceará; municipality: Ubajara; locality: Parque Nacional de Ubajara, Trilha Araticum, Rio das Minas na altura da trilha do teleférico; maximumElevationInMeters: 420; verbatimCoordinates: 3°49'58"S, 40°53'53"W; **Identification:** identifiedBy: Wagner Rafael Maciel de Souza; **Event:** samplingProtocol: Malaise intercept trap; verbatimEventDate: 20.iv.12; **Record Level:** institutionCode: DZRJ; basisOfRecord: PreservedSpecimen
- **Type status:** Other material. **Occurrence:** recordedBy: Rafael, J.A. | Limeira-de-Oliveira, F. | Takiya, D.M. | et al.; individualCount: 1; sex: male; lifeStage: adult; **Location:** country: Brazil; stateProvince: Ceará; municipality: Ubajara; locality: Parque Nacional de Ubajara, Trilha Araticum, Rio das Minas na altura da trilha do teleférico; maximumElevationInMeters: 420; verbatimCoordinates: 3°49'58"S, 40°53'53"W; **Identification:** identifiedBy: Wagner Rafael Maciel de Souza; **Event:** samplingProtocol: Malaise intercept trap; verbatimEventDate: 20.iv.12; **Record Level:** institutionCode: DZRJ; basisOfRecord: PreservedSpecimen
- **Type status:** Other material. **Occurrence:** recordedBy: Rafael, J.A. | Limeira-de-Oliveira, F. | Takiya, D.M. | et al.; individualCount: 1; sex: female; lifeStage: adult; **Location:** country: Brazil; stateProvince: Ceará; municipality: Ubajara; locality: Parque Nacional de Ubajara, Trilha Araticum, Rio das Minas na altura da trilha do teleférico; maximumElevationInMeters: 420; verbatimCoordinates: 3°49'58"S, 40°53'53"W; **Identification:** identifiedBy: Wagner Rafael Maciel de Souza; **Event:** samplingProtocol: Malaise intercept trap; verbatimEventDate: 20.iv.12; **Record Level:** institutionCode: DZRJ; basisOfRecord: PreservedSpecimen
- **Type status:** Other material. **Occurrence:** recordedBy: Rafael, J.A. | Limeira-de-Oliveira, F. | Takiya, D.M. | et al.; individualCount: 1; sex: male; lifeStage: adult; **Location:** country: Brazil; stateProvince: Ceará; municipality: Ubajara; locality: Parque Nacional de Ubajara, Trilha Araticum, Rio das Minas na altura da trilha do teleférico; maximumElevationInMeters: 420; verbatimCoordinates: 3°49'58"S, 40°53'53"W; **Identification:** identifiedBy: Wagner Rafael Maciel de Souza; **Event:** samplingProtocol: Malaise intercept trap; verbatimEventDate: 20.iv.12; **Record Level:** institutionCode: DZRJ; basisOfRecord: PreservedSpecimen
- **Type status:** Other material. **Occurrence:** recordedBy: Rafael, J.A. | Limeira-de-Oliveira, F. | Takiya, D.M. | et al.; individualCount: 13; sex: male; lifeStage: adult; **Location:** country: Brazil; stateProvince: Ceará; municipality: Ubajara; locality: Parque Nacional de Ubajara, Trilha Araticum, Rio das Minas na altura da trilha do teleférico; maximumElevationInMeters: 420; verbatimCoordinates: 3°49'58"S, 40°53'53"W; **Identification:** identifiedBy: Wagner Rafael Maciel de Souza; **Event:** samplingProtocol: Malaise intercept trap; verbatimEventDate: 20.iv.12; **Record Level:** institutionCode: DZRJ; basisOfRecord: PreservedSpecimen
- **Type status:** Other material. **Occurrence:** recordedBy: Rafael, J.A. | Limeira-de-Oliveira, F. | Takiya, D.M. | et al.; individualCount: 12; sex: female; lifeStage: adult; **Location:** country: Brazil; stateProvince: Ceará; municipality: Ubajara; locality: Parque Nacional de Ubajara, Trilha Araticum, Rio das Minas na altura da trilha do teleférico; maximumElevationInMeters: 420; verbatimCoordinates: 3°49'58"S, 40°53'53"W; **Identification:** identifiedBy: Wagner Rafael Maciel de Souza; **Event:** samplingProtocol: Malaise intercept trap; verbatimEventDate: 20.iv.12; **Record Level:** institutionCode: DZRJ; basisOfRecord: PreservedSpecimen
- **Type status:** Other material. **Occurrence:** recordedBy: Rafael, J.A. | Limeira-de-Oliveira, F. | Takiya, D.M. | et al.; individualCount: 1; sex: female; lifeStage: adult; **Location:** country: Brazil; stateProvince: Ceará; municipality: Ubajara; locality: Parque Nacional de Ubajara, Trilha Araticum, Rio das Minas na altura da trilha do teleférico; maximumElevationInMeters: 420; verbatimCoordinates: 3°49'58"S, 40°53'53"W; **Identification:** identifiedBy: Wagner Rafael Maciel de Souza; **Event:** samplingProtocol: Malaise intercept trap; verbatimEventDate: 20.iv.12; **Record Level:** institutionCode: DZRJ; basisOfRecord: PreservedSpecimen
- **Type status:** Other material. **Occurrence:** recordedBy: Takiya, D.M. | Câmara, J.T.; individualCount: 1; sex: male; lifeStage: adult; **Location:** country: Brazil; stateProvince: Ceará; municipality: Ubajara; locality: Parque Nacional de Ubajara, Trilha Samambaia, Rio Gameleira; maximumElevationInMeters: 874; verbatimCoordinates: 3°50'25"S, 40°54'19"W; **Identification:** identifiedBy: Wagner Rafael Maciel de Souza; **Event:** samplingProtocol: Pennsylvania light trap; verbatimEventDate: 21.iv.12; **Record Level:** institutionCode: DZRJ; basisOfRecord: PreservedSpecimen
- **Type status:** Other material. **Occurrence:** recordedBy: Limeira-de-Oliveira | et al.; individualCount: 6; sex: male; lifeStage: adult; **Location:** country: Brazil; stateProvince: Ceará; municipality: Ubajara; locality: Parque Nacional de Ubajara, Trilha Araticum, Rio das Minas na altura da trilha do teleférico; maximumElevationInMeters: 420; verbatimCoordinates: 3°49'58"S, 40°53'53"W; **Identification:** identifiedBy: Wagner Rafael Maciel de Souza; **Event:** samplingProtocol: Malaise intercept trap; verbatimEventDate: 21.v.12; **Record Level:** institutionCode: DZRJ; basisOfRecord: PreservedSpecimen
- **Type status:** Other material. **Occurrence:** recordedBy: Limeira-de-Oliveira | et al.; individualCount: 5; sex: female; lifeStage: adult; **Location:** country: Brazil; stateProvince: Ceará; municipality: Ubajara; locality: Parque Nacional de Ubajara, Trilha Araticum, Rio das Minas na altura da trilha do teleférico; maximumElevationInMeters: 420; verbatimCoordinates: 3°49'58"S, 40°53'53"W; **Identification:** identifiedBy: Wagner Rafael Maciel de Souza; **Event:** samplingProtocol: Malaise intercept trap; verbatimEventDate: 21.v.12; **Record Level:** institutionCode: DZRJ; basisOfRecord: PreservedSpecimen
- **Type status:** Other material. **Occurrence:** recordedBy: Takiya, D.M. | Câmara, J.T.; individualCount: 1; sex: male; lifeStage: adult; **Location:** country: Brazil; stateProvince: Ceará; municipality: Ubajara; locality: Parque Nacional de Ubajara, Trilha Samambaia, Mirante da cachoeira do Gameleira; maximumElevationInMeters: 880; verbatimCoordinates: 3°50'21"S, 40°54'23"W; **Identification:** identifiedBy: Wagner Rafael Maciel de Souza; **Event:** samplingProtocol: Pennsylvania light trap; verbatimEventDate: 23.iv.12; **Record Level:** institutionCode: DZRJ; basisOfRecord: PreservedSpecimen

##### Distribution

Brazil: PI!, CE!, MG. Paraguay.

##### Notes

New species record for Northeastern Brazil.

#### Helicopsychidae

#### Helicopsyche

von Siebold, 1856

#### Helicopsyche (Feropsyche) monda

Flint, 1983

##### Materials

- **Type status:** Other material. **Occurrence:** recordedBy: Santos, A.P.M. | Takiya, D.M.; individualCount: 1; sex: male; lifeStage: adult; **Location:** country: Brazil; stateProvince: Ceará; municipality: Ubajara; locality: Parque Nacional de Ubajara, Trilha Samambaia, Rio Gameleira; maximumElevationInMeters: 874; verbatimCoordinates: 3°50'25"S, 40°54'19"W; **Identification:** identifiedBy: Allan Paulo Moreira dos Santos; **Event:** samplingProtocol: Pennsylvania light trap; verbatimEventDate: 13.ii.13; **Record Level:** institutionCode: DZRJ; basisOfRecord: PreservedSpecimen
- **Type status:** Other material. **Occurrence:** recordedBy: Santos, A.P.M. | Takiya, D.M.; individualCount: 2; sex: female; lifeStage: adult; **Location:** country: Brazil; stateProvince: Ceará; municipality: Ubajara; locality: Parque Nacional de Ubajara, Trilha Samambaia, Rio Gameleira; maximumElevationInMeters: 874; verbatimCoordinates: 3°50'25"S, 40°54'19"W; **Identification:** identifiedBy: Allan Paulo Moreira dos Santos; **Event:** samplingProtocol: Pennsylvania light trap; verbatimEventDate: 13.ii.13; **Record Level:** institutionCode: DZRJ; basisOfRecord: PreservedSpecimen
- **Type status:** Other material. **Occurrence:** recordedBy: Santos, A.P.M. | Takiya, D.M.; individualCount: 4; sex: male; lifeStage: adult; **Location:** country: Brazil; stateProvince: Ceará; municipality: Ubajara; locality: Parque Nacional de Ubajara, Trilha Samambaia, Rio Gameleira; maximumElevationInMeters: 874; verbatimCoordinates: 3°50'25"S, 40°54'19"W; **Identification:** identifiedBy: Allan Paulo Moreira dos Santos; **Event:** samplingProtocol: Pennsylvania light trap; verbatimEventDate: 13.ii.13; **Record Level:** institutionCode: DZRJ; basisOfRecord: PreservedSpecimen
- **Type status:** Other material. **Occurrence:** recordedBy: Santos, A.P.M. | Takiya, D.M.; individualCount: 1; sex: female; lifeStage: adult; **Location:** country: Brazil; stateProvince: Ceará; municipality: Ubajara; locality: Parque Nacional de Ubajara, Trilha Samambaia, Rio Gameleira; maximumElevationInMeters: 874; verbatimCoordinates: 3°50'25"S, 40°54'19"W; **Identification:** identifiedBy: Allan Paulo Moreira dos Santos; **Event:** samplingProtocol: Pennsylvania light trap; verbatimEventDate: 13.ii.13; **Record Level:** institutionCode: DZRJ; basisOfRecord: PreservedSpecimen
- **Type status:** Other material. **Occurrence:** recordedBy: Santos, A.P.M. | Takiya, D.M.; individualCount: 1; sex: male; lifeStage: adult; **Location:** country: Brazil; stateProvince: Ceará; municipality: Ubajara; locality: Parque Nacional de Ubajara, Trilha Samambaia, Rio Gameleira; maximumElevationInMeters: 874; verbatimCoordinates: 3°50'25"S, 40°54'19"W; **Identification:** identifiedBy: Allan Paulo Moreira dos Santos; **Event:** samplingProtocol: Pennsylvania light trap; verbatimEventDate: 14.ii.13; **Record Level:** institutionCode: DZRJ; basisOfRecord: PreservedSpecimen
- **Type status:** Other material. **Occurrence:** recordedBy: Rafael, J.A. | Limeira-de-Oliveira, F. | Takiya, D.M. | Santos, A.P.M. | et al.; individualCount: 2; sex: male; lifeStage: adult; **Location:** country: Brazil; stateProvince: Ceará; municipality: Ubajara; locality: Parque Nacional de Ubajara, Trilha Araticum, Rio das Minas; maximumElevationInMeters: 524; verbatimCoordinates: 3°50'3"S, 40°54'18"W; **Identification:** identifiedBy: Allan Paulo Moreira dos Santos; **Event:** samplingProtocol: Malaise intercept trap; verbatimEventDate: 14.ii.13; **Record Level:** institutionCode: DZRJ; basisOfRecord: PreservedSpecimen
- **Type status:** Other material. **Occurrence:** recordedBy: Santos, A.P.M. | Takiya, D.M.; individualCount: 8; sex: male; lifeStage: adult; **Location:** country: Brazil; stateProvince: Ceará; municipality: Ubajara; locality: Parque Nacional de Ubajara, Cachoeira do Cafundó; maximumElevationInMeters: 783; verbatimCoordinates: 3°50'12"S, 40°54'35"W; **Identification:** identifiedBy: Allan Paulo Moreira dos Santos; **Event:** samplingProtocol: Pennsylvania light trap; verbatimEventDate: 15.ii.13; **Record Level:** institutionCode: DZRJ; basisOfRecord: PreservedSpecimen
- **Type status:** Other material. **Occurrence:** recordedBy: Santos, A.P.M. | Takiya, D.M.; individualCount: 2; sex: male; lifeStage: adult; **Location:** country: Brazil; stateProvince: Ceará; municipality: Ubajara; locality: Parque Nacional de Ubajara, Ponte sobre Rio Miranda; maximumElevationInMeters: 792; verbatimCoordinates: 3°50'7.4"S, 40°54'47.5"W; **Identification:** identifiedBy: Allan Paulo Moreira dos Santos; **Event:** samplingProtocol: Pennsylvania light trap; verbatimEventDate: 15.ii.13; **Record Level:** institutionCode: DZRJ; basisOfRecord: PreservedSpecimen
- **Type status:** Other material. **Occurrence:** recordedBy: Santos, A.P.M. | Takiya, D.M.; individualCount: 2; sex: female; lifeStage: adult; **Location:** country: Brazil; stateProvince: Ceará; municipality: Ubajara; locality: Parque Nacional de Ubajara, Ponte sobre Rio Miranda; maximumElevationInMeters: 792; verbatimCoordinates: 3°50'7.4"S, 40°54'47.5"W; **Identification:** identifiedBy: Allan Paulo Moreira dos Santos; **Event:** samplingProtocol: Pennsylvania light trap; verbatimEventDate: 15.ii.13; **Record Level:** institutionCode: DZRJ; basisOfRecord: PreservedSpecimen
- **Type status:** Other material. **Occurrence:** recordedBy: Rafael, J.A. | Limeira-de-Oliveira, F. | Takiya, D.M. | et al.; individualCount: 1; sex: male; lifeStage: adult; **Location:** country: Brazil; stateProvince: Ceará; municipality: Ubajara; locality: Parque Nacional de Ubajara, Trilha Araticum, Rio das Minas na altura da trilha do teleférico; maximumElevationInMeters: 420; verbatimCoordinates: 3°49'58"S, 40°53'53"W; **Identification:** identifiedBy: Allan Paulo Moreira dos Santos; **Event:** samplingProtocol: Malaise intercept trap; verbatimEventDate: 20.iv.12; **Record Level:** institutionCode: DZRJ; basisOfRecord: PreservedSpecimen
- **Type status:** Other material. **Occurrence:** recordedBy: Rafael, J.A. | Limeira-de-Oliveira, F. | Takiya, D.M. | et al.; individualCount: 1; sex: male; lifeStage: adult; **Location:** country: Brazil; stateProvince: Ceará; municipality: Ubajara; locality: Parque Nacional de Ubajara, Trilha Araticum, Rio das Minas na altura da trilha do teleférico; maximumElevationInMeters: 420; verbatimCoordinates: 3°49'58"S, 40°53'53"W; **Identification:** identifiedBy: Allan Paulo Moreira dos Santos; **Event:** samplingProtocol: Malaise intercept trap; verbatimEventDate: 20.iv.12; **Record Level:** institutionCode: DZRJ; basisOfRecord: PreservedSpecimen
- **Type status:** Other material. **Occurrence:** recordedBy: Takiya, D.M. | Câmara, J.T.; individualCount: 2; sex: male; lifeStage: adult; **Location:** country: Brazil; stateProvince: Ceará; municipality: Ubajara; locality: Parque Nacional de Ubajara, Trilha Samambaia, Rio Gameleira; maximumElevationInMeters: 874; verbatimCoordinates: 3°50'25"S, 40°54'19"W; **Identification:** identifiedBy: Allan Paulo Moreira dos Santos; **Event:** samplingProtocol: Pennsylvania light trap; verbatimEventDate: 21.iv.12; **Record Level:** institutionCode: DZRJ; basisOfRecord: PreservedSpecimen
- **Type status:** Other material. **Occurrence:** recordedBy: Takiya, D.M. | Câmara, J.T.; individualCount: 1; sex: female; lifeStage: adult; **Location:** country: Brazil; stateProvince: Ceará; municipality: Ubajara; locality: Parque Nacional de Ubajara, Trilha Samambaia, Rio Gameleira; maximumElevationInMeters: 874; verbatimCoordinates: 3°50'25"S, 40°54'19"W; **Identification:** identifiedBy: Allan Paulo Moreira dos Santos; **Event:** samplingProtocol: Pennsylvania light trap; verbatimEventDate: 21.iv.12; **Record Level:** institutionCode: DZRJ; basisOfRecord: PreservedSpecimen
- **Type status:** Other material. **Occurrence:** recordedBy: Takiya, D.M. | Câmara, J.T.; individualCount: 4; sex: male; lifeStage: adult; **Location:** country: Brazil; stateProvince: Ceará; municipality: Ubajara; locality: Parque Nacional de Ubajara, Trilha Samambaia, Rio Gameleira; maximumElevationInMeters: 874; verbatimCoordinates: 3°50'25"S, 40°54'19"W; **Identification:** identifiedBy: Allan Paulo Moreira dos Santos; **Event:** samplingProtocol: Pennsylvania light trap; verbatimEventDate: 21.iv.12; **Record Level:** institutionCode: DZRJ; basisOfRecord: PreservedSpecimen
- **Type status:** Other material. **Occurrence:** recordedBy: Rafael, J.A. | Limeira-de-Oliveira, F. | Takiya, D.M. | et al.; individualCount: 3; sex: male; lifeStage: adult; **Location:** country: Brazil; stateProvince: Ceará; municipality: Ubajara; locality: Parque Nacional de Ubajara, Rio Cafundó, pouco acima da cachoeira; maximumElevationInMeters: 795; verbatimCoordinates: 3°50'13"S, 40°54'35"W; **Identification:** identifiedBy: Allan Paulo Moreira dos Santos; **Event:** samplingProtocol: Malaise intercept trap; verbatimEventDate: 21.iv.12; **Record Level:** institutionCode: DZRJ; basisOfRecord: PreservedSpecimen
- **Type status:** Other material. **Occurrence:** recordedBy: Rafael, J.A. | Limeira-de-Oliveira, F. | Takiya, D.M. | et al.; individualCount: 1; sex: female; lifeStage: adult; **Location:** country: Brazil; stateProvince: Ceará; municipality: Ubajara; locality: Parque Nacional de Ubajara, Rio Cafundó, pouco acima da cachoeira; maximumElevationInMeters: 795; verbatimCoordinates: 3°50'13"S, 40°54'35"W; **Identification:** identifiedBy: Allan Paulo Moreira dos Santos; **Event:** samplingProtocol: Malaise intercept trap; verbatimEventDate: 21.iv.12; **Record Level:** institutionCode: DZRJ; basisOfRecord: PreservedSpecimen
- **Type status:** Other material. **Occurrence:** recordedBy: Takiya, D.M. | Câmara, J.T.; individualCount: 4; sex: male; lifeStage: adult; **Location:** country: Brazil; stateProvince: Ceará; municipality: Ubajara; locality: Parque Nacional de Ubajara, Trilha Samambaia, Mirante da cachoeira do Gameleira; maximumElevationInMeters: 880; verbatimCoordinates: 3°50'21"S, 40°54'23"W; **Identification:** identifiedBy: Allan Paulo Moreira dos Santos; **Event:** samplingProtocol: Pennsylvania light trap; verbatimEventDate: 23.iv.12; **Record Level:** institutionCode: DZRJ; basisOfRecord: PreservedSpecimen
- **Type status:** Other material. **Occurrence:** recordedBy: Takiya, D.M. | Câmara, J.T.; individualCount: 8; sex: female; lifeStage: adult; **Location:** country: Brazil; stateProvince: Ceará; municipality: Ubajara; locality: Parque Nacional de Ubajara, Trilha Samambaia, Mirante da cachoeira do Gameleira; maximumElevationInMeters: 880; verbatimCoordinates: 3°50'21"S, 40°54'23"W; **Identification:** identifiedBy: Allan Paulo Moreira dos Santos; **Event:** samplingProtocol: Pennsylvania light trap; verbatimEventDate: 23.iv.12; **Record Level:** institutionCode: DZRJ; basisOfRecord: PreservedSpecimen
- **Type status:** Other material. **Occurrence:** recordedBy: Takiya, D.M. | Rafael, J.A.; individualCount: 3; sex: male; lifeStage: adult; **Location:** country: Brazil; stateProvince: Ceará; municipality: Ubajara; locality: Parque Nacional de Ubajara, Rio Cafundó, pouco acima da cachoeira; maximumElevationInMeters: 795; verbatimCoordinates: 3°50'13"S, 40°54'35"W; **Identification:** identifiedBy: Allan Paulo Moreira dos Santos; **Event:** samplingProtocol: Pennsylvania light trap; verbatimEventDate: 24.iv.12; **Record Level:** institutionCode: DZRJ; basisOfRecord: PreservedSpecimen

##### Distribution

Brazil: CE!, MG, SP, SC.

##### Notes

New species record for Northeastern Brazil.

#### Helicopsyche (Feropsyche) vergelana

Ross, 1956

##### Materials

- **Type status:** Other material. **Occurrence:** recordedBy: Limeira-de-Oliveira | et al.; individualCount: 2; sex: male; lifeStage: adult; **Location:** country: Brazil; stateProvince: Ceará; municipality: Ubajara; locality: Parque Nacional de Ubajara, Rio Cafundó, pouco acima da cachoeira; maximumElevationInMeters: 795; verbatimCoordinates: 3°50'13"S, 40°54'35"W; **Identification:** identifiedBy: Allan Paulo Moreira dos Santos; **Event:** samplingProtocol: Malaise intercept trap; verbatimEventDate: 13.ix.12; **Record Level:** institutionCode: DZRJ; basisOfRecord: PreservedSpecimen
- **Type status:** Other material. **Occurrence:** recordedBy: Limeira-de-Oliveira | et al.; individualCount: 1; sex: male; lifeStage: adult; **Location:** country: Brazil; stateProvince: Ceará; municipality: Ubajara; locality: Parque Nacional de Ubajara, Rio Cafundó, pouco acima da cachoeira; maximumElevationInMeters: 795; verbatimCoordinates: 3°50'13"S, 40°54'35"W; **Identification:** identifiedBy: Allan Paulo Moreira dos Santos; **Event:** samplingProtocol: Malaise intercept trap; verbatimEventDate: 13.xi.12; **Record Level:** institutionCode: DZRJ; basisOfRecord: PreservedSpecimen
- **Type status:** Other material. **Occurrence:** recordedBy: Limeira-de-Oliveira | et al.; individualCount: 4; sex: female; lifeStage: adult; **Location:** country: Brazil; stateProvince: Ceará; municipality: Ubajara; locality: Parque Nacional de Ubajara, Rio Cafundó, pouco acima da cachoeira; maximumElevationInMeters: 795; verbatimCoordinates: 3°50'13"S, 40°54'35"W; **Identification:** identifiedBy: Allan Paulo Moreira dos Santos; **Event:** samplingProtocol: Malaise intercept trap; verbatimEventDate: 13.xi.12; **Record Level:** institutionCode: DZRJ; basisOfRecord: PreservedSpecimen
- **Type status:** Other material. **Occurrence:** recordedBy: Rafael, J.A. | Limeira-de-Oliveira, F. | Takiya, D.M. | Santos, A.P.M. | et al.; individualCount: 32; sex: male; lifeStage: adult; **Location:** country: Brazil; stateProvince: Ceará; municipality: Ubajara; locality: Parque Nacional de Ubajara, Trilha Araticum, Rio das Minas; maximumElevationInMeters: 524; verbatimCoordinates: 3°50'3"S, 40°54'18"W; **Identification:** identifiedBy: Allan Paulo Moreira dos Santos; **Event:** samplingProtocol: Malaise intercept trap; verbatimEventDate: 14.ii.13; **Record Level:** institutionCode: DZRJ; basisOfRecord: PreservedSpecimen
- **Type status:** Other material. **Occurrence:** recordedBy: Rafael, J.A. | Limeira-de-Oliveira, F. | Takiya, D.M. | Santos, A.P.M. | et al.; individualCount: 4; sex: male; lifeStage: adult; **Location:** country: Brazil; stateProvince: Ceará; municipality: Ubajara; locality: Parque Nacional de Ubajara, Trilha Araticum, Rio das Minas na altura da trilha do teleférico; maximumElevationInMeters: 420; verbatimCoordinates: 3°49'58"S, 40°53'53"W; **Identification:** identifiedBy: Allan Paulo Moreira dos Santos; **Event:** samplingProtocol: Malaise intercept trap; verbatimEventDate: 14.ii.13; **Record Level:** institutionCode: DZRJ; basisOfRecord: PreservedSpecimen
- **Type status:** Other material. **Occurrence:** recordedBy: Rafael, J.A. | Limeira-de-Oliveira, F. | Takiya, D.M. | Santos, A.P.M. | et al.; individualCount: 36; sex: female; lifeStage: adult; **Location:** country: Brazil; stateProvince: Ceará; municipality: Ubajara; locality: Parque Nacional de Ubajara, Trilha Araticum, Rio das Minas na altura da trilha do teleférico; maximumElevationInMeters: 420; verbatimCoordinates: 3°49'58"S, 40°53'53"W; **Identification:** identifiedBy: Allan Paulo Moreira dos Santos; **Event:** samplingProtocol: Malaise intercept trap; verbatimEventDate: 14.ii.13; **Record Level:** institutionCode: DZRJ; basisOfRecord: PreservedSpecimen
- **Type status:** Other material. **Occurrence:** recordedBy: Santos, A.P.M. | Takiya, D.M.; individualCount: 1; sex: male; lifeStage: adult; **Location:** country: Brazil; stateProvince: Ceará; municipality: Ubajara; locality: Parque Nacional de Ubajara, Trilha Araticum, Rio da Minas abaixo do teleférico; maximumElevationInMeters: 395; verbatimCoordinates: 3°49'43.3"S, 40°53'51.5"W; **Identification:** identifiedBy: Allan Paulo Moreira dos Santos; **Event:** samplingProtocol: Pennsylvania light trap; verbatimEventDate: 14.ii.13; **Record Level:** institutionCode: DZRJ; basisOfRecord: PreservedSpecimen
- **Type status:** Other material. **Occurrence:** recordedBy: Santos, A.P.M. | Takiya, D.M.; individualCount: 2; sex: male; lifeStage: adult; **Location:** country: Brazil; stateProvince: Ceará; municipality: Ubajara; locality: Parque Nacional de Ubajara, Cachoeira do Cafundó; maximumElevationInMeters: 783; verbatimCoordinates: 3°50'12"S, 40°54'35"W; **Identification:** identifiedBy: Allan Paulo Moreira dos Santos; **Event:** samplingProtocol: Pennsylvania light trap; verbatimEventDate: 15.ii.13; **Record Level:** institutionCode: DZRJ; basisOfRecord: PreservedSpecimen
- **Type status:** Other material. **Occurrence:** recordedBy: Rafael, J.A. | Limeira-de-Oliveira, F. | Takiya, D.M. | Santos, A.P.M. | et al.; individualCount: 4; sex: male; lifeStage: adult; **Location:** country: Brazil; stateProvince: Ceará; municipality: Ubajara; locality: Parque Nacional de Ubajara, Trilha Araticum, Rio das Minas; maximumElevationInMeters: 524; verbatimCoordinates: 3°50'3"S, 40°54'18"W; **Identification:** identifiedBy: Allan Paulo Moreira dos Santos; **Event:** samplingProtocol: Malaise intercept trap; verbatimEventDate: 17.ii.13; **Record Level:** institutionCode: DZRJ; basisOfRecord: PreservedSpecimen
- **Type status:** Other material. **Occurrence:** recordedBy: Rafael, J.A. | Limeira-de-Oliveira, F. | Takiya, D.M. | Santos, A.P.M. | et al.; individualCount: 4; sex: female; lifeStage: adult; **Location:** country: Brazil; stateProvince: Ceará; municipality: Ubajara; locality: Parque Nacional de Ubajara, Trilha Araticum, Rio das Minas; maximumElevationInMeters: 524; verbatimCoordinates: 3°50'3"S, 40°54'18"W; **Identification:** identifiedBy: Allan Paulo Moreira dos Santos; **Event:** samplingProtocol: Malaise intercept trap; verbatimEventDate: 17.ii.13; **Record Level:** institutionCode: DZRJ; basisOfRecord: PreservedSpecimen
- **Type status:** Other material. **Occurrence:** recordedBy: Rafael, J.A. | Limeira-de-Oliveira, F. | Takiya, D.M. | et al.; individualCount: 1; sex: male; lifeStage: adult; **Location:** country: Brazil; stateProvince: Ceará; municipality: Ubajara; locality: Parque Nacional de Ubajara, Trilha Araticum, Rio das Minas na altura da trilha do teleférico; maximumElevationInMeters: 420; verbatimCoordinates: 3°49'58"S, 40°53'53"W; **Identification:** identifiedBy: Allan Paulo Moreira dos Santos; **Event:** samplingProtocol: Malaise intercept trap; verbatimEventDate: 20.iv.12; **Record Level:** institutionCode: DZRJ; basisOfRecord: PreservedSpecimen
- **Type status:** Other material. **Occurrence:** recordedBy: Rafael, J.A. | Limeira-de-Oliveira, F. | Takiya, D.M. | et al.; individualCount: 1; sex: male; lifeStage: adult; **Location:** country: Brazil; stateProvince: Ceará; municipality: Ubajara; locality: Parque Nacional de Ubajara, Trilha Araticum, Rio das Minas na altura da trilha do teleférico; maximumElevationInMeters: 420; verbatimCoordinates: 3°49'58"S, 40°53'53"W; **Identification:** identifiedBy: Allan Paulo Moreira dos Santos; **Event:** samplingProtocol: Malaise intercept trap; verbatimEventDate: 20.iv.12; **Record Level:** institutionCode: DZRJ; basisOfRecord: PreservedSpecimen
- **Type status:** Other material. **Occurrence:** recordedBy: Rafael, J.A. | Limeira-de-Oliveira, F. | Takiya, D.M. | et al.; individualCount: 2; sex: female; lifeStage: adult; **Location:** country: Brazil; stateProvince: Ceará; municipality: Ubajara; locality: Parque Nacional de Ubajara, Trilha Araticum, Rio das Minas na altura da trilha do teleférico; maximumElevationInMeters: 420; verbatimCoordinates: 3°49'58"S, 40°53'53"W; **Identification:** identifiedBy: Allan Paulo Moreira dos Santos; **Event:** samplingProtocol: Malaise intercept trap; verbatimEventDate: 20.iv.12; **Record Level:** institutionCode: DZRJ; basisOfRecord: PreservedSpecimen
- **Type status:** Other material. **Occurrence:** recordedBy: Rafael, J.A. | Limeira-de-Oliveira, F. | Takiya, D.M. | et al.; individualCount: 3; sex: male; lifeStage: adult; **Location:** country: Brazil; stateProvince: Ceará; municipality: Ubajara; locality: Parque Nacional de Ubajara, Trilha Araticum, Rio das Minas na altura da trilha do teleférico; maximumElevationInMeters: 420; verbatimCoordinates: 3°49'58"S, 40°53'53"W; **Identification:** identifiedBy: Allan Paulo Moreira dos Santos; **Event:** samplingProtocol: Malaise intercept trap; verbatimEventDate: 20.iv.12; **Record Level:** institutionCode: DZRJ; basisOfRecord: PreservedSpecimen
- **Type status:** Other material. **Occurrence:** recordedBy: Rafael, J.A. | Limeira-de-Oliveira, F. | Takiya, D.M. | et al.; individualCount: 6; sex: male; lifeStage: adult; **Location:** country: Brazil; stateProvince: Ceará; municipality: Ubajara; locality: Parque Nacional de Ubajara, Trilha Araticum, Rio das Minas na altura da trilha do teleférico; maximumElevationInMeters: 420; verbatimCoordinates: 3°49'58"S, 40°53'53"W; **Identification:** identifiedBy: Allan Paulo Moreira dos Santos; **Event:** samplingProtocol: Malaise intercept trap; verbatimEventDate: 20.iv.12; **Record Level:** institutionCode: DZRJ; basisOfRecord: PreservedSpecimen
- **Type status:** Other material. **Occurrence:** recordedBy: Rafael, J.A. | Limeira-de-Oliveira, F. | Takiya, D.M. | et al.; individualCount: 11; sex: female; lifeStage: adult; **Location:** country: Brazil; stateProvince: Ceará; municipality: Ubajara; locality: Parque Nacional de Ubajara, Trilha Araticum, Rio das Minas na altura da trilha do teleférico; maximumElevationInMeters: 420; verbatimCoordinates: 3°49'58"S, 40°53'53"W; **Identification:** identifiedBy: Allan Paulo Moreira dos Santos; **Event:** samplingProtocol: Malaise intercept trap; verbatimEventDate: 20.iv.12; **Record Level:** institutionCode: DZRJ; basisOfRecord: PreservedSpecimen
- **Type status:** Other material. **Occurrence:** recordedBy: Rafael, J.A. | Limeira-de-Oliveira, F. | Takiya, D.M. | et al.; individualCount: 20; sex: male; lifeStage: adult; **Location:** country: Brazil; stateProvince: Ceará; municipality: Ubajara; locality: Parque Nacional de Ubajara, Trilha Araticum, Rio das Minas na altura da trilha do teleférico; maximumElevationInMeters: 420; verbatimCoordinates: 3°49'58"S, 40°53'53"W; **Identification:** identifiedBy: Allan Paulo Moreira dos Santos; **Event:** samplingProtocol: Malaise intercept trap; verbatimEventDate: 20.iv.12; **Record Level:** institutionCode: DZRJ; basisOfRecord: PreservedSpecimen
- **Type status:** Other material. **Occurrence:** recordedBy: Rafael, J.A. | Limeira-de-Oliveira, F. | Takiya, D.M. | et al.; individualCount: 15; sex: male; lifeStage: adult; **Location:** country: Brazil; stateProvince: Ceará; municipality: Ubajara; locality: Parque Nacional de Ubajara, Rio Cafundó, pouco acima da cachoeira; maximumElevationInMeters: 795; verbatimCoordinates: 3°50'13"S, 40°54'35"W; **Identification:** identifiedBy: Allan Paulo Moreira dos Santos; **Event:** samplingProtocol: Malaise intercept trap; verbatimEventDate: 21.iv.12; **Record Level:** institutionCode: DZRJ; basisOfRecord: PreservedSpecimen
- **Type status:** Other material. **Occurrence:** recordedBy: Limeira-de-Oliveira | et al.; individualCount: 18; sex: male; lifeStage: adult; **Location:** country: Brazil; stateProvince: Ceará; municipality: Ubajara; locality: Parque Nacional de Ubajara, Trilha Araticum, Rio das Minas na altura da trilha do teleférico; maximumElevationInMeters: 420; verbatimCoordinates: 3°49'58"S, 40°53'53"W; **Identification:** identifiedBy: Allan Paulo Moreira dos Santos; **Event:** samplingProtocol: Malaise intercept trap; verbatimEventDate: 21.v.12; **Record Level:** institutionCode: DZRJ; basisOfRecord: PreservedSpecimen
- **Type status:** Other material. **Occurrence:** recordedBy: Limeira-de-Oliveira | et al.; individualCount: 38; sex: female; lifeStage: adult; **Location:** country: Brazil; stateProvince: Ceará; municipality: Ubajara; locality: Parque Nacional de Ubajara, Trilha Araticum, Rio das Minas na altura da trilha do teleférico; maximumElevationInMeters: 420; verbatimCoordinates: 3°49'58"S, 40°53'53"W; **Identification:** identifiedBy: Allan Paulo Moreira dos Santos; **Event:** samplingProtocol: Malaise intercept trap; verbatimEventDate: 21.v.12; **Record Level:** institutionCode: DZRJ; basisOfRecord: PreservedSpecimen
- **Type status:** Other material. **Occurrence:** recordedBy: Takiya, D.M. | Somavilla, A.; individualCount: 2; sex: male; lifeStage: adult; **Location:** country: Brazil; stateProvince: Ceará; municipality: Ubajara; locality: Parque Nacional de Ubajara, Trilha Araticum, Rio das Minas; maximumElevationInMeters: 524; verbatimCoordinates: 3°50'3"S, 40°54'18"W; **Identification:** identifiedBy: Allan Paulo Moreira dos Santos; **Event:** samplingProtocol: Pennsylvania light trap; verbatimEventDate: 22.iv.12; **Record Level:** institutionCode: DZRJ; basisOfRecord: PreservedSpecimen
- **Type status:** Other material. **Occurrence:** recordedBy: Takiya, D.M. | Somavilla, A.; individualCount: 18; sex: female; lifeStage: adult; **Location:** country: Brazil; stateProvince: Ceará; municipality: Ubajara; locality: Parque Nacional de Ubajara, Trilha Araticum, Rio das Minas; maximumElevationInMeters: 524; verbatimCoordinates: 3°50'3"S, 40°54'18"W; **Identification:** identifiedBy: Allan Paulo Moreira dos Santos; **Event:** samplingProtocol: Pennsylvania light trap; verbatimEventDate: 22.iv.12; **Record Level:** institutionCode: DZRJ; basisOfRecord: PreservedSpecimen
- **Type status:** Other material. **Occurrence:** recordedBy: Takiya, D.M. | Rafael, J.A.; individualCount: 1; sex: male; lifeStage: adult; **Location:** country: Brazil; stateProvince: Ceará; municipality: Ubajara; locality: Parque Nacional de Ubajara, Rio Cafundó, pouco acima da cachoeira; maximumElevationInMeters: 795; verbatimCoordinates: 3°50'13"S, 40°54'35"W; **Identification:** identifiedBy: Allan Paulo Moreira dos Santos; **Event:** samplingProtocol: Pennsylvania light trap; verbatimEventDate: 24.iv.12; **Record Level:** institutionCode: DZRJ; basisOfRecord: PreservedSpecimen

##### Distribution

Mexico. Belize. Guatemala. Honduras. Costa Rica. Panama. Trinidad and Tobago. Venezuela. Suriname. Brazil: PI!, CE!, PE. Peru.

##### Notes

New species record for CE.

#### Hydropsychidae

#### Leptonema

Guérin, 1843

#### Leptonema spinulum

Flint, McAlpine & Ross, 1987

##### Materials

- **Type status:** Other material. **Occurrence:** recordedBy: Takiya, D.M. | Rafael, J.A.; individualCount: 1; sex: male; lifeStage: adult; **Location:** country: Brazil; stateProvince: Ceará; municipality: Ubajara; locality: Parque Nacional de Ubajara, Rio Cafundó, pouco acima da cachoeira; maximumElevationInMeters: 795; verbatimCoordinates: 3°50'13"S, 40°54'35"W; **Identification:** identifiedBy: Allan Paulo Moreira dos Santos; **Event:** samplingProtocol: Pennsylvania light trap; verbatimEventDate: 24.iv.12; **Record Level:** institutionCode: DZRJ; basisOfRecord: PreservedSpecimen

##### Distribution

Venezuela. Guyana. Brazil: PA, AM, CE!, MT, DF. Peru. Argentina.

##### Notes

New species record for Northeastern Brazil.

#### Leptonema viridianum

Navás, 1916

##### Materials

- **Type status:** Other material. **Occurrence:** recordedBy: Limeira-de-Oliveira | et al.; individualCount: 1; sex: female; lifeStage: adult; **Location:** country: Brazil; stateProvince: Ceará; municipality: Ubajara; locality: Parque Nacional de Ubajara, Rio Cafundó, pouco acima da cachoeira; maximumElevationInMeters: 795; verbatimCoordinates: 3°50'13"S, 40°54'35"W; **Identification:** identifiedBy: Allan Paulo Moreira dos Santos; **Event:** samplingProtocol: Malaise intercept trap; verbatimEventDate: 1.ii.13; **Record Level:** institutionCode: DZRJ; basisOfRecord: PreservedSpecimen
- **Type status:** Other material. **Occurrence:** recordedBy: Limeira-de-Oliveira | et al.; individualCount: 1; sex: male; lifeStage: adult; **Location:** country: Brazil; stateProvince: Ceará; municipality: Ubajara; locality: Parque Nacional de Ubajara, Rio Cafundó, pouco acima da cachoeira; maximumElevationInMeters: 795; verbatimCoordinates: 3°50'13"S, 40°54'35"W; **Identification:** identifiedBy: Allan Paulo Moreira dos Santos; **Event:** samplingProtocol: Malaise intercept trap; verbatimEventDate: 13.xi.12; **Record Level:** institutionCode: DZRJ; basisOfRecord: PreservedSpecimen
- **Type status:** Other material. **Occurrence:** recordedBy: Santos, A.P.M. | Takiya, D.M.; individualCount: 1; sex: female; lifeStage: adult; **Location:** country: Brazil; stateProvince: Ceará; municipality: Ubajara; locality: Parque Nacional de Ubajara, Trilha Araticum, Rio das Minas; maximumElevationInMeters: 524; verbatimCoordinates: 3°50'3"S, 40°54'18"W; **Identification:** identifiedBy: Allan Paulo Moreira dos Santos; **Event:** samplingProtocol: Pennsylvania light trap; verbatimEventDate: 14.ii.13; **Record Level:** institutionCode: DZRJ; basisOfRecord: PreservedSpecimen
- **Type status:** Other material. **Occurrence:** recordedBy: Santos, A.P.M. | Takiya, D.M.; individualCount: 1; sex: male; lifeStage: adult; **Location:** country: Brazil; stateProvince: Ceará; municipality: Ubajara; locality: Parque Nacional de Ubajara, Trilha Araticum, Rio das Minas; maximumElevationInMeters: 524; verbatimCoordinates: 3°50'3"S, 40°54'18"W; **Identification:** identifiedBy: Allan Paulo Moreira dos Santos; **Event:** samplingProtocol: Pennsylvania light trap; verbatimEventDate: 14.ii.13; **Record Level:** institutionCode: DZRJ; basisOfRecord: PreservedSpecimen
- **Type status:** Other material. **Occurrence:** recordedBy: Santos, A.P.M. | Takiya, D.M.; individualCount: 1; sex: female; lifeStage: adult; **Location:** country: Brazil; stateProvince: Ceará; municipality: Ubajara; locality: Parque Nacional de Ubajara, Trilha Araticum, Rio das Minas; maximumElevationInMeters: 524; verbatimCoordinates: 3°50'3"S, 40°54'18"W; **Identification:** identifiedBy: Allan Paulo Moreira dos Santos; **Event:** samplingProtocol: Pennsylvania light trap; verbatimEventDate: 14.ii.13; **Record Level:** institutionCode: DZRJ; basisOfRecord: PreservedSpecimen
- **Type status:** Other material. **Occurrence:** recordedBy: Santos, A.P.M. | Takiya, D.M.; individualCount: 5; sex: male; lifeStage: adult; **Location:** country: Brazil; stateProvince: Ceará; municipality: Ubajara; locality: Parque Nacional de Ubajara, Trilha Araticum, Rio das Minas; maximumElevationInMeters: 524; verbatimCoordinates: 3°50'3"S, 40°54'18"W; **Identification:** identifiedBy: Allan Paulo Moreira dos Santos; **Event:** samplingProtocol: Pennsylvania light trap; verbatimEventDate: 14.ii.13; **Record Level:** institutionCode: DZRJ; basisOfRecord: PreservedSpecimen
- **Type status:** Other material. **Occurrence:** recordedBy: Rafael, J.A. | Limeira-de-Oliveira, F. | Takiya, D.M. | Santos, A.P.M. | et al.; individualCount: 3; sex: male; lifeStage: adult; **Location:** country: Brazil; stateProvince: Ceará; municipality: Ubajara; locality: Parque Nacional de Ubajara, Trilha Araticum, Rio das Minas na altura da trilha do teleférico; maximumElevationInMeters: 420; verbatimCoordinates: 3°49'58"S, 40°53'53"W; **Identification:** identifiedBy: Allan Paulo Moreira dos Santos; **Event:** samplingProtocol: Malaise intercept trap; verbatimEventDate: 14.ii.13; **Record Level:** institutionCode: DZRJ; basisOfRecord: PreservedSpecimen
- **Type status:** Other material. **Occurrence:** recordedBy: Rafael, J.A. | Limeira-de-Oliveira, F. | Takiya, D.M. | Santos, A.P.M. | et al.; individualCount: 6; sex: female; lifeStage: adult; **Location:** country: Brazil; stateProvince: Ceará; municipality: Ubajara; locality: Parque Nacional de Ubajara, Rio das Minas, próximo ao Portăo Araticum; maximumElevationInMeters: 328; verbatimCoordinates: 3°49'32.6"S, 40°53'32.8"W; **Identification:** identifiedBy: Allan Paulo Moreira dos Santos; **Event:** samplingProtocol: Malaise intercept trap; verbatimEventDate: 14.ii.13; **Record Level:** institutionCode: DZRJ; basisOfRecord: PreservedSpecimen
- **Type status:** Other material. **Occurrence:** recordedBy: Santos, A.P.M. | Takiya, D.M.; individualCount: 3; sex: male; lifeStage: adult; **Location:** country: Brazil; stateProvince: Ceará; municipality: Ubajara; locality: Parque Nacional de Ubajara, Trilha Araticum, Rio das Minas; maximumElevationInMeters: 524; verbatimCoordinates: 3°50'3"S, 40°54'18"W; **Identification:** identifiedBy: Allan Paulo Moreira dos Santos; **Event:** samplingProtocol: Manual; verbatimEventDate: 17.ii.13; **Record Level:** institutionCode: DZRJ; basisOfRecord: PreservedSpecimen
- **Type status:** Other material. **Occurrence:** recordedBy: Rafael, J.A. | Limeira-de-Oliveira, F. | Takiya, D.M. | Santos, A.P.M. | et al.; individualCount: 3; sex: female; lifeStage: adult; **Location:** country: Brazil; stateProvince: Ceará; municipality: Ubajara; locality: Parque Nacional de Ubajara, Rio das Minas, próximo ao Portăo Araticum; maximumElevationInMeters: 328; verbatimCoordinates: 3°49'32.6"S, 40°53'32.8"W; **Identification:** identifiedBy: Allan Paulo Moreira dos Santos; **Event:** samplingProtocol: Malaise intercept trap; verbatimEventDate: 17.ii.13; **Record Level:** institutionCode: DZRJ; basisOfRecord: PreservedSpecimen
- **Type status:** Other material. **Occurrence:** recordedBy: Rafael, J.A. | Limeira-de-Oliveira, F. | Takiya, D.M. | et al.; individualCount: 2; sex: female; lifeStage: adult; **Location:** country: Brazil; stateProvince: Ceará; municipality: Ubajara; locality: Parque Nacional de Ubajara, Trilha Samambaia, Rio Gameleira; maximumElevationInMeters: 874; verbatimCoordinates: 3°50'25"S, 40°54'19"W; **Identification:** identifiedBy: Allan Paulo Moreira dos Santos; **Event:** samplingProtocol: Malaise intercept trap; verbatimEventDate: 20.iv.12; **Record Level:** institutionCode: DZRJ; basisOfRecord: PreservedSpecimen
- **Type status:** Other material. **Occurrence:** recordedBy: Rafael, J.A. | Limeira-de-Oliveira, F. | Takiya, D.M. | et al.; individualCount: 10; sex: male; lifeStage: adult; **Location:** country: Brazil; stateProvince: Ceará; municipality: Ubajara; locality: Parque Nacional de Ubajara, Trilha Araticum, Rio das Minas na altura da trilha do teleférico; maximumElevationInMeters: 420; verbatimCoordinates: 3°49'58"S, 40°53'53"W; **Identification:** identifiedBy: Allan Paulo Moreira dos Santos; **Event:** samplingProtocol: Malaise intercept trap; verbatimEventDate: 20.iv.12; **Record Level:** institutionCode: DZRJ; basisOfRecord: PreservedSpecimen
- **Type status:** Other material. **Occurrence:** recordedBy: Rafael, J.A. | Limeira-de-Oliveira, F. | Takiya, D.M. | et al.; individualCount: 57; sex: female; lifeStage: adult; **Location:** country: Brazil; stateProvince: Ceará; municipality: Ubajara; locality: Parque Nacional de Ubajara, Trilha Araticum, Rio das Minas na altura da trilha do teleférico; maximumElevationInMeters: 420; verbatimCoordinates: 3°49'58"S, 40°53'53"W; **Identification:** identifiedBy: Allan Paulo Moreira dos Santos; **Event:** samplingProtocol: Malaise intercept trap; verbatimEventDate: 20.iv.12; **Record Level:** institutionCode: DZRJ; basisOfRecord: PreservedSpecimen
- **Type status:** Other material. **Occurrence:** recordedBy: Rafael, J.A. | Limeira-de-Oliveira, F. | Takiya, D.M. | et al.; individualCount: 5; sex: male; lifeStage: adult; **Location:** country: Brazil; stateProvince: Ceará; municipality: Ubajara; locality: Parque Nacional de Ubajara, Trilha Araticum, Rio das Minas na altura da trilha do teleférico; maximumElevationInMeters: 420; verbatimCoordinates: 3°49'58"S, 40°53'53"W; **Identification:** identifiedBy: Allan Paulo Moreira dos Santos; **Event:** samplingProtocol: Malaise intercept trap; verbatimEventDate: 20.iv.12; **Record Level:** institutionCode: DZRJ; basisOfRecord: PreservedSpecimen
- **Type status:** Other material. **Occurrence:** recordedBy: Rafael, J.A. | Limeira-de-Oliveira, F. | Takiya, D.M. | et al.; individualCount: 52; sex: female; lifeStage: adult; **Location:** country: Brazil; stateProvince: Ceará; municipality: Ubajara; locality: Parque Nacional de Ubajara, Trilha Araticum, Rio das Minas na altura da trilha do teleférico; maximumElevationInMeters: 420; verbatimCoordinates: 3°49'58"S, 40°53'53"W; **Identification:** identifiedBy: Allan Paulo Moreira dos Santos; **Event:** samplingProtocol: Malaise intercept trap; verbatimEventDate: 20.iv.12; **Record Level:** institutionCode: DZRJ; basisOfRecord: PreservedSpecimen
- **Type status:** Other material. **Occurrence:** recordedBy: Takiya, D.M. | Câmara, J.T.; individualCount: 11; sex: male; lifeStage: adult; **Location:** country: Brazil; stateProvince: Ceará; municipality: Ubajara; locality: Parque Nacional de Ubajara, Trilha Araticum, Rio das Minas; maximumElevationInMeters: 524; verbatimCoordinates: 3°50'3"S, 40°54'18"W; **Identification:** identifiedBy: Allan Paulo Moreira dos Santos; **Event:** samplingProtocol: Pennsylvania light trap; verbatimEventDate: 21.iv.12; **Record Level:** institutionCode: DZRJ; basisOfRecord: PreservedSpecimen
- **Type status:** Other material. **Occurrence:** recordedBy: Takiya, D.M. | Câmara, J.T.; individualCount: 25; sex: female; lifeStage: adult; **Location:** country: Brazil; stateProvince: Ceará; municipality: Ubajara; locality: Parque Nacional de Ubajara, Trilha Araticum, Rio das Minas; maximumElevationInMeters: 524; verbatimCoordinates: 3°50'3"S, 40°54'18"W; **Identification:** identifiedBy: Allan Paulo Moreira dos Santos; **Event:** samplingProtocol: Pennsylvania light trap; verbatimEventDate: 21.iv.12; **Record Level:** institutionCode: DZRJ; basisOfRecord: PreservedSpecimen
- **Type status:** Other material. **Occurrence:** recordedBy: Takiya, D.M. | Somavilla, A.; individualCount: 2; sex: male; lifeStage: adult; **Location:** country: Brazil; stateProvince: Ceará; municipality: Ubajara; locality: Parque Nacional de Ubajara, Trilha Araticum, Rio das Minas; maximumElevationInMeters: 524; verbatimCoordinates: 3°50'3"S, 40°54'18"W; **Identification:** identifiedBy: Allan Paulo Moreira dos Santos; **Event:** samplingProtocol: Pennsylvania light trap; verbatimEventDate: 22.iv.12; **Record Level:** institutionCode: DZRJ; basisOfRecord: PreservedSpecimen
- **Type status:** Other material. **Occurrence:** recordedBy: Takiya, D.M. | Somavilla, A.; individualCount: 11; sex: female; lifeStage: adult; **Location:** country: Brazil; stateProvince: Ceará; municipality: Ubajara; locality: Parque Nacional de Ubajara, Trilha Araticum, Rio das Minas; maximumElevationInMeters: 524; verbatimCoordinates: 3°50'3"S, 40°54'18"W; **Identification:** identifiedBy: Allan Paulo Moreira dos Santos; **Event:** samplingProtocol: Pennsylvania light trap; verbatimEventDate: 22.iv.12; **Record Level:** institutionCode: DZRJ; basisOfRecord: PreservedSpecimen

##### Distribution

Colombia. Venezuela. Guyana. Brazil: PA, PI, CE, GO, MG, DF, ES, RJ. Ecuador. Peru. Bolivia. Paraguay. Argentina.

#### Macrostemum

Kolenati, 1859

#### Macrostemum hyalinum

(F.J. Pictet, 1836)

##### Materials

- **Type status:** Other material. **Occurrence:** recordedBy: Limeira-de-Oliveira | et al.; individualCount: 5; sex: male; lifeStage: adult; **Location:** country: Brazil; stateProvince: Ceará; municipality: Ubajara; locality: Parque Nacional de Ubajara, Rio Cafundó, pouco acima da cachoeira; maximumElevationInMeters: 795; verbatimCoordinates: 3°50'13"S, 40°54'35"W; **Identification:** identifiedBy: Allan Paulo Moreira dos Santos; **Event:** samplingProtocol: Malaise intercept trap; verbatimEventDate: 1.ii.13; **Record Level:** institutionCode: DZRJ; basisOfRecord: PreservedSpecimen
- **Type status:** Other material. **Occurrence:** recordedBy: Limeira-de-Oliveira | et al.; individualCount: 4; sex: female; lifeStage: adult; **Location:** country: Brazil; stateProvince: Ceará; municipality: Ubajara; locality: Parque Nacional de Ubajara, Rio Cafundó, pouco acima da cachoeira; maximumElevationInMeters: 795; verbatimCoordinates: 3°50'13"S, 40°54'35"W; **Identification:** identifiedBy: Allan Paulo Moreira dos Santos; **Event:** samplingProtocol: Malaise intercept trap; verbatimEventDate: 1.ii.13; **Record Level:** institutionCode: DZRJ; basisOfRecord: PreservedSpecimen
- **Type status:** Other material. **Occurrence:** recordedBy: Limeira-de-Oliveira | et al.; individualCount: 1; sex: male; lifeStage: adult; **Location:** country: Brazil; stateProvince: Ceará; municipality: Ubajara; locality: Parque Nacional de Ubajara, Rio Cafundó, pouco acima da cachoeira; maximumElevationInMeters: 795; verbatimCoordinates: 3°50'13"S, 40°54'35"W; **Identification:** identifiedBy: Allan Paulo Moreira dos Santos; **Event:** samplingProtocol: Malaise intercept trap; verbatimEventDate: 1.xii.12; **Record Level:** institutionCode: DZRJ; basisOfRecord: PreservedSpecimen
- **Type status:** Other material. **Occurrence:** recordedBy: Limeira-de-Oliveira | et al.; individualCount: 6; sex: female; lifeStage: adult; **Location:** country: Brazil; stateProvince: Ceará; municipality: Ubajara; locality: Parque Nacional de Ubajara, Rio Cafundó, pouco acima da cachoeira; maximumElevationInMeters: 795; verbatimCoordinates: 3°50'13"S, 40°54'35"W; **Identification:** identifiedBy: Allan Paulo Moreira dos Santos; **Event:** samplingProtocol: Malaise intercept trap; verbatimEventDate: 1.xii.12; **Record Level:** institutionCode: DZRJ; basisOfRecord: PreservedSpecimen
- **Type status:** Other material. **Occurrence:** recordedBy: Limeira-de-Oliveira | et al.; individualCount: 8; sex: male; lifeStage: adult; **Location:** country: Brazil; stateProvince: Ceará; municipality: Ubajara; locality: Parque Nacional de Ubajara, Rio Cafundó, pouco acima da cachoeira; maximumElevationInMeters: 795; verbatimCoordinates: 3°50'13"S, 40°54'35"W; **Identification:** identifiedBy: Allan Paulo Moreira dos Santos; **Event:** samplingProtocol: Malaise intercept trap; verbatimEventDate: 13.xi.12; **Record Level:** institutionCode: DZRJ; basisOfRecord: PreservedSpecimen
- **Type status:** Other material. **Occurrence:** recordedBy: Limeira-de-Oliveira | et al.; individualCount: 5; sex: female; lifeStage: adult; **Location:** country: Brazil; stateProvince: Ceará; municipality: Ubajara; locality: Parque Nacional de Ubajara, Rio Cafundó, pouco acima da cachoeira; maximumElevationInMeters: 795; verbatimCoordinates: 3°50'13"S, 40°54'35"W; **Identification:** identifiedBy: Allan Paulo Moreira dos Santos; **Event:** samplingProtocol: Malaise intercept trap; verbatimEventDate: 13.xi.12; **Record Level:** institutionCode: DZRJ; basisOfRecord: PreservedSpecimen
- **Type status:** Other material. **Occurrence:** recordedBy: Limeira-de-Oliveira | et al.; individualCount: 2; sex: female; lifeStage: adult; **Location:** country: Brazil; stateProvince: Ceará; municipality: Ubajara; locality: Parque Nacional de Ubajara, Rio Cafundó, pouco acima da cachoeira; maximumElevationInMeters: 795; verbatimCoordinates: 3°50'13"S, 40°54'35"W; **Identification:** identifiedBy: Allan Paulo Moreira dos Santos; **Event:** samplingProtocol: Malaise intercept trap; verbatimEventDate: 13.xi.12; **Record Level:** institutionCode: DZRJ; basisOfRecord: PreservedSpecimen
- **Type status:** Other material. **Occurrence:** recordedBy: Limeira-de-Oliveira | et al.; individualCount: 1; sex: female; lifeStage: adult; **Location:** country: Brazil; stateProvince: Ceará; municipality: Ubajara; locality: Parque Nacional de Ubajara, Rio Cafundó, pouco acima da cachoeira; maximumElevationInMeters: 795; verbatimCoordinates: 3°50'13"S, 40°54'35"W; **Identification:** identifiedBy: Allan Paulo Moreira dos Santos; **Event:** samplingProtocol: Malaise intercept trap; verbatimEventDate: 13.xi.12; **Record Level:** institutionCode: DZRJ; basisOfRecord: PreservedSpecimen
- **Type status:** Other material. **Occurrence:** recordedBy: Santos, A.P.M. | Takiya, D.M.; individualCount: 1; sex: female; lifeStage: adult; **Location:** country: Brazil; stateProvince: Ceará; municipality: Ubajara; locality: Parque Nacional de Ubajara, Trilha Samambaia, Rio Gameleira; maximumElevationInMeters: 874; verbatimCoordinates: 3°50'25"S, 40°54'19"W; **Identification:** identifiedBy: Allan Paulo Moreira dos Santos; **Event:** samplingProtocol: Pennsylvania light trap; verbatimEventDate: 14.ii.13; **Record Level:** institutionCode: DZRJ; basisOfRecord: PreservedSpecimen
- **Type status:** Other material. **Occurrence:** recordedBy: Rafael, J.A. | Limeira-de-Oliveira, F. | Takiya, D.M. | Santos, A.P.M. | et al.; individualCount: 3; sex: male; lifeStage: adult; **Location:** country: Brazil; stateProvince: Ceará; municipality: Ubajara; locality: Parque Nacional de Ubajara, Trilha Samambaia, Rio Gameleira; maximumElevationInMeters: 874; verbatimCoordinates: 3°50'25"S, 40°54'19"W; **Identification:** identifiedBy: Allan Paulo Moreira dos Santos; **Event:** samplingProtocol: Malaise intercept trap; verbatimEventDate: 14.ii.13; **Record Level:** institutionCode: DZRJ; basisOfRecord: PreservedSpecimen
- **Type status:** Other material. **Occurrence:** recordedBy: Rafael, J.A. | Limeira-de-Oliveira, F. | Takiya, D.M. | Santos, A.P.M. | et al.; individualCount: 2; sex: male; lifeStage: adult; **Location:** country: Brazil; stateProvince: Ceará; municipality: Ubajara; locality: Parque Nacional de Ubajara, Trilha Samambaia, Rio Gameleira; maximumElevationInMeters: 874; verbatimCoordinates: 3°50'25"S, 40°54'19"W; **Identification:** identifiedBy: Allan Paulo Moreira dos Santos; **Event:** samplingProtocol: Malaise intercept trap; verbatimEventDate: 17.ii.13; **Record Level:** institutionCode: DZRJ; basisOfRecord: PreservedSpecimen
- **Type status:** Other material. **Occurrence:** recordedBy: Rafael, J.A. | Limeira-de-Oliveira, F. | Takiya, D.M. | Santos, A.P.M. | et al.; individualCount: 2; sex: female; lifeStage: adult; **Location:** country: Brazil; stateProvince: Ceará; municipality: Ubajara; locality: Parque Nacional de Ubajara, Trilha Samambaia, Rio Gameleira; maximumElevationInMeters: 874; verbatimCoordinates: 3°50'25"S, 40°54'19"W; **Identification:** identifiedBy: Allan Paulo Moreira dos Santos; **Event:** samplingProtocol: Malaise intercept trap; verbatimEventDate: 17.ii.13; **Record Level:** institutionCode: DZRJ; basisOfRecord: PreservedSpecimen
- **Type status:** Other material. **Occurrence:** recordedBy: Limeira-de-Oliveira | et al.; individualCount: 1; sex: male; lifeStage: adult; **Location:** country: Brazil; stateProvince: Ceará; municipality: Ubajara; locality: Parque Nacional de Ubajara, Rio Cafundó, pouco acima da cachoeira; maximumElevationInMeters: 795; verbatimCoordinates: 3°50'13"S, 40°54'35"W; **Identification:** identifiedBy: Allan Paulo Moreira dos Santos; **Event:** samplingProtocol: Malaise intercept trap; verbatimEventDate: 18.xi.12; **Record Level:** institutionCode: DZRJ; basisOfRecord: PreservedSpecimen
- **Type status:** Other material. **Occurrence:** recordedBy: Limeira-de-Oliveira | et al.; individualCount: 4; sex: male; lifeStage: adult; **Location:** country: Brazil; stateProvince: Ceará; municipality: Ubajara; locality: Parque Nacional de Ubajara, Rio Cafundó, pouco acima da cachoeira; maximumElevationInMeters: 795; verbatimCoordinates: 3°50'13"S, 40°54'35"W; **Identification:** identifiedBy: Allan Paulo Moreira dos Santos; **Event:** samplingProtocol: Malaise intercept trap; verbatimEventDate: 18.xi.12; **Record Level:** institutionCode: DZRJ; basisOfRecord: PreservedSpecimen
- **Type status:** Other material. **Occurrence:** recordedBy: Rafael, J.A. | Limeira-de-Oliveira, F. | Takiya, D.M. | et al.; individualCount: 1; sex: male; lifeStage: adult; **Location:** country: Brazil; stateProvince: Ceará; municipality: Ubajara; locality: Parque Nacional de Ubajara, Trilha Samambaia, Rio Gameleira; maximumElevationInMeters: 874; verbatimCoordinates: 3°50'25"S, 40°54'19"W; **Identification:** identifiedBy: Allan Paulo Moreira dos Santos; **Event:** samplingProtocol: Malaise intercept trap; verbatimEventDate: 20.iv.12; **Record Level:** institutionCode: DZRJ; basisOfRecord: PreservedSpecimen
- **Type status:** Other material. **Occurrence:** recordedBy: Rafael, J.A. | Limeira-de-Oliveira, F. | Takiya, D.M. | et al.; individualCount: 3; sex: female; lifeStage: adult; **Location:** country: Brazil; stateProvince: Ceará; municipality: Ubajara; locality: Parque Nacional de Ubajara, Trilha Samambaia, Rio Gameleira; maximumElevationInMeters: 874; verbatimCoordinates: 3°50'25"S, 40°54'19"W; **Identification:** identifiedBy: Allan Paulo Moreira dos Santos; **Event:** samplingProtocol: Malaise intercept trap; verbatimEventDate: 20.iv.12; **Record Level:** institutionCode: DZRJ; basisOfRecord: PreservedSpecimen
- **Type status:** Other material. **Occurrence:** recordedBy: Rafael, J.A. | Limeira-de-Oliveira, F. | Takiya, D.M. | et al.; individualCount: 1; sex: male; lifeStage: adult; **Location:** country: Brazil; stateProvince: Ceará; municipality: Ubajara; locality: Parque Nacional de Ubajara, Trilha Samambaia, Rio Gameleira; maximumElevationInMeters: 874; verbatimCoordinates: 3°50'25"S, 40°54'19"W; **Identification:** identifiedBy: Allan Paulo Moreira dos Santos; **Event:** samplingProtocol: Malaise intercept trap; verbatimEventDate: 20.iv.12; **Record Level:** institutionCode: DZRJ; basisOfRecord: PreservedSpecimen
- **Type status:** Other material. **Occurrence:** recordedBy: Rafael, J.A. | Limeira-de-Oliveira, F. | Takiya, D.M. | et al.; individualCount: 2; sex: female; lifeStage: adult; **Location:** country: Brazil; stateProvince: Ceará; municipality: Ubajara; locality: Parque Nacional de Ubajara, Trilha Samambaia, Rio Gameleira; maximumElevationInMeters: 874; verbatimCoordinates: 3°50'25"S, 40°54'19"W; **Identification:** identifiedBy: Allan Paulo Moreira dos Santos; **Event:** samplingProtocol: Malaise intercept trap; verbatimEventDate: 20.iv.12; **Record Level:** institutionCode: DZRJ; basisOfRecord: PreservedSpecimen

##### Distribution

Colombia. Venezuela. Guyana. Brazil: PA, CE, PB, PE, MT, AC, BA, ES, SP, RJ, PR. Peru.

#### Macrostemum ulmeri

(Banks, 1913)

##### Materials

- **Type status:** Other material. **Occurrence:** recordedBy: Rafael, J.A. | Limeira-de-Oliveira, F. | Takiya, D.M. | Santos, A.P.M. | et al.; individualCount: 1; sex: male; lifeStage: adult; **Location:** country: Brazil; stateProvince: Ceará; municipality: Ubajara; locality: Parque Nacional de Ubajara, Trilha Araticum, Rio das Minas; maximumElevationInMeters: 524; verbatimCoordinates: 3°50'3"S, 40°54'18"W; **Identification:** identifiedBy: Allan Paulo Moreira dos Santos; **Event:** samplingProtocol: Malaise intercept trap; verbatimEventDate: 17.ii.13; **Record Level:** institutionCode: DZRJ; basisOfRecord: PreservedSpecimen
- **Type status:** Other material. **Occurrence:** recordedBy: Rafael, J.A. | Limeira-de-Oliveira, F. | Takiya, D.M. | et al.; individualCount: 1; sex: female; lifeStage: adult; **Location:** country: Brazil; stateProvince: Ceará; municipality: Ubajara; locality: Parque Nacional de Ubajara, Trilha Araticum, Rio das Minas na altura da trilha do teleférico; maximumElevationInMeters: 420; verbatimCoordinates: 3°49'58"S, 40°53'53"W; **Identification:** identifiedBy: Allan Paulo Moreira dos Santos; **Event:** samplingProtocol: Malaise intercept trap; verbatimEventDate: 20.iv.12; **Record Level:** institutionCode: DZRJ; basisOfRecord: PreservedSpecimen
- **Type status:** Other material. **Occurrence:** recordedBy: Limeira-de-Oliveira | et al.; individualCount: 1; sex: male; lifeStage: adult; **Location:** country: Brazil; stateProvince: Ceará; municipality: Ubajara; locality: Parque Nacional de Ubajara, Rio Cafundó, pouco acima da cachoeira; maximumElevationInMeters: 795; verbatimCoordinates: 3°50'13"S, 40°54'35"W; **Identification:** identifiedBy: Allan Paulo Moreira dos Santos; **Event:** samplingProtocol: Malaise intercept trap; verbatimEventDate: 1.xii.12; **Record Level:** institutionCode: DZRJ; basisOfRecord: PreservedSpecimen
- **Type status:** Other material. **Occurrence:** recordedBy: Limeira-de-Oliveira | et al.; individualCount: 6; sex: female; lifeStage: adult; **Location:** country: Brazil; stateProvince: Ceará; municipality: Ubajara; locality: Parque Nacional de Ubajara, Rio Cafundó, pouco acima da cachoeira; maximumElevationInMeters: 795; verbatimCoordinates: 3°50'13"S, 40°54'35"W; **Identification:** identifiedBy: Allan Paulo Moreira dos Santos; **Event:** samplingProtocol: Malaise intercept trap; verbatimEventDate: 1.xii.12; **Record Level:** institutionCode: DZRJ; basisOfRecord: PreservedSpecimen
- **Type status:** Other material. **Occurrence:** recordedBy: Limeira-de-Oliveira | et al.; individualCount: 8; sex: male; lifeStage: adult; **Location:** country: Brazil; stateProvince: Ceará; municipality: Ubajara; locality: Parque Nacional de Ubajara, Rio Cafundó, pouco acima da cachoeira; maximumElevationInMeters: 795; verbatimCoordinates: 3°50'13"S, 40°54'35"W; **Identification:** identifiedBy: Allan Paulo Moreira dos Santos; **Event:** samplingProtocol: Malaise intercept trap; verbatimEventDate: 13.xi.12; **Record Level:** institutionCode: DZRJ; basisOfRecord: PreservedSpecimen
- **Type status:** Other material. **Occurrence:** recordedBy: Limeira-de-Oliveira | et al.; individualCount: 5; sex: female; lifeStage: adult; **Location:** country: Brazil; stateProvince: Ceará; municipality: Ubajara; locality: Parque Nacional de Ubajara, Rio Cafundó, pouco acima da cachoeira; maximumElevationInMeters: 795; verbatimCoordinates: 3°50'13"S, 40°54'35"W; **Identification:** identifiedBy: Allan Paulo Moreira dos Santos; **Event:** samplingProtocol: Malaise intercept trap; verbatimEventDate: 13.xi.12; **Record Level:** institutionCode: DZRJ; basisOfRecord: PreservedSpecimen
- **Type status:** Other material. **Occurrence:** recordedBy: Limeira-de-Oliveira | et al.; individualCount: 2; sex: female; lifeStage: adult; **Location:** country: Brazil; stateProvince: Ceará; municipality: Ubajara; locality: Parque Nacional de Ubajara, Rio Cafundó, pouco acima da cachoeira; maximumElevationInMeters: 795; verbatimCoordinates: 3°50'13"S, 40°54'35"W; **Identification:** identifiedBy: Allan Paulo Moreira dos Santos; **Event:** samplingProtocol: Malaise intercept trap; verbatimEventDate: 13.xi.12; **Record Level:** institutionCode: DZRJ; basisOfRecord: PreservedSpecimen
- **Type status:** Other material. **Occurrence:** recordedBy: Limeira-de-Oliveira | et al.; individualCount: 1; sex: female; lifeStage: adult; **Location:** country: Brazil; stateProvince: Ceará; municipality: Ubajara; locality: Parque Nacional de Ubajara, Rio Cafundó, pouco acima da cachoeira; maximumElevationInMeters: 795; verbatimCoordinates: 3°50'13"S, 40°54'35"W; **Identification:** identifiedBy: Allan Paulo Moreira dos Santos; **Event:** samplingProtocol: Malaise intercept trap; verbatimEventDate: 13.xi.12; **Record Level:** institutionCode: DZRJ; basisOfRecord: PreservedSpecimen
- **Type status:** Other material. **Occurrence:** recordedBy: Santos, A.P.M. | Takiya, D.M.; individualCount: 1; sex: female; lifeStage: adult; **Location:** country: Brazil; stateProvince: Ceará; municipality: Ubajara; locality: Parque Nacional de Ubajara, Trilha Samambaia, Rio Gameleira; maximumElevationInMeters: 874; verbatimCoordinates: 3°50'25"S, 40°54'19"W; **Identification:** identifiedBy: Allan Paulo Moreira dos Santos; **Event:** samplingProtocol: Pennsylvania light trap; verbatimEventDate: 14.ii.13; **Record Level:** institutionCode: DZRJ; basisOfRecord: PreservedSpecimen
- **Type status:** Other material. **Occurrence:** recordedBy: Rafael, J.A. | Limeira-de-Oliveira, F. | Takiya, D.M. | Santos, A.P.M. | et al.; individualCount: 3; sex: male; lifeStage: adult; **Location:** country: Brazil; stateProvince: Ceará; municipality: Ubajara; locality: Parque Nacional de Ubajara, Trilha Samambaia, Rio Gameleira; maximumElevationInMeters: 874; verbatimCoordinates: 3°50'25"S, 40°54'19"W; **Identification:** identifiedBy: Allan Paulo Moreira dos Santos; **Event:** samplingProtocol: Malaise intercept trap; verbatimEventDate: 14.ii.13; **Record Level:** institutionCode: DZRJ; basisOfRecord: PreservedSpecimen
- **Type status:** Other material. **Occurrence:** recordedBy: Rafael, J.A. | Limeira-de-Oliveira, F. | Takiya, D.M. | Santos, A.P.M. | et al.; individualCount: 2; sex: male; lifeStage: adult; **Location:** country: Brazil; stateProvince: Ceará; municipality: Ubajara; locality: Parque Nacional de Ubajara, Trilha Samambaia, Rio Gameleira; maximumElevationInMeters: 874; verbatimCoordinates: 3°50'25"S, 40°54'19"W; **Identification:** identifiedBy: Allan Paulo Moreira dos Santos; **Event:** samplingProtocol: Malaise intercept trap; verbatimEventDate: 17.ii.13; **Record Level:** institutionCode: DZRJ; basisOfRecord: PreservedSpecimen
- **Type status:** Other material. **Occurrence:** recordedBy: Rafael, J.A. | Limeira-de-Oliveira, F. | Takiya, D.M. | Santos, A.P.M. | et al.; individualCount: 2; sex: female; lifeStage: adult; **Location:** country: Brazil; stateProvince: Ceará; municipality: Ubajara; locality: Parque Nacional de Ubajara, Trilha Samambaia, Rio Gameleira; maximumElevationInMeters: 874; verbatimCoordinates: 3°50'25"S, 40°54'19"W; **Identification:** identifiedBy: Allan Paulo Moreira dos Santos; **Event:** samplingProtocol: Malaise intercept trap; verbatimEventDate: 17.ii.13; **Record Level:** institutionCode: DZRJ; basisOfRecord: PreservedSpecimen
- **Type status:** Other material. **Occurrence:** recordedBy: Limeira-de-Oliveira | et al.; individualCount: 1; sex: male; lifeStage: adult; **Location:** country: Brazil; stateProvince: Ceará; municipality: Ubajara; locality: Parque Nacional de Ubajara, Rio Cafundó, pouco acima da cachoeira; maximumElevationInMeters: 795; verbatimCoordinates: 3°50'13"S, 40°54'35"W; **Identification:** identifiedBy: Allan Paulo Moreira dos Santos; **Event:** samplingProtocol: Malaise intercept trap; verbatimEventDate: 18.xi.12; **Record Level:** institutionCode: DZRJ; basisOfRecord: PreservedSpecimen
- **Type status:** Other material. **Occurrence:** recordedBy: Limeira-de-Oliveira | et al.; individualCount: 4; sex: male; lifeStage: adult; **Location:** country: Brazil; stateProvince: Ceará; municipality: Ubajara; locality: Parque Nacional de Ubajara, Rio Cafundó, pouco acima da cachoeira; maximumElevationInMeters: 795; verbatimCoordinates: 3°50'13"S, 40°54'35"W; **Identification:** identifiedBy: Allan Paulo Moreira dos Santos; **Event:** samplingProtocol: Malaise intercept trap; verbatimEventDate: 18.xi.12; **Record Level:** institutionCode: DZRJ; basisOfRecord: PreservedSpecimen
- **Type status:** Other material. **Occurrence:** recordedBy: Rafael, J.A. | Limeira-de-Oliveira, F. | Takiya, D.M. | et al.; individualCount: 1; sex: male; lifeStage: adult; **Location:** country: Brazil; stateProvince: Ceará; municipality: Ubajara; locality: Parque Nacional de Ubajara, Trilha Samambaia, Rio Gameleira; maximumElevationInMeters: 874; verbatimCoordinates: 3°50'25"S, 40°54'19"W; **Identification:** identifiedBy: Allan Paulo Moreira dos Santos; **Event:** samplingProtocol: Malaise intercept trap; verbatimEventDate: 20.iv.12; **Record Level:** institutionCode: DZRJ; basisOfRecord: PreservedSpecimen
- **Type status:** Other material. **Occurrence:** recordedBy: Rafael, J.A. | Limeira-de-Oliveira, F. | Takiya, D.M. | et al.; individualCount: 3; sex: female; lifeStage: adult; **Location:** country: Brazil; stateProvince: Ceará; municipality: Ubajara; locality: Parque Nacional de Ubajara, Trilha Samambaia, Rio Gameleira; maximumElevationInMeters: 874; verbatimCoordinates: 3°50'25"S, 40°54'19"W; **Identification:** identifiedBy: Allan Paulo Moreira dos Santos; **Event:** samplingProtocol: Malaise intercept trap; verbatimEventDate: 20.iv.12; **Record Level:** institutionCode: DZRJ; basisOfRecord: PreservedSpecimen
- **Type status:** Other material. **Occurrence:** recordedBy: Rafael, J.A. | Limeira-de-Oliveira, F. | Takiya, D.M. | et al.; individualCount: 1; sex: male; lifeStage: adult; **Location:** country: Brazil; stateProvince: Ceará; municipality: Ubajara; locality: Parque Nacional de Ubajara, Trilha Samambaia, Rio Gameleira; maximumElevationInMeters: 874; verbatimCoordinates: 3°50'25"S, 40°54'19"W; **Identification:** identifiedBy: Allan Paulo Moreira dos Santos; **Event:** samplingProtocol: Malaise intercept trap; verbatimEventDate: 20.iv.12; **Record Level:** institutionCode: DZRJ; basisOfRecord: PreservedSpecimen
- **Type status:** Other material. **Occurrence:** recordedBy: Rafael, J.A. | Limeira-de-Oliveira, F. | Takiya, D.M. | et al.; individualCount: 2; sex: female; lifeStage: adult; **Location:** country: Brazil; stateProvince: Ceará; municipality: Ubajara; locality: Parque Nacional de Ubajara, Trilha Samambaia, Rio Gameleira; maximumElevationInMeters: 874; verbatimCoordinates: 3°50'25"S, 40°54'19"W; **Identification:** identifiedBy: Allan Paulo Moreira dos Santos; **Event:** samplingProtocol: Malaise intercept trap; verbatimEventDate: 20.iv.12; **Record Level:** institutionCode: DZRJ; basisOfRecord: PreservedSpecimen

##### Distribution

Honduras. Costa Rica. Panama. Colombia. Suriname. Brazil: PA, AM, PI!, CE!, MT, AC, RO, SP. Ecuador. Peru.

##### Notes

New species record for CE.

#### Smicridea

McLachlan, 1871

#### Smicridea (Smicridea) bivittata

(Hagen, 1861)

##### Materials

- **Type status:** Other material. **Occurrence:** recordedBy: Rafael, J.A. | Limeira-de-Oliveira, F. | Takiya, D.M. | Santos, A.P.M. | et al.; individualCount: 1; sex: male; lifeStage: adult; **Location:** country: Brazil; stateProvince: Ceará; municipality: Ubajara; locality: Parque Nacional de Ubajara, Trilha Araticum, Rio das Minas; maximumElevationInMeters: 524; verbatimCoordinates: 3°50'3"S, 40°54'18"W; **Identification:** identifiedBy: Allan Paulo Moreira dos Santos; **Event:** samplingProtocol: Malaise intercept trap; verbatimEventDate: 17.ii.13; **Record Level:** institutionCode: DZRJ; basisOfRecord: PreservedSpecimen
- **Type status:** Other material. **Occurrence:** recordedBy: Rafael, J.A. | Limeira-de-Oliveira, F. | Takiya, D.M. | et al.; individualCount: 1; sex: male; lifeStage: adult; **Location:** country: Brazil; stateProvince: Ceará; municipality: Ubajara; locality: Parque Nacional de Ubajara, Trilha Araticum, Rio das Minas na altura da trilha do teleférico; maximumElevationInMeters: 420; verbatimCoordinates: 3°49'58"S, 40°53'53"W; **Identification:** identifiedBy: Allan Paulo Moreira dos Santos; **Event:** samplingProtocol: Malaise intercept trap; verbatimEventDate: 20.iv.12; **Record Level:** institutionCode: DZRJ; basisOfRecord: PreservedSpecimen
- **Type status:** Other material. **Occurrence:** recordedBy: Takiya, D.M. | Câmara, J.T.; individualCount: 8; sex: male; lifeStage: adult; **Location:** country: Brazil; stateProvince: Ceará; municipality: Ubajara; locality: Parque Nacional de Ubajara, Trilha Samambaia, Rio Gameleira; maximumElevationInMeters: 874; verbatimCoordinates: 3°50'25"S, 40°54'19"W; **Identification:** identifiedBy: Allan Paulo Moreira dos Santos; **Event:** samplingProtocol: Pennsylvania light trap; verbatimEventDate: 21.iv.12; **Record Level:** institutionCode: DZRJ; basisOfRecord: PreservedSpecimen
- **Type status:** Other material. **Occurrence:** recordedBy: Takiya, D.M. | Câmara, J.T.; individualCount: 6; sex: female; lifeStage: adult; **Location:** country: Brazil; stateProvince: Ceará; municipality: Ubajara; locality: Parque Nacional de Ubajara, Trilha Samambaia, Rio Gameleira; maximumElevationInMeters: 874; verbatimCoordinates: 3°50'25"S, 40°54'19"W; **Identification:** identifiedBy: Allan Paulo Moreira dos Santos; **Event:** samplingProtocol: Pennsylvania light trap; verbatimEventDate: 21.iv.12; **Record Level:** institutionCode: DZRJ; basisOfRecord: PreservedSpecimen
- **Type status:** Other material. **Occurrence:** recordedBy: Takiya, D.M. | Câmara, J.T.; individualCount: 18; sex: male; lifeStage: adult; **Location:** country: Brazil; stateProvince: Ceará; municipality: Ubajara; locality: Parque Nacional de Ubajara, Trilha Samambaia, Rio Gameleira; maximumElevationInMeters: 874; verbatimCoordinates: 3°50'25"S, 40°54'19"W; **Identification:** identifiedBy: Allan Paulo Moreira dos Santos; **Event:** samplingProtocol: Pennsylvania light trap; verbatimEventDate: 21.iv.12; **Record Level:** institutionCode: DZRJ; basisOfRecord: PreservedSpecimen
- **Type status:** Other material. **Occurrence:** recordedBy: Takiya, D.M. | Câmara, J.T.; individualCount: 10; sex: female; lifeStage: adult; **Location:** country: Brazil; stateProvince: Ceará; municipality: Ubajara; locality: Parque Nacional de Ubajara, Trilha Samambaia, Rio Gameleira; maximumElevationInMeters: 874; verbatimCoordinates: 3°50'25"S, 40°54'19"W; **Identification:** identifiedBy: Allan Paulo Moreira dos Santos; **Event:** samplingProtocol: Pennsylvania light trap; verbatimEventDate: 21.iv.12; **Record Level:** institutionCode: DZRJ; basisOfRecord: PreservedSpecimen
- **Type status:** Other material. **Occurrence:** recordedBy: Rafael, J.A. | Limeira-de-Oliveira, F. | Takiya, D.M. | et al.; individualCount: 2; sex: male; lifeStage: adult; **Location:** country: Brazil; stateProvince: Ceará; municipality: Ubajara; locality: Parque Nacional de Ubajara, Rio Cafundó, pouco acima da cachoeira; maximumElevationInMeters: 795; verbatimCoordinates: 3°50'13"S, 40°54'35"W; **Identification:** identifiedBy: Allan Paulo Moreira dos Santos; **Event:** samplingProtocol: Malaise intercept trap; verbatimEventDate: 21.iv.12; **Record Level:** institutionCode: DZRJ; basisOfRecord: PreservedSpecimen
- **Type status:** Other material. **Occurrence:** recordedBy: Takiya, D.M. | Somavilla, A.; individualCount: 1; sex: male; lifeStage: adult; **Location:** country: Brazil; stateProvince: Ceará; municipality: Ubajara; locality: Parque Nacional de Ubajara, Trilha Araticum, Rio das Minas; maximumElevationInMeters: 524; verbatimCoordinates: 3°50'3"S, 40°54'18"W; **Identification:** identifiedBy: Allan Paulo Moreira dos Santos; **Event:** samplingProtocol: Pennsylvania light trap; verbatimEventDate: 22.iv.12; **Record Level:** institutionCode: DZRJ; basisOfRecord: PreservedSpecimen
- **Type status:** Other material. **Occurrence:** recordedBy: Takiya, D.M. | Somavilla, A.; individualCount: 1; sex: female; lifeStage: adult; **Location:** country: Brazil; stateProvince: Ceará; municipality: Ubajara; locality: Parque Nacional de Ubajara, Trilha Araticum, Rio das Minas; maximumElevationInMeters: 524; verbatimCoordinates: 3°50'3"S, 40°54'18"W; **Identification:** identifiedBy: Allan Paulo Moreira dos Santos; **Event:** samplingProtocol: Pennsylvania light trap; verbatimEventDate: 22.iv.12; **Record Level:** institutionCode: DZRJ; basisOfRecord: PreservedSpecimen
- **Type status:** Other material. **Occurrence:** recordedBy: Takiya, D.M. | Rafael, J.A.; individualCount: 1; sex: male; lifeStage: adult; **Location:** country: Brazil; stateProvince: Ceará; municipality: Ubajara; locality: Parque Nacional de Ubajara, Rio Cafundó, pouco acima da cachoeira; maximumElevationInMeters: 795; verbatimCoordinates: 3°50'13"S, 40°54'35"W; **Identification:** identifiedBy: Allan Paulo Moreira dos Santos; **Event:** samplingProtocol: Pennsylvania light trap; verbatimEventDate: 24.iv.12; **Record Level:** institutionCode: DZRJ; basisOfRecord: PreservedSpecimen

##### Distribution

Mexico. Guatemala. Honduras. El Salvador. Costa Rica. Trinidad and Tobago. Venezuela. Suriname. Brazil: PA, PI!, CE!, MG, SP. Ecuador.

##### Notes

New species record for Northeastern Brazil.

#### Smicridea (Smicridea) franciscana

Rocha, Dumas & Nessimian, 2016

##### Materials

- **Type status:** Other material. **Occurrence:** recordedBy: Rafael, J.A. | Limeira-de-Oliveira, F. | Takiya, D.M. | Santos, A.P.M. | et al.; individualCount: 1; sex: male; lifeStage: adult; **Location:** country: Brazil; stateProvince: Ceará; municipality: Ubajara; locality: Parque Nacional de Ubajara, Trilha Araticum, Rio das Minas na altura da trilha do teleférico; maximumElevationInMeters: 420; verbatimCoordinates: 3°49'58"S, 40°53'53"W; **Identification:** identifiedBy: Allan Paulo Moreira dos Santos; **Event:** samplingProtocol: Malaise intercept trap; verbatimEventDate: 14.ii.13; **Record Level:** institutionCode: DZRJ; basisOfRecord: PreservedSpecimen
- **Type status:** Other material. **Occurrence:** recordedBy: Rafael, J.A. | Limeira-de-Oliveira, F. | Takiya, D.M. | Santos, A.P.M. | et al.; individualCount: 3; sex: male; lifeStage: adult; **Location:** country: Brazil; stateProvince: Ceará; municipality: Ubajara; locality: Parque Nacional de Ubajara, Rio das Minas, próximo ao Portão Araticum; maximumElevationInMeters: 328; verbatimCoordinates: 3°49'32.6"S, 40°53'32.8"W; **Identification:** identifiedBy: Allan Paulo Moreira dos Santos; **Event:** samplingProtocol: Malaise intercept trap; verbatimEventDate: 17.ii.13; **Record Level:** institutionCode: DZRJ; basisOfRecord: PreservedSpecimen
- **Type status:** Other material. **Occurrence:** recordedBy: Takiya, D.M. | Câmara, J.T.; individualCount: 8; sex: male; lifeStage: adult; **Location:** country: Brazil; stateProvince: Ceará; municipality: Ubajara; locality: Parque Nacional de Ubajara, Trilha Samambaia, Rio Gameleira; maximumElevationInMeters: 874; verbatimCoordinates: 3°50'25"S, 40°54'19"W; **Identification:** identifiedBy: Allan Paulo Moreira dos Santos; **Event:** samplingProtocol: Pennsylvania light trap; verbatimEventDate: 21.iv.12; **Record Level:** institutionCode: DZRJ; basisOfRecord: PreservedSpecimen
- **Type status:** Other material. **Occurrence:** recordedBy: Takiya, D.M. | Câmara, J.T.; individualCount: 6; sex: female; lifeStage: adult; **Location:** country: Brazil; stateProvince: Ceará; municipality: Ubajara; locality: Parque Nacional de Ubajara, Trilha Samambaia, Rio Gameleira; maximumElevationInMeters: 874; verbatimCoordinates: 3°50'25"S, 40°54'19"W; **Identification:** identifiedBy: Allan Paulo Moreira dos Santos; **Event:** samplingProtocol: Pennsylvania light trap; verbatimEventDate: 21.iv.12; **Record Level:** institutionCode: DZRJ; basisOfRecord: PreservedSpecimen
- **Type status:** Other material. **Occurrence:** recordedBy: Takiya, D.M. | Câmara, J.T.; individualCount: 18; sex: male; lifeStage: adult; **Location:** country: Brazil; stateProvince: Ceará; municipality: Ubajara; locality: Parque Nacional de Ubajara, Trilha Samambaia, Rio Gameleira; maximumElevationInMeters: 874; verbatimCoordinates: 3°50'25"S, 40°54'19"W; **Identification:** identifiedBy: Allan Paulo Moreira dos Santos; **Event:** samplingProtocol: Pennsylvania light trap; verbatimEventDate: 21.iv.12; **Record Level:** institutionCode: DZRJ; basisOfRecord: PreservedSpecimen
- **Type status:** Other material. **Occurrence:** recordedBy: Takiya, D.M. | Câmara, J.T.; individualCount: 10; sex: female; lifeStage: adult; **Location:** country: Brazil; stateProvince: Ceará; municipality: Ubajara; locality: Parque Nacional de Ubajara, Trilha Samambaia, Rio Gameleira; maximumElevationInMeters: 874; verbatimCoordinates: 3°50'25"S, 40°54'19"W; **Identification:** identifiedBy: Allan Paulo Moreira dos Santos; **Event:** samplingProtocol: Pennsylvania light trap; verbatimEventDate: 21.iv.12; **Record Level:** institutionCode: DZRJ; basisOfRecord: PreservedSpecimen
- **Type status:** Other material. **Occurrence:** recordedBy: Rafael, J.A. | Limeira-de-Oliveira, F. | Takiya, D.M. | et al.; individualCount: 2; sex: male; lifeStage: adult; **Location:** country: Brazil; stateProvince: Ceará; municipality: Ubajara; locality: Parque Nacional de Ubajara, Rio Cafundó, pouco acima da cachoeira; maximumElevationInMeters: 795; verbatimCoordinates: 3°50'13"S, 40°54'35"W; **Identification:** identifiedBy: Allan Paulo Moreira dos Santos; **Event:** samplingProtocol: Malaise intercept trap; verbatimEventDate: 21.iv.12; **Record Level:** institutionCode: DZRJ; basisOfRecord: PreservedSpecimen
- **Type status:** Other material. **Occurrence:** recordedBy: Takiya, D.M. | Somavilla, A.; individualCount: 1; sex: male; lifeStage: adult; **Location:** country: Brazil; stateProvince: Ceará; municipality: Ubajara; locality: Parque Nacional de Ubajara, Trilha Araticum, Rio das Minas; maximumElevationInMeters: 524; verbatimCoordinates: 3°50'3"S, 40°54'18"W; **Identification:** identifiedBy: Allan Paulo Moreira dos Santos; **Event:** samplingProtocol: Pennsylvania light trap; verbatimEventDate: 22.iv.12; **Record Level:** institutionCode: DZRJ; basisOfRecord: PreservedSpecimen
- **Type status:** Other material. **Occurrence:** recordedBy: Takiya, D.M. | Somavilla, A.; individualCount: 1; sex: female; lifeStage: adult; **Location:** country: Brazil; stateProvince: Ceará; municipality: Ubajara; locality: Parque Nacional de Ubajara, Trilha Araticum, Rio das Minas; maximumElevationInMeters: 524; verbatimCoordinates: 3°50'3"S, 40°54'18"W; **Identification:** identifiedBy: Allan Paulo Moreira dos Santos; **Event:** samplingProtocol: Pennsylvania light trap; verbatimEventDate: 22.iv.12; **Record Level:** institutionCode: DZRJ; basisOfRecord: PreservedSpecimen
- **Type status:** Other material. **Occurrence:** recordedBy: Takiya, D.M. | Rafael, J.A.; individualCount: 1; sex: male; lifeStage: adult; **Location:** country: Brazil; stateProvince: Ceará; municipality: Ubajara; locality: Parque Nacional de Ubajara, Rio Cafundó, pouco acima da cachoeira; maximumElevationInMeters: 795; verbatimCoordinates: 3°50'13"S, 40°54'35"W; **Identification:** identifiedBy: Allan Paulo Moreira dos Santos; **Event:** samplingProtocol: Pennsylvania light trap; verbatimEventDate: 24.iv.12; **Record Level:** institutionCode: DZRJ; basisOfRecord: PreservedSpecimen

##### Distribution

Brazil: CE!, MG.

##### Notes

New species record for Northeastern Brazil.

#### Smicridea (Smicridea) sp. 2*

##### Materials

- **Type status:** Other material. **Occurrence:** recordedBy: Limeira-de-Oliveira | et al.; individualCount: 3; sex: male; lifeStage: adult; **Location:** country: Brazil; stateProvince: Ceará; municipality: Ubajara; locality: Parque Nacional de Ubajara, Rio Cafundó, pouco acima da cachoeira; maximumElevationInMeters: 795; verbatimCoordinates: 3°50'13"S, 40°54'35"W; **Identification:** identifiedBy: Allan Paulo Moreira dos Santos; **Event:** samplingProtocol: Malaise intercept trap; verbatimEventDate: 1.ii.13; **Record Level:** institutionCode: DZRJ; basisOfRecord: PreservedSpecimen
- **Type status:** Other material. **Occurrence:** recordedBy: Limeira-de-Oliveira | et al.; individualCount: 1; sex: female; lifeStage: adult; **Location:** country: Brazil; stateProvince: Ceará; municipality: Ubajara; locality: Parque Nacional de Ubajara, Rio Cafundó, pouco acima da cachoeira; maximumElevationInMeters: 795; verbatimCoordinates: 3°50'13"S, 40°54'35"W; **Identification:** identifiedBy: Allan Paulo Moreira dos Santos; **Event:** samplingProtocol: Malaise intercept trap; verbatimEventDate: 1.ii.13; **Record Level:** institutionCode: DZRJ; basisOfRecord: PreservedSpecimen
- **Type status:** Other material. **Occurrence:** recordedBy: Limeira-de-Oliveira | et al.; individualCount: 1; sex: male; lifeStage: adult; **Location:** country: Brazil; stateProvince: Ceará; municipality: Ubajara; locality: Parque Nacional de Ubajara, Rio Cafundó, pouco acima da cachoeira; maximumElevationInMeters: 795; verbatimCoordinates: 3°50'13"S, 40°54'35"W; **Identification:** identifiedBy: Allan Paulo Moreira dos Santos; **Event:** samplingProtocol: Malaise intercept trap; verbatimEventDate: 13.ix.12; **Record Level:** institutionCode: DZRJ; basisOfRecord: PreservedSpecimen
- **Type status:** Other material. **Occurrence:** recordedBy: Limeira-de-Oliveira | et al.; individualCount: 8; sex: male; lifeStage: adult; **Location:** country: Brazil; stateProvince: Ceará; municipality: Ubajara; locality: Parque Nacional de Ubajara, Rio Cafundó, pouco acima da cachoeira; maximumElevationInMeters: 795; verbatimCoordinates: 3°50'13"S, 40°54'35"W; **Identification:** identifiedBy: Allan Paulo Moreira dos Santos; **Event:** samplingProtocol: Malaise intercept trap; verbatimEventDate: 13.xi.12; **Record Level:** institutionCode: DZRJ; basisOfRecord: PreservedSpecimen
- **Type status:** Other material. **Occurrence:** recordedBy: Limeira-de-Oliveira | et al.; individualCount: 5; sex: female; lifeStage: adult; **Location:** country: Brazil; stateProvince: Ceará; municipality: Ubajara; locality: Parque Nacional de Ubajara, Rio Cafundó, pouco acima da cachoeira; maximumElevationInMeters: 795; verbatimCoordinates: 3°50'13"S, 40°54'35"W; **Identification:** identifiedBy: Allan Paulo Moreira dos Santos; **Event:** samplingProtocol: Malaise intercept trap; verbatimEventDate: 13.xi.12; **Record Level:** institutionCode: DZRJ; basisOfRecord: PreservedSpecimen
- **Type status:** Other material. **Occurrence:** recordedBy: Rafael, J.A. | Limeira-de-Oliveira, F. | Takiya, D.M. | et al.; individualCount: 28; sex: male; lifeStage: adult; **Location:** country: Brazil; stateProvince: Ceará; municipality: Ubajara; locality: Parque Nacional de Ubajara, Trilha Samambaia, Rio Gameleira; maximumElevationInMeters: 874; verbatimCoordinates: 3°50'25"S, 40°54'19"W; **Identification:** identifiedBy: Allan Paulo Moreira dos Santos; **Event:** samplingProtocol: Malaise intercept trap; verbatimEventDate: 20.iv.12; **Record Level:** institutionCode: DZRJ; basisOfRecord: PreservedSpecimen
- **Type status:** Other material. **Occurrence:** recordedBy: Rafael, J.A. | Limeira-de-Oliveira, F. | Takiya, D.M. | et al.; individualCount: 48; sex: male; lifeStage: adult; **Location:** country: Brazil; stateProvince: Ceará; municipality: Ubajara; locality: Parque Nacional de Ubajara, Trilha Samambaia, Rio Gameleira; maximumElevationInMeters: 874; verbatimCoordinates: 3°50'25"S, 40°54'19"W; **Identification:** identifiedBy: Allan Paulo Moreira dos Santos; **Event:** samplingProtocol: Malaise intercept trap; verbatimEventDate: 20.iv.12; **Record Level:** institutionCode: DZRJ; basisOfRecord: PreservedSpecimen

##### Notes

Undescribed species.

#### Smicridea (Smicridea) sp. 3

##### Materials

- **Type status:** Other material. **Occurrence:** recordedBy: Limeira-de-Oliveira | et al.; individualCount: 2; sex: male; lifeStage: adult; **Location:** country: Brazil; stateProvince: Ceará; municipality: Ubajara; locality: Parque Nacional de Ubajara, Rio Cafundó, pouco acima da cachoeira; maximumElevationInMeters: 795; verbatimCoordinates: 3°50'13"S, 40°54'35"W; **Identification:** identifiedBy: Allan Paulo Moreira dos Santos; **Event:** samplingProtocol: Malaise intercept trap; verbatimEventDate: 13.xi.12; **Record Level:** institutionCode: DZRJ; basisOfRecord: PreservedSpecimen
- **Type status:** Other material. **Occurrence:** recordedBy: Limeira-de-Oliveira | et al.; individualCount: 2; sex: female; lifeStage: adult; **Location:** country: Brazil; stateProvince: Ceará; municipality: Ubajara; locality: Parque Nacional de Ubajara, Rio Cafundó, pouco acima da cachoeira; maximumElevationInMeters: 795; verbatimCoordinates: 3°50'13"S, 40°54'35"W; **Identification:** identifiedBy: Allan Paulo Moreira dos Santos; **Event:** samplingProtocol: Malaise intercept trap; verbatimEventDate: 13.xi.12; **Record Level:** institutionCode: DZRJ; basisOfRecord: PreservedSpecimen
- **Type status:** Other material. **Occurrence:** recordedBy: Rafael, J.A. | Limeira-de-Oliveira, F. | Takiya, D.M. | Santos, A.P.M. | et al.; individualCount: 5; sex: male; lifeStage: adult; **Location:** country: Brazil; stateProvince: Ceará; municipality: Ubajara; locality: Parque Nacional de Ubajara, Trilha Araticum, Rio das Minas; maximumElevationInMeters: 524; verbatimCoordinates: 3°50'3"S, 40°54'18"W; **Identification:** identifiedBy: Allan Paulo Moreira dos Santos; **Event:** samplingProtocol: Malaise intercept trap; verbatimEventDate: 14.ii.13; **Record Level:** institutionCode: DZRJ; basisOfRecord: PreservedSpecimen
- **Type status:** Other material. **Occurrence:** recordedBy: Rafael, J.A. | Limeira-de-Oliveira, F. | Takiya, D.M. | Santos, A.P.M. | et al.; individualCount: 1; sex: female; lifeStage: adult; **Location:** country: Brazil; stateProvince: Ceará; municipality: Ubajara; locality: Parque Nacional de Ubajara, Trilha Araticum, Rio das Minas; maximumElevationInMeters: 524; verbatimCoordinates: 3°50'3"S, 40°54'18"W; **Identification:** identifiedBy: Allan Paulo Moreira dos Santos; **Event:** samplingProtocol: Malaise intercept trap; verbatimEventDate: 14.ii.13; **Record Level:** institutionCode: DZRJ; basisOfRecord: PreservedSpecimen
- **Type status:** Other material. **Occurrence:** recordedBy: Rafael, J.A. | Limeira-de-Oliveira, F. | Takiya, D.M. | Santos, A.P.M. | et al.; individualCount: 2; sex: male; lifeStage: adult; **Location:** country: Brazil; stateProvince: Ceará; municipality: Ubajara; locality: Parque Nacional de Ubajara, Trilha Araticum, Rio das Minas na altura da trilha do teleférico; maximumElevationInMeters: 420; verbatimCoordinates: 3°49'58"S, 40°53'53"W; **Identification:** identifiedBy: Allan Paulo Moreira dos Santos; **Event:** samplingProtocol: Malaise intercept trap; verbatimEventDate: 14.ii.13; **Record Level:** institutionCode: DZRJ; basisOfRecord: PreservedSpecimen
- **Type status:** Other material. **Occurrence:** recordedBy: Rafael, J.A. | Limeira-de-Oliveira, F. | Takiya, D.M. | Santos, A.P.M. | et al.; individualCount: 1; sex: male; lifeStage: adult; **Location:** country: Brazil; stateProvince: Ceará; municipality: Ubajara; locality: Parque Nacional de Ubajara, Trilha Araticum, Rio das Minas; maximumElevationInMeters: 524; verbatimCoordinates: 3°50'3"S, 40°54'18"W; **Identification:** identifiedBy: Allan Paulo Moreira dos Santos; **Event:** samplingProtocol: Malaise intercept trap; verbatimEventDate: 17.ii.13; **Record Level:** institutionCode: DZRJ; basisOfRecord: PreservedSpecimen
- **Type status:** Other material. **Occurrence:** recordedBy: Rafael, J.A. | Limeira-de-Oliveira, F. | Takiya, D.M. | Santos, A.P.M. | et al.; individualCount: 2; sex: female; lifeStage: adult; **Location:** country: Brazil; stateProvince: Ceará; municipality: Ubajara; locality: Parque Nacional de Ubajara, Trilha Araticum, Rio das Minas; maximumElevationInMeters: 524; verbatimCoordinates: 3°50'3"S, 40°54'18"W; **Identification:** identifiedBy: Allan Paulo Moreira dos Santos; **Event:** samplingProtocol: Malaise intercept trap; verbatimEventDate: 17.ii.13; **Record Level:** institutionCode: DZRJ; basisOfRecord: PreservedSpecimen
- **Type status:** Other material. **Occurrence:** recordedBy: Rafael, J.A. | Limeira-de-Oliveira, F. | Takiya, D.M. | et al.; individualCount: 1; sex: male; lifeStage: adult; **Location:** country: Brazil; stateProvince: Ceará; municipality: Ubajara; locality: Parque Nacional de Ubajara, Trilha Samambaia, Rio Gameleira; maximumElevationInMeters: 874; verbatimCoordinates: 3°50'25"S, 40°54'19"W; **Identification:** identifiedBy: Allan Paulo Moreira dos Santos; **Event:** samplingProtocol: Malaise intercept trap; verbatimEventDate: 20.iv.12; **Record Level:** institutionCode: DZRJ; basisOfRecord: PreservedSpecimen
- **Type status:** Other material. **Occurrence:** recordedBy: Rafael, J.A. | Limeira-de-Oliveira, F. | Takiya, D.M. | et al.; individualCount: 2; sex: male; lifeStage: adult; **Location:** country: Brazil; stateProvince: Ceará; municipality: Ubajara; locality: Parque Nacional de Ubajara, Trilha Samambaia, Rio Gameleira; maximumElevationInMeters: 874; verbatimCoordinates: 3°50'25"S, 40°54'19"W; **Identification:** identifiedBy: Allan Paulo Moreira dos Santos; **Event:** samplingProtocol: Malaise intercept trap; verbatimEventDate: 20.iv.12; **Record Level:** institutionCode: DZRJ; basisOfRecord: PreservedSpecimen
- **Type status:** Other material. **Occurrence:** recordedBy: Rafael, J.A. | Limeira-de-Oliveira, F. | Takiya, D.M. | et al.; individualCount: 1; sex: male; lifeStage: adult; **Location:** country: Brazil; stateProvince: Ceará; municipality: Ubajara; locality: Parque Nacional de Ubajara, Trilha Araticum, Rio das Minas na altura da trilha do teleférico; maximumElevationInMeters: 420; verbatimCoordinates: 3°49'58"S, 40°53'53"W; **Identification:** identifiedBy: Allan Paulo Moreira dos Santos; **Event:** samplingProtocol: Malaise intercept trap; verbatimEventDate: 20.iv.12; **Record Level:** institutionCode: DZRJ; basisOfRecord: PreservedSpecimen
- **Type status:** Other material. **Occurrence:** recordedBy: Rafael, J.A. | Limeira-de-Oliveira, F. | Takiya, D.M. | et al.; individualCount: 1; sex: male; lifeStage: adult; **Location:** country: Brazil; stateProvince: Ceará; municipality: Ubajara; locality: Parque Nacional de Ubajara, Trilha Araticum, Rio das Minas na altura da trilha do teleférico; maximumElevationInMeters: 420; verbatimCoordinates: 3°49'58"S, 40°53'53"W; **Identification:** identifiedBy: Allan Paulo Moreira dos Santos; **Event:** samplingProtocol: Malaise intercept trap; verbatimEventDate: 20.iv.12; **Record Level:** institutionCode: DZRJ; basisOfRecord: PreservedSpecimen
- **Type status:** Other material. **Occurrence:** recordedBy: Rafael, J.A. | Limeira-de-Oliveira, F. | Takiya, D.M. | et al.; individualCount: 1; sex: male; lifeStage: adult; **Location:** country: Brazil; stateProvince: Ceará; municipality: Ubajara; locality: Parque Nacional de Ubajara, Rio Cafundó, pouco acima da cachoeira; maximumElevationInMeters: 795; verbatimCoordinates: 3°50'13"S, 40°54'35"W; **Identification:** identifiedBy: Allan Paulo Moreira dos Santos; **Event:** samplingProtocol: Malaise intercept trap; verbatimEventDate: 21.iv.12; **Record Level:** institutionCode: DZRJ; basisOfRecord: PreservedSpecimen

##### Notes

Undescribed species.

#### Hydroptilidae

##### Notes

Family firstly recorded from CE in Souza et al. 2014b.

#### Alisotrichia

Flint, 1964

##### Notes

New genus record for CE.

#### Alisotrichia sp. 1

##### Materials

- **Type status:** Other material. **Occurrence:** recordedBy: Limeira-de-Oliveira | et al.; individualCount: 23; sex: male; lifeStage: adult; **Location:** country: Brazil; stateProvince: Ceará; municipality: Ubajara; locality: Parque Nacional de Ubajara, Rio Cafundó, pouco acima da cachoeira; maximumElevationInMeters: 795; verbatimCoordinates: 3°50'13"S, 40°54'35"W; **Identification:** identifiedBy: Allan Paulo Moreira dos Santos; **Event:** samplingProtocol: Malaise intercept trap; verbatimEventDate: 13.ix.12; **Record Level:** institutionCode: DZRJ; basisOfRecord: PreservedSpecimen
- **Type status:** Other material. **Occurrence:** recordedBy: Limeira-de-Oliveira | et al.; individualCount: 10; sex: female; lifeStage: adult; **Location:** country: Brazil; stateProvince: Ceará; municipality: Ubajara; locality: Parque Nacional de Ubajara, Rio Cafundó, pouco acima da cachoeira; maximumElevationInMeters: 795; verbatimCoordinates: 3°50'13"S, 40°54'35"W; **Identification:** identifiedBy: Allan Paulo Moreira dos Santos; **Event:** samplingProtocol: Malaise intercept trap; verbatimEventDate: 13.ix.12; **Record Level:** institutionCode: DZRJ; basisOfRecord: PreservedSpecimen
- **Type status:** Other material. **Occurrence:** recordedBy: Limeira-de-Oliveira | et al.; individualCount: 23; sex: male; lifeStage: adult; **Location:** country: Brazil; stateProvince: Ceará; municipality: Ubajara; locality: Parque Nacional de Ubajara, Rio Cafundó, pouco acima da cachoeira; maximumElevationInMeters: 795; verbatimCoordinates: 3°50'13"S, 40°54'35"W; **Identification:** identifiedBy: Allan Paulo Moreira dos Santos; **Event:** samplingProtocol: Malaise intercept trap; verbatimEventDate: 13.ix.12; **Record Level:** institutionCode: DZRJ; basisOfRecord: PreservedSpecimen
- **Type status:** Other material. **Occurrence:** recordedBy: Limeira-de-Oliveira | et al.; individualCount: 10; sex: female; lifeStage: adult; **Location:** country: Brazil; stateProvince: Ceará; municipality: Ubajara; locality: Parque Nacional de Ubajara, Rio Cafundó, pouco acima da cachoeira; maximumElevationInMeters: 795; verbatimCoordinates: 3°50'13"S, 40°54'35"W; **Identification:** identifiedBy: Allan Paulo Moreira dos Santos; **Event:** samplingProtocol: Malaise intercept trap; verbatimEventDate: 13.ix.12; **Record Level:** institutionCode: DZRJ; basisOfRecord: PreservedSpecimen
- **Type status:** Other material. **Occurrence:** recordedBy: Limeira-de-Oliveira | et al.; individualCount: 1; sex: male; lifeStage: adult; **Location:** country: Brazil; stateProvince: Ceará; municipality: Ubajara; locality: Parque Nacional de Ubajara, Rio Cafundó, pouco acima da cachoeira; maximumElevationInMeters: 795; verbatimCoordinates: 3°50'13"S, 40°54'35"W; **Identification:** identifiedBy: Allan Paulo Moreira dos Santos; **Event:** samplingProtocol: Malaise intercept trap; verbatimEventDate: 13.xi.12; **Record Level:** institutionCode: DZRJ; basisOfRecord: PreservedSpecimen
- **Type status:** Other material. **Occurrence:** recordedBy: Rafael, J.A. | Limeira-de-Oliveira, F. | Takiya, D.M. | et al.; individualCount: 3; sex: male; lifeStage: adult; **Location:** country: Brazil; stateProvince: Ceará; municipality: Ubajara; locality: Parque Nacional de Ubajara, Trilha Samambaia, Rio Gameleira; maximumElevationInMeters: 874; verbatimCoordinates: 3°50'25"S, 40°54'19"W; **Identification:** identifiedBy: Allan Paulo Moreira dos Santos; **Event:** samplingProtocol: Malaise intercept trap; verbatimEventDate: 20.iv.12; **Record Level:** institutionCode: DZRJ; basisOfRecord: PreservedSpecimen
- **Type status:** Other material. **Occurrence:** recordedBy: Rafael, J.A. | Limeira-de-Oliveira, F. | Takiya, D.M. | et al.; individualCount: 4; sex: female; lifeStage: adult; **Location:** country: Brazil; stateProvince: Ceará; municipality: Ubajara; locality: Parque Nacional de Ubajara, Trilha Samambaia, Rio Gameleira; maximumElevationInMeters: 874; verbatimCoordinates: 3°50'25"S, 40°54'19"W; **Identification:** identifiedBy: Allan Paulo Moreira dos Santos; **Event:** samplingProtocol: Malaise intercept trap; verbatimEventDate: 20.iv.12; **Record Level:** institutionCode: DZRJ; basisOfRecord: PreservedSpecimen
- **Type status:** Other material. **Occurrence:** recordedBy: Rafael, J.A. | Limeira-de-Oliveira, F. | Takiya, D.M. | et al.; individualCount: 10; sex: male; lifeStage: adult; **Location:** country: Brazil; stateProvince: Ceará; municipality: Ubajara; locality: Parque Nacional de Ubajara, Trilha Samambaia, Rio Gameleira; maximumElevationInMeters: 874; verbatimCoordinates: 3°50'25"S, 40°54'19"W; **Identification:** identifiedBy: Allan Paulo Moreira dos Santos; **Event:** samplingProtocol: Malaise intercept trap; verbatimEventDate: 20.iv.12; **Record Level:** institutionCode: DZRJ; basisOfRecord: PreservedSpecimen
- **Type status:** Other material. **Occurrence:** recordedBy: Rafael, J.A. | Limeira-de-Oliveira, F. | Takiya, D.M. | et al.; individualCount: 10; sex: female; lifeStage: adult; **Location:** country: Brazil; stateProvince: Ceará; municipality: Ubajara; locality: Parque Nacional de Ubajara, Trilha Samambaia, Rio Gameleira; maximumElevationInMeters: 874; verbatimCoordinates: 3°50'25"S, 40°54'19"W; **Identification:** identifiedBy: Allan Paulo Moreira dos Santos; **Event:** samplingProtocol: Malaise intercept trap; verbatimEventDate: 20.iv.12; **Record Level:** institutionCode: DZRJ; basisOfRecord: PreservedSpecimen
- **Type status:** Other material. **Occurrence:** recordedBy: Rafael, J.A. | Limeira-de-Oliveira, F. | Takiya, D.M. | et al.; individualCount: 1; sex: male; lifeStage: adult; **Location:** country: Brazil; stateProvince: Ceará; municipality: Ubajara; locality: Parque Nacional de Ubajara, Trilha Araticum, Rio das Minas na altura da trilha do teleférico; maximumElevationInMeters: 420; verbatimCoordinates: 3°49'58"S, 40°53'53"W; **Identification:** identifiedBy: Allan Paulo Moreira dos Santos; **Event:** samplingProtocol: Malaise intercept trap; verbatimEventDate: 20.iv.12; **Record Level:** institutionCode: DZRJ; basisOfRecord: PreservedSpecimen
- **Type status:** Other material. **Occurrence:** recordedBy: Rafael, J.A. | Limeira-de-Oliveira, F. | Takiya, D.M. | et al.; individualCount: 2; sex: male; lifeStage: adult; **Location:** country: Brazil; stateProvince: Ceará; municipality: Ubajara; locality: Parque Nacional de Ubajara, Trilha Araticum, Rio das Minas na altura da trilha do teleférico; maximumElevationInMeters: 420; verbatimCoordinates: 3°49'58"S, 40°53'53"W; **Identification:** identifiedBy: Allan Paulo Moreira dos Santos; **Event:** samplingProtocol: Malaise intercept trap; verbatimEventDate: 20.iv.12; **Record Level:** institutionCode: DZRJ; basisOfRecord: PreservedSpecimen
- **Type status:** Other material. **Occurrence:** recordedBy: Rafael, J.A. | Limeira-de-Oliveira, F. | Takiya, D.M. | et al.; individualCount: 6; sex: male; lifeStage: adult; **Location:** country: Brazil; stateProvince: Ceará; municipality: Ubajara; locality: Parque Nacional de Ubajara, Trilha Araticum, Rio das Minas na altura da trilha do teleférico; maximumElevationInMeters: 420; verbatimCoordinates: 3°49'58"S, 40°53'53"W; **Identification:** identifiedBy: Allan Paulo Moreira dos Santos; **Event:** samplingProtocol: Malaise intercept trap; verbatimEventDate: 20.iv.12; **Record Level:** institutionCode: DZRJ; basisOfRecord: PreservedSpecimen

##### Notes

Undescribed species.

#### Alisotrichia sp. 2

##### Materials

- **Type status:** Other material. **Occurrence:** recordedBy: Rafael, J.A. | Limeira-de-Oliveira, F. | Takiya, D.M. | Santos, A.P.M. | et al.; individualCount: 1; sex: male; lifeStage: adult; **Location:** country: Brazil; stateProvince: Ceará; municipality: Ubajara; locality: Parque Nacional de Ubajara, Rio das Minas, próximo ao Portão Araticum; maximumElevationInMeters: 328; verbatimCoordinates: 3°49'32.6"S, 40°53'32.8"W; **Identification:** identifiedBy: Allan Paulo Moreira dos Santos; **Event:** samplingProtocol: Malaise intercept trap; verbatimEventDate: 17.ii.13; **Record Level:** institutionCode: DZRJ; basisOfRecord: PreservedSpecimen
- **Type status:** Other material. **Occurrence:** recordedBy: Rafael, J.A. | Limeira-de-Oliveira, F. | Takiya, D.M. | et al.; individualCount: 1; sex: male; lifeStage: adult; **Location:** country: Brazil; stateProvince: Ceará; municipality: Ubajara; locality: Parque Nacional de Ubajara, Trilha Araticum, Rio das Minas na altura da trilha do teleférico; maximumElevationInMeters: 420; verbatimCoordinates: 3°49'58"S, 40°53'53"W; **Identification:** identifiedBy: Allan Paulo Moreira dos Santos; **Event:** samplingProtocol: Malaise intercept trap; verbatimEventDate: 20.iv.12; **Record Level:** institutionCode: DZRJ; basisOfRecord: PreservedSpecimen
- **Type status:** Other material. **Occurrence:** recordedBy: Rafael, J.A. | Limeira-de-Oliveira, F. | Takiya, D.M. | et al.; individualCount: 1; sex: female; lifeStage: adult; **Location:** country: Brazil; stateProvince: Ceará; municipality: Ubajara; locality: Parque Nacional de Ubajara, Trilha Araticum, Rio das Minas na altura da trilha do teleférico; maximumElevationInMeters: 420; verbatimCoordinates: 3°49'58"S, 40°53'53"W; **Identification:** identifiedBy: Allan Paulo Moreira dos Santos; **Event:** samplingProtocol: Malaise intercept trap; verbatimEventDate: 20.iv.12; **Record Level:** institutionCode: DZRJ; basisOfRecord: PreservedSpecimen
- **Type status:** Other material. **Occurrence:** recordedBy: Rafael, J.A. | Limeira-de-Oliveira, F. | Takiya, D.M. | et al.; individualCount: 11; sex: male; lifeStage: adult; **Location:** country: Brazil; stateProvince: Ceará; municipality: Ubajara; locality: Parque Nacional de Ubajara, Trilha Araticum, Rio das Minas na altura da trilha do teleférico; maximumElevationInMeters: 420; verbatimCoordinates: 3°49'58"S, 40°53'53"W; **Identification:** identifiedBy: Allan Paulo Moreira dos Santos; **Event:** samplingProtocol: Malaise intercept trap; verbatimEventDate: 20.iv.12; **Record Level:** institutionCode: DZRJ; basisOfRecord: PreservedSpecimen
- **Type status:** Other material. **Occurrence:** recordedBy: Rafael, J.A. | Limeira-de-Oliveira, F. | Takiya, D.M. | et al.; individualCount: 18; sex: male; lifeStage: adult; **Location:** country: Brazil; stateProvince: Ceará; municipality: Ubajara; locality: Parque Nacional de Ubajara, Trilha Araticum, Rio das Minas na altura da trilha do teleférico; maximumElevationInMeters: 420; verbatimCoordinates: 3°49'58"S, 40°53'53"W; **Identification:** identifiedBy: Allan Paulo Moreira dos Santos; **Event:** samplingProtocol: Malaise intercept trap; verbatimEventDate: 20.iv.12; **Record Level:** institutionCode: DZRJ; basisOfRecord: PreservedSpecimen
- **Type status:** Other material. **Occurrence:** recordedBy: Rafael, J.A. | Limeira-de-Oliveira, F. | Takiya, D.M. | et al.; individualCount: 25; sex: male; lifeStage: adult; **Location:** country: Brazil; stateProvince: Ceará; municipality: Ubajara; locality: Parque Nacional de Ubajara, Trilha Araticum, Rio das Minas na altura da trilha do teleférico; maximumElevationInMeters: 420; verbatimCoordinates: 3°49'58"S, 40°53'53"W; **Identification:** identifiedBy: Allan Paulo Moreira dos Santos; **Event:** samplingProtocol: Malaise intercept trap; verbatimEventDate: 20.iv.12; **Record Level:** institutionCode: DZRJ; basisOfRecord: PreservedSpecimen
- **Type status:** Other material. **Occurrence:** recordedBy: Rafael, J.A. | Limeira-de-Oliveira, F. | Takiya, D.M. | et al.; individualCount: 27; sex: male; lifeStage: adult; **Location:** country: Brazil; stateProvince: Ceará; municipality: Ubajara; locality: Parque Nacional de Ubajara, Trilha Araticum, Rio das Minas na altura da trilha do teleférico; maximumElevationInMeters: 420; verbatimCoordinates: 3°49'58"S, 40°53'53"W; **Identification:** identifiedBy: Allan Paulo Moreira dos Santos; **Event:** samplingProtocol: Malaise intercept trap; verbatimEventDate: 20.iv.12; **Record Level:** institutionCode: DZRJ; basisOfRecord: PreservedSpecimen
- **Type status:** Other material. **Occurrence:** recordedBy: Rafael, J.A. | Limeira-de-Oliveira, F. | Takiya, D.M. | et al.; individualCount: 79; sex: male; lifeStage: adult; **Location:** country: Brazil; stateProvince: Ceará; municipality: Ubajara; locality: Parque Nacional de Ubajara, Trilha Araticum, Rio das Minas na altura da trilha do teleférico; maximumElevationInMeters: 420; verbatimCoordinates: 3°49'58"S, 40°53'53"W; **Identification:** identifiedBy: Allan Paulo Moreira dos Santos; **Event:** samplingProtocol: Malaise intercept trap; verbatimEventDate: 20.iv.12; **Record Level:** institutionCode: DZRJ; basisOfRecord: PreservedSpecimen
- **Type status:** Other material. **Occurrence:** recordedBy: Limeira-de-Oliveira | et al.; individualCount: 1; sex: male; lifeStage: adult; **Location:** country: Brazil; stateProvince: Ceará; municipality: Ubajara; locality: Parque Nacional de Ubajara, Trilha Araticum, Rio das Minas na altura da trilha do teleférico; maximumElevationInMeters: 420; verbatimCoordinates: 3°49'58"S, 40°53'53"W; **Identification:** identifiedBy: Allan Paulo Moreira dos Santos; **Event:** samplingProtocol: Malaise intercept trap; verbatimEventDate: 21.v.12; **Record Level:** institutionCode: DZRJ; basisOfRecord: PreservedSpecimen
- **Type status:** Other material. **Occurrence:** recordedBy: Limeira-de-Oliveira | et al.; individualCount: 7; sex: female; lifeStage: adult; **Location:** country: Brazil; stateProvince: Ceará; municipality: Ubajara; locality: Parque Nacional de Ubajara, Trilha Araticum, Rio das Minas na altura da trilha do teleférico; maximumElevationInMeters: 420; verbatimCoordinates: 3°49'58"S, 40°53'53"W; **Identification:** identifiedBy: Allan Paulo Moreira dos Santos; **Event:** samplingProtocol: Malaise intercept trap; verbatimEventDate: 21.v.12; **Record Level:** institutionCode: DZRJ; basisOfRecord: PreservedSpecimen
- **Type status:** Other material. **Occurrence:** recordedBy: Rafael, J.A. | Limeira-de-Oliveira, F. | Takiya, D.M. | et al.; individualCount: 2; sex: male; lifeStage: adult; **Location:** country: Brazil; stateProvince: Ceará; municipality: Ubajara; locality: Parque Nacional de Ubajara, Trilha Araticum, Rio das Minas na altura da trilha do teleférico; maximumElevationInMeters: 420; verbatimCoordinates: 3°49'58"S, 40°53'53"W; **Identification:** identifiedBy: Allan Paulo Moreira dos Santos; **Event:** samplingProtocol: Malaise intercept trap; verbatimEventDate: 20.iv.12; **Record Level:** institutionCode: DZRJ; basisOfRecord: PreservedSpecimen
- **Type status:** Other material. **Occurrence:** recordedBy: Rafael, J.A. | Limeira-de-Oliveira, F. | Takiya, D.M. | et al.; individualCount: 6; sex: male; lifeStage: adult; **Location:** country: Brazil; stateProvince: Ceará; municipality: Ubajara; locality: Parque Nacional de Ubajara, Trilha Araticum, Rio das Minas na altura da trilha do teleférico; maximumElevationInMeters: 420; verbatimCoordinates: 3°49'58"S, 40°53'53"W; **Identification:** identifiedBy: Allan Paulo Moreira dos Santos; **Event:** samplingProtocol: Malaise intercept trap; verbatimEventDate: 20.iv.12; **Record Level:** institutionCode: DZRJ; basisOfRecord: PreservedSpecimen

##### Notes

Undescribed species.

#### Alisotrichia sp. 3

##### Materials

- **Type status:** Other material. **Occurrence:** recordedBy: Rafael, J.A. | Limeira-de-Oliveira, F. | Takiya, D.M. | et al.; individualCount: 1; sex: male; lifeStage: adult; **Location:** country: Brazil; stateProvince: Ceará; municipality: Ubajara; locality: Parque Nacional de Ubajara, Trilha Araticum, Rio das Minas na altura da trilha do teleférico; maximumElevationInMeters: 420; verbatimCoordinates: 3°49'58"S, 40°53'53"W; **Identification:** identifiedBy: Allan Paulo Moreira dos Santos; **Event:** samplingProtocol: Malaise intercept trap; verbatimEventDate: 20.iv.12; **Record Level:** institutionCode: DZRJ; basisOfRecord: PreservedSpecimen
- **Type status:** Other material. **Occurrence:** recordedBy: Rafael, J.A. | Limeira-de-Oliveira, F. | Takiya, D.M. | et al.; individualCount: 1; sex: male; lifeStage: adult; **Location:** country: Brazil; stateProvince: Ceará; municipality: Ubajara; locality: Parque Nacional de Ubajara, Trilha Araticum, Rio das Minas na altura da trilha do teleférico; maximumElevationInMeters: 420; verbatimCoordinates: 3°49'58"S, 40°53'53"W; **Identification:** identifiedBy: Allan Paulo Moreira dos Santos; **Event:** samplingProtocol: Malaise intercept trap; verbatimEventDate: 20.iv.12; **Record Level:** institutionCode: DZRJ; basisOfRecord: PreservedSpecimen
- **Type status:** Other material. **Occurrence:** recordedBy: Rafael, J.A. | Limeira-de-Oliveira, F. | Takiya, D.M. | et al.; individualCount: 1; sex: female; lifeStage: adult; **Location:** country: Brazil; stateProvince: Ceará; municipality: Ubajara; locality: Parque Nacional de Ubajara, Trilha Araticum, Rio das Minas na altura da trilha do teleférico; maximumElevationInMeters: 420; verbatimCoordinates: 3°49'58"S, 40°53'53"W; **Identification:** identifiedBy: Allan Paulo Moreira dos Santos; **Event:** samplingProtocol: Malaise intercept trap; verbatimEventDate: 20.iv.12; **Record Level:** institutionCode: DZRJ; basisOfRecord: PreservedSpecimen
- **Type status:** Other material. **Occurrence:** recordedBy: Rafael, J.A. | Limeira-de-Oliveira, F. | Takiya, D.M. | et al.; individualCount: 11; sex: male; lifeStage: adult; **Location:** country: Brazil; stateProvince: Ceará; municipality: Ubajara; locality: Parque Nacional de Ubajara, Trilha Araticum, Rio das Minas na altura da trilha do teleférico; maximumElevationInMeters: 420; verbatimCoordinates: 3°49'58"S, 40°53'53"W; **Identification:** identifiedBy: Allan Paulo Moreira dos Santos; **Event:** samplingProtocol: Malaise intercept trap; verbatimEventDate: 20.iv.12; **Record Level:** institutionCode: DZRJ; basisOfRecord: PreservedSpecimen
- **Type status:** Other material. **Occurrence:** recordedBy: Rafael, J.A. | Limeira-de-Oliveira, F. | Takiya, D.M. | et al.; individualCount: 18; sex: male; lifeStage: adult; **Location:** country: Brazil; stateProvince: Ceará; municipality: Ubajara; locality: Parque Nacional de Ubajara, Trilha Araticum, Rio das Minas na altura da trilha do teleférico; maximumElevationInMeters: 420; verbatimCoordinates: 3°49'58"S, 40°53'53"W; **Identification:** identifiedBy: Allan Paulo Moreira dos Santos; **Event:** samplingProtocol: Malaise intercept trap; verbatimEventDate: 20.iv.12; **Record Level:** institutionCode: DZRJ; basisOfRecord: PreservedSpecimen
- **Type status:** Other material. **Occurrence:** recordedBy: Rafael, J.A. | Limeira-de-Oliveira, F. | Takiya, D.M. | et al.; individualCount: 25; sex: male; lifeStage: adult; **Location:** country: Brazil; stateProvince: Ceará; municipality: Ubajara; locality: Parque Nacional de Ubajara, Trilha Araticum, Rio das Minas na altura da trilha do teleférico; maximumElevationInMeters: 420; verbatimCoordinates: 3°49'58"S, 40°53'53"W; **Identification:** identifiedBy: Allan Paulo Moreira dos Santos; **Event:** samplingProtocol: Malaise intercept trap; verbatimEventDate: 20.iv.12; **Record Level:** institutionCode: DZRJ; basisOfRecord: PreservedSpecimen
- **Type status:** Other material. **Occurrence:** recordedBy: Rafael, J.A. | Limeira-de-Oliveira, F. | Takiya, D.M. | et al.; individualCount: 27; sex: male; lifeStage: adult; **Location:** country: Brazil; stateProvince: Ceará; municipality: Ubajara; locality: Parque Nacional de Ubajara, Trilha Araticum, Rio das Minas na altura da trilha do teleférico; maximumElevationInMeters: 420; verbatimCoordinates: 3°49'58"S, 40°53'53"W; **Identification:** identifiedBy: Allan Paulo Moreira dos Santos; **Event:** samplingProtocol: Malaise intercept trap; verbatimEventDate: 20.iv.12; **Record Level:** institutionCode: DZRJ; basisOfRecord: PreservedSpecimen
- **Type status:** Other material. **Occurrence:** recordedBy: Rafael, J.A. | Limeira-de-Oliveira, F. | Takiya, D.M. | et al.; individualCount: 79; sex: male; lifeStage: adult; **Location:** country: Brazil; stateProvince: Ceará; municipality: Ubajara; locality: Parque Nacional de Ubajara, Trilha Araticum, Rio das Minas na altura da trilha do teleférico; maximumElevationInMeters: 420; verbatimCoordinates: 3°49'58"S, 40°53'53"W; **Identification:** identifiedBy: Allan Paulo Moreira dos Santos; **Event:** samplingProtocol: Malaise intercept trap; verbatimEventDate: 20.iv.12; **Record Level:** institutionCode: DZRJ; basisOfRecord: PreservedSpecimen
- **Type status:** Other material. **Occurrence:** recordedBy: Limeira-de-Oliveira | et al.; individualCount: 1; sex: male; lifeStage: adult; **Location:** country: Brazil; stateProvince: Ceará; municipality: Ubajara; locality: Parque Nacional de Ubajara, Trilha Araticum, Rio das Minas na altura da trilha do teleférico; maximumElevationInMeters: 420; verbatimCoordinates: 3°49'58"S, 40°53'53"W; **Identification:** identifiedBy: Allan Paulo Moreira dos Santos; **Event:** samplingProtocol: Malaise intercept trap; verbatimEventDate: 21.v.12; **Record Level:** institutionCode: DZRJ; basisOfRecord: PreservedSpecimen
- **Type status:** Other material. **Occurrence:** recordedBy: Limeira-de-Oliveira | et al.; individualCount: 7; sex: female; lifeStage: adult; **Location:** country: Brazil; stateProvince: Ceará; municipality: Ubajara; locality: Parque Nacional de Ubajara, Trilha Araticum, Rio das Minas na altura da trilha do teleférico; maximumElevationInMeters: 420; verbatimCoordinates: 3°49'58"S, 40°53'53"W; **Identification:** identifiedBy: Allan Paulo Moreira dos Santos; **Event:** samplingProtocol: Malaise intercept trap; verbatimEventDate: 21.v.12; **Record Level:** institutionCode: DZRJ; basisOfRecord: PreservedSpecimen

##### Notes

Undescribed species.

#### Hydroptila

Dalman, 1918

##### Notes

Genus firstly record from CE in Souza et al. 2014a.

#### Hydroptila marighellai

Souza, Santos & Takiya, 2014

##### Materials

- **Type status:** Other material. **Occurrence:** recordedBy: Rafael, J.A. | Limeira-de-Oliveira, F. | Takiya, D.M. | et al.; individualCount: 2; sex: male; lifeStage: adult; **Location:** country: Brazil; stateProvince: Ceará; municipality: Ubajara; locality: Parque Nacional de Ubajara, Trilha Araticum, Rio das Minas na altura da trilha do teleférico; maximumElevationInMeters: 420; verbatimCoordinates: 3°49'58"S, 40°53'53"W; **Identification:** identifiedBy: Allan Paulo Moreira dos Santos; **Event:** samplingProtocol: Malaise intercept trap; verbatimEventDate: 20.iv.12; **Record Level:** institutionCode: DZRJ; basisOfRecord: PreservedSpecimen
- **Type status:** Other material. **Occurrence:** recordedBy: Rafael, J.A. | Limeira-de-Oliveira, F. | Takiya, D.M. | et al.; individualCount: 3; sex: female; lifeStage: adult; **Location:** country: Brazil; stateProvince: Ceará; municipality: Ubajara; locality: Parque Nacional de Ubajara, Trilha Araticum, Rio das Minas na altura da trilha do teleférico; maximumElevationInMeters: 420; verbatimCoordinates: 3°49'58"S, 40°53'53"W; **Identification:** identifiedBy: Allan Paulo Moreira dos Santos; **Event:** samplingProtocol: Malaise intercept trap; verbatimEventDate: 20.iv.12; **Record Level:** institutionCode: DZRJ; basisOfRecord: PreservedSpecimen
- **Type status:** Other material. **Occurrence:** recordedBy: Takiya, D.M. | Somavilla, A.; individualCount: 2; sex: male; lifeStage: adult; **Location:** country: Brazil; stateProvince: Ceará; municipality: Ubajara; locality: Parque Nacional de Ubajara, Trilha Araticum, Rio das Minas; maximumElevationInMeters: 524; verbatimCoordinates: 3°50'3"S, 40°54'18"W; **Identification:** identifiedBy: Allan Paulo Moreira dos Santos; **Event:** samplingProtocol: Pennsylvania light trap; verbatimEventDate: 22.iv.12; **Record Level:** institutionCode: DZRJ; basisOfRecord: PreservedSpecimen

##### Distribution

Brazil: CE, PE, AL.

##### Notes

Species described in Souza et al. 2014a.

#### Metrichia

Ross, 1938

##### Notes

Genus firstly recorded from CE in Santos et al. 2016a.

#### Metrichia acuminata

Santos, Takiya & Nessimian, 2016

##### Materials

- **Type status:** Other material. **Occurrence:** recordedBy: Limeira-de-Oliveira | et al.; individualCount: 2; sex: male; lifeStage: adult; **Location:** country: Brazil; stateProvince: Ceará; municipality: Ubajara; locality: Parque Nacional de Ubajara, Rio Cafundó, pouco acima da cachoeira; maximumElevationInMeters: 795; verbatimCoordinates: 3°50'13"S, 40°54'35"W; **Identification:** identifiedBy: Allan Paulo Moreira dos Santos; **Event:** samplingProtocol: Malaise intercept trap; verbatimEventDate: 13.xi.12; **Record Level:** institutionCode: DZRJ; basisOfRecord: PreservedSpecimen
- **Type status:** Other material. **Occurrence:** recordedBy: Rafael, J.A. | Limeira-de-Oliveira, F. | Takiya, D.M. | Santos, A.P.M. | et al.; individualCount: 2; sex: male; lifeStage: adult; **Location:** country: Brazil; stateProvince: Ceará; municipality: Ubajara; locality: Parque Nacional de Ubajara, Trilha Araticum, Rio das Minas; maximumElevationInMeters: 524; verbatimCoordinates: 3°50'3"S, 40°54'18"W; **Identification:** identifiedBy: Allan Paulo Moreira dos Santos; **Event:** samplingProtocol: Malaise intercept trap; verbatimEventDate: 14.ii.13; **Record Level:** institutionCode: DZRJ; basisOfRecord: PreservedSpecimen
- **Type status:** Other material. **Occurrence:** recordedBy: Takiya, D.M. | Câmara, J.T.; individualCount: 1; sex: male; lifeStage: adult; **Location:** country: Brazil; stateProvince: Ceará; municipality: Ubajara; locality: Parque Nacional de Ubajara, Trilha Samambaia, Mirante da cachoeira do Gameleira; maximumElevationInMeters: 880; verbatimCoordinates: 3°50'21"S, 40°54'23"W; **Identification:** identifiedBy: Allan Paulo Moreira dos Santos; **Event:** samplingProtocol: Pennsylvania light trap; verbatimEventDate: 23.iv.12; **Record Level:** institutionCode: DZRJ; basisOfRecord: PreservedSpecimen

##### Distribution

Brazil: CE, AL.

##### Notes

Species described in Santos et al. 2016a.

#### Metrichia vulgaris

Santos, Takiya & Nessimian, 2016

##### Materials

- **Type status:** Other material. **Occurrence:** recordedBy: Rafael, J.A. | Limeira-de-Oliveira, F. | Takiya, D.M. | et al.; individualCount: 1; sex: male; lifeStage: adult; **Location:** country: Brazil; stateProvince: Ceará; municipality: Ubajara; locality: Parque Nacional de Ubajara, Trilha Samambaia, Rio Gameleira; maximumElevationInMeters: 874; verbatimCoordinates: 3°50'25"S, 40°54'19"W; **Identification:** identifiedBy: Allan Paulo Moreira dos Santos; **Event:** samplingProtocol: Malaise intercept trap; verbatimEventDate: 20.iv.12; **Record Level:** institutionCode: DZRJ; basisOfRecord: PreservedSpecimen
- **Type status:** Other material. **Occurrence:** recordedBy: Rafael, J.A. | Limeira-de-Oliveira, F. | Takiya, D.M. | Santos, A.P.M. | et al.; individualCount: 2; sex: male; lifeStage: adult; **Location:** country: Brazil; stateProvince: Ceará; municipality: Ubajara; locality: Parque Nacional de Ubajara, Trilha Araticum, Rio das Minas; maximumElevationInMeters: 524; verbatimCoordinates: 3°50'3"S, 40°54'18"W; **Identification:** identifiedBy: Allan Paulo Moreira dos Santos; **Event:** samplingProtocol: Malaise intercept trap; verbatimEventDate: 14.ii.13; **Record Level:** institutionCode: DZRJ; basisOfRecord: PreservedSpecimen
- **Type status:** Other material. **Occurrence:** recordedBy: Takiya, D.M. | Câmara, J.T.; individualCount: 1; sex: male; lifeStage: adult; **Location:** country: Brazil; stateProvince: Ceará; municipality: Ubajara; locality: Parque Nacional de Ubajara, Trilha Samambaia, Mirante da cachoeira do Gameleira; maximumElevationInMeters: 880; verbatimCoordinates: 3°50'21"S, 40°54'23"W; **Identification:** identifiedBy: Allan Paulo Moreira dos Santos; **Event:** samplingProtocol: Pennsylvania light trap; verbatimEventDate: 23.iv.12; **Record Level:** institutionCode: DZRJ; basisOfRecord: PreservedSpecimen

##### Distribution

Brazil: CE, GO, MG, RJ.

##### Notes

Species described in Santos et al. 2016a.

#### Metrichia rafaeli

Santos, Takiya & Nessimian, 2016

##### Materials

- **Type status:** Other material. **Occurrence:** recordedBy: Limeira-de-Oliveira | et al.; individualCount: 1; sex: male; lifeStage: adult; **Location:** country: Brazil; stateProvince: Ceará; municipality: Ubajara; locality: Parque Nacional de Ubajara, Rio Cafundó, pouco acima da cachoeira; maximumElevationInMeters: 795; verbatimCoordinates: 3°50'13"S, 40°54'35"W; **Identification:** identifiedBy: Allan Paulo Moreira dos Santos; **Event:** samplingProtocol: Malaise intercept trap; verbatimEventDate: 13.ix.12; **Record Level:** institutionCode: DZRJ; basisOfRecord: PreservedSpecimen
- **Type status:** Other material. **Occurrence:** recordedBy: Rafael, J.A. | Limeira-de-Oliveira, F. | Takiya, D.M. | Santos, A.P.M. | et al.; individualCount: 1; sex: male; lifeStage: adult; **Location:** country: Brazil; stateProvince: Ceará; municipality: Ubajara; locality: Parque Nacional de Ubajara, Trilha Araticum, Rio das Minas na altura da trilha do teleférico; maximumElevationInMeters: 420; verbatimCoordinates: 3°49'58"S, 40°53'53"W; **Identification:** identifiedBy: Allan Paulo Moreira dos Santos; **Event:** samplingProtocol: Malaise intercept trap; verbatimEventDate: 14.ii.13; **Record Level:** institutionCode: DZRJ; basisOfRecord: PreservedSpecimen
- **Type status:** Other material. **Occurrence:** recordedBy: Santos, A.P.M. | Takiya, D.M.; individualCount: 1; sex: male; lifeStage: adult; **Location:** country: Brazil; stateProvince: Ceará; municipality: Ubajara; locality: Parque Nacional de Ubajara, Cachoeira do Cafundó; maximumElevationInMeters: 783; verbatimCoordinates: 3°50'12"S, 40°54'35"W; **Identification:** identifiedBy: Allan Paulo Moreira dos Santos; **Event:** samplingProtocol: Pennsylvania light trap; verbatimEventDate: 15.ii.13; **Record Level:** institutionCode: DZRJ; basisOfRecord: PreservedSpecimen
- **Type status:** Other material. **Occurrence:** recordedBy: Rafael, J.A. | Limeira-de-Oliveira, F. | Takiya, D.M. | Santos, A.P.M. | et al.; individualCount: 1; sex: male; lifeStage: adult; **Location:** country: Brazil; stateProvince: Ceará; municipality: Ubajara; locality: Parque Nacional de Ubajara, Trilha Samambaia, Rio Gameleira; maximumElevationInMeters: 874; verbatimCoordinates: 3°50'25"S, 40°54'19"W; **Identification:** identifiedBy: Allan Paulo Moreira dos Santos; **Event:** samplingProtocol: Malaise intercept trap; verbatimEventDate: 17.ii.13; **Record Level:** institutionCode: DZRJ; basisOfRecord: PreservedSpecimen
- **Type status:** Other material. **Occurrence:** recordedBy: Rafael, J.A. | Limeira-de-Oliveira, F. | Takiya, D.M. | Santos, A.P.M. | et al.; individualCount: 2; sex: male; lifeStage: adult; **Location:** country: Brazil; stateProvince: Ceará; municipality: Ubajara; locality: Parque Nacional de Ubajara, Trilha Araticum, Rio das Minas; maximumElevationInMeters: 524; verbatimCoordinates: 3°50'3"S, 40°54'18"W; **Identification:** identifiedBy: Allan Paulo Moreira dos Santos; **Event:** samplingProtocol: Malaise intercept trap; verbatimEventDate: 17.ii.13; **Record Level:** institutionCode: DZRJ; basisOfRecord: PreservedSpecimen
- **Type status:** Other material. **Occurrence:** recordedBy: Rafael, J.A. | Limeira-de-Oliveira, F. | Takiya, D.M. | et al.; individualCount: 1; sex: male; lifeStage: adult; **Location:** country: Brazil; stateProvince: Ceará; municipality: Ubajara; locality: Parque Nacional de Ubajara, Trilha Araticum, Rio das Minas na altura da trilha do teleférico; maximumElevationInMeters: 420; verbatimCoordinates: 3°49'58"S, 40°53'53"W; **Identification:** identifiedBy: Allan Paulo Moreira dos Santos; **Event:** samplingProtocol: Malaise intercept trap; verbatimEventDate: 20.iv.12; **Record Level:** institutionCode: DZRJ; basisOfRecord: PreservedSpecimen

##### Distribution

Brazil: CE.

##### Notes

Species described in Santos et al. 2016a.

#### Metrichia ubajara

Santos, Takiya & Nessimian, 2016

##### Materials

- **Type status:** Other material. **Occurrence:** recordedBy: Limeira-de-Oliveira | et al.; individualCount: 2; sex: male; lifeStage: adult; **Location:** country: Brazil; stateProvince: Ceará; municipality: Ubajara; locality: Parque Nacional de Ubajara, Rio Cafundó, pouco acima da cachoeira; maximumElevationInMeters: 795; verbatimCoordinates: 3°50'13"S, 40°54'35"W; **Identification:** identifiedBy: Allan Paulo Moreira dos Santos; **Event:** samplingProtocol: Malaise intercept trap; verbatimEventDate: 13.xi.12; **Record Level:** institutionCode: DZRJ; basisOfRecord: PreservedSpecimen
- **Type status:** Other material. **Occurrence:** recordedBy: Limeira-de-Oliveira | et al.; individualCount: 10; sex: male; lifeStage: adult; **Location:** country: Brazil; stateProvince: Ceará; municipality: Ubajara; locality: Parque Nacional de Ubajara, Rio Cafundó, pouco acima da cachoeira; maximumElevationInMeters: 795; verbatimCoordinates: 3°50'13"S, 40°54'35"W; **Identification:** identifiedBy: Allan Paulo Moreira dos Santos; **Event:** samplingProtocol: Malaise intercept trap; verbatimEventDate: 13.xi.12; **Record Level:** institutionCode: DZRJ; basisOfRecord: PreservedSpecimen
- **Type status:** Other material. **Occurrence:** recordedBy: Limeira-de-Oliveira | et al.; individualCount: 26; sex: male; lifeStage: adult; **Location:** country: Brazil; stateProvince: Ceará; municipality: Ubajara; locality: Parque Nacional de Ubajara, Rio Cafundó, pouco acima da cachoeira; maximumElevationInMeters: 795; verbatimCoordinates: 3°50'13"S, 40°54'35"W; **Identification:** identifiedBy: Allan Paulo Moreira dos Santos; **Event:** samplingProtocol: Malaise intercept trap; verbatimEventDate: 13.xi.12; **Record Level:** institutionCode: DZRJ; basisOfRecord: PreservedSpecimen
- **Type status:** Other material. **Occurrence:** recordedBy: Rafael, J.A. | Limeira-de-Oliveira, F. | Takiya, D.M. | Santos, A.P.M. | et al.; individualCount: 7; sex: male; lifeStage: adult; **Location:** country: Brazil; stateProvince: Ceará; municipality: Ubajara; locality: Parque Nacional de Ubajara, Trilha Araticum, Rio das Minas; maximumElevationInMeters: 524; verbatimCoordinates: 3°50'3"S, 40°54'18"W; **Identification:** identifiedBy: Allan Paulo Moreira dos Santos; **Event:** samplingProtocol: Malaise intercept trap; verbatimEventDate: 14.ii.13; **Record Level:** institutionCode: DZRJ; basisOfRecord: PreservedSpecimen
- **Type status:** Other material. **Occurrence:** recordedBy: Rafael, J.A. | Limeira-de-Oliveira, F. | Takiya, D.M. | Santos, A.P.M. | et al.; individualCount: 9; sex: male; lifeStage: adult; **Location:** country: Brazil; stateProvince: Ceará; municipality: Ubajara; locality: Parque Nacional de Ubajara, Trilha Araticum, Rio das Minas na altura da trilha do teleférico; maximumElevationInMeters: 420; verbatimCoordinates: 3°49'58"S, 40°53'53"W; **Identification:** identifiedBy: Allan Paulo Moreira dos Santos; **Event:** samplingProtocol: Malaise intercept trap; verbatimEventDate: 14.ii.13; **Record Level:** institutionCode: DZRJ; basisOfRecord: PreservedSpecimen
- **Type status:** Other material. **Occurrence:** recordedBy: Rafael, J.A. | Limeira-de-Oliveira, F. | Takiya, D.M. | Santos, A.P.M. | et al.; individualCount: 2; sex: male; lifeStage: adult; **Location:** country: Brazil; stateProvince: Ceará; municipality: Ubajara; locality: Parque Nacional de Ubajara, Rio das Minas, próximo ao Portão Araticum; maximumElevationInMeters: 328; verbatimCoordinates: 3°49'32.6"S, 40°53'32.8"W; **Identification:** identifiedBy: Allan Paulo Moreira dos Santos; **Event:** samplingProtocol: Malaise intercept trap; verbatimEventDate: 14.ii.13; **Record Level:** institutionCode: DZRJ; basisOfRecord: PreservedSpecimen
- **Type status:** Other material. **Occurrence:** recordedBy: Limeira-de-Oliveira | et al.; individualCount: 9; sex: male; lifeStage: adult; **Location:** country: Brazil; stateProvince: Ceará; municipality: Ubajara; locality: Parque Nacional de Ubajara, Rio Cafundó, pouco acima da cachoeira; maximumElevationInMeters: 795; verbatimCoordinates: 3°50'13"S, 40°54'35"W; **Identification:** identifiedBy: Allan Paulo Moreira dos Santos; **Event:** samplingProtocol: Malaise intercept trap; verbatimEventDate: 18.xi.12; **Record Level:** institutionCode: DZRJ; basisOfRecord: PreservedSpecimen
- **Type status:** Other material. **Occurrence:** recordedBy: Rafael, J.A. | Limeira-de-Oliveira, F. | Takiya, D.M. | et al.; individualCount: 1; sex: male; lifeStage: adult; **Location:** country: Brazil; stateProvince: Ceará; municipality: Ubajara; locality: Parque Nacional de Ubajara, Trilha Araticum, Rio das Minas na altura da trilha do teleférico; maximumElevationInMeters: 420; verbatimCoordinates: 3°49'58"S, 40°53'53"W; **Identification:** identifiedBy: Allan Paulo Moreira dos Santos; **Event:** samplingProtocol: Malaise intercept trap; verbatimEventDate: 20.iv.12; **Record Level:** institutionCode: DZRJ; basisOfRecord: PreservedSpecimen
- **Type status:** Other material. **Occurrence:** recordedBy: Rafael, J.A. | Limeira-de-Oliveira, F. | Takiya, D.M. | et al.; individualCount: 1; sex: male; lifeStage: adult; **Location:** country: Brazil; stateProvince: Ceará; municipality: Ubajara; locality: Parque Nacional de Ubajara, Trilha Araticum, Rio das Minas na altura da trilha do teleférico; maximumElevationInMeters: 420; verbatimCoordinates: 3°49'58"S, 40°53'53"W; **Identification:** identifiedBy: Allan Paulo Moreira dos Santos; **Event:** samplingProtocol: Malaise intercept trap; verbatimEventDate: 20.iv.12; **Record Level:** institutionCode: DZRJ; basisOfRecord: PreservedSpecimen
- **Type status:** Other material. **Occurrence:** recordedBy: Rafael, J.A. | Limeira-de-Oliveira, F. | Takiya, D.M. | et al.; individualCount: 1; sex: male; lifeStage: adult; **Location:** country: Brazil; stateProvince: Ceará; municipality: Ubajara; locality: Parque Nacional de Ubajara, Trilha Araticum, Rio das Minas na altura da trilha do teleférico; maximumElevationInMeters: 420; verbatimCoordinates: 3°49'58"S, 40°53'53"W; **Identification:** identifiedBy: Allan Paulo Moreira dos Santos; **Event:** samplingProtocol: Malaise intercept trap; verbatimEventDate: 20.iv.12; **Record Level:** institutionCode: DZRJ; basisOfRecord: PreservedSpecimen

##### Distribution

Brazil: CE.

##### Notes

Species described in Santos et al. 2016a.

#### Neotrichia

Morton, 1905

##### Notes

New genus record for CE.

#### Neotrichia sp. 2*

##### Materials

- **Type status:** Other material. **Occurrence:** recordedBy: Santos, A.P.M. | Takiya, D.M.; individualCount: 1; sex: male; lifeStage: adult; **Location:** country: Brazil; stateProvince: Ceará; municipality: Ubajara; locality: Parque Nacional de Ubajara, Trilha Samambaia, Rio Gameleira; maximumElevationInMeters: 874; verbatimCoordinates: 3°50'25"S, 40°54'19"W; **Identification:** identifiedBy: Allan Paulo Moreira dos Santos; **Event:** samplingProtocol: Pennsylvania light trap; verbatimEventDate: 13.ii.13; **Record Level:** institutionCode: DZRJ; basisOfRecord: PreservedSpecimen
- **Type status:** Other material. **Occurrence:** recordedBy: Santos, A.P.M. | Takiya, D.M.; individualCount: 1; sex: male; lifeStage: adult; **Location:** country: Brazil; stateProvince: Ceará; municipality: Ubajara; locality: Parque Nacional de Ubajara, Trilha Araticum, Rio da Minas abaixo do teleférico; maximumElevationInMeters: 395; verbatimCoordinates: 3°49'43.3"S, 40°53'51.5"W; **Identification:** identifiedBy: Allan Paulo Moreira dos Santos; **Event:** samplingProtocol: Pennsylvania light trap; verbatimEventDate: 14.ii.13; **Record Level:** institutionCode: DZRJ; basisOfRecord: PreservedSpecimen
- **Type status:** Other material. **Occurrence:** recordedBy: Rafael, J.A. | Limeira-de-Oliveira, F. | Takiya, D.M. | et al.; individualCount: 1; sex: male; lifeStage: adult; **Location:** country: Brazil; stateProvince: Ceará; municipality: Ubajara; locality: Parque Nacional de Ubajara, Trilha Araticum, Rio das Minas na altura da trilha do teleférico; maximumElevationInMeters: 420; verbatimCoordinates: 3°49'58"S, 40°53'53"W; **Identification:** identifiedBy: Allan Paulo Moreira dos Santos; **Event:** samplingProtocol: Malaise intercept trap; verbatimEventDate: 20.iv.12; **Record Level:** institutionCode: DZRJ; basisOfRecord: PreservedSpecimen

#### Neotrichia sp. 5

##### Materials

- **Type status:** Other material. **Occurrence:** recordedBy: Rafael, J.A. | Limeira-de-Oliveira, F. | Takiya, D.M. | Santos, A.P.M. | et al.; individualCount: 1; sex: male; lifeStage: adult; **Location:** country: Brazil; stateProvince: Ceará; municipality: Ubajara; locality: Parque Nacional de Ubajara, Trilha Araticum, Rio das Minas na altura da trilha do teleférico; maximumElevationInMeters: 420; verbatimCoordinates: 3°49'58"S, 40°53'53"W; **Identification:** identifiedBy: Allan Paulo Moreira dos Santos; **Event:** samplingProtocol: Malaise intercept trap; verbatimEventDate: 14.ii.13; **Record Level:** institutionCode: DZRJ; basisOfRecord: PreservedSpecimen
- **Type status:** Other material. **Occurrence:** recordedBy: Santos, A.P.M. | Takiya, D.M.; individualCount: 1; sex: male; lifeStage: adult; **Location:** country: Brazil; stateProvince: Ceará; municipality: Ubajara; locality: Parque Nacional de Ubajara, Trilha Araticum, Rio da Minas abaixo do teleférico; maximumElevationInMeters: 395; verbatimCoordinates: 3°49'43.3"S, 40°53'51.5"W; **Identification:** identifiedBy: Allan Paulo Moreira dos Santos; **Event:** samplingProtocol: Pennsylvania light trap; verbatimEventDate: 14.ii.13; **Record Level:** institutionCode: DZRJ; basisOfRecord: PreservedSpecimen
- **Type status:** Other material. **Occurrence:** recordedBy: Rafael, J.A. | Limeira-de-Oliveira, F. | Takiya, D.M. | et al.; individualCount: 1; sex: male; lifeStage: adult; **Location:** country: Brazil; stateProvince: Ceará; municipality: Ubajara; locality: Parque Nacional de Ubajara, Trilha Araticum, Rio das Minas na altura da trilha do teleférico; maximumElevationInMeters: 420; verbatimCoordinates: 3°49'58"S, 40°53'53"W; **Identification:** identifiedBy: Allan Paulo Moreira dos Santos; **Event:** samplingProtocol: Malaise intercept trap; verbatimEventDate: 20.iv.12; **Record Level:** institutionCode: DZRJ; basisOfRecord: PreservedSpecimen

#### Ochrotrichia

Mosely, 1934

##### Notes

Genus firstly recorded from CE in Souza et al. 2014a​.

#### Ochrotrichia caatinga

Souza, Santos & Takiya, 2014

##### Materials

- **Type status:** Other material. **Occurrence:** recordedBy: Rafael, J.A. | Limeira-de-Oliveira, F. | Takiya, D.M. | Santos, A.P.M. | et al.; individualCount: 1; sex: male; lifeStage: adult; **Location:** country: Brazil; stateProvince: Ceará; municipality: Ubajara; locality: Parque Nacional de Ubajara, Trilha Araticum, Rio das Minas; maximumElevationInMeters: 524; verbatimCoordinates: 3°50'3"S, 40°54'18"W; **Identification:** identifiedBy: Allan Paulo Moreira dos Santos; **Event:** samplingProtocol: Malaise intercept trap; verbatimEventDate: 14.ii.13; **Record Level:** institutionCode: DZRJ; basisOfRecord: PreservedSpecimen
- **Type status:** Other material. **Occurrence:** recordedBy: Rafael, J.A. | Limeira-de-Oliveira, F. | Takiya, D.M. | Santos, A.P.M. | et al.; individualCount: 1; sex: male; lifeStage: adult; **Location:** country: Brazil; stateProvince: Ceará; municipality: Ubajara; locality: Parque Nacional de Ubajara, Trilha Samambaia, Rio Gameleira; maximumElevationInMeters: 874; verbatimCoordinates: 3°50'25"S, 40°54'19"W; **Identification:** identifiedBy: Allan Paulo Moreira dos Santos; **Event:** samplingProtocol: Malaise intercept trap; verbatimEventDate: 17.ii.13; **Record Level:** institutionCode: DZRJ; basisOfRecord: PreservedSpecimen
- **Type status:** Other material. **Occurrence:** recordedBy: Rafael, J.A. | Limeira-de-Oliveira, F. | Takiya, D.M. | Santos, A.P.M. | et al.; individualCount: 1; sex: male; lifeStage: adult; **Location:** country: Brazil; stateProvince: Ceará; municipality: Ubajara; locality: Parque Nacional de Ubajara, Trilha Araticum, Rio das Minas; maximumElevationInMeters: 524; verbatimCoordinates: 3°50'3"S, 40°54'18"W; **Identification:** identifiedBy: Allan Paulo Moreira dos Santos; **Event:** samplingProtocol: Malaise intercept trap; verbatimEventDate: 17.ii.13; **Record Level:** institutionCode: DZRJ; basisOfRecord: PreservedSpecimen
- **Type status:** Other material. **Occurrence:** recordedBy: Rafael, J.A. | Limeira-de-Oliveira, F. | Takiya, D.M. | et al.; individualCount: 3; sex: male; lifeStage: adult; **Location:** country: Brazil; stateProvince: Ceará; municipality: Ubajara; locality: Parque Nacional de Ubajara, Trilha Samambaia, Rio Gameleira; maximumElevationInMeters: 874; verbatimCoordinates: 3°50'25"S, 40°54'19"W; **Identification:** identifiedBy: Allan Paulo Moreira dos Santos; **Event:** samplingProtocol: Malaise intercept trap; verbatimEventDate: 20.iv.12; **Record Level:** institutionCode: DZRJ; basisOfRecord: PreservedSpecimen
- **Type status:** Other material. **Occurrence:** recordedBy: Rafael, J.A. | Limeira-de-Oliveira, F. | Takiya, D.M. | et al.; individualCount: 1; sex: male; lifeStage: adult; **Location:** country: Brazil; stateProvince: Ceará; municipality: Ubajara; locality: Parque Nacional de Ubajara, Rio Cafundó, pouco acima da cachoeira; maximumElevationInMeters: 795; verbatimCoordinates: 3°50'13"S, 40°54'35"W; **Identification:** identifiedBy: Allan Paulo Moreira dos Santos; **Event:** samplingProtocol: Malaise intercept trap; verbatimEventDate: 21.iv.12; **Record Level:** institutionCode: DZRJ; basisOfRecord: PreservedSpecimen

##### Distribution

Brazil: CE.

##### Notes

Species described in Souza et al. 2014a.

#### Ochrotrichia limeirai

Souza, Santos & Takiya, 2014

##### Materials

- **Type status:** Other material. **Occurrence:** recordedBy: Limeira-de-Oliveira | et al.; individualCount: 1; sex: male; lifeStage: adult; **Location:** country: Brazil; stateProvince: Ceará; municipality: Ubajara; locality: Parque Nacional de Ubajara, Rio Cafundó, pouco acima da cachoeira; maximumElevationInMeters: 795; verbatimCoordinates: 3°50'13"S, 40°54'35"W; **Identification:** identifiedBy: Allan Paulo Moreira dos Santos; **Event:** samplingProtocol: Malaise intercept trap; verbatimEventDate: 13.xi.12; **Record Level:** institutionCode: DZRJ; basisOfRecord: PreservedSpecimen
- **Type status:** Other material. **Occurrence:** recordedBy: Rafael, J.A. | Limeira-de-Oliveira, F. | Takiya, D.M. | Santos, A.P.M. | et al.; individualCount: 1; sex: male; lifeStage: adult; **Location:** country: Brazil; stateProvince: Ceará; municipality: Ubajara; locality: Parque Nacional de Ubajara, Trilha Samambaia, Rio Gameleira; maximumElevationInMeters: 874; verbatimCoordinates: 3°50'25"S, 40°54'19"W; **Identification:** identifiedBy: Allan Paulo Moreira dos Santos; **Event:** samplingProtocol: Malaise intercept trap; verbatimEventDate: 17.ii.13; **Record Level:** institutionCode: DZRJ; basisOfRecord: PreservedSpecimen
- **Type status:** Other material. **Occurrence:** recordedBy: Rafael, J.A. | Limeira-de-Oliveira, F. | Takiya, D.M. | Santos, A.P.M. | et al.; individualCount: 1; sex: male; lifeStage: adult; **Location:** country: Brazil; stateProvince: Ceará; municipality: Ubajara; locality: Parque Nacional de Ubajara, Trilha Araticum, Rio das Minas; maximumElevationInMeters: 524; verbatimCoordinates: 3°50'3"S, 40°54'18"W; **Identification:** identifiedBy: Allan Paulo Moreira dos Santos; **Event:** samplingProtocol: Malaise intercept trap; verbatimEventDate: 17.ii.13; **Record Level:** institutionCode: DZRJ; basisOfRecord: PreservedSpecimen
- **Type status:** Other material. **Occurrence:** recordedBy: Rafael, J.A. | Limeira-de-Oliveira, F. | Takiya, D.M. | et al.; individualCount: 3; sex: male; lifeStage: adult; **Location:** country: Brazil; stateProvince: Ceará; municipality: Ubajara; locality: Parque Nacional de Ubajara, Trilha Samambaia, Rio Gameleira; maximumElevationInMeters: 874; verbatimCoordinates: 3°50'25"S, 40°54'19"W; **Identification:** identifiedBy: Allan Paulo Moreira dos Santos; **Event:** samplingProtocol: Malaise intercept trap; verbatimEventDate: 20.iv.12; **Record Level:** institutionCode: DZRJ; basisOfRecord: PreservedSpecimen
- **Type status:** Other material. **Occurrence:** recordedBy: Rafael, J.A. | Limeira-de-Oliveira, F. | Takiya, D.M. | et al.; individualCount: 1; sex: male; lifeStage: adult; **Location:** country: Brazil; stateProvince: Ceará; municipality: Ubajara; locality: Parque Nacional de Ubajara, Rio Cafundó, pouco acima da cachoeira; maximumElevationInMeters: 795; verbatimCoordinates: 3°50'13"S, 40°54'35"W; **Identification:** identifiedBy: Allan Paulo Moreira dos Santos; **Event:** samplingProtocol: Malaise intercept trap; verbatimEventDate: 21.iv.12; **Record Level:** institutionCode: DZRJ; basisOfRecord: PreservedSpecimen

##### Distribution

Brazil: CE.

##### Notes

Species described in Souza et al. 2014a.

#### Ochrotrichia patulosa

(Wasmund & Holzenthal, 2007)

##### Materials

- **Type status:** Other material. **Occurrence:** recordedBy: Limeira-de-Oliveira | et al.; individualCount: 1; sex: male; lifeStage: adult; **Location:** country: Brazil; stateProvince: Ceará; municipality: Ubajara; locality: Parque Nacional de Ubajara, Rio Cafundó, pouco acima da cachoeira; maximumElevationInMeters: 795; verbatimCoordinates: 3°50'13"S, 40°54'35"W; **Identification:** identifiedBy: Wagner Rafael Maciel de Souza; **Event:** samplingProtocol: Malaise intercept trap; verbatimEventDate: 13.xi.12; **Record Level:** institutionCode: DZRJ; basisOfRecord: PreservedSpecimen

##### Distribution

Brazil: CE, RJ.

##### Notes

Species firstly recorded from CE in Souza et al. 2014a.

#### Oxyethira

Eaton, 1873

##### Notes

New genus record for CE.

#### Oxyethira parce

(Edwards & Arnold, 1961)

##### Materials

- **Type status:** Other material. **Occurrence:** recordedBy: Rafael, J.A. | Limeira-de-Oliveira, F. | Takiya, D.M. | Santos, A.P.M. | et al.; individualCount: 2; sex: male; lifeStage: adult; **Location:** country: Brazil; stateProvince: Ceará; municipality: Ubajara; locality: Parque Nacional de Ubajara, Trilha Araticum, Rio das Minas; maximumElevationInMeters: 524; verbatimCoordinates: 3°50'3"S, 40°54'18"W; **Identification:** identifiedBy: Allan Paulo Moreira dos Santos; **Event:** samplingProtocol: Malaise intercept trap; verbatimEventDate: 14.ii.13; **Record Level:** institutionCode: DZRJ; basisOfRecord: PreservedSpecimen
- **Type status:** Other material. **Occurrence:** recordedBy: Rafael, J.A. | Limeira-de-Oliveira, F. | Takiya, D.M. | Santos, A.P.M. | et al.; individualCount: 1; sex: male; lifeStage: adult; **Location:** country: Brazil; stateProvince: Ceará; municipality: Ubajara; locality: Parque Nacional de Ubajara, Trilha Araticum, Rio das Minas; maximumElevationInMeters: 524; verbatimCoordinates: 3°50'3"S, 40°54'18"W; **Identification:** identifiedBy: Allan Paulo Moreira dos Santos; **Event:** samplingProtocol: Malaise intercept trap; verbatimEventDate: 17.ii.13; **Record Level:** institutionCode: DZRJ; basisOfRecord: PreservedSpecimen
- **Type status:** Other material. **Occurrence:** recordedBy: Takiya, D.M. | Somavilla, A.; individualCount: 2; sex: male; lifeStage: adult; **Location:** country: Brazil; stateProvince: Ceará; municipality: Ubajara; locality: Parque Nacional de Ubajara, Trilha Araticum, Rio das Minas; maximumElevationInMeters: 524; verbatimCoordinates: 3°50'3"S, 40°54'18"W; **Identification:** identifiedBy: Allan Paulo Moreira dos Santos; **Event:** samplingProtocol: Pennsylvania light trap; verbatimEventDate: 22.iv.12; **Record Level:** institutionCode: DZRJ; basisOfRecord: PreservedSpecimen

##### Distribution

USA, Mexico. Costa Rica. Panama. Trinidad and Tobago. Colombia. Venezuela. Guyana. Brazil: CE!, MG. Ecuador. Peru. Bolivia. Argentina.

##### Notes

New species record for Northeastern Brazil.

#### Oxyethira tica

Harris & Holzenthal, 1992

##### Materials

- **Type status:** Other material. **Occurrence:** recordedBy: Rafael, J.A. | Limeira-de-Oliveira, F. | Takiya, D.M. | Santos, A.P.M. | et al.; individualCount: 1; sex: male; lifeStage: adult; **Location:** country: Brazil; stateProvince: Ceará; municipality: Ubajara; locality: Parque Nacional de Ubajara, Trilha Araticum, Rio das Minas; maximumElevationInMeters: 524; verbatimCoordinates: 3°50'3"S, 40°54'18"W; **Identification:** identifiedBy: Allan Paulo Moreira dos Santos; **Event:** samplingProtocol: Malaise intercept trap; verbatimEventDate: 14.ii.13; **Record Level:** institutionCode: DZRJ; basisOfRecord: PreservedSpecimen
- **Type status:** Other material. **Occurrence:** recordedBy: Rafael, J.A. | Limeira-de-Oliveira, F. | Takiya, D.M. | Santos, A.P.M. | et al.; individualCount: 1; sex: male; lifeStage: adult; **Location:** country: Brazil; stateProvince: Ceará; municipality: Ubajara; locality: Parque Nacional de Ubajara, Trilha Araticum, Rio das Minas na altura da trilha do teleférico; maximumElevationInMeters: 420; verbatimCoordinates: 3°49'58"S, 40°53'53"W; **Identification:** identifiedBy: Allan Paulo Moreira dos Santos; **Event:** samplingProtocol: Malaise intercept trap; verbatimEventDate: 14.ii.13; **Record Level:** institutionCode: DZRJ; basisOfRecord: PreservedSpecimen
- **Type status:** Other material. **Occurrence:** recordedBy: Rafael, J.A. | Limeira-de-Oliveira, F. | Takiya, D.M. | Santos, A.P.M. | et al.; individualCount: 1; sex: female; lifeStage: adult; **Location:** country: Brazil; stateProvince: Ceará; municipality: Ubajara; locality: Parque Nacional de Ubajara, Trilha Araticum, Rio das Minas na altura da trilha do teleférico; maximumElevationInMeters: 420; verbatimCoordinates: 3°49'58"S, 40°53'53"W; **Identification:** identifiedBy: Allan Paulo Moreira dos Santos; **Event:** samplingProtocol: Malaise intercept trap; verbatimEventDate: 14.ii.13; **Record Level:** institutionCode: DZRJ; basisOfRecord: PreservedSpecimen
- **Type status:** Other material. **Occurrence:** recordedBy: Rafael, J.A. | Limeira-de-Oliveira, F. | Takiya, D.M. | Santos, A.P.M. | et al.; individualCount: 1; sex: male; lifeStage: adult; **Location:** country: Brazil; stateProvince: Ceará; municipality: Ubajara; locality: Parque Nacional de Ubajara, Rio das Minas, próximo ao Portão Araticum; maximumElevationInMeters: 328; verbatimCoordinates: 3°49'32.6"S, 40°53'32.8"W; **Identification:** identifiedBy: Allan Paulo Moreira dos Santos; **Event:** samplingProtocol: Malaise intercept trap; verbatimEventDate: 17.ii.13; **Record Level:** institutionCode: DZRJ; basisOfRecord: PreservedSpecimen
- **Type status:** Other material. **Occurrence:** recordedBy: Takiya, D.M. | Somavilla, A.; individualCount: 1; sex: male; lifeStage: adult; **Location:** country: Brazil; stateProvince: Ceará; municipality: Ubajara; locality: Parque Nacional de Ubajara, Trilha Araticum, Rio das Minas; maximumElevationInMeters: 524; verbatimCoordinates: 3°50'3"S, 40°54'18"W; **Identification:** identifiedBy: Allan Paulo Moreira dos Santos; **Event:** samplingProtocol: Pennsylvania light trap; verbatimEventDate: 22.iv.12; **Record Level:** institutionCode: DZRJ; basisOfRecord: PreservedSpecimen
- **Type status:** Other material. **Occurrence:** recordedBy: Takiya, D.M. | Rafael, J.A.; individualCount: 1; sex: male; lifeStage: adult; **Location:** country: Brazil; stateProvince: Ceará; municipality: Ubajara; locality: Parque Nacional de Ubajara, Rio Cafundó, pouco acima da cachoeira; maximumElevationInMeters: 795; verbatimCoordinates: 3°50'13"S, 40°54'35"W; **Identification:** identifiedBy: Allan Paulo Moreira dos Santos; **Event:** samplingProtocol: Pennsylvania light trap; verbatimEventDate: 24.iv.12; **Record Level:** institutionCode: DZRJ; basisOfRecord: PreservedSpecimen

##### Distribution

Mexico. Honduras. Costa Rica. Panama. Guadeloupe. Dominica. Santa Lucia. Saint Vicent and the Grenadines. Granada. Trinidad and Tobago. Venezuela. Brazil: AM, PI!, CE!, MG, RJ. Ecuador.

##### Notes

New species record for Northeastern Brazil.

#### Leptoceridae

#### Atanatolica

Mosely, 1936

##### Notes

Genus firstly recorded from CE in Henriques-Oliveira and Santos 2014.

#### Atanatolica nordestina

Henriques-Oliveira & Santos, 2014

##### Materials

- **Type status:** Other material. **Occurrence:** recordedBy: Limeira-de-Oliveira | et al.; individualCount: 2; sex: male; lifeStage: adult; **Location:** country: Brazil; stateProvince: Ceará; municipality: Ubajara; locality: Parque Nacional de Ubajara, Rio Cafundó, pouco acima da cachoeira; maximumElevationInMeters: 795; verbatimCoordinates: 3°50'13"S, 40°54'35"W; **Identification:** identifiedBy: Ana Lucia Henriques Oliveira | Allan Paulo Moreira dos Santos; **Event:** samplingProtocol: Malaise intercept trap; verbatimEventDate: 1.ii.13; **Record Level:** institutionCode: DZRJ; basisOfRecord: PreservedSpecimen
- **Type status:** Other material. **Occurrence:** recordedBy: Limeira-de-Oliveira | et al.; individualCount: 1; sex: female; lifeStage: adult; **Location:** country: Brazil; stateProvince: Ceará; municipality: Ubajara; locality: Parque Nacional de Ubajara, Rio Cafundó, pouco acima da cachoeira; maximumElevationInMeters: 795; verbatimCoordinates: 3°50'13"S, 40°54'35"W; **Identification:** identifiedBy: Ana Lucia Henriques Oliveira | Allan Paulo Moreira dos Santos; **Event:** samplingProtocol: Malaise intercept trap; verbatimEventDate: 1.ii.13; **Record Level:** institutionCode: DZRJ; basisOfRecord: PreservedSpecimen
- **Type status:** Other material. **Occurrence:** recordedBy: Limeira-de-Oliveira | et al.; individualCount: 2; sex: female; lifeStage: adult; **Location:** country: Brazil; stateProvince: Ceará; municipality: Ubajara; locality: Parque Nacional de Ubajara, Rio Cafundó, pouco acima da cachoeira; maximumElevationInMeters: 795; verbatimCoordinates: 3°50'13"S, 40°54'35"W; **Identification:** identifiedBy: Ana Lucia Henriques Oliveira | Allan Paulo Moreira dos Santos; **Event:** samplingProtocol: Malaise intercept trap; verbatimEventDate: 13.xi.12; **Record Level:** institutionCode: DZRJ; basisOfRecord: PreservedSpecimen
- **Type status:** Other material. **Occurrence:** recordedBy: Santos, A.P.M. | Takiya, D.M.; individualCount: 1; sex: male; lifeStage: adult; **Location:** country: Brazil; stateProvince: Ceará; municipality: Ubajara; locality: Parque Nacional de Ubajara, Cachoeira do Cafundó; maximumElevationInMeters: 783; verbatimCoordinates: 3°50'12"S, 40°54'35"W; **Identification:** identifiedBy: Ana Lucia Henriques Oliveira | Allan Paulo Moreira dos Santos; **Event:** samplingProtocol: Manual; verbatimEventDate: 16.ii.13; **Record Level:** institutionCode: DZRJ; basisOfRecord: PreservedSpecimen
- **Type status:** Other material. **Occurrence:** recordedBy: Santos, A.P.M. | Takiya, D.M.; individualCount: 1; sex: male; lifeStage: adult; **Location:** country: Brazil; stateProvince: Ceará; municipality: Ubajara; locality: Parque Nacional de Ubajara, Trilha Araticum, Rio Cafundó; maximumElevationInMeters: 753; verbatimCoordinates: 3°50'12"S, 40°54'31"W; **Identification:** identifiedBy: Ana Lucia Henriques Oliveira | Allan Paulo Moreira dos Santos; **Event:** samplingProtocol: Manual; verbatimEventDate: 17.ii.13; **Record Level:** institutionCode: DZRJ; basisOfRecord: PreservedSpecimen
- **Type status:** Other material. **Occurrence:** recordedBy: Santos, A.P.M. | Takiya, D.M.; individualCount: 12; lifeStage: immature; **Location:** country: Brazil; stateProvince: Ceará; municipality: Ubajara; locality: Parque Nacional de Ubajara, Trilha Araticum, Rio Cafundó; maximumElevationInMeters: 753; verbatimCoordinates: 3°50'12"S, 40°54'31"W; **Identification:** identifiedBy: Ana Lucia Henriques Oliveira | Allan Paulo Moreira dos Santos; **Event:** samplingProtocol: Manual; verbatimEventDate: 17.ii.13; **Record Level:** institutionCode: DZRJ; basisOfRecord: PreservedSpecimen
- **Type status:** Other material. **Occurrence:** recordedBy: Santos, A.P.M. | Takiya, D.M.; individualCount: 11; lifeStage: immature; **Location:** country: Brazil; stateProvince: Ceará; municipality: Ubajara; locality: Parque Nacional de Ubajara, Rio das Minas, próximo ao Portão Araticum; maximumElevationInMeters: 328; verbatimCoordinates: 3°49'32.6"S, 40°53'32.8"W; **Identification:** identifiedBy: Ana Lucia Henriques Oliveira | Allan Paulo Moreira dos Santos; **Event:** samplingProtocol: Manual; verbatimEventDate: 17.ii.13; **Record Level:** institutionCode: DZRJ; basisOfRecord: PreservedSpecimen
- **Type status:** Other material. **Occurrence:** recordedBy: Limeira-de-Oliveira | et al.; individualCount: 3; sex: male; lifeStage: adult; **Location:** country: Brazil; stateProvince: Ceará; municipality: Ubajara; locality: Parque Nacional de Ubajara, Rio Cafundó, pouco acima da cachoeira; maximumElevationInMeters: 795; verbatimCoordinates: 3°50'13"S, 40°54'35"W; **Identification:** identifiedBy: Ana Lucia Henriques Oliveira | Allan Paulo Moreira dos Santos; **Event:** samplingProtocol: Malaise intercept trap; verbatimEventDate: 18.xi.12; **Record Level:** institutionCode: DZRJ; basisOfRecord: PreservedSpecimen
- **Type status:** Other material. **Occurrence:** recordedBy: Limeira-de-Oliveira | et al.; individualCount: 3; sex: female; lifeStage: adult; **Location:** country: Brazil; stateProvince: Ceará; municipality: Ubajara; locality: Parque Nacional de Ubajara, Rio Cafundó, pouco acima da cachoeira; maximumElevationInMeters: 795; verbatimCoordinates: 3°50'13"S, 40°54'35"W; **Identification:** identifiedBy: Ana Lucia Henriques Oliveira | Allan Paulo Moreira dos Santos; **Event:** samplingProtocol: Malaise intercept trap; verbatimEventDate: 18.xi.12; **Record Level:** institutionCode: DZRJ; basisOfRecord: PreservedSpecimen
- **Type status:** Other material. **Occurrence:** recordedBy: Rafael, J.A. | Limeira-de-Oliveira, F. | Takiya, D.M. | et al.; individualCount: 2; sex: male; lifeStage: adult; **Location:** country: Brazil; stateProvince: Ceará; municipality: Ubajara; locality: Parque Nacional de Ubajara, Trilha Araticum, Rio das Minas na altura da trilha do teleférico; maximumElevationInMeters: 420; verbatimCoordinates: 3°49'58"S, 40°53'53"W; **Identification:** identifiedBy: Ana Lucia Henriques Oliveira| Allan Paulo Moreira dos Santos; **Event:** samplingProtocol: Malaise intercept trap; verbatimEventDate: 20.iv.12; **Record Level:** institutionCode: DZRJ; basisOfRecord: PreservedSpecimen
- **Type status:** Other material. **Occurrence:** recordedBy: Takiya, D.M. | Rafael, J.A.; individualCount: 1; sex: male; lifeStage: adult; **Location:** country: Brazil; stateProvince: Ceará; municipality: Ubajara; locality: Parque Nacional de Ubajara, Rio Cafundó, pouco acima da cachoeira; maximumElevationInMeters: 795; verbatimCoordinates: 3°50'13"S, 40°54'35"W; **Identification:** identifiedBy: Ana Lucia Henriques Oliveira | Allan Paulo Moreira dos Santos; **Event:** samplingProtocol: Pennsylvania light trap; verbatimEventDate: 24.iv.12; **Record Level:** institutionCode: DZRJ; basisOfRecord: PreservedSpecimen

##### Distribution

Brazil: CE.

##### Notes

Species described in Henriques-Oliveira and Santos 2014. See Fig. 26.

#### Nectopsyche

Müller, 1879

#### Nectopsyche sp.

##### Materials

- **Type status:** Other material. **Occurrence:** recordedBy: Takiya, D.M.; individualCount: 10; lifeStage: immature; **Location:** country: Brazil; stateProvince: Ceará; municipality: Ubajara; locality: Parque Nacional de Ubajara, Trilha Araticum, Rio Cafundó; maximumElevationInMeters: 753; verbatimCoordinates: 3°50'12"S, 40°54'31"W; **Identification:** identifiedBy: Allan Paulo Moreira dos Santos; **Event:** samplingProtocol: Manual; verbatimEventDate: 23.iv.12; **Record Level:** institutionCode: DZRJ; basisOfRecord: PreservedSpecimen

#### Oecetis

McLachlan, 1877

#### Oecetis fibra

Chen & Morse, 2012

##### Materials

- **Type status:** Other material. **Occurrence:** recordedBy: Santos, A.P.M. | Takiya, D.M.; individualCount: 1; sex: male; lifeStage: adult; **Location:** country: Brazil; stateProvince: Ceará; municipality: Ubajara; locality: Parque Nacional de Ubajara, Trilha Samambaia, Rio Gameleira; maximumElevationInMeters: 874; verbatimCoordinates: 3°50'25"S, 40°54'19"W; **Identification:** identifiedBy: Allan Paulo Moreira dos Santos; **Event:** samplingProtocol: Pennsylvania light trap; verbatimEventDate: 13.ii.13; **Record Level:** institutionCode: DZRJ; basisOfRecord: PreservedSpecimen
- **Type status:** Other material. **Occurrence:** recordedBy: Limeira-de-Oliveira | et al.; individualCount: 2; sex: male; lifeStage: adult; **Location:** country: Brazil; stateProvince: Ceará; municipality: Ubajara; locality: Parque Nacional de Ubajara, Rio Cafundó, pouco acima da cachoeira; maximumElevationInMeters: 795; verbatimCoordinates: 3°50'13"S, 40°54'35"W; **Identification:** identifiedBy: Ana Lucia Henriques Oliveira; **Event:** samplingProtocol: Malaise intercept trap; verbatimEventDate: 13.xi.12; **Record Level:** institutionCode: DZRJ; basisOfRecord: PreservedSpecimen
- **Type status:** Other material. **Occurrence:** recordedBy: Limeira-de-Oliveira | et al.; individualCount: 1; sex: female; lifeStage: adult; **Location:** country: Brazil; stateProvince: Ceará; municipality: Ubajara; locality: Parque Nacional de Ubajara, Rio Cafundó, pouco acima da cachoeira; maximumElevationInMeters: 795; verbatimCoordinates: 3°50'13"S, 40°54'35"W; **Identification:** identifiedBy: Ana Lucia Henriques Oliveira; **Event:** samplingProtocol: Malaise intercept trap; verbatimEventDate: 13.xi.12; **Record Level:** institutionCode: DZRJ; basisOfRecord: PreservedSpecimen
- **Type status:** Other material. **Occurrence:** recordedBy: Santos, A.P.M. | Takiya, D.M.; individualCount: 1; sex: female; lifeStage: adult; **Location:** country: Brazil; stateProvince: Ceará; municipality: Ubajara; locality: Parque Nacional de Ubajara, Trilha Samambaia, Rio Gameleira; maximumElevationInMeters: 874; verbatimCoordinates: 3°50'25"S, 40°54'19"W; **Identification:** identifiedBy: Ana Lucia Henriques Oliveira; **Event:** samplingProtocol: Pennsylvania light trap; verbatimEventDate: 14.ii.13; **Record Level:** institutionCode: DZRJ; basisOfRecord: PreservedSpecimen
- **Type status:** Other material. **Occurrence:** recordedBy: Limeira-de-Oliveira | et al.; individualCount: 1; sex: male; lifeStage: adult; **Location:** country: Brazil; stateProvince: Ceará; municipality: Ubajara; locality: Parque Nacional de Ubajara, Rio Cafundó, pouco acima da cachoeira; maximumElevationInMeters: 795; verbatimCoordinates: 3°50'13"S, 40°54'35"W; **Identification:** identifiedBy: Ana Lucia Henriques Oliveira; **Event:** samplingProtocol: Malaise intercept trap; verbatimEventDate: 18.xi.12; **Record Level:** institutionCode: DZRJ; basisOfRecord: PreservedSpecimen
- **Type status:** Other material. **Occurrence:** recordedBy: Limeira-de-Oliveira | et al.; individualCount: 1; sex: female; lifeStage: adult; **Location:** country: Brazil; stateProvince: Ceará; municipality: Ubajara; locality: Parque Nacional de Ubajara, Rio Cafundó, pouco acima da cachoeira; maximumElevationInMeters: 795; verbatimCoordinates: 3°50'13"S, 40°54'35"W; **Identification:** identifiedBy: Ana Lucia Henriques Oliveira; **Event:** samplingProtocol: Malaise intercept trap; verbatimEventDate: 18.xi.12; **Record Level:** institutionCode: DZRJ; basisOfRecord: PreservedSpecimen

##### Distribution

Brazil: CE!, ES, SP, RJ, PR, SC.

##### Notes

New species record for Northeastern Brazil.

#### Odontoceridae

#### Marilia

Müller, 1880

#### Marilia flexuosa

Ulmer, 1905

##### Materials

- **Type status:** Other material. **Occurrence:** recordedBy: Santos, A.P.M. | Takiya, D.M.; individualCount: 2; sex: female; lifeStage: adult; **Location:** country: Brazil; stateProvince: Ceará; municipality: Ubajara; locality: Parque Nacional de Ubajara, Trilha Samambaia, Rio Gameleira; maximumElevationInMeters: 874; verbatimCoordinates: 3°50'25"S, 40°54'19"W; **Identification:** identifiedBy: Allan Paulo Moreira dos Santos; **Event:** samplingProtocol: Pennsylvania light trap; verbatimEventDate: 13.ii.13; **Record Level:** institutionCode: DZRJ; basisOfRecord: PreservedSpecimen
- **Type status:** Other material. **Occurrence:** recordedBy: Santos, A.P.M. | Takiya, D.M.; individualCount: 1; sex: male; lifeStage: adult; **Location:** country: Brazil; stateProvince: Ceará; municipality: Ubajara; locality: Parque Nacional de Ubajara, Trilha Samambaia, Rio Gameleira; maximumElevationInMeters: 874; verbatimCoordinates: 3°50'25"S, 40°54'19"W; **Identification:** identifiedBy: Allan Paulo Moreira dos Santos; **Event:** samplingProtocol: Pennsylvania light trap; verbatimEventDate: 14.ii.13; **Record Level:** institutionCode: DZRJ; basisOfRecord: PreservedSpecimen
- **Type status:** Other material. **Occurrence:** recordedBy: Santos, A.P.M. | Takiya, D.M.; individualCount: 1; sex: female; lifeStage: adult; **Location:** country: Brazil; stateProvince: Ceará; municipality: Ubajara; locality: Parque Nacional de Ubajara, Trilha Samambaia, Rio Gameleira; maximumElevationInMeters: 874; verbatimCoordinates: 3°50'25"S, 40°54'19"W; **Identification:** identifiedBy: Allan Paulo Moreira dos Santos; **Event:** samplingProtocol: Pennsylvania light trap; verbatimEventDate: 14.ii.13; **Record Level:** institutionCode: DZRJ; basisOfRecord: PreservedSpecimen
- **Type status:** Other material. **Occurrence:** recordedBy: Santos, A.P.M. | Takiya, D.M.; individualCount: 1; sex: male; lifeStage: adult; **Location:** country: Brazil; stateProvince: Ceará; municipality: Ubajara; locality: Parque Nacional de Ubajara, Trilha Araticum, Rio das Minas; maximumElevationInMeters: 524; verbatimCoordinates: 3°50'3"S, 40°54'18"W; **Identification:** identifiedBy: Allan Paulo Moreira dos Santos; **Event:** samplingProtocol: Pennsylvania light trap; verbatimEventDate: 14.ii.13; **Record Level:** institutionCode: DZRJ; basisOfRecord: PreservedSpecimen
- **Type status:** Other material. **Occurrence:** recordedBy: Rafael, J.A. | Limeira-de-Oliveira, F. | Takiya, D.M. | Santos, A.P.M. | et al.; individualCount: 6; sex: male; lifeStage: adult; **Location:** country: Brazil; stateProvince: Ceará; municipality: Ubajara; locality: Parque Nacional de Ubajara, Trilha Araticum, Rio das Minas; maximumElevationInMeters: 524; verbatimCoordinates: 3°50'3"S, 40°54'18"W; **Identification:** identifiedBy: Allan Paulo Moreira dos Santos; **Event:** samplingProtocol: Malaise intercept trap; verbatimEventDate: 14.ii.13; **Record Level:** institutionCode: DZRJ; basisOfRecord: PreservedSpecimen
- **Type status:** Other material. **Occurrence:** recordedBy: Rafael, J.A. | Limeira-de-Oliveira, F. | Takiya, D.M. | Santos, A.P.M. | et al.; individualCount: 6; sex: female; lifeStage: adult; **Location:** country: Brazil; stateProvince: Ceará; municipality: Ubajara; locality: Parque Nacional de Ubajara, Trilha Araticum, Rio das Minas; maximumElevationInMeters: 524; verbatimCoordinates: 3°50'3"S, 40°54'18"W; **Identification:** identifiedBy: Allan Paulo Moreira dos Santos; **Event:** samplingProtocol: Malaise intercept trap; verbatimEventDate: 14.ii.13; **Record Level:** institutionCode: DZRJ; basisOfRecord: PreservedSpecimen
- **Type status:** Other material. **Occurrence:** recordedBy: Rafael, J.A. | Limeira-de-Oliveira, F. | Takiya, D.M. | Santos, A.P.M. | et al.; individualCount: 4; sex: male; lifeStage: adult; **Location:** country: Brazil; stateProvince: Ceará; municipality: Ubajara; locality: Parque Nacional de Ubajara, Trilha Araticum, Rio das Minas na altura da trilha do teleférico; maximumElevationInMeters: 420; verbatimCoordinates: 3°49'58"S, 40°53'53"W; **Identification:** identifiedBy: Allan Paulo Moreira dos Santos; **Event:** samplingProtocol: Malaise intercept trap; verbatimEventDate: 14.ii.13; **Record Level:** institutionCode: DZRJ; basisOfRecord: PreservedSpecimen
- **Type status:** Other material. **Occurrence:** recordedBy: Rafael, J.A. | Limeira-de-Oliveira, F. | Takiya, D.M. | Santos, A.P.M. | et al.; individualCount: 6; sex: female; lifeStage: adult; **Location:** country: Brazil; stateProvince: Ceará; municipality: Ubajara; locality: Parque Nacional de Ubajara, Trilha Araticum, Rio das Minas na altura da trilha do teleférico; maximumElevationInMeters: 420; verbatimCoordinates: 3°49'58"S, 40°53'53"W; **Identification:** identifiedBy: Allan Paulo Moreira dos Santos; **Event:** samplingProtocol: Malaise intercept trap; verbatimEventDate: 14.ii.13; **Record Level:** institutionCode: DZRJ; basisOfRecord: PreservedSpecimen
- **Type status:** Other material. **Occurrence:** recordedBy: Santos, A.P.M. | Takiya, D.M.; individualCount: 3; sex: male; lifeStage: adult; **Location:** country: Brazil; stateProvince: Ceará; municipality: Ubajara; locality: Parque Nacional de Ubajara, Trilha Araticum, Rio da Minas abaixo do teleférico; maximumElevationInMeters: 395; verbatimCoordinates: 3°49'43.3"S, 40°53'51.5"W; **Identification:** identifiedBy: Allan Paulo Moreira dos Santos; **Event:** samplingProtocol: Pennsylvania light trap; verbatimEventDate: 14.ii.13; **Record Level:** institutionCode: DZRJ; basisOfRecord: PreservedSpecimen
- **Type status:** Other material. **Occurrence:** recordedBy: Santos, A.P.M. | Takiya, D.M.; individualCount: 2; sex: female; lifeStage: adult; **Location:** country: Brazil; stateProvince: Ceará; municipality: Ubajara; locality: Parque Nacional de Ubajara, Trilha Araticum, Rio da Minas abaixo do teleférico; maximumElevationInMeters: 395; verbatimCoordinates: 3°49'43.3"S, 40°53'51.5"W; **Identification:** identifiedBy: Allan Paulo Moreira dos Santos; **Event:** samplingProtocol: Pennsylvania light trap; verbatimEventDate: 14.ii.13; **Record Level:** institutionCode: DZRJ; basisOfRecord: PreservedSpecimen
- **Type status:** Other material. **Occurrence:** recordedBy: Rafael, J.A. | Limeira-de-Oliveira, F. | Takiya, D.M. | Santos, A.P.M. | et al.; individualCount: 1; sex: male; lifeStage: adult; **Location:** country: Brazil; stateProvince: Ceará; municipality: Ubajara; locality: Parque Nacional de Ubajara, Rio das Minas, próximo ao Portão Araticum; maximumElevationInMeters: 328; verbatimCoordinates: 3°49'32.6"S, 40°53'32.8"W; **Identification:** identifiedBy: Allan Paulo Moreira dos Santos; **Event:** samplingProtocol: Malaise intercept trap; verbatimEventDate: 14.ii.13; **Record Level:** institutionCode: DZRJ; basisOfRecord: PreservedSpecimen
- **Type status:** Other material. **Occurrence:** recordedBy: Rafael, J.A. | Limeira-de-Oliveira, F. | Takiya, D.M. | Santos, A.P.M. | et al.; individualCount: 2; sex: male; lifeStage: adult; **Location:** country: Brazil; stateProvince: Ceará; municipality: Ubajara; locality: Parque Nacional de Ubajara, Rio das Minas, próximo ao Portão Araticum; maximumElevationInMeters: 328; verbatimCoordinates: 3°49'32.6"S, 40°53'32.8"W; **Identification:** identifiedBy: Allan Paulo Moreira dos Santos; **Event:** samplingProtocol: Malaise intercept trap; verbatimEventDate: 14.ii.13; **Record Level:** institutionCode: DZRJ; basisOfRecord: PreservedSpecimen
- **Type status:** Other material. **Occurrence:** recordedBy: Rafael, J.A. | Limeira-de-Oliveira, F. | Takiya, D.M. | Santos, A.P.M. | et al.; individualCount: 2; sex: female; lifeStage: adult; **Location:** country: Brazil; stateProvince: Ceará; municipality: Ubajara; locality: Parque Nacional de Ubajara, Rio das Minas, próximo ao Portão Araticum; maximumElevationInMeters: 328; verbatimCoordinates: 3°49'32.6"S, 40°53'32.8"W; **Identification:** identifiedBy: Allan Paulo Moreira dos Santos; **Event:** samplingProtocol: Malaise intercept trap; verbatimEventDate: 14.ii.13; **Record Level:** institutionCode: DZRJ; basisOfRecord: PreservedSpecimen
- **Type status:** Other material. **Occurrence:** recordedBy: Santos, A.P.M. | Takiya, D.M.; individualCount: 1; sex: female; lifeStage: adult; **Location:** country: Brazil; stateProvince: Ceará; municipality: Ubajara; locality: Parque Nacional de Ubajara, Cachoeira do Cafundó; maximumElevationInMeters: 783; verbatimCoordinates: 3°50'12"S, 40°54'35"W; **Identification:** identifiedBy: Allan Paulo Moreira dos Santos; **Event:** samplingProtocol: Pennsylvania light trap; verbatimEventDate: 15.ii.13; **Record Level:** institutionCode: DZRJ; basisOfRecord: PreservedSpecimen
- **Type status:** Other material. **Occurrence:** recordedBy: Rafael, J.A. | Limeira-de-Oliveira, F. | Takiya, D.M. | Santos, A.P.M. | et al.; individualCount: 1; sex: female; lifeStage: adult; **Location:** country: Brazil; stateProvince: Ceará; municipality: Ubajara; locality: Parque Nacional de Ubajara, Trilha Samambaia, Rio Gameleira; maximumElevationInMeters: 874; verbatimCoordinates: 3°50'25"S, 40°54'19"W; **Identification:** identifiedBy: Allan Paulo Moreira dos Santos; **Event:** samplingProtocol: Malaise intercept trap; verbatimEventDate: 17.ii.13; **Record Level:** institutionCode: DZRJ; basisOfRecord: PreservedSpecimen
- **Type status:** Other material. **Occurrence:** recordedBy: Rafael, J.A. | Limeira-de-Oliveira, F. | Takiya, D.M. | Santos, A.P.M. | et al.; individualCount: 1; sex: female; lifeStage: adult; **Location:** country: Brazil; stateProvince: Ceará; municipality: Ubajara; locality: Parque Nacional de Ubajara, Trilha Araticum, Rio das Minas; maximumElevationInMeters: 524; verbatimCoordinates: 3°50'3"S, 40°54'18"W; **Identification:** identifiedBy: Allan Paulo Moreira dos Santos; **Event:** samplingProtocol: Malaise intercept trap; verbatimEventDate: 17.ii.13; **Record Level:** institutionCode: DZRJ; basisOfRecord: PreservedSpecimen
- **Type status:** Other material. **Occurrence:** recordedBy: Rafael, J.A. | Limeira-de-Oliveira, F. | Takiya, D.M. | Santos, A.P.M. | et al.; individualCount: 3; sex: male; lifeStage: adult; **Location:** country: Brazil; stateProvince: Ceará; municipality: Ubajara; locality: Parque Nacional de Ubajara, Rio das Minas, próximo ao Portão Araticum; maximumElevationInMeters: 328; verbatimCoordinates: 3°49'32.6"S, 40°53'32.8"W; **Identification:** identifiedBy: Allan Paulo Moreira dos Santos; **Event:** samplingProtocol: Malaise intercept trap; verbatimEventDate: 17.ii.13; **Record Level:** institutionCode: DZRJ; basisOfRecord: PreservedSpecimen
- **Type status:** Other material. **Occurrence:** recordedBy: Rafael, J.A. | Limeira-de-Oliveira, F. | Takiya, D.M. | Santos, A.P.M. | et al.; individualCount: 2; sex: female; lifeStage: adult; **Location:** country: Brazil; stateProvince: Ceará; municipality: Ubajara; locality: Parque Nacional de Ubajara, Rio das Minas, próximo ao Portão Araticum; maximumElevationInMeters: 328; verbatimCoordinates: 3°49'32.6"S, 40°53'32.8"W; **Identification:** identifiedBy: Allan Paulo Moreira dos Santos; **Event:** samplingProtocol: Malaise intercept trap; verbatimEventDate: 17.ii.13; **Record Level:** institutionCode: DZRJ; basisOfRecord: PreservedSpecimen
- **Type status:** Other material. **Occurrence:** recordedBy: Limeira-de-Oliveira | et al.; individualCount: 1; sex: male; lifeStage: adult; **Location:** country: Brazil; stateProvince: Ceará; municipality: Ubajara; locality: Parque Nacional de Ubajara, Rio Cafundó, pouco acima da cachoeira; maximumElevationInMeters: 795; verbatimCoordinates: 3°50'13"S, 40°54'35"W; **Identification:** identifiedBy: Allan Paulo Moreira dos Santos; **Event:** samplingProtocol: Malaise intercept trap; verbatimEventDate: 19.i.13; **Record Level:** institutionCode: DZRJ; basisOfRecord: PreservedSpecimen
- **Type status:** Other material. **Occurrence:** recordedBy: Rafael, J.A. | Limeira-de-Oliveira, F. | Takiya, D.M. | et al.; individualCount: 1; sex: male; lifeStage: adult; **Location:** country: Brazil; stateProvince: Ceará; municipality: Ubajara; locality: Parque Nacional de Ubajara, Trilha Araticum, Rio das Minas na altura da trilha do teleférico; maximumElevationInMeters: 420; verbatimCoordinates: 3°49'58"S, 40°53'53"W; **Identification:** identifiedBy: Allan Paulo Moreira dos Santos; **Event:** samplingProtocol: Malaise intercept trap; verbatimEventDate: 20.iv.12; **Record Level:** institutionCode: DZRJ; basisOfRecord: PreservedSpecimen
- **Type status:** Other material. **Occurrence:** recordedBy: Rafael, J.A. | Limeira-de-Oliveira, F. | Takiya, D.M. | et al.; individualCount: 1; sex: female; lifeStage: adult; **Location:** country: Brazil; stateProvince: Ceará; municipality: Ubajara; locality: Parque Nacional de Ubajara, Trilha Araticum, Rio das Minas na altura da trilha do teleférico; maximumElevationInMeters: 420; verbatimCoordinates: 3°49'58"S, 40°53'53"W; **Identification:** identifiedBy: Allan Paulo Moreira dos Santos; **Event:** samplingProtocol: Malaise intercept trap; verbatimEventDate: 20.iv.12; **Record Level:** institutionCode: DZRJ; basisOfRecord: PreservedSpecimen
- **Type status:** Other material. **Occurrence:** recordedBy: Rafael, J.A. | Limeira-de-Oliveira, F. | Takiya, D.M. | et al.; individualCount: 1; sex: male; lifeStage: adult; **Location:** country: Brazil; stateProvince: Ceará; municipality: Ubajara; locality: Parque Nacional de Ubajara, Trilha Araticum, Rio das Minas na altura da trilha do teleférico; maximumElevationInMeters: 420; verbatimCoordinates: 3°49'58"S, 40°53'53"W; **Identification:** identifiedBy: Allan Paulo Moreira dos Santos; **Event:** samplingProtocol: Malaise intercept trap; verbatimEventDate: 20.iv.12; **Record Level:** institutionCode: DZRJ; basisOfRecord: PreservedSpecimen
- **Type status:** Other material. **Occurrence:** recordedBy: Rafael, J.A. | Limeira-de-Oliveira, F. | Takiya, D.M. | et al.; individualCount: 2; sex: male; lifeStage: adult; **Location:** country: Brazil; stateProvince: Ceará; municipality: Ubajara; locality: Parque Nacional de Ubajara, Trilha Araticum, Rio das Minas na altura da trilha do teleférico; maximumElevationInMeters: 420; verbatimCoordinates: 3°49'58"S, 40°53'53"W; **Identification:** identifiedBy: Allan Paulo Moreira dos Santos; **Event:** samplingProtocol: Malaise intercept trap; verbatimEventDate: 20.iv.12; **Record Level:** institutionCode: DZRJ; basisOfRecord: PreservedSpecimen
- **Type status:** Other material. **Occurrence:** recordedBy: Rafael, J.A. | Limeira-de-Oliveira, F. | Takiya, D.M. | et al.; individualCount: 5; sex: female; lifeStage: adult; **Location:** country: Brazil; stateProvince: Ceará; municipality: Ubajara; locality: Parque Nacional de Ubajara, Trilha Araticum, Rio das Minas na altura da trilha do teleférico; maximumElevationInMeters: 420; verbatimCoordinates: 3°49'58"S, 40°53'53"W; **Identification:** identifiedBy: Allan Paulo Moreira dos Santos; **Event:** samplingProtocol: Malaise intercept trap; verbatimEventDate: 20.iv.12; **Record Level:** institutionCode: DZRJ; basisOfRecord: PreservedSpecimen
- **Type status:** Other material. **Occurrence:** recordedBy: Rafael, J.A. | Limeira-de-Oliveira, F. | Takiya, D.M. | et al.; individualCount: 1; sex: female; lifeStage: adult; **Location:** country: Brazil; stateProvince: Ceará; municipality: Ubajara; locality: Parque Nacional de Ubajara, Trilha Araticum, Rio das Minas na altura da trilha do teleférico; maximumElevationInMeters: 420; verbatimCoordinates: 3°49'58"S, 40°53'53"W; **Identification:** identifiedBy: Allan Paulo Moreira dos Santos; **Event:** samplingProtocol: Malaise intercept trap; verbatimEventDate: 20.iv.12; **Record Level:** institutionCode: DZRJ; basisOfRecord: PreservedSpecimen
- **Type status:** Other material. **Occurrence:** recordedBy: Limeira-de-Oliveira | et al.; individualCount: 1; sex: male; lifeStage: adult; **Location:** country: Brazil; stateProvince: Ceará; municipality: Ubajara; locality: Parque Nacional de Ubajara, Trilha Araticum, Rio das Minas na altura da trilha do teleférico; maximumElevationInMeters: 420; verbatimCoordinates: 3°49'58"S, 40°53'53"W; **Identification:** identifiedBy: Allan Paulo Moreira dos Santos; **Event:** samplingProtocol: Malaise intercept trap; verbatimEventDate: 21.v.12; **Record Level:** institutionCode: DZRJ; basisOfRecord: PreservedSpecimen
- **Type status:** Other material. **Occurrence:** recordedBy: Limeira-de-Oliveira | et al.; individualCount: 4; sex: female; lifeStage: adult; **Location:** country: Brazil; stateProvince: Ceará; municipality: Ubajara; locality: Parque Nacional de Ubajara, Trilha Araticum, Rio das Minas na altura da trilha do teleférico; maximumElevationInMeters: 420; verbatimCoordinates: 3°49'58"S, 40°53'53"W; **Identification:** identifiedBy: Allan Paulo Moreira dos Santos; **Event:** samplingProtocol: Malaise intercept trap; verbatimEventDate: 21.v.12; **Record Level:** institutionCode: DZRJ; basisOfRecord: PreservedSpecimen
- **Type status:** Other material. **Occurrence:** recordedBy: Takiya, D.M. | Câmara, J.T.; individualCount: 5; sex: male; lifeStage: adult; **Location:** country: Brazil; stateProvince: Ceará; municipality: Ubajara; locality: Parque Nacional de Ubajara, Trilha Samambaia, Mirante da cachoeira do Gameleira; maximumElevationInMeters: 880; verbatimCoordinates: 3°50'21"S, 40°54'23"W; **Identification:** identifiedBy: Allan Paulo Moreira dos Santos; **Event:** samplingProtocol: Pennsylvania light trap; verbatimEventDate: 23.iv.12; **Record Level:** institutionCode: DZRJ; basisOfRecord: PreservedSpecimen

##### Distribution

Canada. USA. Mexico. Guatemala. Nicaragua. Costa Rica. Panama. Colombia. Brazil: CE!. Peru. Argentina.

##### Notes

New species record for Northeastern Brazil.

#### Chimarra

Stephens, 1829

#### Chimarra (Chimarra) calori

Blahnik & Holzenthal, 2012

##### Materials

- **Type status:** Other material. **Occurrence:** recordedBy: Rafael, J.A. | Limeira-de-Oliveira, F. | Takiya, D.M. | Santos, A.P.M. | et al.; individualCount: 4; sex: male; lifeStage: adult; **Location:** country: Brazil; stateProvince: Ceará; municipality: Ubajara; locality: Parque Nacional de Ubajara, Trilha Araticum, Rio das Minas; maximumElevationInMeters: 524; verbatimCoordinates: 3°50'3"S, 40°54'18"W; **Identification:** identifiedBy: Allan Paulo Moreira dos Santos; **Event:** samplingProtocol: Malaise intercept trap; verbatimEventDate: 14.ii.13; **Record Level:** institutionCode: DZRJ; basisOfRecord: PreservedSpecimen
- **Type status:** Other material. **Occurrence:** recordedBy: Rafael, J.A. | Limeira-de-Oliveira, F. | Takiya, D.M. | Santos, A.P.M. | et al.; individualCount: 4; sex: female; lifeStage: adult; **Location:** country: Brazil; stateProvince: Ceará; municipality: Ubajara; locality: Parque Nacional de Ubajara, Trilha Araticum, Rio das Minas; maximumElevationInMeters: 524; verbatimCoordinates: 3°50'3"S, 40°54'18"W; **Identification:** identifiedBy: Allan Paulo Moreira dos Santos; **Event:** samplingProtocol: Malaise intercept trap; verbatimEventDate: 14.ii.13; **Record Level:** institutionCode: DZRJ; basisOfRecord: PreservedSpecimen
- **Type status:** Other material. **Occurrence:** recordedBy: Rafael, J.A. | Limeira-de-Oliveira, F. | Takiya, D.M. | Santos, A.P.M. | et al.; individualCount: 2; sex: male; lifeStage: adult; **Location:** country: Brazil; stateProvince: Ceará; municipality: Ubajara; locality: Parque Nacional de Ubajara, Trilha Araticum, Rio das Minas na altura da trilha do teleférico; maximumElevationInMeters: 420; verbatimCoordinates: 3°49'58"S, 40°53'53"W; **Identification:** identifiedBy: Allan Paulo Moreira dos Santos; **Event:** samplingProtocol: Malaise intercept trap; verbatimEventDate: 14.ii.13; **Record Level:** institutionCode: DZRJ; basisOfRecord: PreservedSpecimen
- **Type status:** Other material. **Occurrence:** recordedBy: Rafael, J.A. | Limeira-de-Oliveira, F. | Takiya, D.M. | Santos, A.P.M. | et al.; individualCount: 6; sex: male; lifeStage: adult; **Location:** country: Brazil; stateProvince: Ceará; municipality: Ubajara; locality: Parque Nacional de Ubajara, Trilha Araticum, Rio das Minas na altura da trilha do teleférico; maximumElevationInMeters: 420; verbatimCoordinates: 3°49'58"S, 40°53'53"W; **Identification:** identifiedBy: Allan Paulo Moreira dos Santos; **Event:** samplingProtocol: Malaise intercept trap; verbatimEventDate: 14.ii.13; **Record Level:** institutionCode: DZRJ; basisOfRecord: PreservedSpecimen
- **Type status:** Other material. **Occurrence:** recordedBy: Rafael, J.A. | Limeira-de-Oliveira, F. | Takiya, D.M. | Santos, A.P.M. | et al.; individualCount: 8; sex: female; lifeStage: adult; **Location:** country: Brazil; stateProvince: Ceará; municipality: Ubajara; locality: Parque Nacional de Ubajara, Trilha Araticum, Rio das Minas na altura da trilha do teleférico; maximumElevationInMeters: 420; verbatimCoordinates: 3°49'58"S, 40°53'53"W; **Identification:** identifiedBy: Allan Paulo Moreira dos Santos; **Event:** samplingProtocol: Malaise intercept trap; verbatimEventDate: 14.ii.13; **Record Level:** institutionCode: DZRJ; basisOfRecord: PreservedSpecimen
- **Type status:** Other material. **Occurrence:** recordedBy: Rafael, J.A. | Limeira-de-Oliveira, F. | Takiya, D.M. | Santos, A.P.M. | et al.; individualCount: 4; sex: male; lifeStage: adult; **Location:** country: Brazil; stateProvince: Ceará; municipality: Ubajara; locality: Parque Nacional de Ubajara, Rio das Minas, próximo ao Portão Araticum; maximumElevationInMeters: 328; verbatimCoordinates: 3°49'32.6"S, 40°53'32.8"W; **Identification:** identifiedBy: Allan Paulo Moreira dos Santos; **Event:** samplingProtocol: Malaise intercept trap; verbatimEventDate: 14.ii.13; **Record Level:** institutionCode: DZRJ; basisOfRecord: PreservedSpecimen
- **Type status:** Other material. **Occurrence:** recordedBy: Rafael, J.A. | Limeira-de-Oliveira, F. | Takiya, D.M. | Santos, A.P.M. | et al.; individualCount: 13; sex: female; lifeStage: adult; **Location:** country: Brazil; stateProvince: Ceará; municipality: Ubajara; locality: Parque Nacional de Ubajara, Rio das Minas, próximo ao Portão Araticum; maximumElevationInMeters: 328; verbatimCoordinates: 3°49'32.6"S, 40°53'32.8"W; **Identification:** identifiedBy: Allan Paulo Moreira dos Santos; **Event:** samplingProtocol: Malaise intercept trap; verbatimEventDate: 14.ii.13; **Record Level:** institutionCode: DZRJ; basisOfRecord: PreservedSpecimen
- **Type status:** Other material. **Occurrence:** recordedBy: Rafael, J.A. | Limeira-de-Oliveira, F. | Takiya, D.M. | Santos, A.P.M. | et al.; individualCount: 3; sex: male; lifeStage: adult; **Location:** country: Brazil; stateProvince: Ceará; municipality: Ubajara; locality: Parque Nacional de Ubajara, Trilha Araticum, Rio das Minas; maximumElevationInMeters: 524; verbatimCoordinates: 3°50'3"S, 40°54'18"W; **Identification:** identifiedBy: Allan Paulo Moreira dos Santos; **Event:** samplingProtocol: Malaise intercept trap; verbatimEventDate: 17.ii.13; **Record Level:** institutionCode: DZRJ; basisOfRecord: PreservedSpecimen
- **Type status:** Other material. **Occurrence:** recordedBy: Rafael, J.A. | Limeira-de-Oliveira, F. | Takiya, D.M. | Santos, A.P.M. | et al.; individualCount: 10; sex: male; lifeStage: adult; **Location:** country: Brazil; stateProvince: Ceará; municipality: Ubajara; locality: Parque Nacional de Ubajara, Rio das Minas, próximo ao Portão Araticum; maximumElevationInMeters: 328; verbatimCoordinates: 3°49'32.6"S, 40°53'32.8"W; **Identification:** identifiedBy: Allan Paulo Moreira dos Santos; **Event:** samplingProtocol: Malaise intercept trap; verbatimEventDate: 17.ii.13; **Record Level:** institutionCode: DZRJ; basisOfRecord: PreservedSpecimen
- **Type status:** Other material. **Occurrence:** recordedBy: Rafael, J.A. | Limeira-de-Oliveira, F. | Takiya, D.M. | Santos, A.P.M. | et al.; individualCount: 14; sex: female; lifeStage: adult; **Location:** country: Brazil; stateProvince: Ceará; municipality: Ubajara; locality: Parque Nacional de Ubajara, Rio das Minas, próximo ao Portão Araticum; maximumElevationInMeters: 328; verbatimCoordinates: 3°49'32.6"S, 40°53'32.8"W; **Identification:** identifiedBy: Allan Paulo Moreira dos Santos; **Event:** samplingProtocol: Malaise intercept trap; verbatimEventDate: 17.ii.13; **Record Level:** institutionCode: DZRJ; basisOfRecord: PreservedSpecimen
- **Type status:** Other material. **Occurrence:** recordedBy: Rafael, J.A. | Limeira-de-Oliveira, F. | Takiya, D.M. | et al.; individualCount: 1; sex: male; lifeStage: adult; **Location:** country: Brazil; stateProvince: Ceará; municipality: Ubajara; locality: Parque Nacional de Ubajara, Trilha Araticum, Rio das Minas na altura da trilha do teleférico; maximumElevationInMeters: 420; verbatimCoordinates: 3°49'58"S, 40°53'53"W; **Identification:** identifiedBy: Allan Paulo Moreira dos Santos; **Event:** samplingProtocol: Malaise intercept trap; verbatimEventDate: 20.iv.12; **Record Level:** institutionCode: DZRJ; basisOfRecord: PreservedSpecimen
- **Type status:** Other material. **Occurrence:** recordedBy: Rafael, J.A. | Limeira-de-Oliveira, F. | Takiya, D.M. | et al.; individualCount: 5; sex: male; lifeStage: adult; **Location:** country: Brazil; stateProvince: Ceará; municipality: Ubajara; locality: Parque Nacional de Ubajara, Trilha Araticum, Rio das Minas na altura da trilha do teleférico; maximumElevationInMeters: 420; verbatimCoordinates: 3°49'58"S, 40°53'53"W; **Identification:** identifiedBy: Allan Paulo Moreira dos Santos; **Event:** samplingProtocol: Malaise intercept trap; verbatimEventDate: 20.iv.12; **Record Level:** institutionCode: DZRJ; basisOfRecord: PreservedSpecimen

##### Distribution

Brazil: CE!, MG, SP.

##### Notes

New species record for Northeastern Brazil.

#### Chimarra (Curgia) conica

Flint, 1983

##### Materials

- **Type status:** Other material. **Occurrence:** recordedBy: Rafael, J.A. | Limeira-de-Oliveira, F. | Takiya, D.M. | Santos, A.P.M. | et al.; individualCount: 1; sex: male; lifeStage: adult; **Location:** country: Brazil; stateProvince: Ceará; municipality: Ubajara; locality: Parque Nacional de Ubajara, Trilha Araticum, Rio das Minas na altura da trilha do teleférico; maximumElevationInMeters: 420; verbatimCoordinates: 3°49'58"S, 40°53'53"W; **Identification:** identifiedBy: Allan Paulo Moreira dos Santos; **Event:** samplingProtocol: Malaise intercept trap; verbatimEventDate: 14.ii.13; **Record Level:** institutionCode: DZRJ; basisOfRecord: PreservedSpecimen
- **Type status:** Other material. **Occurrence:** recordedBy: Rafael, J.A. | Limeira-de-Oliveira, F. | Takiya, D.M. | Santos, A.P.M. | et al.; individualCount: 3; sex: male; lifeStage: adult; **Location:** country: Brazil; stateProvince: Ceará; municipality: Ubajara; locality: Parque Nacional de Ubajara, Trilha Araticum, Rio das Minas na altura da trilha do teleférico; maximumElevationInMeters: 420; verbatimCoordinates: 3°49'58"S, 40°53'53"W; **Identification:** identifiedBy: Allan Paulo Moreira dos Santos; **Event:** samplingProtocol: Malaise intercept trap; verbatimEventDate: 14.ii.13; **Record Level:** institutionCode: DZRJ; basisOfRecord: PreservedSpecimen
- **Type status:** Other material. **Occurrence:** recordedBy: Rafael, J.A. | Limeira-de-Oliveira, F. | Takiya, D.M. | Santos, A.P.M. | et al.; individualCount: 5; sex: female; lifeStage: adult; **Location:** country: Brazil; stateProvince: Ceará; municipality: Ubajara; locality: Parque Nacional de Ubajara, Trilha Araticum, Rio das Minas na altura da trilha do teleférico; maximumElevationInMeters: 420; verbatimCoordinates: 3°49'58"S, 40°53'53"W; **Identification:** identifiedBy: Allan Paulo Moreira dos Santos; **Event:** samplingProtocol: Malaise intercept trap; verbatimEventDate: 14.ii.13; **Record Level:** institutionCode: DZRJ; basisOfRecord: PreservedSpecimen
- **Type status:** Other material. **Occurrence:** recordedBy: Rafael, J.A. | Limeira-de-Oliveira, F. | Takiya, D.M. | Santos, A.P.M. | et al.; individualCount: 25; sex: male; lifeStage: adult; **Location:** country: Brazil; stateProvince: Ceará; municipality: Ubajara; locality: Parque Nacional de Ubajara, Trilha Araticum, Rio das Minas na altura da trilha do teleférico; maximumElevationInMeters: 420; verbatimCoordinates: 3°49'58"S, 40°53'53"W; **Identification:** identifiedBy: Allan Paulo Moreira dos Santos; **Event:** samplingProtocol: Malaise intercept trap; verbatimEventDate: 14.ii.13; **Record Level:** institutionCode: DZRJ; basisOfRecord: PreservedSpecimen
- **Type status:** Other material. **Occurrence:** recordedBy: Rafael, J.A. | Limeira-de-Oliveira, F. | Takiya, D.M. | Santos, A.P.M. | et al.; individualCount: 12; sex: female; lifeStage: adult; **Location:** country: Brazil; stateProvince: Ceará; municipality: Ubajara; locality: Parque Nacional de Ubajara, Trilha Araticum, Rio das Minas na altura da trilha do teleférico; maximumElevationInMeters: 420; verbatimCoordinates: 3°49'58"S, 40°53'53"W; **Identification:** identifiedBy: Allan Paulo Moreira dos Santos; **Event:** samplingProtocol: Malaise intercept trap; verbatimEventDate: 14.ii.13; **Record Level:** institutionCode: DZRJ; basisOfRecord: PreservedSpecimen
- **Type status:** Other material. **Occurrence:** recordedBy: Rafael, J.A. | Limeira-de-Oliveira, F. | Takiya, D.M. | Santos, A.P.M. | et al.; individualCount: 12; sex: male; lifeStage: adult; **Location:** country: Brazil; stateProvince: Ceará; municipality: Ubajara; locality: Parque Nacional de Ubajara, Rio das Minas, próximo ao Portão Araticum; maximumElevationInMeters: 328; verbatimCoordinates: 3°49'32.6"S, 40°53'32.8"W; **Identification:** identifiedBy: Allan Paulo Moreira dos Santos; **Event:** samplingProtocol: Malaise intercept trap; verbatimEventDate: 14.ii.13; **Record Level:** institutionCode: DZRJ; basisOfRecord: PreservedSpecimen
- **Type status:** Other material. **Occurrence:** recordedBy: Rafael, J.A. | Limeira-de-Oliveira, F. | Takiya, D.M. | Santos, A.P.M. | et al.; individualCount: 39; sex: female; lifeStage: adult; **Location:** country: Brazil; stateProvince: Ceará; municipality: Ubajara; locality: Parque Nacional de Ubajara, Rio das Minas, próximo ao Portão Araticum; maximumElevationInMeters: 328; verbatimCoordinates: 3°49'32.6"S, 40°53'32.8"W; **Identification:** identifiedBy: Allan Paulo Moreira dos Santos; **Event:** samplingProtocol: Malaise intercept trap; verbatimEventDate: 14.ii.13; **Record Level:** institutionCode: DZRJ; basisOfRecord: PreservedSpecimen
- **Type status:** Other material. **Occurrence:** recordedBy: Rafael, J.A. | Limeira-de-Oliveira, F. | Takiya, D.M. | Santos, A.P.M. | et al.; individualCount: 19; sex: male; lifeStage: adult; **Location:** country: Brazil; stateProvince: Ceará; municipality: Ubajara; locality: Parque Nacional de Ubajara, Rio das Minas, próximo ao Portão Araticum; maximumElevationInMeters: 328; verbatimCoordinates: 3°49'32.6"S, 40°53'32.8"W; **Identification:** identifiedBy: Allan Paulo Moreira dos Santos; **Event:** samplingProtocol: Malaise intercept trap; verbatimEventDate: 14.ii.13; **Record Level:** institutionCode: DZRJ; basisOfRecord: PreservedSpecimen
- **Type status:** Other material. **Occurrence:** recordedBy: Rafael, J.A. | Limeira-de-Oliveira, F. | Takiya, D.M. | Santos, A.P.M. | et al.; individualCount: 37; sex: female; lifeStage: adult; **Location:** country: Brazil; stateProvince: Ceará; municipality: Ubajara; locality: Parque Nacional de Ubajara, Rio das Minas, próximo ao Portão Araticum; maximumElevationInMeters: 328; verbatimCoordinates: 3°49'32.6"S, 40°53'32.8"W; **Identification:** identifiedBy: Allan Paulo Moreira dos Santos; **Event:** samplingProtocol: Malaise intercept trap; verbatimEventDate: 14.ii.13; **Record Level:** institutionCode: DZRJ; basisOfRecord: PreservedSpecimen
- **Type status:** Other material. **Occurrence:** recordedBy: Rafael, J.A. | Limeira-de-Oliveira, F. | Takiya, D.M. | Santos, A.P.M. | et al.; individualCount: 2; sex: male; lifeStage: adult; **Location:** country: Brazil; stateProvince: Ceará; municipality: Ubajara; locality: Parque Nacional de Ubajara, Trilha Araticum, Rio das Minas; maximumElevationInMeters: 524; verbatimCoordinates: 3°50'3"S, 40°54'18"W; **Identification:** identifiedBy: Allan Paulo Moreira dos Santos; **Event:** samplingProtocol: Malaise intercept trap; verbatimEventDate: 17.ii.13; **Record Level:** institutionCode: DZRJ; basisOfRecord: PreservedSpecimen
- **Type status:** Other material. **Occurrence:** recordedBy: Rafael, J.A. | Limeira-de-Oliveira, F. | Takiya, D.M. | Santos, A.P.M. | et al.; individualCount: 19; sex: male; lifeStage: adult; **Location:** country: Brazil; stateProvince: Ceará; municipality: Ubajara; locality: Parque Nacional de Ubajara, Rio das Minas, próximo ao Portão Araticum; maximumElevationInMeters: 328; verbatimCoordinates: 3°49'32.6"S, 40°53'32.8"W; **Identification:** identifiedBy: Allan Paulo Moreira dos Santos; **Event:** samplingProtocol: Malaise intercept trap; verbatimEventDate: 17.ii.13; **Record Level:** institutionCode: DZRJ; basisOfRecord: PreservedSpecimen
- **Type status:** Other material. **Occurrence:** recordedBy: Rafael, J.A. | Limeira-de-Oliveira, F. | Takiya, D.M. | Santos, A.P.M. | et al.; individualCount: 13; sex: female; lifeStage: adult; **Location:** country: Brazil; stateProvince: Ceará; municipality: Ubajara; locality: Parque Nacional de Ubajara, Rio das Minas, próximo ao Portão Araticum; maximumElevationInMeters: 328; verbatimCoordinates: 3°49'32.6"S, 40°53'32.8"W; **Identification:** identifiedBy: Allan Paulo Moreira dos Santos; **Event:** samplingProtocol: Malaise intercept trap; verbatimEventDate: 17.ii.13; **Record Level:** institutionCode: DZRJ; basisOfRecord: PreservedSpecimen
- **Type status:** Other material. **Occurrence:** recordedBy: Rafael, J.A. | Limeira-de-Oliveira, F. | Takiya, D.M. | et al.; individualCount: 1; sex: male; lifeStage: adult; **Location:** country: Brazil; stateProvince: Ceará; municipality: Ubajara; locality: Parque Nacional de Ubajara, Trilha Araticum, Rio das Minas na altura da trilha do teleférico; maximumElevationInMeters: 420; verbatimCoordinates: 3°49'58"S, 40°53'53"W; **Identification:** identifiedBy: Allan Paulo Moreira dos Santos; **Event:** samplingProtocol: Malaise intercept trap; verbatimEventDate: 20.iv.12; **Record Level:** institutionCode: DZRJ; basisOfRecord: PreservedSpecimen
- **Type status:** Other material. **Occurrence:** recordedBy: Rafael, J.A. | Limeira-de-Oliveira, F. | Takiya, D.M. | et al.; individualCount: 3; sex: male; lifeStage: adult; **Location:** country: Brazil; stateProvince: Ceará; municipality: Ubajara; locality: Parque Nacional de Ubajara, Trilha Araticum, Rio das Minas na altura da trilha do teleférico; maximumElevationInMeters: 420; verbatimCoordinates: 3°49'58"S, 40°53'53"W; **Identification:** identifiedBy: Allan Paulo Moreira dos Santos; **Event:** samplingProtocol: Malaise intercept trap; verbatimEventDate: 20.iv.12; **Record Level:** institutionCode: DZRJ; basisOfRecord: PreservedSpecimen
- **Type status:** Other material. **Occurrence:** recordedBy: Rafael, J.A. | Limeira-de-Oliveira, F. | Takiya, D.M. | et al.; individualCount: 1; sex: female; lifeStage: adult; **Location:** country: Brazil; stateProvince: Ceará; municipality: Ubajara; locality: Parque Nacional de Ubajara, Trilha Araticum, Rio das Minas na altura da trilha do teleférico; maximumElevationInMeters: 420; verbatimCoordinates: 3°49'58"S, 40°53'53"W; **Identification:** identifiedBy: Allan Paulo Moreira dos Santos; **Event:** samplingProtocol: Malaise intercept trap; verbatimEventDate: 20.iv.12; **Record Level:** institutionCode: DZRJ; basisOfRecord: PreservedSpecimen
- **Type status:** Other material. **Occurrence:** recordedBy: Rafael, J.A. | Limeira-de-Oliveira, F. | Takiya, D.M. | et al.; individualCount: 7; sex: male; lifeStage: adult; **Location:** country: Brazil; stateProvince: Ceará; municipality: Ubajara; locality: Parque Nacional de Ubajara, Trilha Araticum, Rio das Minas na altura da trilha do teleférico; maximumElevationInMeters: 420; verbatimCoordinates: 3°49'58"S, 40°53'53"W; **Identification:** identifiedBy: Allan Paulo Moreira dos Santos; **Event:** samplingProtocol: Malaise intercept trap; verbatimEventDate: 20.iv.12; **Record Level:** institutionCode: DZRJ; basisOfRecord: PreservedSpecimen

##### Distribution

Brazil: CE, MT, RO, GO, MG, ES, RJ, SC.

#### Chimarra (Curgia) cultellata

Flint, 1983

##### Materials

- **Type status:** Other material. **Occurrence:** recordedBy: Rafael, J.A. | Limeira-de-Oliveira, F. | Takiya, D.M. | et al.; individualCount: 1; sex: male; lifeStage: adult; **Location:** country: Brazil; stateProvince: Ceará; municipality: Ubajara; locality: Parque Nacional de Ubajara, Trilha Araticum, Rio das Minas na altura da trilha do teleférico; maximumElevationInMeters: 420; verbatimCoordinates: 3°49'58"S, 40°53'53"W; **Identification:** identifiedBy: Allan Paulo Moreira dos Santos; **Event:** samplingProtocol: Malaise intercept trap; verbatimEventDate: 20.iv.12; **Record Level:** institutionCode: DZRJ; basisOfRecord: PreservedSpecimen
- **Type status:** Other material. **Occurrence:** recordedBy: Rafael, J.A. | Limeira-de-Oliveira, F. | Takiya, D.M. | et al.; individualCount: 1; sex: male; lifeStage: adult; **Location:** country: Brazil; stateProvince: Ceará; municipality: Ubajara; locality: Parque Nacional de Ubajara, Trilha Araticum, Rio das Minas na altura da trilha do teleférico; maximumElevationInMeters: 420; verbatimCoordinates: 3°49'58"S, 40°53'53"W; **Identification:** identifiedBy: Allan Paulo Moreira dos Santos; **Event:** samplingProtocol: Malaise intercept trap; verbatimEventDate: 20.iv.12; **Record Level:** institutionCode: DZRJ; basisOfRecord: PreservedSpecimen
- **Type status:** Other material. **Occurrence:** recordedBy: Takiya, D.M. | Somavilla, A.; individualCount: 3; sex: male; lifeStage: adult; **Location:** country: Brazil; stateProvince: Ceará; municipality: Ubajara; locality: Parque Nacional de Ubajara, Trilha Araticum, Rio das Minas; maximumElevationInMeters: 524; verbatimCoordinates: 3°50'3"S, 40°54'18"W; **Identification:** identifiedBy: Allan Paulo Moreira dos Santos; **Event:** samplingProtocol: Pennsylvania light trap; verbatimEventDate: 22.iv.12; **Record Level:** institutionCode: DZRJ; basisOfRecord: PreservedSpecimen
- **Type status:** Other material. **Occurrence:** recordedBy: Takiya, D.M. | Somavilla, A.; individualCount: 2; sex: female; lifeStage: adult; **Location:** country: Brazil; stateProvince: Ceará; municipality: Ubajara; locality: Parque Nacional de Ubajara, Trilha Araticum, Rio das Minas; maximumElevationInMeters: 524; verbatimCoordinates: 3°50'3"S, 40°54'18"W; **Identification:** identifiedBy: Allan Paulo Moreira dos Santos; **Event:** samplingProtocol: Pennsylvania light trap; verbatimEventDate: 22.iv.12; **Record Level:** institutionCode: DZRJ; basisOfRecord: PreservedSpecimen
- **Type status:** Other material. **Occurrence:** recordedBy: Takiya, D.M. | Rafael, J.A.; individualCount: 1; sex: male; lifeStage: adult; **Location:** country: Brazil; stateProvince: Ceará; municipality: Ubajara; locality: Parque Nacional de Ubajara, Rio Cafundó, pouco acima da cachoeira; maximumElevationInMeters: 795; verbatimCoordinates: 3°50'13"S, 40°54'35"W; **Identification:** identifiedBy: Allan Paulo Moreira dos Santos; **Event:** samplingProtocol: Pennsylvania light trap; verbatimEventDate: 24.iv.12; **Record Level:** institutionCode: DZRJ; basisOfRecord: PreservedSpecimen
- **Type status:** Other material. **Occurrence:** recordedBy: Rafael, J.A. | Limeira-de-Oliveira, F. | Takiya, D.M. | Santos, A.P.M. | et al.; individualCount: 12; sex: male; lifeStage: adult; **Location:** country: Brazil; stateProvince: Ceará; municipality: Ubajara; locality: Parque Nacional de Ubajara, Rio das Minas, próximo ao Portão Araticum; maximumElevationInMeters: 328; verbatimCoordinates: 3°49'32.6"S, 40°53'32.8"W; **Identification:** identifiedBy: Allan Paulo Moreira dos Santos; **Event:** samplingProtocol: Malaise intercept trap; verbatimEventDate: 14.ii.13; **Record Level:** institutionCode: DZRJ; basisOfRecord: PreservedSpecimen
- **Type status:** Other material. **Occurrence:** recordedBy: Rafael, J.A. | Limeira-de-Oliveira, F. | Takiya, D.M. | Santos, A.P.M. | et al.; individualCount: 39; sex: female; lifeStage: adult; **Location:** country: Brazil; stateProvince: Ceará; municipality: Ubajara; locality: Parque Nacional de Ubajara, Rio das Minas, próximo ao Portão Araticum; maximumElevationInMeters: 328; verbatimCoordinates: 3°49'32.6"S, 40°53'32.8"W; **Identification:** identifiedBy: Allan Paulo Moreira dos Santos; **Event:** samplingProtocol: Malaise intercept trap; verbatimEventDate: 14.ii.13; **Record Level:** institutionCode: DZRJ; basisOfRecord: PreservedSpecimen
- **Type status:** Other material. **Occurrence:** recordedBy: Rafael, J.A. | Limeira-de-Oliveira, F. | Takiya, D.M. | Santos, A.P.M. | et al.; individualCount: 19; sex: male; lifeStage: adult; **Location:** country: Brazil; stateProvince: Ceará; municipality: Ubajara; locality: Parque Nacional de Ubajara, Rio das Minas, próximo ao Portão Araticum; maximumElevationInMeters: 328; verbatimCoordinates: 3°49'32.6"S, 40°53'32.8"W; **Identification:** identifiedBy: Allan Paulo Moreira dos Santos; **Event:** samplingProtocol: Malaise intercept trap; verbatimEventDate: 14.ii.13; **Record Level:** institutionCode: DZRJ; basisOfRecord: PreservedSpecimen
- **Type status:** Other material. **Occurrence:** recordedBy: Rafael, J.A. | Limeira-de-Oliveira, F. | Takiya, D.M. | Santos, A.P.M. | et al.; individualCount: 37; sex: female; lifeStage: adult; **Location:** country: Brazil; stateProvince: Ceará; municipality: Ubajara; locality: Parque Nacional de Ubajara, Rio das Minas, próximo ao Portão Araticum; maximumElevationInMeters: 328; verbatimCoordinates: 3°49'32.6"S, 40°53'32.8"W; **Identification:** identifiedBy: Allan Paulo Moreira dos Santos; **Event:** samplingProtocol: Malaise intercept trap; verbatimEventDate: 14.ii.13; **Record Level:** institutionCode: DZRJ; basisOfRecord: PreservedSpecimen
- **Type status:** Other material. **Occurrence:** recordedBy: Rafael, J.A. | Limeira-de-Oliveira, F. | Takiya, D.M. | Santos, A.P.M. | et al.; individualCount: 2; sex: male; lifeStage: adult; **Location:** country: Brazil; stateProvince: Ceará; municipality: Ubajara; locality: Parque Nacional de Ubajara, Trilha Araticum, Rio das Minas; maximumElevationInMeters: 524; verbatimCoordinates: 3°50'3"S, 40°54'18"W; **Identification:** identifiedBy: Allan Paulo Moreira dos Santos; **Event:** samplingProtocol: Malaise intercept trap; verbatimEventDate: 17.ii.13; **Record Level:** institutionCode: DZRJ; basisOfRecord: PreservedSpecimen
- **Type status:** Other material. **Occurrence:** recordedBy: Rafael, J.A. | Limeira-de-Oliveira, F. | Takiya, D.M. | Santos, A.P.M. | et al.; individualCount: 19; sex: male; lifeStage: adult; **Location:** country: Brazil; stateProvince: Ceará; municipality: Ubajara; locality: Parque Nacional de Ubajara, Rio das Minas, próximo ao Portão Araticum; maximumElevationInMeters: 328; verbatimCoordinates: 3°49'32.6"S, 40°53'32.8"W; **Identification:** identifiedBy: Allan Paulo Moreira dos Santos; **Event:** samplingProtocol: Malaise intercept trap; verbatimEventDate: 17.ii.13; **Record Level:** institutionCode: DZRJ; basisOfRecord: PreservedSpecimen
- **Type status:** Other material. **Occurrence:** recordedBy: Rafael, J.A. | Limeira-de-Oliveira, F. | Takiya, D.M. | Santos, A.P.M. | et al.; individualCount: 13; sex: female; lifeStage: adult; **Location:** country: Brazil; stateProvince: Ceará; municipality: Ubajara; locality: Parque Nacional de Ubajara, Rio das Minas, próximo ao Portão Araticum; maximumElevationInMeters: 328; verbatimCoordinates: 3°49'32.6"S, 40°53'32.8"W; **Identification:** identifiedBy: Allan Paulo Moreira dos Santos; **Event:** samplingProtocol: Malaise intercept trap; verbatimEventDate: 17.ii.13; **Record Level:** institutionCode: DZRJ; basisOfRecord: PreservedSpecimen
- **Type status:** Other material. **Occurrence:** recordedBy: Rafael, J.A. | Limeira-de-Oliveira, F. | Takiya, D.M. | et al.; individualCount: 1; sex: male; lifeStage: adult; **Location:** country: Brazil; stateProvince: Ceará; municipality: Ubajara; locality: Parque Nacional de Ubajara, Trilha Araticum, Rio das Minas na altura da trilha do teleférico; maximumElevationInMeters: 420; verbatimCoordinates: 3°49'58"S, 40°53'53"W; **Identification:** identifiedBy: Allan Paulo Moreira dos Santos; **Event:** samplingProtocol: Malaise intercept trap; verbatimEventDate: 20.iv.12; **Record Level:** institutionCode: DZRJ; basisOfRecord: PreservedSpecimen
- **Type status:** Other material. **Occurrence:** recordedBy: Rafael, J.A. | Limeira-de-Oliveira, F. | Takiya, D.M. | et al.; individualCount: 3; sex: male; lifeStage: adult; **Location:** country: Brazil; stateProvince: Ceará; municipality: Ubajara; locality: Parque Nacional de Ubajara, Trilha Araticum, Rio das Minas na altura da trilha do teleférico; maximumElevationInMeters: 420; verbatimCoordinates: 3°49'58"S, 40°53'53"W; **Identification:** identifiedBy: Allan Paulo Moreira dos Santos; **Event:** samplingProtocol: Malaise intercept trap; verbatimEventDate: 20.iv.12; **Record Level:** institutionCode: DZRJ; basisOfRecord: PreservedSpecimen
- **Type status:** Other material. **Occurrence:** recordedBy: Rafael, J.A. | Limeira-de-Oliveira, F. | Takiya, D.M. | et al.; individualCount: 1; sex: female; lifeStage: adult; **Location:** country: Brazil; stateProvince: Ceará; municipality: Ubajara; locality: Parque Nacional de Ubajara, Trilha Araticum, Rio das Minas na altura da trilha do teleférico; maximumElevationInMeters: 420; verbatimCoordinates: 3°49'58"S, 40°53'53"W; **Identification:** identifiedBy: Allan Paulo Moreira dos Santos; **Event:** samplingProtocol: Malaise intercept trap; verbatimEventDate: 20.iv.12; **Record Level:** institutionCode: DZRJ; basisOfRecord: PreservedSpecimen
- **Type status:** Other material. **Occurrence:** recordedBy: Rafael, J.A. | Limeira-de-Oliveira, F. | Takiya, D.M. | et al.; individualCount: 7; sex: male; lifeStage: adult; **Location:** country: Brazil; stateProvince: Ceará; municipality: Ubajara; locality: Parque Nacional de Ubajara, Trilha Araticum, Rio das Minas na altura da trilha do teleférico; maximumElevationInMeters: 420; verbatimCoordinates: 3°49'58"S, 40°53'53"W; **Identification:** identifiedBy: Allan Paulo Moreira dos Santos; **Event:** samplingProtocol: Malaise intercept trap; verbatimEventDate: 20.iv.12; **Record Level:** institutionCode: DZRJ; basisOfRecord: PreservedSpecimen

##### Distribution

Brazil: PA, AM, CE!, RO, MG, DF, RJ, SC.

##### Notes

New species record for Northeastern Brazil.

#### Polycentropodidae

##### Notes

New family record for CE.

#### Cernotina

Ross, 1938

##### Notes

New genus record for CE.

#### Cernotina sp. 2*

##### Materials

- **Type status:** Other material. **Occurrence:** recordedBy: Santos, A.P.M. | Takiya, D.M.; individualCount: 1; sex: male; lifeStage: adult; **Location:** country: Brazil; stateProvince: Ceará; municipality: Ubajara; locality: Parque Nacional de Ubajara, Trilha Samambaia, Rio Gameleira; maximumElevationInMeters: 874; verbatimCoordinates: 3°50'25"S, 40°54'19"W; **Identification:** identifiedBy: Allan Paulo Moreira dos Santos; **Event:** samplingProtocol: Pennsylvania light trap; verbatimEventDate: 13.ii.13; **Record Level:** institutionCode: DZRJ; basisOfRecord: PreservedSpecimen
- **Type status:** Other material. **Occurrence:** recordedBy: Rafael, J.A. | Limeira-de-Oliveira, F. | Takiya, D.M. | Santos, A.P.M. | et al.; individualCount: 1; sex: male; lifeStage: adult; **Location:** country: Brazil; stateProvince: Ceará; municipality: Ubajara; locality: Parque Nacional de Ubajara, Rio Cafundó, pouco acima da cachoeira; maximumElevationInMeters: 795; verbatimCoordinates: 3°50'13"S, 40°54'35"W; **Identification:** identifiedBy: Allan Paulo Moreira dos Santos; **Event:** samplingProtocol: Malaise intercept trap; verbatimEventDate: 13.ii.13; **Record Level:** institutionCode: DZRJ; basisOfRecord: PreservedSpecimen
- **Type status:** Other material. **Occurrence:** recordedBy: Santos, A.P.M. | Takiya, D.M.; individualCount: 1; sex: male; lifeStage: adult; **Location:** country: Brazil; stateProvince: Ceará; municipality: Ubajara; locality: Parque Nacional de Ubajara, Trilha Samambaia, Rio Gameleira; maximumElevationInMeters: 874; verbatimCoordinates: 3°50'25"S, 40°54'19"W; **Identification:** identifiedBy: Allan Paulo Moreira dos Santos; **Event:** samplingProtocol: Pennsylvania light trap; verbatimEventDate: 14.ii.13; **Record Level:** institutionCode: DZRJ; basisOfRecord: PreservedSpecimen
- **Type status:** Other material. **Occurrence:** recordedBy: Rafael, J.A. | Limeira-de-Oliveira, F. | Takiya, D.M. | Santos, A.P.M. | et al.; individualCount: 1; sex: male; lifeStage: adult; **Location:** country: Brazil; stateProvince: Ceará; municipality: Ubajara; locality: Parque Nacional de Ubajara, Trilha Samambaia, Rio Gameleira; maximumElevationInMeters: 874; verbatimCoordinates: 3°50'25"S, 40°54'19"W; **Identification:** identifiedBy: Allan Paulo Moreira dos Santos; **Event:** samplingProtocol: Malaise intercept trap; verbatimEventDate: 17.ii.13; **Record Level:** institutionCode: DZRJ; basisOfRecord: PreservedSpecimen
- **Type status:** Other material. **Occurrence:** recordedBy: Rafael, J.A. | Limeira-de-Oliveira, F. | Takiya, D.M. | Santos, A.P.M. | et al.; individualCount: 1; sex: male; lifeStage: adult; **Location:** country: Brazil; stateProvince: Ceará; municipality: Ubajara; locality: Parque Nacional de Ubajara, Trilha Araticum, Rio das Minas; maximumElevationInMeters: 524; verbatimCoordinates: 3°50'3"S, 40°54'18"W; **Identification:** identifiedBy: Allan Paulo Moreira dos Santos; **Event:** samplingProtocol: Malaise intercept trap; verbatimEventDate: 17.ii.13; **Record Level:** institutionCode: DZRJ; basisOfRecord: PreservedSpecimen
- **Type status:** Other material. **Occurrence:** recordedBy: Rafael, J.A. | Limeira-de-Oliveira, F. | Takiya, D.M. | et al.; individualCount: 2; sex: male; lifeStage: adult; **Location:** country: Brazil; stateProvince: Ceará; municipality: Ubajara; locality: Parque Nacional de Ubajara, Trilha Araticum, Rio das Minas na altura da trilha do teleférico; maximumElevationInMeters: 420; verbatimCoordinates: 3°49'58"S, 40°53'53"W; **Identification:** identifiedBy: Allan Paulo Moreira dos Santos; **Event:** samplingProtocol: Malaise intercept trap; verbatimEventDate: 20.iv.12; **Record Level:** institutionCode: DZRJ; basisOfRecord: PreservedSpecimen
- **Type status:** Other material. **Occurrence:** recordedBy: Rafael, J.A. | Limeira-de-Oliveira, F. | Takiya, D.M. | et al.; individualCount: 1; sex: female; lifeStage: adult; **Location:** country: Brazil; stateProvince: Ceará; municipality: Ubajara; locality: Parque Nacional de Ubajara, Trilha Araticum, Rio das Minas na altura da trilha do teleférico; maximumElevationInMeters: 420; verbatimCoordinates: 3°49'58"S, 40°53'53"W; **Identification:** identifiedBy: Allan Paulo Moreira dos Santos; **Event:** samplingProtocol: Malaise intercept trap; verbatimEventDate: 20.iv.12; **Record Level:** institutionCode: DZRJ; basisOfRecord: PreservedSpecimen
- **Type status:** Other material. **Occurrence:** recordedBy: Rafael, J.A. | Limeira-de-Oliveira, F. | Takiya, D.M. | et al.; individualCount: 2; sex: male; lifeStage: adult; **Location:** country: Brazil; stateProvince: Ceará; municipality: Ubajara; locality: Parque Nacional de Ubajara, Trilha Araticum, Rio das Minas na altura da trilha do teleférico; maximumElevationInMeters: 420; verbatimCoordinates: 3°49'58"S, 40°53'53"W; **Identification:** identifiedBy: Allan Paulo Moreira dos Santos; **Event:** samplingProtocol: Malaise intercept trap; verbatimEventDate: 20.iv.12; **Record Level:** institutionCode: DZRJ; basisOfRecord: PreservedSpecimen
- **Type status:** Other material. **Occurrence:** recordedBy: Rafael, J.A. | Limeira-de-Oliveira, F. | Takiya, D.M. | et al.; individualCount: 10; sex: male; lifeStage: adult; **Location:** country: Brazil; stateProvince: Ceará; municipality: Ubajara; locality: Parque Nacional de Ubajara, Rio Cafundó, pouco acima da cachoeira; maximumElevationInMeters: 795; verbatimCoordinates: 3°50'13"S, 40°54'35"W; **Identification:** identifiedBy: Allan Paulo Moreira dos Santos; **Event:** samplingProtocol: Malaise intercept trap; verbatimEventDate: 21.iv.12; **Record Level:** institutionCode: DZRJ; basisOfRecord: PreservedSpecimen

#### Polycentropus

Curtis, 1835

##### Notes

New genus record for CE.

#### Polycentropus sp. 1

##### Materials

- **Type status:** Other material. **Occurrence:** recordedBy: Rafael, J.A. | Limeira-de-Oliveira, F. | Takiya, D.M. | Santos, A.P.M. | et al.; individualCount: 1; sex: male; lifeStage: adult; **Location:** country: Brazil; stateProvince: Ceará; municipality: Ubajara; locality: Parque Nacional de Ubajara, Trilha Araticum, Rio das Minas; maximumElevationInMeters: 524; verbatimCoordinates: 3°50'3"S, 40°54'18"W; **Identification:** identifiedBy: Allan Paulo Moreira dos Santos; **Event:** samplingProtocol: Malaise intercept trap; verbatimEventDate: 14.ii.13; **Record Level:** institutionCode: DZRJ; basisOfRecord: PreservedSpecimen

#### Xiphocentronidae

##### Notes

New family record for CE.

#### Xiphocentron

Brauer, 1870

##### Notes

New genus record for CE.

#### Xiphocentron sp. 1

##### Materials

- **Type status:** Other material. **Occurrence:** recordedBy: Rafael, J.A. | Limeira-de-Oliveira, F. | Takiya, D.M. | Santos, A.P.M. | et al.; individualCount: 1; sex: male; lifeStage: adult; **Location:** country: Brazil; stateProvince: Ceará; municipality: Ubajara; locality: Parque Nacional de Ubajara, Rio Cafundó, pouco acima da cachoeira; maximumElevationInMeters: 795; verbatimCoordinates: 3°50'13"S, 40°54'35"W; **Identification:** identifiedBy: Allan Paulo Moreira dos Santos; **Event:** samplingProtocol: Malaise intercept trap; verbatimEventDate: 13.ii.13; **Record Level:** institutionCode: DZRJ; basisOfRecord: PreservedSpecimen
- **Type status:** Other material. **Occurrence:** recordedBy: Rafael, J.A. | Limeira-de-Oliveira, F. | Takiya, D.M. | Santos, A.P.M. | et al.; individualCount: 3; sex: female; lifeStage: adult; **Location:** country: Brazil; stateProvince: Ceará; municipality: Ubajara; locality: Parque Nacional de Ubajara, Rio Cafundó, pouco acima da cachoeira; maximumElevationInMeters: 795; verbatimCoordinates: 3°50'13"S, 40°54'35"W; **Identification:** identifiedBy: Allan Paulo Moreira dos Santos; **Event:** samplingProtocol: Malaise intercept trap; verbatimEventDate: 13.ii.13; **Record Level:** institutionCode: DZRJ; basisOfRecord: PreservedSpecimen
- **Type status:** Other material. **Occurrence:** recordedBy: Limeira-de-Oliveira | et al.; individualCount: 2; sex: male; lifeStage: adult; **Location:** country: Brazil; stateProvince: Ceará; municipality: Ubajara; locality: Parque Nacional de Ubajara, Rio Cafundó, pouco acima da cachoeira; maximumElevationInMeters: 795; verbatimCoordinates: 3°50'13"S, 40°54'35"W; **Identification:** identifiedBy: Allan Paulo Moreira dos Santos; **Event:** samplingProtocol: Malaise intercept trap; verbatimEventDate: 13.xi.12; **Record Level:** institutionCode: DZRJ; basisOfRecord: PreservedSpecimen

### Aquatic insects from Parque Nacional de Sete Cidades (PNSC)

#### Coleoptera

#### Epimetopidae

##### Notes

New family record for PI.

#### Epimetopus

Lacordaire, 1854

##### Notes

New genus record for PI.

#### Epimetopus lacordairei

Orchymont, 1933

##### Materials

- **Type status:** Other material. **Occurrence:** recordedBy: Takiya, D.M.; individualCount: 1; sex: female; lifeStage: adult; **Location:** country: Brazil; stateProvince: Piauí; municipality: Piracuruca; locality: Parque Nacional de Sete Cidades, Cachoeira do Riachão; maximumElevationInMeters: 171; verbatimCoordinates: 4°6'28"S, 41°40'13"W; **Identification:** identifiedBy: Bruno Clarkson; **Event:** samplingProtocol: White sheet light trap; verbatimEventDate: 18.iv.12; **Record Level:** institutionCode: DZRJ; basisOfRecord: PreservedSpecimen

##### Distribution

Brazil: PI!, MS. Bolivia. Paraguay

##### Notes

New species record for Northeastern Brazil.

#### Hydrophilidae

#### Crenitulus

Winters, 1926

##### Notes

New genus record for PI.

#### Crenitulus suturalis

(LeConte, 1866)

##### Materials

- **Type status:** Other material. **Occurrence:** recordedBy: Santos, A.P.M. | Takiya, D.M.; individualCount: 1; sex: female; lifeStage: adult; **Location:** country: Brazil; stateProvince: Piauí; municipality: Piracuruca; locality: Parque Nacional de Sete Cidades, Olha d'água dos Milagres; maximumElevationInMeters: 180; verbatimCoordinates: 4°5'31.8"S, 41°40'48.2"W; **Identification:** identifiedBy: Bruno Clarkson; **Event:** samplingProtocol: Pennsylvania light trap; verbatimEventDate: 15.ii.13; **Record Level:** institutionCode: DZRJ; basisOfRecord: PreservedSpecimen
- **Type status:** Other material. **Occurrence:** recordedBy: Takiya, D.M.; individualCount: 1; sex: male; lifeStage: adult; **Location:** country: Brazil; stateProvince: Piauí; municipality: Piracuruca; locality: Parque Nacional de Sete Cidades, Riacho da Piedade; maximumElevationInMeters: 169; verbatimCoordinates: 4°6'34"S, 41°43'39"W; **Identification:** identifiedBy: Bruno Clarkson; **Event:** samplingProtocol: Manual; verbatimEventDate: 18.iv.12; **Record Level:** institutionCode: DZRJ; basisOfRecord: PreservedSpecimen

##### Distribution

USA. Mexico. Belize. Guatemala. Honduras. Nicaragua. Costa Rica. Dominican Republic. Jamaica. Brazil: PI!, CE, PE, AL, MS, SP, RJ, SC. Peru. Bolivia. Paraguay. Argentina.

##### Notes

New species record for PI.

#### Berosus

Leach, 1817

#### Berosus patruelis

Berg, 1885

##### Materials

- **Type status:** Other material. **Occurrence:** recordedBy: Takiya, D.M.; individualCount: 4; sex: male; lifeStage: adult; **Location:** country: Brazil; stateProvince: Piauí; municipality: Piracuruca; locality: Parque Nacional de Sete Cidades, Cachoeira do Riachão; maximumElevationInMeters: 171; verbatimCoordinates: 4°6'28"S, 41°40'13"W; **Identification:** identifiedBy: Bruno Clarkson; **Event:** samplingProtocol: Pennsylvania light trap; verbatimEventDate: 18.iv.12; **Record Level:** institutionCode: DZRJ; basisOfRecord: PreservedSpecimen
- **Type status:** Other material. **Occurrence:** recordedBy: Takiya, D.M.; individualCount: 1; sex: female; lifeStage: adult; **Location:** country: Brazil; stateProvince: Piauí; municipality: Piracuruca; locality: Parque Nacional de Sete Cidades, Cachoeira do Riachão; maximumElevationInMeters: 171; verbatimCoordinates: 4°6'28"S, 41°40'13"W; **Identification:** identifiedBy: Bruno Clarkson; **Event:** samplingProtocol: Pennsylvania light trap; verbatimEventDate: 18.iv.12; **Record Level:** institutionCode: DZRJ; basisOfRecord: PreservedSpecimen
- **Type status:** Other material. **Occurrence:** recordedBy: Takiya, D.M.; individualCount: 1; sex: male; lifeStage: adult; **Location:** country: Brazil; stateProvince: Piauí; municipality: Piracuruca; locality: Parque Nacional de Sete Cidades, Alojamento; maximumElevationInMeters: 193; verbatimCoordinates: 4°5'57"S, 41°42'34"W; **Identification:** identifiedBy: Bruno Clarkson; **Event:** samplingProtocol: White sheet light trap; verbatimEventDate: 18.iv.12; **Record Level:** institutionCode: DZRJ; basisOfRecord: PreservedSpecimen
- **Type status:** Other material. **Occurrence:** recordedBy: Takiya, D.M.; individualCount: 1; sex: female; lifeStage: adult; **Location:** country: Brazil; stateProvince: Piauí; municipality: Piracuruca; locality: Parque Nacional de Sete Cidades, Alojamento; maximumElevationInMeters: 193; verbatimCoordinates: 4°5'57"S, 41°42'34"W; **Identification:** identifiedBy: Bruno Clarkson; **Event:** samplingProtocol: White sheet light trap; verbatimEventDate: 18.iv.12; **Record Level:** institutionCode: DZRJ; basisOfRecord: PreservedSpecimen

##### Distribution

Venezuela. Brazil: PI, PE, MS. Bolivia. Paraguay. Argentina.

#### Berosus consobrinus

Knisch, 1921

##### Materials

- **Type status:** Other material. **Occurrence:** recordedBy: Takiya, D.M.; individualCount: 2; sex: female; lifeStage: adult; **Location:** country: Brazil; stateProvince: Piauí; municipality: Piracuruca; locality: Parque Nacional de Sete Cidades, Cachoeira do Riachão; maximumElevationInMeters: 171; verbatimCoordinates: 4°6'28"S, 41°40'13"W; **Identification:** identifiedBy: Bruno Clarkson; **Event:** samplingProtocol: Pennsylvania light trap; verbatimEventDate: 18.iv.12; **Record Level:** institutionCode: DZRJ; basisOfRecord: PreservedSpecimen
- **Type status:** Other material. **Occurrence:** recordedBy: Takiya, D.M.; individualCount: 2; sex: male; lifeStage: adult; **Location:** country: Brazil; stateProvince: Piauí; municipality: Piracuruca; locality: Parque Nacional de Sete Cidades, Cachoeira do Riachão; maximumElevationInMeters: 171; verbatimCoordinates: 4°6'28"S, 41°40'13"W; **Identification:** identifiedBy: Bruno Clarkson; **Event:** samplingProtocol: Pennsylvania light trap; verbatimEventDate: 18.iv.12; **Record Level:** institutionCode: DZRJ; basisOfRecord: PreservedSpecimen
- **Type status:** Other material. **Occurrence:** recordedBy: Takiya, D.M.; individualCount: 3; sex: male; lifeStage: adult; **Location:** country: Brazil; stateProvince: Piauí; municipality: Piracuruca; locality: Parque Nacional de Sete Cidades, Alojamento; maximumElevationInMeters: 193; verbatimCoordinates: 4°5'57"S, 41°42'34"W; **Identification:** identifiedBy: Bruno Clarkson; **Event:** samplingProtocol: White sheet light trap; verbatimEventDate: 18.iv.12; **Record Level:** institutionCode: DZRJ; basisOfRecord: PreservedSpecimen
- **Type status:** Other material. **Occurrence:** recordedBy: Takiya, D.M.; individualCount: 2; sex: female; lifeStage: adult; **Location:** country: Brazil; stateProvince: Piauí; municipality: Piracuruca; locality: Parque Nacional de Sete Cidades, Alojamento; maximumElevationInMeters: 193; verbatimCoordinates: 4°5'57"S, 41°42'34"W; **Identification:** identifiedBy: Bruno Clarkson; **Event:** samplingProtocol: White sheet light trap; verbatimEventDate: 18.iv.12; **Record Level:** institutionCode: DZRJ; basisOfRecord: PreservedSpecimen

##### Distribution

Venezuela. Brazil: PI!, MS.

##### Notes

New species record for Northeastern Brazil.

#### Berosus erraticus

Mouchamps, 1963

##### Materials

- **Type status:** Other material. **Occurrence:** recordedBy: Takiya, D.M.; individualCount: 1; sex: male; lifeStage: adult; **Location:** country: Brazil; stateProvince: Piauí; municipality: Piracuruca; locality: Parque Nacional de Sete Cidades, Cachoeira do Riachão; maximumElevationInMeters: 171; verbatimCoordinates: 4°6'28"S, 41°40'13"W; **Identification:** identifiedBy: Bruno Clarkson; **Event:** samplingProtocol: Pennsylvania light trap; verbatimEventDate: 18.iv.12; **Record Level:** institutionCode: DZRJ; basisOfRecord: PreservedSpecimen

##### Distribution

Venezuela. Brazil: AM, PI!, RJ. Bolivia. Paraguay. Argentina. Uruguay.

##### Notes

New species record for Northeastern Brazil.

#### Berosus festivus

Berg, 1885

##### Materials

- **Type status:** Other material. **Occurrence:** recordedBy: Santos, A.P.M. | Takiya, D.M.; individualCount: 1; sex: male; lifeStage: adult; **Location:** country: Brazil; stateProvince: Piauí; municipality: Piracuruca; locality: Parque Nacional de Sete Cidades, Cachoeira do Riachão; maximumElevationInMeters: 171; verbatimCoordinates: 4°6'28"S, 41°40'13"W; **Identification:** identifiedBy: Bruno Clarkson; **Event:** samplingProtocol: Manual; verbatimEventDate: 19.ii.13; **Record Level:** institutionCode: DZRJ; basisOfRecord: PreservedSpecimen
- **Type status:** Other material. **Occurrence:** recordedBy: Santos, A.P.M. | Takiya, D.M.; individualCount: 1; sex: male; lifeStage: adult; **Location:** country: Brazil; stateProvince: Piauí; municipality: Piracuruca; locality: Parque Nacional de Sete Cidades, Poço do Bananeira; maximumElevationInMeters: 158; verbatimCoordinates: 4°5'55.8"S, 41°40'33.8"W; **Identification:** identifiedBy: Bruno Clarkson; **Event:** samplingProtocol: Manual; verbatimEventDate: 9.ii.13; **Record Level:** institutionCode: DZRJ; basisOfRecord: PreservedSpecimen

##### Distribution

Venezuela. Guyana. Brazil: PI!, CE, PE, MT, MS, RJ. Argentina. Uruguay.

##### Notes

New species record for PI.

#### Berosus sp. 1

##### Materials

- **Type status:** Other material. **Occurrence:** recordedBy: Santos, A.P.M. | Takiya, D.M.; individualCount: 1; sex: female; lifeStage: adult; **Location:** country: Brazil; stateProvince: Piauí; municipality: Piracuruca; locality: Parque Nacional de Sete Cidades, Riacho da Bananeira; maximumElevationInMeters: 189; verbatimCoordinates: 4°5'59"S, 41°40'48"W; **Identification:** identifiedBy: Bruno Clarkson; **Event:** samplingProtocol: Manual; verbatimEventDate: 11.ii.13; **Record Level:** institutionCode: DZRJ; basisOfRecord: PreservedSpecimen

#### Berosus sp. 2

##### Materials

- **Type status:** Other material. **Occurrence:** recordedBy: Takiya, D.M.; individualCount: 1; sex: female; lifeStage: adult; **Location:** country: Brazil; stateProvince: Piauí; municipality: Piracuruca; locality: Parque Nacional de Sete Cidades, Cachoeira do Riachão; maximumElevationInMeters: 171; verbatimCoordinates: 4°6'28"S, 41°40'13"W; **Identification:** identifiedBy: Bruno Clarkson; **Event:** samplingProtocol: Pennsylvania light trap; verbatimEventDate: 19.iv.12; **Record Level:** institutionCode: DZRJ; basisOfRecord: PreservedSpecimen
- **Type status:** Other material. **Occurrence:** recordedBy: Takiya, D.M.; individualCount: 2; sex: male; lifeStage: adult; **Location:** country: Brazil; stateProvince: Piauí; municipality: Piracuruca; locality: Parque Nacional de Sete Cidades, Cachoeira do Riachão; maximumElevationInMeters: 171; verbatimCoordinates: 4°6'28"S, 41°40'13"W; **Identification:** identifiedBy: Bruno Clarkson; **Event:** samplingProtocol: Pennsylvania light trap; verbatimEventDate: 19.iv.12; **Record Level:** institutionCode: DZRJ; basisOfRecord: PreservedSpecimen
- **Type status:** Other material. **Occurrence:** recordedBy: Takiya, D.M.; individualCount: 2; sex: female; lifeStage: adult; **Location:** country: Brazil; stateProvince: Piauí; municipality: Piracuruca; locality: Parque Nacional de Sete Cidades, Cachoeira do Riachão; maximumElevationInMeters: 171; verbatimCoordinates: 4°6'28"S, 41°40'13"W; **Identification:** identifiedBy: Bruno Clarkson; **Event:** samplingProtocol: Pennsylvania light trap; verbatimEventDate: 19.iv.12; **Record Level:** institutionCode: DZRJ; basisOfRecord: PreservedSpecimen
- **Type status:** Other material. **Occurrence:** recordedBy: Takiya, D.M.; individualCount: 1; sex: male; lifeStage: adult; **Location:** country: Brazil; stateProvince: Piauí; municipality: Piracuruca; locality: Parque Nacional de Sete Cidades, Cachoeira do Riachão; maximumElevationInMeters: 171; verbatimCoordinates: 4°6'28"S, 41°40'13"W; **Identification:** identifiedBy: Bruno Clarkson; **Event:** samplingProtocol: Pennsylvania light trap; verbatimEventDate: 19.iv.12; **Record Level:** institutionCode: DZRJ; basisOfRecord: PreservedSpecimen

##### Notes

Undescribed species.

#### Chasmogenus

Sharp, 1882

##### Notes

New genus record for PI.

#### Chasmogenus (Chasmogenus) sp. 1*

##### Materials

- **Type status:** Other material. **Occurrence:** recordedBy: Takiya, D.M.; individualCount: 1; lifeStage: adult; **Location:** country: Brazil; stateProvince: Piauí; municipality: Piracuruca; locality: Parque Nacional de Sete Cidades, Cachoeira do Riachão; maximumElevationInMeters: 171; verbatimCoordinates: 4°6'28"S, 41°40'13"W; **Identification:** identifiedBy: Bruno Clarkson; **Event:** samplingProtocol: Pennsylvania light trap; verbatimEventDate: 18.iv.12; **Record Level:** institutionCode: DZRJ; basisOfRecord: PreservedSpecimen
- **Type status:** Other material. **Occurrence:** recordedBy: Takiya, D.M.; individualCount: 2; lifeStage: adult; **Location:** country: Brazil; stateProvince: Piauí; municipality: Piracuruca; locality: Parque Nacional de Sete Cidades, Cachoeira do Riachão; maximumElevationInMeters: 171; verbatimCoordinates: 4°6'28"S, 41°40'13"W; **Identification:** identifiedBy: Bruno Clarkson; **Event:** samplingProtocol: Pennsylvania light trap; verbatimEventDate: 20.iv.12; **Record Level:** institutionCode: DZRJ; basisOfRecord: PreservedSpecimen

#### Enochrus

Thomson, 1859

#### Enochrus (Methydrus) atlantis

Orchymont, 1943

##### Materials

- **Type status:** Other material. **Occurrence:** recordedBy: Santos, A.P.M. | Takiya, D.M.; individualCount: 1; sex: male; lifeStage: adult; **Location:** country: Brazil; stateProvince: Piauí; municipality: Piracuruca; locality: Parque Nacional de Sete Cidades, Alojamento; maximumElevationInMeters: 193; verbatimCoordinates: 4°5'57"S, 41°42'34"W; **Identification:** identifiedBy: Bruno Clarkson; **Event:** samplingProtocol: White sheet light trap; verbatimEventDate: 8.11.13; **Record Level:** institutionCode: DZRJ; basisOfRecord: PreservedSpecimen

##### Distribution

Brazil: PI, PE, MS, SP, RJ.

#### Enochrus (Methydrus) sp. 1

##### Materials

- **Type status:** Other material. **Occurrence:** recordedBy: Santos, A.P.M. | Takiya, D.M.; individualCount: 1; sex: male; lifeStage: adult; **Location:** country: Brazil; stateProvince: Piauí; municipality: Piracuruca; locality: Parque Nacional de Sete Cidades, Olha d'água dos Milagres; maximumElevationInMeters: 180; verbatimCoordinates: 4°5'31.8"S, 41°40'48.2"W; **Identification:** identifiedBy: Bruno Clarkson; **Event:** samplingProtocol: Manual; verbatimEventDate: 9.11.13; **Record Level:** institutionCode: DZRJ; basisOfRecord: PreservedSpecimen

#### Helochares

Mulsant, 1844

##### Notes

New genus record for PI.

#### Helochares (Helochares) tectiformis

Fernández 1982

##### Materials

- **Type status:** Other material. **Occurrence:** recordedBy: Santos, A.P.M. | Takiya, D.M.; individualCount: 1; sex: female; lifeStage: adult; **Location:** country: Brazil; stateProvince: Piauí; municipality: Piracuruca; locality: Parque Nacional de Sete Cidades, Cachoeira do Riachão; maximumElevationInMeters: 171; verbatimCoordinates: 4°6'28"S, 41°40'13"W; **Identification:** identifiedBy: Bruno Clarkson; **Event:** samplingProtocol: Pennsylvania light trap; verbatimEventDate: 10.ii.13; **Record Level:** institutionCode: DZRJ; basisOfRecord: PreservedSpecimen
- **Type status:** Other material. **Occurrence:** recordedBy: Takiya, D.M.; individualCount: 1; sex: male; lifeStage: adult; **Location:** country: Brazil; stateProvince: Piauí; municipality: Piracuruca; locality: Parque Nacional de Sete Cidades, Cachoeira do Riachão; maximumElevationInMeters: 171; verbatimCoordinates: 4°6'28"S, 41°40'13"W; **Identification:** identifiedBy: Bruno Clarkson; **Event:** samplingProtocol: Pennsylvania light trap; verbatimEventDate: 18.iv.12; **Record Level:** institutionCode: DZRJ; basisOfRecord: PreservedSpecimen

##### Distribution

Venezuela. Brazil: PI!, MS. Paraguay. Argentina.

##### Notes

New species record for Northeastern Brazil.

#### Helochares (Helochares) sp. 1*

##### Materials

- **Type status:** Other material. **Occurrence:** recordedBy: Takiya, D.M.; individualCount: 2; sex: female; lifeStage: adult; **Location:** country: Brazil; stateProvince: Piauí; municipality: Piracuruca; locality: Parque Nacional de Sete Cidades, Alojamento; maximumElevationInMeters: 193; verbatimCoordinates: 4°5'57"S, 41°42'34"W; **Identification:** identifiedBy: Bruno Clarkson; **Event:** samplingProtocol: White sheet light trap; verbatimEventDate: 18.iv.12; **Record Level:** institutionCode: DZRJ; basisOfRecord: PreservedSpecimen

#### Hemiosus

Sharp, 1882

#### Hemiosus varidius

Orchymont, 1940

##### Materials

- **Type status:** Other material. **Occurrence:** recordedBy: Santos, A.P.M. | Takiya, D.M.; individualCount: 2; sex: male; lifeStage: adult; **Location:** country: Brazil; stateProvince: Piauí; municipality: Piracuruca; locality: Parque Nacional de Sete Cidades, Cachoeira do Riachão; maximumElevationInMeters: 171; verbatimCoordinates: 4°6'28"S, 41°40'13"W; **Identification:** identifiedBy: Bruno Clarkson; **Event:** samplingProtocol: Manual; verbatimEventDate: 19.ii.13; **Record Level:** institutionCode: DZRJ; basisOfRecord: PreservedSpecimen
- **Type status:** Other material. **Occurrence:** recordedBy: Santos, A.P.M. | Takiya, D.M.; individualCount: 2; sex: female; lifeStage: adult; **Location:** country: Brazil; stateProvince: Piauí; municipality: Piracuruca; locality: Parque Nacional de Sete Cidades, Cachoeira do Riachão; maximumElevationInMeters: 171; verbatimCoordinates: 4°6'28"S, 41°40'13"W; **Identification:** identifiedBy: Bruno Clarkson; **Event:** samplingProtocol: Manual; verbatimEventDate: 19.ii.13; **Record Level:** institutionCode: DZRJ; basisOfRecord: PreservedSpecimen
- **Type status:** Other material. **Occurrence:** recordedBy: Santos, A.P.M. | Takiya, D.M.; individualCount: 1; lifeStage: immature; **Location:** country: Brazil; stateProvince: Piauí; municipality: Piracuruca; locality: Parque Nacional de Sete Cidades, Cachoeira do Riachão; maximumElevationInMeters: 171; verbatimCoordinates: 4°6'28"S, 41°40'13"W; **Identification:** identifiedBy: Bruno Clarkson; **Event:** samplingProtocol: Manual; verbatimEventDate: 19.ii.13; **Record Level:** institutionCode: DZRJ; basisOfRecord: PreservedSpecimen
- **Type status:** Other material. **Occurrence:** recordedBy: Takiya, D.M.; individualCount: 6; sex: male; lifeStage: adult; **Location:** country: Brazil; stateProvince: Piauí; municipality: Piracuruca; locality: Parque Nacional de Sete Cidades, Cachoeira do Riachão; maximumElevationInMeters: 171; verbatimCoordinates: 4°6'28"S, 41°40'13"W; **Identification:** identifiedBy: Bruno Clarkson; **Event:** samplingProtocol: Pennsylvania light trap; verbatimEventDate: 19.iv.12; **Record Level:** institutionCode: DZRJ; basisOfRecord: PreservedSpecimen
- **Type status:** Other material. **Occurrence:** recordedBy: Takiya, D.M.; individualCount: 9; sex: female; lifeStage: adult; **Location:** country: Brazil; stateProvince: Piauí; municipality: Piracuruca; locality: Parque Nacional de Sete Cidades, Cachoeira do Riachão; maximumElevationInMeters: 171; verbatimCoordinates: 4°6'28"S, 41°40'13"W; **Identification:** identifiedBy: Bruno Clarkson; **Event:** samplingProtocol: Pennsylvania light trap; verbatimEventDate: 19.iv.12; **Record Level:** institutionCode: DZRJ; basisOfRecord: PreservedSpecimen
- **Type status:** Other material. **Occurrence:** recordedBy: Takiya, D.M.; individualCount: 1; sex: male; lifeStage: adult; **Location:** country: Brazil; stateProvince: Piauí; municipality: Piracuruca; locality: Parque Nacional de Sete Cidades, Cachoeira do Riachão; maximumElevationInMeters: 171; verbatimCoordinates: 4°6'28"S, 41°40'13"W; **Identification:** identifiedBy: Bruno Clarkson; **Event:** samplingProtocol: Pennsylvania light trap; verbatimEventDate: 19.iv.12; **Record Level:** institutionCode: DZRJ; basisOfRecord: PreservedSpecimen

##### Distribution

Brazil: PI!, PE.

##### Notes

New species record for PI.

#### Hemiosus sp. 1

##### Materials

- **Type status:** Other material. **Occurrence:** recordedBy: Takiya, D.M.; individualCount: 1; sex: male; lifeStage: adult; **Location:** country: Brazil; stateProvince: Piauí; municipality: Piracuruca; locality: Parque Nacional de Sete Cidades, Cachoeira do Riachão; maximumElevationInMeters: 171; verbatimCoordinates: 4°6'28"S, 41°40'13"W; **Identification:** identifiedBy: Bruno Clarkson; **Event:** samplingProtocol: Pennsylvania light trap; verbatimEventDate: 18.iv.12; **Record Level:** institutionCode: DZRJ; basisOfRecord: PreservedSpecimen
- **Type status:** Other material. **Occurrence:** recordedBy: Takiya, D.M.; individualCount: 3; sex: female; lifeStage: adult; **Location:** country: Brazil; stateProvince: Piauí; municipality: Piracuruca; locality: Parque Nacional de Sete Cidades, Cachoeira do Riachão; maximumElevationInMeters: 171; verbatimCoordinates: 4°6'28"S, 41°40'13"W; **Identification:** identifiedBy: Bruno Clarkson; **Event:** samplingProtocol: Pennsylvania light trap; verbatimEventDate: 19.iv.12; **Record Level:** institutionCode: DZRJ; basisOfRecord: PreservedSpecimen
- **Type status:** Other material. **Occurrence:** recordedBy: Santos, A.P.M. | Takiya, D.M.; individualCount: 1; sex: female; lifeStage: adult; **Location:** country: Brazil; stateProvince: Piauí; municipality: Piracuruca; locality: Parque Nacional de Sete Cidades, Olha d'água dos Milagres; maximumElevationInMeters: 180; verbatimCoordinates: 4°5'31.8"S, 41°40'48.2"W; **Identification:** identifiedBy: Bruno Clarkson; **Event:** samplingProtocol: Manual; verbatimEventDate: 11.ii.13; **Record Level:** institutionCode: DZRJ; basisOfRecord: PreservedSpecimen

#### Oocyclus

Sharp, 1882

##### Notes

New genus record for PI.

#### Oocyclus schubarti

Orchymont, 1940

##### Materials

- **Type status:** Other material. **Occurrence:** recordedBy: Santos, A.P.M. | Takiya, D.M.; individualCount: 1; sex: female; lifeStage: adult; **Location:** country: Brazil; stateProvince: Piauí; municipality: Piracuruca; locality: Parque Nacional de Sete Cidades, Cachoeira do Riachão; maximumElevationInMeters: 171; verbatimCoordinates: 4°6'28"S, 41°40'13"W; **Identification:** identifiedBy: Bruno Clarkson; **Event:** samplingProtocol: Manual; verbatimEventDate: 9.ii.13; **Record Level:** institutionCode: DZRJ; basisOfRecord: PreservedSpecimen

##### Distribution

Brazil: PI!, CE!, AL. Argentina?

##### Notes

New species record for PI.

#### Paracymus

Thomson, 1867

#### Paracymus gracilis

Orchymont, 1942

##### Materials

- **Type status:** Other material. **Occurrence:** recordedBy: Santos, A.P.M. | Takiya, D.M.; individualCount: 1; sex: male; lifeStage: adult; **Location:** country: Brazil; stateProvince: Piauí; municipality: Piracuruca; locality: Parque Nacional de Sete Cidades, Olho d'água Piscina do Bacuri; maximumElevationInMeters: 171; verbatimCoordinates: 4°6'1.2"S, 41°42'38.8"W; **Identification:** identifiedBy: Bruno Clarkson; **Event:** samplingProtocol: White sheet light trap; verbatimEventDate: 11.ii.13; **Record Level:** institutionCode: DZRJ; basisOfRecord: PreservedSpecimen
- **Type status:** Other material. **Occurrence:** recordedBy: Santos, A.P.M. | Takiya, D.M.; individualCount: 3; sex: female; lifeStage: adult; **Location:** country: Brazil; stateProvince: Piauí; municipality: Piracuruca; locality: Parque Nacional de Sete Cidades, Olho d'água Piscina do Bacuri; maximumElevationInMeters: 171; verbatimCoordinates: 4°6'1.2"S, 41°42'38.8"W; **Identification:** identifiedBy: Bruno Clarkson; **Event:** samplingProtocol: White sheet light trap; verbatimEventDate: 11.ii.13; **Record Level:** institutionCode: DZRJ; basisOfRecord: PreservedSpecimen
- **Type status:** Other material. **Occurrence:** recordedBy: Santos, A.P.M. | Takiya, D.M.; individualCount: 1; sex: male; lifeStage: adult; **Location:** country: Brazil; stateProvince: Piauí; municipality: Piracuruca; locality: Parque Nacional de Sete Cidades, Poço do Bananeira; maximumElevationInMeters: 158; verbatimCoordinates: 4°5'55.8"S, 41°40'33.8"W; **Identification:** identifiedBy: Bruno Clarkson; **Event:** samplingProtocol: Pennsylvania light trap; verbatimEventDate: 11.ii.13; **Record Level:** institutionCode: DZRJ; basisOfRecord: PreservedSpecimen
- **Type status:** Other material. **Occurrence:** recordedBy: Takiya, D.M.; individualCount: 1; sex: male; lifeStage: adult; **Location:** country: Brazil; stateProvince: Piauí; municipality: Piracuruca; locality: Parque Nacional de Sete Cidades, Cachoeira do Riachão; maximumElevationInMeters: 171; verbatimCoordinates: 4°6'28"S, 41°40'13"W; **Identification:** identifiedBy: Bruno Clarkson; **Event:** samplingProtocol: Pennsylvania light trap; verbatimEventDate: 18.iv.12; **Record Level:** institutionCode: DZRJ; basisOfRecord: PreservedSpecimen
- **Type status:** Other material. **Occurrence:** recordedBy: Takiya, D.M.; individualCount: 2; sex: female; lifeStage: adult; **Location:** country: Brazil; stateProvince: Piauí; municipality: Piracuruca; locality: Parque Nacional de Sete Cidades, Cachoeira do Riachão; maximumElevationInMeters: 171; verbatimCoordinates: 4°6'28"S, 41°40'13"W; **Identification:** identifiedBy: Bruno Clarkson; **Event:** samplingProtocol: Pennsylvania light trap; verbatimEventDate: 18.iv.12; **Record Level:** institutionCode: DZRJ; basisOfRecord: PreservedSpecimen

##### Distribution

Brazil: PI!, PE, SP. Paraguay.

##### Notes

New species record for PI.

#### Paracymus limbatus

Wooldridge, 1973

##### Materials

- **Type status:** Other material. **Occurrence:** recordedBy: Santos, A.P.M. | Takiya, D.M.; individualCount: 1; sex: male; lifeStage: adult; **Location:** country: Brazil; stateProvince: Piauí; municipality: Piracuruca; locality: Parque Nacional de Sete Cidades, Cachoeira do Riachão; maximumElevationInMeters: 171; verbatimCoordinates: 4°6'28"S, 41°40'13"W; **Identification:** identifiedBy: Bruno Clarkson; **Event:** samplingProtocol: Pennsylvania light trap; verbatimEventDate: 10.ii.13; **Record Level:** institutionCode: DZRJ; basisOfRecord: PreservedSpecimen

##### Distribution

Trinidad and Tobago. Colombia. Venezuela. Suriname. Brazil: PA, PI!, RN, BA, MT, SP. Peru. Bolivia. Paraguay.

##### Notes

New species record for PI.

#### Tropisternus

Solier, 1834

#### Tropisternus (Strepitornus) collaris

Fabricius, 1775

##### Materials

- **Type status:** Other material. **Occurrence:** recordedBy: Takiya, D.M.; individualCount: 1; sex: male; lifeStage: adult; **Location:** country: Brazil; stateProvince: Piauí; municipality: Piracuruca; locality: Parque Nacional de Sete Cidades, Riacho da Bananeira; maximumElevationInMeters: 189; verbatimCoordinates: 4°5'59"S, 41°40'48"W; **Identification:** identifiedBy: Bruno Clarkson; **Event:** samplingProtocol: Pennsylvania light trap; verbatimEventDate: 18.iv.12; **Record Level:** institutionCode: DZRJ; basisOfRecord: PreservedSpecimen

##### Distribution

USA. Mexico. Nicaragua. Costa Rica. Cuba. Haiti. Virgin Islands. Venezuela. Brazil: AM, CE, PI, PE, AL, SP, RJ, PR, RS. Peru. Bolivia. Paraguay. Argentina.

#### Tropisternus (Pristoternus) laevis

(Sturm, 1826)

##### Materials

- **Type status:** Other material. **Occurrence:** recordedBy: Santos, A.P.M. | Takiya, D.M.; individualCount: 1; sex: male; lifeStage: adult; **Location:** country: Brazil; stateProvince: Piauí; municipality: Piracuruca; locality: Parque Nacional de Sete Cidades, Riacho da Bananeira; maximumElevationInMeters: 189; verbatimCoordinates: 4°5'59"S, 41°40'48"W; **Identification:** identifiedBy: Bruno Clarkson; **Event:** samplingProtocol: Manual; verbatimEventDate: 11.ii.13; **Record Level:** institutionCode: DZRJ; basisOfRecord: PreservedSpecimen

##### Distribution

Venezuela. Guyana. French Guiana. Brazil: PA, AM, PI, CE, PE, AL, RJ. Paraguay. Argentina.

#### Diptera

#### Empididae

##### Notes

New family record for PI.

#### Hemerodromiinae

##### Notes

New subfamily record for PI.

#### Hemerodromia

Meigen, 1823

##### Notes

New genus record for PI.

#### Hemerodromia mesomelaena

Bezzi, 1909

##### Materials

- **Type status:** Other material. **Occurrence:** recordedBy: Rafael, J.A. | Limeira-de-Oliveira, F. | Takiya, D.M. | Santos, A.P.M. | et al.; individualCount: 7; lifeStage: adult; **Location:** country: Brazil; stateProvince: Piauí; municipality: Piracuruca; locality: Parque Nacional de Sete Cidades, Alojamento; maximumElevationInMeters: 193; verbatimCoordinates: 4°5'57"S, 41°42'34"W; **Identification:** identifiedBy: Josenir Teixeira Câmara; **Event:** samplingProtocol: Malaise intercept trap; verbatimEventDate: 14.ii.13; **Record Level:** institutionCode: DZRJ; basisOfRecord: PreservedSpecimen

##### Distribution

Brazil: PI!, CE!, PR. Peru. Argentina.

##### Notes

New species record for Northeastern Brazil.

#### Ephemeroptera

#### Baetidae

#### Callibaetis

Eaton, 1881

#### Callibaetis itannae

Cruz, Salles & Hamada, 2014

##### Materials

- **Type status:** Other material. **Occurrence:** recordedBy: Takiya, D.M.; individualCount: 11; sex: male; lifeStage: adult; **Location:** country: Brazil; stateProvince: Piauí; municipality: Piracuruca; locality: Parque Nacional de Sete Cidades, Cachoeira do Riachão; maximumElevationInMeters: 171; verbatimCoordinates: 4°6'28"S, 41°40'13"W; **Identification:** identifiedBy: Inês Corrêa Gonçalves; **Event:** samplingProtocol: Pennsylvania light trap; verbatimEventDate: 18.iv.12; **Record Level:** institutionCode: DZRJ; basisOfRecord: PreservedSpecimen
- **Type status:** Other material. **Occurrence:** recordedBy: Takiya, D.M.; individualCount: 3; sex: female; lifeStage: adult; **Location:** country: Brazil; stateProvince: Piauí; municipality: Piracuruca; locality: Parque Nacional de Sete Cidades, Cachoeira do Riachão; maximumElevationInMeters: 171; verbatimCoordinates: 4°6'28"S, 41°40'13"W; **Identification:** identifiedBy: Inês Corrêa Gonçalves; **Event:** samplingProtocol: Pennsylvania light trap; verbatimEventDate: 18.iv.12; **Record Level:** institutionCode: DZRJ; basisOfRecord: PreservedSpecimen

##### Distribution

Brazil: PI!, RO.

##### Notes

New species record for Northeastern Brazil.

#### Callibaetis nigracyclus

Cruz, Salles & Hamada, 2014

##### Materials

- **Type status:** Other material. **Occurrence:** recordedBy: Santos, A.P.M. | Takiya, D.M.; individualCount: 3; sex: male; lifeStage: adult; **Location:** country: Brazil; stateProvince: Piauí; municipality: Piracuruca; locality: Parque Nacional de Sete Cidades, Olha d'água dos Milagres; maximumElevationInMeters: 180; verbatimCoordinates: 4°5'31.8"S, 41°40'48.2"W; **Identification:** identifiedBy: Inês Corrêa Gonçalves; **Event:** samplingProtocol: Pennsylvania light trap; verbatimEventDate: 9.ii.13; **Record Level:** institutionCode: DZRJ; basisOfRecord: PreservedSpecimen
- **Type status:** Other material. **Occurrence:** recordedBy: Santos, A.P.M.; individualCount: 1; sex: female; lifeStage: immature; **Location:** country: Brazil; stateProvince: Piauí; municipality: Piracuruca; locality: Parque Nacional de Sete Cidades, Cachoeira do Riachão; maximumElevationInMeters: 171; verbatimCoordinates: 4°6'28"S, 41°40'13"W; **Identification:** identifiedBy: Inês Corrêa Gonçalves; **Event:** samplingProtocol: White sheet light trap; verbatimEventDate: 9.ii.13; **Record Level:** institutionCode: DZRJ; basisOfRecord: PreservedSpecimen

##### Distribution

Brazil: PA, AM, PI!.

##### Notes

New species record for Northeastern Brazil.

#### Leptophlebiidae

##### Notes

New family record for PI.

#### Farrodes

Peters, 1971

##### Notes

New genus record for PI.

#### Farrodes tepui

Domínguez, Molineri & Peters, 1996

##### Materials

- **Type status:** Other material. **Occurrence:** recordedBy: Takiya, D.M.; individualCount: 3; sex: male; lifeStage: adult; **Location:** country: Brazil; stateProvince: Piauí; municipality: Piracuruca; locality: Parque Nacional de Sete Cidades, Cachoeira do Riachão; maximumElevationInMeters: 171; verbatimCoordinates: 4°6'28"S, 41°40'13"W; **Identification:** identifiedBy: Inês Corrêa Gonçalves; **Event:** samplingProtocol: Pennsylvania light trap; verbatimEventDate: 19.iv.12; **Record Level:** institutionCode: DZRJ; basisOfRecord: PreservedSpecimen
- **Type status:** Other material. **Occurrence:** recordedBy: Takiya, D.M.; individualCount: 8; sex: female; lifeStage: adult; **Location:** country: Brazil; stateProvince: Piauí; municipality: Piracuruca; locality: Parque Nacional de Sete Cidades, Cachoeira do Riachão; maximumElevationInMeters: 171; verbatimCoordinates: 4°6'28"S, 41°40'13"W; **Identification:** identifiedBy: Inês Corrêa Gonçalves; **Event:** samplingProtocol: Pennsylvania light trap; verbatimEventDate: 19.iv.12; **Record Level:** institutionCode: DZRJ; basisOfRecord: PreservedSpecimen
- **Type status:** Other material. **Occurrence:** recordedBy: Rafael, J.A. | Limeira-de-Oliveira, F. | Takiya, D.M. | et al.; individualCount: 2; sex: male; lifeStage: adult; **Location:** country: Brazil; stateProvince: Piauí; municipality: Piracuruca; locality: Parque Nacional de Sete Cidades, Riacho da Piedade; maximumElevationInMeters: 169; verbatimCoordinates: 4°6'34"S, 41°43'39"W; **Identification:** identifiedBy: Inês Corrêa Gonçalves; **Event:** samplingProtocol: Malaise intercept trap; verbatimEventDate: 21.iv.12; **Record Level:** institutionCode: DZRJ; basisOfRecord: PreservedSpecimen

##### Distribution

Venezuela. Brazil: PI!, CE!, PE.

##### Notes

New species record for PI.

#### Fittkaulus

Savage & Peters, 1978

##### Notes

New genus record for PI.

#### Fittkaulus cururuensis

Savage, 1986

##### Materials

- **Type status:** Other material. **Occurrence:** recordedBy: Takiya, D.M.; individualCount: 1; sex: male; lifeStage: adult; **Location:** country: Brazil; stateProvince: Piauí; municipality: Piracuruca; locality: Parque Nacional de Sete Cidades, Riacho da Piedade; maximumElevationInMeters: 169; verbatimCoordinates: 4°6'34"S, 41°43'39"W; **Identification:** identifiedBy: Inês Corrêa Gonçalves; **Event:** samplingProtocol: Manual; verbatimEventDate: 18.iv.12; **Record Level:** institutionCode: DZRJ; basisOfRecord: PreservedSpecimen
- **Type status:** Other material. **Occurrence:** recordedBy: Takiya, D.M.; individualCount: 1; sex: male; lifeStage: adult; **Location:** country: Brazil; stateProvince: Piauí; municipality: Piracuruca; locality: Parque Nacional de Sete Cidades, Riacho da Piedade; maximumElevationInMeters: 169; verbatimCoordinates: 4°6'34"S, 41°43'39"W; **Identification:** identifiedBy: Inês Corrêa Gonçalves; **Event:** samplingProtocol: Pennsylvania light trap; verbatimEventDate: 19.iv.12; **Record Level:** institutionCode: DZRJ; basisOfRecord: PreservedSpecimen
- **Type status:** Other material. **Occurrence:** recordedBy: Takiya, D.M.; individualCount: 1; sex: female; lifeStage: adult; **Location:** country: Brazil; stateProvince: Piauí; municipality: Piracuruca; locality: Parque Nacional de Sete Cidades, Riacho da Piedade; maximumElevationInMeters: 169; verbatimCoordinates: 4°6'34"S, 41°43'39"W; **Identification:** identifiedBy: Inês Corrêa Gonçalves; **Event:** samplingProtocol: Pennsylvania light trap; verbatimEventDate: 19.iv.12; **Record Level:** institutionCode: DZRJ; basisOfRecord: PreservedSpecimen
- **Type status:** Other material. **Occurrence:** recordedBy: Takiya, D.M. | Rafael, J.A.; individualCount: 1; sex: male; lifeStage: adult; **Location:** country: Brazil; stateProvince: Piauí; municipality: Piracuruca; locality: Parque Nacional de Sete Cidades, Cachoeira do Riachão; maximumElevationInMeters: 171; verbatimCoordinates: 4°6'28"S, 41°40'13"W; **Identification:** identifiedBy: Inês Corrêa Gonçalves; **Event:** samplingProtocol: Pennsylvania light trap; verbatimEventDate: 20.iv.12; **Record Level:** institutionCode: DZRJ; basisOfRecord: PreservedSpecimen
- **Type status:** Other material. **Occurrence:** recordedBy: Rafael, J.A. | Limeira-de-Oliveira, F. | Takiya, D.M. | et al.; individualCount: 3; sex: female; lifeStage: adult; **Location:** country: Brazil; stateProvince: Piauí; municipality: Piracuruca; locality: Parque Nacional de Sete Cidades, Riacho da Piedade; maximumElevationInMeters: 169; verbatimCoordinates: 4°6'34"S, 41°43'39"W; **Identification:** identifiedBy: Inês Corrêa Gonçalves; **Event:** samplingProtocol: Malaise intercept trap; verbatimEventDate: 21.iv.12; **Record Level:** institutionCode: DZRJ; basisOfRecord: PreservedSpecimen
- **Type status:** Other material. **Occurrence:** recordedBy: Rafael, J.A. | Limeira-de-Oliveira, F. | Takiya, D.M. | et al.; individualCount: 2; sex: male; lifeStage: adult; **Location:** country: Brazil; stateProvince: Piauí; municipality: Piracuruca; locality: Parque Nacional de Sete Cidades, Riacho da Piedade; maximumElevationInMeters: 169; verbatimCoordinates: 4°6'34"S, 41°43'39"W; **Identification:** identifiedBy: Inês Corrêa Gonçalves; **Event:** samplingProtocol: Malaise intercept trap; verbatimEventDate: 21.iv.12; **Record Level:** institutionCode: DZRJ; basisOfRecord: PreservedSpecimen
- **Type status:** Other material. **Occurrence:** recordedBy: Takiya, D.M. | Rafael, J.A.; individualCount: 5; sex: female; lifeStage: adult; **Location:** country: Brazil; stateProvince: Piauí; municipality: Piracuruca; locality: Parque Nacional de Sete Cidades, Cachoeira do Riachão; maximumElevationInMeters: 171; verbatimCoordinates: 4°6'28"S, 41°40'13"W; **Identification:** identifiedBy: Inês Corrêa Gonçalves; **Event:** samplingProtocol: Pennsylvania light trap; verbatimEventDate: 20.iv.12; **Record Level:** institutionCode: DZRJ; basisOfRecord: PreservedSpecimen

##### Distribution

Brazil: PA, PI!, PE, MT, ES.

##### Notes

New species record for PI.

#### Miroculis

Edmunds, 1963

##### Notes

New genus record for PI.

#### Miroculis sp. 1

##### Materials

- **Type status:** Other material. **Occurrence:** recordedBy: Takiya, D.M.; individualCount: 2; sex: male; lifeStage: adult; **Location:** country: Brazil; stateProvince: Piauí; municipality: Piracuruca; locality: Parque Nacional de Sete Cidades, Cachoeira do Riachão; maximumElevationInMeters: 171; verbatimCoordinates: 4°6'28"S, 41°40'13"W; **Identification:** identifiedBy: Inês Corrêa Gonçalves; **Event:** samplingProtocol: Pennsylvania light trap; verbatimEventDate: 19.iv.12; **Record Level:** institutionCode: DZRJ; basisOfRecord: PreservedSpecimen
- **Type status:** Other material. **Occurrence:** recordedBy: Takiya, D.M. | Rafael, J.A.; individualCount: 1; sex: male; lifeStage: adult; **Location:** country: Brazil; stateProvince: Piauí; municipality: Piracuruca; locality: Parque Nacional de Sete Cidades, Cachoeira do Riachão; maximumElevationInMeters: 171; verbatimCoordinates: 4°6'28"S, 41°40'13"W; **Identification:** identifiedBy: Inês Corrêa Gonçalves; **Event:** samplingProtocol: Pennsylvania light trap; verbatimEventDate: 20.iv.12; **Record Level:** institutionCode: DZRJ; basisOfRecord: PreservedSpecimen
- **Type status:** Other material. **Occurrence:** recordedBy: Rafael, J.A. | Limeira-de-Oliveira, F. | Takiya, D.M. | et al.; individualCount: 3; sex: female; lifeStage: adult; **Location:** country: Brazil; stateProvince: Piauí; municipality: Piracuruca; locality: Parque Nacional de Sete Cidades, Riacho da Piedade; maximumElevationInMeters: 169; verbatimCoordinates: 4°6'34"S, 41°43'39"W; **Identification:** identifiedBy: Inês Corrêa Gonçalves; **Event:** samplingProtocol: Malaise intercept trap; verbatimEventDate: 21.iv.12; **Record Level:** institutionCode: DZRJ; basisOfRecord: PreservedSpecimen

##### Notes

Undescribed species.

#### Simothraulopsis

Demoulin, 1966

##### Notes

New genus record for PI.

#### Simothraulopsis demerara

(Traver, 1947)

##### Materials

- **Type status:** Other material. **Occurrence:** recordedBy: Takiya, D.M.; individualCount: 3; sex: male; lifeStage: adult; **Location:** country: Brazil; stateProvince: Piauí; municipality: Piracuruca; locality: Parque Nacional de Sete Cidades, Cachoeira do Riachão; maximumElevationInMeters: 171; verbatimCoordinates: 4°6'28"S, 41°40'13"W; **Identification:** identifiedBy: Inês Corrêa Gonçalves; **Event:** samplingProtocol: Pennsylvania light trap; verbatimEventDate: 18.iv.12; **Record Level:** institutionCode: DZRJ; basisOfRecord: PreservedSpecimen
- **Type status:** Other material. **Occurrence:** recordedBy: Takiya, D.M.; individualCount: 2; sex: female; lifeStage: adult; **Location:** country: Brazil; stateProvince: Piauí; municipality: Piracuruca; locality: Parque Nacional de Sete Cidades, Cachoeira do Riachão; maximumElevationInMeters: 171; verbatimCoordinates: 4°6'28"S, 41°40'13"W; **Identification:** identifiedBy: Inês Corrêa Gonçalves; **Event:** samplingProtocol: Pennsylvania light trap; verbatimEventDate: 18.iv.12; **Record Level:** institutionCode: DZRJ; basisOfRecord: PreservedSpecimen
- **Type status:** Other material. **Occurrence:** recordedBy: Takiya, D.M.; individualCount: 1; sex: male; lifeStage: adult; **Location:** country: Brazil; stateProvince: Piauí; municipality: Piracuruca; locality: Parque Nacional de Sete Cidades, Cachoeira do Riachão; maximumElevationInMeters: 171; verbatimCoordinates: 4°6'28"S, 41°40'13"W; **Identification:** identifiedBy: Inês Corrêa Gonçalves; **Event:** samplingProtocol: Pennsylvania light trap; verbatimEventDate: 19.iv.12; **Record Level:** institutionCode: DZRJ; basisOfRecord: PreservedSpecimen
- **Type status:** Other material. **Occurrence:** recordedBy: Takiya, D.M.; individualCount: 2; sex: female; lifeStage: adult; **Location:** country: Brazil; stateProvince: Piauí; municipality: Piracuruca; locality: Parque Nacional de Sete Cidades, Cachoeira do Riachão; maximumElevationInMeters: 171; verbatimCoordinates: 4°6'28"S, 41°40'13"W; **Identification:** identifiedBy: Inês Corrêa Gonçalves; **Event:** samplingProtocol: Pennsylvania light trap; verbatimEventDate: 19.iv.12; **Record Level:** institutionCode: DZRJ; basisOfRecord: PreservedSpecimen
- **Type status:** Other material. **Occurrence:** recordedBy: Takiya, D.M.; individualCount: 1; sex: female; lifeStage: adult; **Location:** country: Brazil; stateProvince: Piauí; municipality: Piracuruca; locality: Parque Nacional de Sete Cidades, Riacho da Piedade; maximumElevationInMeters: 169; verbatimCoordinates: 4°6'34"S, 41°43'39"W; **Identification:** identifiedBy: Inês Corrêa Gonçalves; **Event:** samplingProtocol: Pennsylvania light trap; verbatimEventDate: 19.iv.12; **Record Level:** institutionCode: DZRJ; basisOfRecord: PreservedSpecimen
- **Type status:** Other material. **Occurrence:** recordedBy: Takiya, D.M. | Rafael, J.A.; individualCount: 3; sex: male; lifeStage: adult; **Location:** country: Brazil; stateProvince: Piauí; municipality: Piracuruca; locality: Parque Nacional de Sete Cidades, Cachoeira do Riachão; maximumElevationInMeters: 171; verbatimCoordinates: 4°6'28"S, 41°40'13"W; **Identification:** identifiedBy: Inês Corrêa Gonçalves; **Event:** samplingProtocol: Pennsylvania light trap; verbatimEventDate: 20.iv.12; **Record Level:** institutionCode: DZRJ; basisOfRecord: PreservedSpecimen
- **Type status:** Other material. **Occurrence:** recordedBy: Takiya, D.M.; individualCount: 1; sex: male; lifeStage: adult; **Location:** country: Brazil; stateProvince: Piauí; municipality: Piracuruca; locality: Parque Nacional de Sete Cidades, Riacho da Piedade; maximumElevationInMeters: 169; verbatimCoordinates: 4°6'34"S, 41°43'39"W; **Identification:** identifiedBy: Inês Corrêa Gonçalves; **Event:** samplingProtocol: Pennsylvania light trap; verbatimEventDate: 22.iv.12; **Record Level:** institutionCode: DZRJ; basisOfRecord: PreservedSpecimen
- **Type status:** Other material. **Occurrence:** recordedBy: Santos, A.P.M. | Takiya, D.M.; individualCount: 1; sex: male; lifeStage: adult; **Location:** country: Brazil; stateProvince: Piauí; municipality: Piracuruca; locality: Parque Nacional de Sete Cidades, Olha d'água dos Milagres; maximumElevationInMeters: 180; verbatimCoordinates: 4°5'31.8"S, 41°40'48.2"W; **Identification:** identifiedBy: Inês Corrêa Gonçalves; **Event:** samplingProtocol: White sheet light trap; verbatimEventDate: 9.ii.13; **Record Level:** institutionCode: DZRJ; basisOfRecord: PreservedSpecimen
- **Type status:** Other material. **Occurrence:** recordedBy: Santos, A.P.M. | Takiya, D.M.; individualCount: 1; sex: female; lifeStage: adult; **Location:** country: Brazil; stateProvince: Piauí; municipality: Piracuruca; locality: Parque Nacional de Sete Cidades, Poço do Bananeira; maximumElevationInMeters: 158; verbatimCoordinates: 4°5'55.8"S, 41°40'33.8"W; **Identification:** identifiedBy: Inês Corrêa Gonçalves; **Event:** samplingProtocol: Pennsylvania light trap; verbatimEventDate: 10.ii.13; **Record Level:** institutionCode: DZRJ; basisOfRecord: PreservedSpecimen
- **Type status:** Other material. **Occurrence:** recordedBy: Santos, A.P.M. | Takiya, D.M.; individualCount: 6; sex: female; lifeStage: adult; **Location:** country: Brazil; stateProvince: Piauí; municipality: Piracuruca; locality: Parque Nacional de Sete Cidades, Poço do Bananeira; maximumElevationInMeters: 158; verbatimCoordinates: 4°5'55.8"S, 41°40'33.8"W; **Identification:** identifiedBy: Inês Corrêa Gonçalves; **Event:** samplingProtocol: Pennsylvania light trap; verbatimEventDate: 11.ii.13; **Record Level:** institutionCode: DZRJ; basisOfRecord: PreservedSpecimen

##### Distribution

Colombia. Venezuela. Guyana. Suriname. French Guiana. Brazil: PA, AM, PI!, PE, ES. Ecuador. Bolivia. Argentina.

##### Notes

New species record for PI.

#### Thraulodes

Ulmer, 1920

##### Notes

New genus record for PI.

#### Thraulodes sp. 1

##### Materials

- **Type status:** Other material. **Occurrence:** recordedBy: Rafael, J.A. | Limeira-de-Oliveira, F. | Takiya, D.M. | et al.; individualCount: 1; sex: female; lifeStage: adult; **Location:** country: Brazil; stateProvince: Piauí; municipality: Piracuruca; locality: Parque Nacional de Sete Cidades, Riacho da Bananeira; maximumElevationInMeters: 189; verbatimCoordinates: 4°5'59"S, 41°40'48"W; **Identification:** identifiedBy: Inês Corrêa Gonçalves; **Event:** samplingProtocol: Malaise intercept trap; verbatimEventDate: 19.iv.12; **Record Level:** institutionCode: DZRJ; basisOfRecord: PreservedSpecimen

#### Ulmeritoides

Traver, 1959

##### Notes

New genus record for PI.

#### Ulmeritoides flavopedes

(Spieth, 1943)

##### Materials

- **Type status:** Other material. **Occurrence:** recordedBy: Santos, A.P.M. | Takiya, D.M.; individualCount: 1; sex: female; lifeStage: adult; **Location:** country: Brazil; stateProvince: Piauí; municipality: Piracuruca; locality: Parque Nacional de Sete Cidades, Poço do Bananeira; maximumElevationInMeters: 158; verbatimCoordinates: 4°5'55.8"S, 41°40'33.8"W; **Identification:** identifiedBy: Inês Corrêa Gonçalves; **Event:** samplingProtocol: Pennsylvania light trap; verbatimEventDate: 10.ii.13; **Record Level:** institutionCode: DZRJ; basisOfRecord: PreservedSpecimen
- **Type status:** Other material. **Occurrence:** recordedBy: Santos, A.P.M. | Takiya, D.M.; individualCount: 1; sex: male; lifeStage: adult; **Location:** country: Brazil; stateProvince: Piauí; municipality: Piracuruca; locality: Parque Nacional de Sete Cidades, Poço do Bananeira; maximumElevationInMeters: 158; verbatimCoordinates: 4°5'55.8"S, 41°40'33.8"W; **Identification:** identifiedBy: Inês Corrêa Gonçalves; **Event:** samplingProtocol: Pennsylvania light trap; verbatimEventDate: 11.ii.13; **Record Level:** institutionCode: DZRJ; basisOfRecord: PreservedSpecimen
- **Type status:** Other material. **Occurrence:** recordedBy: Santos, A.P.M. | Takiya, D.M.; individualCount: 1; sex: female; lifeStage: adult; **Location:** country: Brazil; stateProvince: Piauí; municipality: Piracuruca; locality: Parque Nacional de Sete Cidades, Poço do Bananeira; maximumElevationInMeters: 158; verbatimCoordinates: 4°5'55.8"S, 41°40'33.8"W; **Identification:** identifiedBy: Inês Corrêa Gonçalves; **Event:** samplingProtocol: Pennsylvania light trap; verbatimEventDate: 11.ii.13; **Record Level:** institutionCode: DZRJ; basisOfRecord: PreservedSpecimen
- **Type status:** Other material. **Occurrence:** recordedBy: Santos, A.P.M. | Takiya, D.M.; individualCount: 1; sex: male; lifeStage: adult; **Location:** country: Brazil; stateProvince: Piauí; municipality: Piracuruca; locality: Parque Nacional de Sete Cidades, Poço do Bananeira; maximumElevationInMeters: 158; verbatimCoordinates: 4°5'55.8"S, 41°40'33.8"W; **Identification:** identifiedBy: Inês Corrêa Gonçalves; **Event:** samplingProtocol: Pennsylvania light trap; verbatimEventDate: 9.ii.13; **Record Level:** institutionCode: DZRJ; basisOfRecord: PreservedSpecimen
- **Type status:** Other material. **Occurrence:** recordedBy: Takiya, D.M.; individualCount: 7; sex: male; lifeStage: adult; **Location:** country: Brazil; stateProvince: Piauí; municipality: Piracuruca; locality: Parque Nacional de Sete Cidades, Cachoeira do Riachão; maximumElevationInMeters: 171; verbatimCoordinates: 4°6'28"S, 41°40'13"W; **Identification:** identifiedBy: Inês Corrêa Gonçalves; **Event:** samplingProtocol: Pennsylvania light trap; verbatimEventDate: 19.iv.12; **Record Level:** institutionCode: DZRJ; basisOfRecord: PreservedSpecimen
- **Type status:** Other material. **Occurrence:** recordedBy: Takiya, D.M.; individualCount: 3; sex: female; lifeStage: adult; **Location:** country: Brazil; stateProvince: Piauí; municipality: Piracuruca; locality: Parque Nacional de Sete Cidades, Cachoeira do Riachão; maximumElevationInMeters: 171; verbatimCoordinates: 4°6'28"S, 41°40'13"W; **Identification:** identifiedBy: Inês Corrêa Gonçalves; **Event:** samplingProtocol: Pennsylvania light trap; verbatimEventDate: 19.iv.12; **Record Level:** institutionCode: DZRJ; basisOfRecord: PreservedSpecimen
- **Type status:** Other material. **Occurrence:** recordedBy: Takiya, D.M. | Rafael, J.A.; individualCount: 14; sex: male; lifeStage: adult; **Location:** country: Brazil; stateProvince: Piauí; municipality: Piracuruca; locality: Parque Nacional de Sete Cidades, Cachoeira do Riachão; maximumElevationInMeters: 171; verbatimCoordinates: 4°6'28"S, 41°40'13"W; **Identification:** identifiedBy: Inês Corrêa Gonçalves; **Event:** samplingProtocol: Pennsylvania light trap; verbatimEventDate: 20.iv.12; **Record Level:** institutionCode: DZRJ; basisOfRecord: PreservedSpecimen
- **Type status:** Other material. **Occurrence:** recordedBy: Takiya, D.M. | Rafael, J.A.; individualCount: 2; sex: female; lifeStage: adult; **Location:** country: Brazil; stateProvince: Piauí; municipality: Piracuruca; locality: Parque Nacional de Sete Cidades, Cachoeira do Riachão; maximumElevationInMeters: 171; verbatimCoordinates: 4°6'28"S, 41°40'13"W; **Identification:** identifiedBy: Inês Corrêa Gonçalves; **Event:** samplingProtocol: Pennsylvania light trap; verbatimEventDate: 20.iv.12; **Record Level:** institutionCode: DZRJ; basisOfRecord: PreservedSpecimen

##### Distribution

Suriname. Brazil: RR, PI!, PE, MT.

##### Notes

New species record for PI.

#### Polymitarcyidae

#### Campsurus

Eaton, 1868

#### Campsurus truncatus

Ulmer, 1920

##### Materials

- **Type status:** Other material. **Occurrence:** recordedBy: Takiya, D.M.; individualCount: 1; sex: female; lifeStage: adult; **Location:** country: Brazil; stateProvince: Piauí; municipality: Piracuruca; locality: Parque Nacional de Sete Cidades, Cachoeira do Riachão; maximumElevationInMeters: 171; verbatimCoordinates: 4°6'28"S, 41°40'13"W; **Identification:** identifiedBy: Inês Corrêa Gonçalves; **Event:** samplingProtocol: Pennsylvania light trap; verbatimEventDate: 18.iv.12; **Record Level:** institutionCode: DZRJ; basisOfRecord: PreservedSpecimen
- **Type status:** Other material. **Occurrence:** recordedBy: Takiya, D.M.; individualCount: 1; sex: male; lifeStage: adult; **Location:** country: Brazil; stateProvince: Piauí; municipality: Piracuruca; locality: Parque Nacional de Sete Cidades, Cachoeira do Riachão; maximumElevationInMeters: 171; verbatimCoordinates: 4°6'28"S, 41°40'13"W; **Identification:** identifiedBy: Inês Corrêa Gonçalves; **Event:** samplingProtocol: Pennsylvania light trap; verbatimEventDate: 18.iv.12; **Record Level:** institutionCode: DZRJ; basisOfRecord: PreservedSpecimen
- **Type status:** Other material. **Occurrence:** recordedBy: Takiya, D.M.; individualCount: 2; sex: female; lifeStage: adult; **Location:** country: Brazil; stateProvince: Piauí; municipality: Piracuruca; locality: Parque Nacional de Sete Cidades, Cachoeira do Riachão; maximumElevationInMeters: 171; verbatimCoordinates: 4°6'28"S, 41°40'13"W; **Identification:** identifiedBy: Inês Corrêa Gonçalves; **Event:** samplingProtocol: Pennsylvania light trap; verbatimEventDate: 19.iv.12; **Record Level:** institutionCode: DZRJ; basisOfRecord: PreservedSpecimen
- **Type status:** Other material. **Occurrence:** recordedBy: Takiya, D.M.; individualCount: 5; sex: female; lifeStage: adult; **Location:** country: Brazil; stateProvince: Piauí; municipality: Piracuruca; locality: Parque Nacional de Sete Cidades, Olho d'água Piscina do Bacuri; maximumElevationInMeters: 171; verbatimCoordinates: 4°6'1.2"S, 41°42'38.8"W; **Identification:** identifiedBy: Inês Corrêa Gonçalves; **Event:** samplingProtocol: White sheet light trap; verbatimEventDate: 20.iv.12; **Record Level:** institutionCode: DZRJ; basisOfRecord: PreservedSpecimen
- **Type status:** Other material. **Occurrence:** recordedBy: Santos, A.P.M. | Takiya, D.M.; individualCount: 2; sex: female; lifeStage: adult; **Location:** country: Brazil; stateProvince: Piauí; municipality: Piracuruca; locality: Parque Nacional de Sete Cidades, Cachoeira do Riachão; maximumElevationInMeters: 171; verbatimCoordinates: 4°6'28"S, 41°40'13"W; **Identification:** identifiedBy: Inês Corrêa Gonçalves; **Event:** samplingProtocol: Pennsylvania light trap; verbatimEventDate: 12.ii.13; **Record Level:** institutionCode: DZRJ; basisOfRecord: PreservedSpecimen
- **Type status:** Other material. **Occurrence:** recordedBy: Santos, A.P.M. | Takiya, D.M.; individualCount: 1; sex: female; lifeStage: adult; **Location:** country: Brazil; stateProvince: Piauí; municipality: Piracuruca; locality: Parque Nacional de Sete Cidades, Alojamento; maximumElevationInMeters: 193; verbatimCoordinates: 4°5'57"S, 41°42'34"W; **Identification:** identifiedBy: Inês Corrêa Gonçalves; **Event:** samplingProtocol: White sheet light trap; verbatimEventDate: 8.ii.13; **Record Level:** institutionCode: DZRJ; basisOfRecord: PreservedSpecimen
- **Type status:** Other material. **Occurrence:** recordedBy: Santos, A.P.M. | Takiya, D.M.; individualCount: 3; sex: female; lifeStage: adult; **Location:** country: Brazil; stateProvince: Piauí; municipality: Piracuruca; locality: Parque Nacional de Sete Cidades, Cachoeira do Riachão; maximumElevationInMeters: 171; verbatimCoordinates: 4°6'28"S, 41°40'13"W; **Identification:** identifiedBy: Inês Corrêa Gonçalves; **Event:** samplingProtocol: White sheet light trap; verbatimEventDate: 8.ii.13; **Record Level:** institutionCode: DZRJ; basisOfRecord: PreservedSpecimen
- **Type status:** Other material. **Occurrence:** recordedBy: Santos, A.P.M. | Takiya, D.M.; individualCount: 2; sex: female; lifeStage: adult; **Location:** country: Brazil; stateProvince: Piauí; municipality: Piracuruca; locality: Parque Nacional de Sete Cidades, Cachoeira do Riachão; maximumElevationInMeters: 171; verbatimCoordinates: 4°6'28"S, 41°40'13"W; **Identification:** identifiedBy: Inês Corrêa Gonçalves; **Event:** samplingProtocol: White sheet light trap; verbatimEventDate: 8.ii.13; **Record Level:** institutionCode: DZRJ; basisOfRecord: PreservedSpecimen
- **Type status:** Other material. **Occurrence:** recordedBy: Santos, A.P.M. | Takiya, D.M.; individualCount: 1; sex: female; lifeStage: adult; **Location:** country: Brazil; stateProvince: Piauí; municipality: Piracuruca; locality: Parque Nacional de Sete Cidades, Olho d'água Piscina do Bacuri; maximumElevationInMeters: 171; verbatimCoordinates: 4°6'1.2"S, 41°42'38.8"W; **Identification:** identifiedBy: Inês Corrêa Gonçalves; **Event:** samplingProtocol: White sheet light trap; verbatimEventDate: 8.ii.13; **Record Level:** institutionCode: DZRJ; basisOfRecord: PreservedSpecimen
- **Type status:** Other material. **Occurrence:** recordedBy: Santos, A.P.M. | Takiya, D.M.; individualCount: 2; sex: female; lifeStage: adult; **Location:** country: Brazil; stateProvince: Piauí; municipality: Piracuruca; locality: Parque Nacional de Sete Cidades, Poço do Bananeira; maximumElevationInMeters: 158; verbatimCoordinates: 4°5'55.8"S, 41°40'33.8"W; **Identification:** identifiedBy: Inês Corrêa Gonçalves; **Event:** samplingProtocol: Pennsylvania light trap; verbatimEventDate: 9.ii.13; **Record Level:** institutionCode: DZRJ; basisOfRecord: PreservedSpecimen

##### Distribution

Colombia. Brazil: PI!, PE, ES, RJ. Peru. Bolivia.

##### Notes

New species record for PI.

#### Hemiptera

#### Gerromorpha

#### Gerridae

#### Neogerris

Matsumura, 1913

##### Notes

Genus firstly recorded from PI in Cordeiro and Moreira 2015.

#### Neogerris lubricus

(White, 1879)

##### Materials

- **Type status:** Other material. **Occurrence:** recordedBy: Santos, A.P.M. | Takiya, D.M.; individualCount: 1; sex: male; lifeStage: adult; **Location:** country: Brazil; stateProvince: Piauí; municipality: Piracuruca; locality: Parque Nacional de Sete Cidades, Cachoeira do Riachão; maximumElevationInMeters: 171; verbatimCoordinates: 4°6'28"S, 41°40'13"W; **Identification:** identifiedBy: Isabelle da R. S. Cordeiro; **Event:** samplingProtocol: Manual; verbatimEventDate: 12.ii.13; **Record Level:** institutionCode: DZRJ; basisOfRecord: PreservedSpecimen

##### Distribution

Panama. Trinidad & Tobago. Colombia. Guyana. Suriname. Brazil: AP, PA, AM, PI, MT, RO, BA, MG, MS, SP, RJ. Ecuador. Peru. Bolivia. Paraguay. Argentina.

##### Notes

Species firstly recorded from PI in Cordeiro and Moreira 2015.

#### Mesoveliidae

##### Notes

New family record for PI.

#### Mesovelia

Mulsant & Rey,1852

##### Notes

New genus record for PI.

#### Mesovelia sp. 1

##### Materials

- **Type status:** Other material. **Occurrence:** recordedBy: Santos, A.P.M. | Takiya, D.M.; individualCount: 1; lifeStage: immature; **Location:** country: Brazil; stateProvince: Piauí; municipality: Piracuruca; locality: Parque Nacional de Sete Cidades, Cachoeira do Riachão; maximumElevationInMeters: 171; verbatimCoordinates: 4°6'28"S, 41°40'13"W; **Identification:** identifiedBy: Isabelle da R. S. Cordeiro; **Event:** samplingProtocol: Manual; verbatimEventDate: 9.ii.13; **Record Level:** institutionCode: DZRJ; basisOfRecord: PreservedSpecimen

#### Veliidae

##### Notes

Family firstly recorded from PI in Cordeiro and Moreira 2015.

#### Microvelia

Westwood, 1834

##### Notes

Genus firstly recorded from PI in Cordeiro and Moreira 2015.

#### Microvelia ayacuchana

Drake & Maldonado Capriles 1952

##### Materials

- **Type status:** Other material. **Occurrence:** recordedBy: Santos, A.P.M. | Takiya, D.M.; individualCount: 1; sex: male; lifeStage: adult; **Location:** country: Brazil; stateProvince: Piauí; municipality: Piracuruca; locality: Parque Nacional de Sete Cidades, Cachoeira do Riachão; maximumElevationInMeters: 171; verbatimCoordinates: 4°6'28"S, 41°40'13"W; **Identification:** identifiedBy: Isabelle da R. S. Cordeiro; **Event:** samplingProtocol: Manual; verbatimEventDate: 9.ii.13; **Record Level:** institutionCode: DZRJ; basisOfRecord: PreservedSpecimen
- **Type status:** Other material. **Occurrence:** recordedBy: Santos, A.P.M. | Takiya, D.M.; individualCount: 1; sex: female; lifeStage: adult; **Location:** country: Brazil; stateProvince: Piauí; municipality: Piracuruca; locality: Parque Nacional de Sete Cidades, Cachoeira do Riachão; maximumElevationInMeters: 171; verbatimCoordinates: 4°6'28"S, 41°40'13"W; **Identification:** identifiedBy: Isabelle da R. S. Cordeiro; **Event:** samplingProtocol: Manual; verbatimEventDate: 9.ii.13; **Record Level:** institutionCode: DZRJ; basisOfRecord: PreservedSpecimen

##### Distribution

Venezuela. Guyana. Suriname. Brazil: PA, PI, ES.

##### Notes

Species firstly recorded from Northeastern Brazil in Cordeiro and Moreira 2015​.

#### Microvelia pulchella

Westwood, 1834

##### Materials

- **Type status:** Other material. **Occurrence:** recordedBy: Santos, A.P.M. | Takiya, D.M.; individualCount: 5; sex: male; lifeStage: adult; **Location:** country: Brazil; stateProvince: Piauí; municipality: Piracuruca; locality: Parque Nacional de Sete Cidades, Rio da Bananeira; maximumElevationInMeters: 189; verbatimCoordinates: 4°5'59"S, 41°40'48"W; **Identification:** identifiedBy: Isabelle da R. S. Cordeiro; **Event:** samplingProtocol: Manual; verbatimEventDate: 8.ii.13; **Record Level:** institutionCode: DZRJ; basisOfRecord: PreservedSpecimen
- **Type status:** Other material. **Occurrence:** recordedBy: Santos, A.P.M. | Takiya, D.M.; individualCount: 2; sex: female; lifeStage: adult; **Location:** country: Brazil; stateProvince: Piauí; municipality: Piracuruca; locality: Parque Nacional de Sete Cidades, Rio da Bananeira; maximumElevationInMeters: 189; verbatimCoordinates: 4°5'59"S, 41°40'48"W; **Identification:** identifiedBy: Isabelle da R. S. Cordeiro; **Event:** samplingProtocol: Manual; verbatimEventDate: 8.ii.13; **Record Level:** institutionCode: DZRJ; basisOfRecord: PreservedSpecimen

##### Distribution

Canada. USA. Mexico. Guatemala. Costa Rica. Panama. Bahamas. Cuba. Dominican Republic. Cayman Islands. Jamaica. Puerto Rico. U.S. Virgin Islands. Anguilla. St. Martin. Saba. St. Kitts & Nevis. Guadeloupe. Martinique. Aruba. St. Vincent & Grenadines. Barbados. Curaçao. Klein Curaçao. Bonaire. Klein Bonaire. Grenada. Trinidad and Tobago. Colombia. Venezuela. Brazil: PA, AM, MA, PI, PE, BA, AL, MG, MS, ES, SP, RJ, SC. Ecuador. Peru. Argentina.

##### Notes

Species firstly recorded from PI in Cordeiro and Moreira 2015​.

#### Platyvelia

Polhemus & Polhemus, 1993

##### Notes

Genus firstly recorded from PI in Cordeiro and Moreira 2015.

#### Platyvelia brachialis

(Stål, 1860)

##### Materials

- **Type status:** Other material. **Occurrence:** recordedBy: Santos, A.P.M. | Takiya, D.M.; individualCount: 1; sex: female; lifeStage: adult; **Location:** country: Brazil; stateProvince: Piauí; municipality: Piracuruca; locality: Parque Nacional de Sete Cidades, Olha d'água dos Milagres; maximumElevationInMeters: 180; verbatimCoordinates: 4°5'31.8"S, 41°40'48.2"W; **Identification:** identifiedBy: Isabelle da R. S. Cordeiro; **Event:** samplingProtocol: Manual; verbatimEventDate: 11.ii.13; **Record Level:** institutionCode: DZRJ; basisOfRecord: PreservedSpecimen

##### Distribution

USA. Mexico. Guatemala. Nicaragua. Costa Rica. Panama. Cuba. Dominican Republic. Jamaica. Grenada. Trinidad and Tobago. Suriname. Brazil: PI, PE, MT, GO, MG, MS, ES, RJ, SC. Peru. Argentina.

##### Notes

Species firstly recorded from PI in Cordeiro and Moreira 2015​.

#### Nepomorpha

#### Belostomatidae

#### Belostoma

Latreille, 1807

#### Belostoma sp. 1*

##### Materials

- **Type status:** Other material. **Occurrence:** recordedBy: Santos, A.P.M. | Takiya, D.M.; individualCount: 1; sex: female; lifeStage: adult; **Location:** country: Brazil; stateProvince: Piauí; municipality: Piracuruca; locality: Parque Nacional de Sete Cidades, Cachoeira do Riachão; maximumElevationInMeters: 171; verbatimCoordinates: 4°6'28"S, 41°40'13"W; **Identification:** identifiedBy: Julianna Freires Barbosa; **Event:** samplingProtocol: Manual; verbatimEventDate: 12.ii.13; **Record Level:** institutionCode: DZRJ; basisOfRecord: PreservedSpecimen

#### Corixidae

##### Notes

New family record for PI.

#### Tenagobia

Bergroth, 1899

##### Notes

New genus record for PI.

#### Tenagobia (Incertagobia) sp.

##### Materials

- **Type status:** Other material. **Occurrence:** recordedBy: Santos, A.P.M. | Takiya, D.M.; individualCount: 1; sex: male; lifeStage: adult; **Location:** country: Brazil; stateProvince: Piauí; municipality: Piracuruca; locality: Parque Nacional de Sete Cidades, Cachoeira do Riachão; maximumElevationInMeters: 171; verbatimCoordinates: 4°6'28"S, 41°40'13"W; **Identification:** identifiedBy: Isabelle da R. S. Cordeiro; **Event:** samplingProtocol: Manual; verbatimEventDate: 9.ii.13; **Record Level:** institutionCode: DZRJ; basisOfRecord: PreservedSpecimen

#### Gelastocoridae

##### Notes

New family record for PI.

#### Gelastocoris

Kirkaldy, 1897

##### Notes

New genus record for PI.

#### Gelastocoris sp. 1

##### Materials

- **Type status:** Other material. **Occurrence:** recordedBy: Santos, A.P.M. | Takiya, D.M.; individualCount: 1; sex: female; lifeStage: adult; **Location:** country: Brazil; stateProvince: Piauí; municipality: Piracuruca; locality: Parque Nacional de Sete Cidades, Riacho da Bananeira; maximumElevationInMeters: 189; verbatimCoordinates: 4°5'59"S, 41°40'48"W; **Identification:** identifiedBy: Julianna Freires Barbosa; **Event:** samplingProtocol: Manual; verbatimEventDate: 8.ii.13; **Record Level:** institutionCode: DZRJ; basisOfRecord: PreservedSpecimen
- **Type status:** Other material. **Occurrence:** recordedBy: Santos, A.P.M. | Takiya, D.M.; individualCount: 1; lifeStage: immature; **Location:** country: Brazil; stateProvince: Piauí; municipality: Piracuruca; locality: Parque Nacional de Sete Cidades, Olha d'água dos Milagres; maximumElevationInMeters: 180; verbatimCoordinates: 4°5'31.8"S, 41°40'48.2"W; **Identification:** identifiedBy: Isabelle da R. S. Cordeiro; **Event:** samplingProtocol: Manual; verbatimEventDate: 8.ii.13; **Record Level:** institutionCode: DZRJ; basisOfRecord: PreservedSpecimen

#### Pleidae

##### Notes

New family record for PI.

#### Neoplea

Esaki & china, 1928

##### Notes

New genus record for PI.

#### Neoplea sp. 1

##### Materials

- **Type status:** Other material. **Occurrence:** recordedBy: Takiya, D.M.; individualCount: 2; sex: male; lifeStage: adult; **Location:** country: Brazil; stateProvince: Piauí; municipality: Piracuruca; locality: Parque Nacional de Sete Cidades, Cachoeira do Riachão; maximumElevationInMeters: 171; verbatimCoordinates: 4°6'28"S, 41°40'13"W; **Identification:** identifiedBy: Julianna Freires Barbosa; **Event:** samplingProtocol: Pennsylvania light trap; verbatimEventDate: 19.iv.12; **Record Level:** institutionCode: DZRJ; basisOfRecord: PreservedSpecimen
- **Type status:** Other material. **Occurrence:** recordedBy: Takiya, D.M.; individualCount: 4; sex: female; lifeStage: adult; **Location:** country: Brazil; stateProvince: Piauí; municipality: Piracuruca; locality: Parque Nacional de Sete Cidades, Cachoeira do Riachão; maximumElevationInMeters: 171; verbatimCoordinates: 4°6'28"S, 41°40'13"W; **Identification:** identifiedBy: Julianna Freires Barbosa; **Event:** samplingProtocol: Pennsylvania light trap; verbatimEventDate: 19.iv.12; **Record Level:** institutionCode: DZRJ; basisOfRecord: PreservedSpecimen

#### Naucoridae

##### Notes

New family record for PI.

#### Pelocoris

Stal, 1876

##### Notes

New genus record for Northeastern Brazil.

#### Pelocoris sp. 1

##### Materials

- **Type status:** Other material. **Occurrence:** recordedBy: Santos, A.P.M. | Takiya, D.M.; individualCount: 1; sex: female; lifeStage: adult; **Location:** country: Brazil; stateProvince: Piauí; municipality: Piracuruca; locality: Parque Nacional de Sete Cidades, Cachoeira do Riachão; maximumElevationInMeters: 171; verbatimCoordinates: 4°6'28"S, 41°40'13"W; **Identification:** identifiedBy: Julianna Freires Barbosa; **Event:** samplingProtocol: Manual; verbatimEventDate: 9.ii.13; **Record Level:** institutionCode: DZRJ; basisOfRecord: PreservedSpecimen

#### Notonectidae

#### Buenoa

Kirkaldy, 1904

##### Notes

New genus record for PI.

#### Buenoa mutabilis

Truxal, 1953

##### Materials

- **Type status:** Other material. **Occurrence:** recordedBy: Santos, A.P.M. | Takiya, D.M.; individualCount: 1; sex: male; lifeStage: adult; **Location:** country: Brazil; stateProvince: Piauí; municipality: Piracuruca; locality: Parque Nacional de Sete Cidades, Riacho da Bananeira; maximumElevationInMeters: 189; verbatimCoordinates: 4°5'59"S, 41°40'48"W; **Identification:** identifiedBy: Julianna Freires Barbosa; **Event:** samplingProtocol: Manual; verbatimEventDate: 8.ii.13; **Record Level:** institutionCode: DZRJ; basisOfRecord: PreservedSpecimen

##### Distribution

Haiti. Venezuela. Guyana. Brazil: PI!, GO, MG. Peru. Paraguay.

##### Notes

New species record for Northeastern Brazil.

#### Buenoa pseudomutabilis

Barbosa, Ribeiro & Nessimian, 2010

##### Materials

- **Type status:** Other material. **Occurrence:** recordedBy: Takiya, D.M.; individualCount: 1; sex: male; lifeStage: adult; **Location:** country: Brazil; stateProvince: Piauí; municipality: Piracuruca; locality: Parque Nacional de Sete Cidades, Cachoeira do Riachão; maximumElevationInMeters: 171; verbatimCoordinates: 4°6'28"S, 41°40'13"W; **Identification:** identifiedBy: Julianna Freires Barbosa; **Event:** samplingProtocol: Pennsylvania light trap; verbatimEventDate: 18.iv.12; **Record Level:** institutionCode: DZRJ; basisOfRecord: PreservedSpecimen
- **Type status:** Other material. **Occurrence:** recordedBy: Takiya, D.M.; individualCount: 2; sex: female; lifeStage: adult; **Location:** country: Brazil; stateProvince: Piauí; municipality: Piracuruca; locality: Parque Nacional de Sete Cidades, Cachoeira do Riachão; maximumElevationInMeters: 171; verbatimCoordinates: 4°6'28"S, 41°40'13"W; **Identification:** identifiedBy: Julianna Freires Barbosa; **Event:** samplingProtocol: Pennsylvania light trap; verbatimEventDate: 18.iv.12; **Record Level:** institutionCode: DZRJ; basisOfRecord: PreservedSpecimen
- **Type status:** Other material. **Occurrence:** recordedBy: Santos, A.P.M. | Takiya, D.M.; individualCount: 2; sex: male; lifeStage: adult; **Location:** country: Brazil; stateProvince: Piauí; municipality: Piracuruca; locality: Parque Nacional de Sete Cidades, Cachoeira do Riachão; maximumElevationInMeters: 171; verbatimCoordinates: 4°6'28"S, 41°40'13"W; **Identification:** identifiedBy: Julianna Freires Barbosa; **Event:** samplingProtocol: Manual; verbatimEventDate: 9.ii.13; **Record Level:** institutionCode: DZRJ; basisOfRecord: PreservedSpecimen
- **Type status:** Other material. **Occurrence:** recordedBy: Santos, A.P.M. | Takiya, D.M.; individualCount: 3; sex: female; lifeStage: adult; **Location:** country: Brazil; stateProvince: Piauí; municipality: Piracuruca; locality: Parque Nacional de Sete Cidades, Cachoeira do Riachão; maximumElevationInMeters: 171; verbatimCoordinates: 4°6'28"S, 41°40'13"W; **Identification:** identifiedBy: Julianna Freires Barbosa; **Event:** samplingProtocol: Manual; verbatimEventDate: 9.ii.13; **Record Level:** institutionCode: DZRJ; basisOfRecord: PreservedSpecimen
- **Type status:** Other material. **Occurrence:** recordedBy: Santos, A.P.M. | Takiya, D.M.; individualCount: 1; sex: male; lifeStage: adult; **Location:** country: Brazil; stateProvince: Piauí; municipality: Piracuruca; locality: Parque Nacional de Sete Cidades, Poço do Bananeira; maximumElevationInMeters: 189; verbatimCoordinates: 4°5'55.8"S, 41°40'33.8"W; **Identification:** identifiedBy: Julianna Freires Barbosa; **Event:** samplingProtocol: Manual; verbatimEventDate: 8.ii.13; **Record Level:** institutionCode: DZRJ; basisOfRecord: PreservedSpecimen
- **Type status:** Other material. **Occurrence:** recordedBy: Santos, A.P.M. | Takiya, D.M.; individualCount: 14; lifeStage: immature; **Location:** country: Brazil; stateProvince: Piauí; municipality: Piracuruca; locality: Parque Nacional de Sete Cidades, Cachoeira do Riachão; maximumElevationInMeters: 171; verbatimCoordinates: 4°6'28"S, 41°40'13"W; **Identification:** identifiedBy: Julianna Freires Barbosa; **Event:** samplingProtocol: Manual; verbatimEventDate: 9.ii.13; **Record Level:** institutionCode: DZRJ; basisOfRecord: PreservedSpecimen

##### Distribution

Brazil: PI!, RJ.

##### Notes

New species record for Northeastern Brazil.

#### Buenoa salutis

Kirkaldy, 1904

##### Materials

- **Type status:** Other material. **Occurrence:** recordedBy: Takiya, D.M.; individualCount: 1; sex: male; lifeStage: adult; **Location:** country: Brazil; stateProvince: Piauí; municipality: Piracuruca; locality: Parque Nacional de Sete Cidades, Alojamento; maximumElevationInMeters: 193; verbatimCoordinates: 4°5'57"S, 41°42'34"W; **Identification:** identifiedBy: Julianna Freires Barbosa; **Event:** samplingProtocol: White sheet light trap; verbatimEventDate: 18.iv.12; **Record Level:** institutionCode: DZRJ; basisOfRecord: PreservedSpecimen
- **Type status:** Other material. **Occurrence:** recordedBy: Takiya, D.M.; individualCount: 3; sex: female; lifeStage: adult; **Location:** country: Brazil; stateProvince: Piauí; municipality: Piracuruca; locality: Parque Nacional de Sete Cidades, Alojamento; maximumElevationInMeters: 193; verbatimCoordinates: 4°5'57"S, 41°42'34"W; **Identification:** identifiedBy: Julianna Freires Barbosa; **Event:** samplingProtocol: White sheet light trap; verbatimEventDate: 18.iv.12; **Record Level:** institutionCode: DZRJ | CZMA; basisOfRecord: PreservedSpecimen
- **Type status:** Other material. **Occurrence:** recordedBy: Takiya, D.M.; individualCount: 2; sex: female; lifeStage: adult; **Location:** country: Brazil; stateProvince: Piauí; municipality: Piracuruca; locality: Parque Nacional de Sete Cidades, Cachoeira do Riachão; maximumElevationInMeters: 171; verbatimCoordinates: 4°6'28"S, 41°40'13"W; **Identification:** identifiedBy: Julianna Freires Barbosa; **Event:** samplingProtocol: Pennsylvania light trap; verbatimEventDate: 19.iv.12; **Record Level:** institutionCode: CZMA; basisOfRecord: PreservedSpecimen
- **Type status:** Other material. **Occurrence:** recordedBy: Takiya, D.M.; individualCount: 2; sex: male; **Location:** country: Brazil; stateProvince: Piauí; municipality: Piracuruca; locality: Parque Nacional de Sete Cidades, Riacho da Bananeira; maximumElevationInMeters: 189; verbatimCoordinates: 4°5'59"S, 41°40'48"W; **Identification:** identifiedBy: Julianna Freires Barbosa; **Event:** samplingProtocol: Pennsylvania light trap; verbatimEventDate: 18.iv.12; **Record Level:** institutionCode: DZRJ | CZMA; basisOfRecord: PreservedSpecimen
- **Type status:** Other material. **Occurrence:** recordedBy: Takiya, D.M.; individualCount: 2; sex: female; **Location:** country: Brazil; stateProvince: Piauí; municipality: Piracuruca; locality: Parque Nacional de Sete Cidades, Riacho da Bananeira; maximumElevationInMeters: 189; verbatimCoordinates: 4°5'59"S, 41°40'48"W; **Identification:** identifiedBy: Julianna Freires Barbosa; **Event:** samplingProtocol: Pennsylvania light trap; verbatimEventDate: 18.iv.12; **Record Level:** institutionCode: DZRJ | CZMA; basisOfRecord: PreservedSpecimen

##### Distribution

Trinidad and Tobago. Colombia. Venezuela. Guyana. Suriname. French Guiana. Brazil: RR, PA, AM, PI!, CE, PB, PE, MT, MS, MG, SP, RJ, RS. Bolivia. Paraguay. Argentina.

##### Notes

New species record for PI.

#### Buenoa tarsalis

Truxal, 1953

##### Materials

- **Type status:** Other material. **Occurrence:** recordedBy: Santos, A.P.M. | Takiya, D.M.; individualCount: 2; sex: male; lifeStage: adult; **Location:** country: Brazil; stateProvince: Piauí; municipality: Piracuruca; locality: Parque Nacional de Sete Cidades, Riacho da Bananeira; maximumElevationInMeters: 189; verbatimCoordinates: 4°5'59"S, 41°40'48"W; **Identification:** identifiedBy: Julianna Freires Barbosa; **Event:** samplingProtocol: Manual; verbatimEventDate: 8.ii.13; **Record Level:** institutionCode: DZRJ; basisOfRecord: PreservedSpecimen
- **Type status:** Other material. **Occurrence:** recordedBy: Santos, A.P.M. | Takiya, D.M.; individualCount: 15; lifeStage: immature; **Location:** country: Brazil; stateProvince: Piauí; municipality: Piracuruca; locality: Parque Nacional de Sete Cidades, Riacho da Bananeira; maximumElevationInMeters: 189; verbatimCoordinates: 4°5'59"S, 41°40'48"W; **Identification:** identifiedBy: Julianna Freires Barbosa; **Event:** samplingProtocol: Manual; verbatimEventDate: 8.ii.13; **Record Level:** institutionCode: DZRJ; basisOfRecord: PreservedSpecimen
- **Type status:** Other material. **Occurrence:** recordedBy: Takiya, D.M.; individualCount: 2; sex: male; lifeStage: adult; **Location:** country: Brazil; stateProvince: Piauí; municipality: Piracuruca; locality: Parque Nacional de Sete Cidades, Alojamento; maximumElevationInMeters: 193; verbatimCoordinates: 4°5'57"S, 41°42'34"W; **Identification:** identifiedBy: Julianna Freires Barbosa; **Event:** samplingProtocol: White sheet light trap; verbatimEventDate: 18.iv.12; **Record Level:** institutionCode: DZRJ; basisOfRecord: PreservedSpecimen
- **Type status:** Other material. **Occurrence:** recordedBy: Takiya, D.M.; individualCount: 3; sex: female; lifeStage: adult; **Location:** country: Brazil; stateProvince: Piauí; municipality: Piracuruca; locality: Parque Nacional de Sete Cidades, Alojamento; maximumElevationInMeters: 193; verbatimCoordinates: 4°5'57"S, 41°42'34"W; **Identification:** identifiedBy: Julianna Freires Barbosa; **Event:** samplingProtocol: White sheet light trap; verbatimEventDate: 18.iv.12; **Record Level:** institutionCode: DZRJ; basisOfRecord: PreservedSpecimen

##### Distribution

Brazil: PA, AM, PI!, CE, RN, PB, PE, MG, RJ.

##### Notes

New species record for PI.

#### Buenoa unguis

Truxal, 1953

##### Materials

- **Type status:** Other material. **Occurrence:** recordedBy: Santos, A.P.M. | Takiya, D.M.; individualCount: 1; sex: male; lifeStage: adult; **Location:** country: Brazil; stateProvince: Piauí; municipality: Piracuruca; locality: Parque Nacional de Sete Cidades, Riacho da Bananeira; maximumElevationInMeters: 189; verbatimCoordinates: 4°5'59"S, 41°40'48"W; **Identification:** identifiedBy: Julianna Freires Barbosa; **Event:** samplingProtocol: Manual; verbatimEventDate: 8.ii.13; **Record Level:** institutionCode: DZRJ; basisOfRecord: PreservedSpecimen
- **Type status:** Other material. **Occurrence:** recordedBy: Santos, A.P.M. | Takiya, D.M.; individualCount: 9; lifeStage: immature; **Location:** country: Brazil; stateProvince: Piauí; municipality: Piracuruca; locality: Parque Nacional de Sete Cidades, Riacho da Bananeira; maximumElevationInMeters: 189; verbatimCoordinates: 4°5'59"S, 41°40'48"W; **Identification:** identifiedBy: Julianna Freires Barbosa; **Event:** samplingProtocol: Manual; verbatimEventDate: 8.ii.13; **Record Level:** institutionCode: DZRJ; basisOfRecord: PreservedSpecimen
- **Type status:** Other material. **Occurrence:** recordedBy: Takiya, D.M.; individualCount: 10; sex: male; lifeStage: adult; **Location:** country: Brazil; stateProvince: Piauí; municipality: Piracuruca; locality: Parque Nacional de Sete Cidades, Riacho da Bananeira; maximumElevationInMeters: 189; verbatimCoordinates: 4°5'59"S, 41°40'48"W; **Identification:** identifiedBy: Julianna Freires Barbosa; **Event:** samplingProtocol: Pennsylvania light trap; verbatimEventDate: 18.iv.12; **Record Level:** institutionCode: DZRJ; basisOfRecord: PreservedSpecimen
- **Type status:** Other material. **Occurrence:** recordedBy: Takiya, D.M.; individualCount: 16; sex: female; lifeStage: adult; **Location:** country: Brazil; stateProvince: Piauí; municipality: Piracuruca; locality: Parque Nacional de Sete Cidades, Riacho da Bananeira; maximumElevationInMeters: 189; verbatimCoordinates: 4°5'59"S, 41°40'48"W; **Identification:** identifiedBy: Julianna Freires Barbosa; **Event:** samplingProtocol: Pennsylvania light trap; verbatimEventDate: 18.iv.12; **Record Level:** institutionCode: DZRJ; basisOfRecord: PreservedSpecimen
- **Type status:** Other material. **Occurrence:** recordedBy: Takiya, D.M.; individualCount: 7; sex: male; lifeStage: adult; **Location:** country: Brazil; stateProvince: Piauí; municipality: Piracuruca; locality: Parque Nacional de Sete Cidades, Alojamento; maximumElevationInMeters: 193; verbatimCoordinates: 4°5'57"S, 41°42'34"W; **Identification:** identifiedBy: Julianna Freires Barbosa; **Event:** samplingProtocol: White sheet light trap; verbatimEventDate: 18.iv.12; **Record Level:** institutionCode: DZRJ; basisOfRecord: PreservedSpecimen
- **Type status:** Other material. **Occurrence:** recordedBy: Takiya, D.M.; individualCount: 15; sex: female; lifeStage: adult; **Location:** country: Brazil; stateProvince: Piauí; municipality: Piracuruca; locality: Parque Nacional de Sete Cidades, Alojamento; maximumElevationInMeters: 193; verbatimCoordinates: 4°5'57"S, 41°42'34"W; **Identification:** identifiedBy: Julianna Freires Barbosa; **Event:** samplingProtocol: White sheet light trap; verbatimEventDate: 18.iv.12; **Record Level:** institutionCode: DZRJ; basisOfRecord: PreservedSpecimen
- **Type status:** Other material. **Occurrence:** recordedBy: Takiya, D.M.; individualCount: 3; sex: male; lifeStage: adult; **Location:** country: Brazil; stateProvince: Piauí; municipality: Piracuruca; locality: Parque Nacional de Sete Cidades, Cachoeira do Riachão; maximumElevationInMeters: 171; verbatimCoordinates: 4°6'28"S, 41°40'13"W; **Identification:** identifiedBy: Julianna Freires Barbosa; **Event:** samplingProtocol: Pennsylvania light trap; verbatimEventDate: 19.iv.12; **Record Level:** institutionCode: DZRJ; basisOfRecord: PreservedSpecimen
- **Type status:** Other material. **Occurrence:** recordedBy: Takiya, D.M.; individualCount: 12; sex: female; lifeStage: adult; **Location:** country: Brazil; stateProvince: Piauí; municipality: Piracuruca; locality: Parque Nacional de Sete Cidades, Cachoeira do Riachão; maximumElevationInMeters: 171; verbatimCoordinates: 4°6'28"S, 41°40'13"W; **Identification:** identifiedBy: Julianna Freires Barbosa; **Event:** samplingProtocol: Pennsylvania light trap; verbatimEventDate: 19.iv.12; **Record Level:** institutionCode: DZRJ; basisOfRecord: PreservedSpecimen

##### Distribution

Venezuela. Brazil: PA, AM, PI!, CE, RN, TO, PB, PE, MG, SP, RJ. Peru. Bolivia. Paraguay. Argentina.

##### Notes

New species record for PI.

#### Buenoa sp. 1

##### Materials

- **Type status:** Other material. **Occurrence:** recordedBy: Santos, A.P.M. | Takiya, D.M.; individualCount: 1; sex: male; lifeStage: adult; **Location:** country: Brazil; stateProvince: Piauí; municipality: Piracuruca; locality: Parque Nacional de Sete Cidades, Cachoeira do Riachão; maximumElevationInMeters: 171; verbatimCoordinates: 4°6'28"S, 41°40'13"W; **Identification:** identifiedBy: Julianna Freires Barbosa; **Event:** samplingProtocol: Manual; verbatimEventDate: 12.ii.13; **Record Level:** institutionCode: DZRJ; basisOfRecord: PreservedSpecimen
- **Type status:** Other material. **Occurrence:** recordedBy: Santos, A.P.M. | Takiya, D.M.; individualCount: 1; lifeStage: immature; **Location:** country: Brazil; stateProvince: Piauí; municipality: Piracuruca; locality: Parque Nacional de Sete Cidades, Cachoeira do Riachão; maximumElevationInMeters: 171; verbatimCoordinates: 4°6'28"S, 41°40'13"W; **Identification:** identifiedBy: Julianna Freires Barbosa; **Event:** samplingProtocol: Manual; verbatimEventDate: 12.ii.13; **Record Level:** institutionCode: DZRJ; basisOfRecord: PreservedSpecimen

##### Notes

Undescribed species.

#### Martarega

White, 1879

#### Martarega bentoi

Truxal, 1949

##### Materials

- **Type status:** Other material. **Occurrence:** recordedBy: Santos, A.P.M. | Takiya, D.M.; individualCount: 1; sex: male; lifeStage: adult; **Location:** country: Brazil; stateProvince: Piauí; municipality: Piracuruca; locality: Parque Nacional de Sete Cidades, Alojamento; maximumElevationInMeters: 193; verbatimCoordinates: 4°5'57"S, 41°42'34"W; **Identification:** identifiedBy: Julianna Freires Barbosa; **Event:** samplingProtocol: Manual; verbatimEventDate: 12.ii.13; **Record Level:** institutionCode: DZRJ; basisOfRecord: PreservedSpecimen
- **Type status:** Other material. **Occurrence:** recordedBy: Santos, A.P.M. | Takiya, D.M.; individualCount: 5; lifeStage: immature; **Location:** country: Brazil; stateProvince: Piauí; municipality: Piracuruca; locality: Parque Nacional de Sete Cidades, Alojamento; maximumElevationInMeters: 193; verbatimCoordinates: 4°5'57"S, 41°42'34"W; **Identification:** identifiedBy: Julianna Freires Barbosa; **Event:** samplingProtocol: Manual; verbatimEventDate: 12.ii.13; **Record Level:** institutionCode: DZRJ; basisOfRecord: PreservedSpecimen
- **Type status:** Other material. **Occurrence:** recordedBy: Takiya, D.M.; individualCount: 3; sex: male; lifeStage: adult; **Location:** country: Brazil; stateProvince: Piauí; municipality: Piracuruca; locality: Parque Nacional de Sete Cidades, Cachoeira do Riachão; maximumElevationInMeters: 171; verbatimCoordinates: 4°6'28"S, 41°40'13"W; **Identification:** identifiedBy: Julianna Freires Barbosa; **Event:** samplingProtocol: Manual; verbatimEventDate: 18.iv.12; **Record Level:** institutionCode: DZRJ; basisOfRecord: PreservedSpecimen
- **Type status:** Other material. **Occurrence:** recordedBy: Takiya, D.M.; individualCount: 1; sex: female; lifeStage: adult; **Location:** country: Brazil; stateProvince: Piauí; municipality: Piracuruca; locality: Parque Nacional de Sete Cidades, Cachoeira do Riachão; maximumElevationInMeters: 171; verbatimCoordinates: 4°6'28"S, 41°40'13"W; **Identification:** identifiedBy: Julianna Freires Barbosa; **Event:** samplingProtocol: Manual; verbatimEventDate: 18.iv.12; **Record Level:** institutionCode: DZRj; basisOfRecord: PreservedSpecimen

##### Distribution

Brazil: PI, CE!, PE, MT, MG, RJ. Argentina.

#### Martarega membranacea

White, 1870

##### Materials

- **Type status:** Other material. **Occurrence:** recordedBy: Santos, A.P.M. | Takiya, D.M.; individualCount: 1; sex: male; lifeStage: adult; **Location:** country: Brazil; stateProvince: Piauí; municipality: Piracuruca; locality: Parque Nacional de Sete Cidades, Poço do Bananeira; maximumElevationInMeters: 158; verbatimCoordinates: 4°5'55.8"S, 41°40'33.8"W; **Identification:** identifiedBy: Julianna Freires Barbosa; **Event:** samplingProtocol: Manual; verbatimEventDate: 9.ii.13; **Record Level:** institutionCode: DZRJ; basisOfRecord: PreservedSpecimen
- **Type status:** Other material. **Occurrence:** recordedBy: Santos, A.P.M. | Takiya, D.M.; individualCount: 6; lifeStage: immature; **Location:** country: Brazil; stateProvince: Piauí; municipality: Piracuruca; locality: Parque Nacional de Sete Cidades, Poço do Bananeira; maximumElevationInMeters: 158; verbatimCoordinates: 4°5'55.8"S, 41°40'33.8"W; **Identification:** identifiedBy: Julianna Freires Barbosa; **Event:** samplingProtocol: Manual; verbatimEventDate: 9.ii.13; **Record Level:** institutionCode: DZRJ; basisOfRecord: PreservedSpecimen

##### Distribution

Colombia. Guyana. Suriname. Brazil: PA, AM, PI!, TO, RO, MT, GO, MG, MS, SP, RJ. Ecuador. Bolivia. Argentina.

##### Notes

New species record for Northeastern Brazil.

#### Notonecta

Linnaeus, 1758

##### Notes

Genus firstly recorded from PI in Barbosa and Nessimian 2013.

#### Notonecta disturbata

Hungerford, 1926

##### Materials

- **Type status:** Other material. **Occurrence:** recordedBy: Santos, A.P.M. | Takiya, D.M.; individualCount: 3; sex: male; lifeStage: adult; **Location:** country: Brazil; stateProvince: Piauí; municipality: Piracuruca; locality: Parque Nacional de Sete Cidades, Alojamento; maximumElevationInMeters: 193; verbatimCoordinates: 4°5'57"S, 41°42'34"W; **Identification:** identifiedBy: Julianna Freires Barbosa; **Event:** samplingProtocol: Manual; verbatimEventDate: 12.ii.13; **Record Level:** institutionCode: DZRJ; basisOfRecord: PreservedSpecimen
- **Type status:** Other material. **Occurrence:** recordedBy: Santos, A.P.M. | Takiya, D.M.; individualCount: 1; sex: female; lifeStage: adult; **Location:** country: Brazil; stateProvince: Piauí; municipality: Piracuruca; locality: Parque Nacional de Sete Cidades, Alojamento; maximumElevationInMeters: 193; verbatimCoordinates: 4°5'57"S, 41°42'34"W; **Identification:** identifiedBy: Julianna Freires Barbosa; **Event:** samplingProtocol: Manual; verbatimEventDate: 12.ii.13; **Record Level:** institutionCode: DZRJ; basisOfRecord: PreservedSpecimen
- **Type status:** Other material. **Occurrence:** recordedBy: Santos, A.P.M. | Takiya, D.M.; individualCount: 2; sex: male; lifeStage: adult; **Location:** country: Brazil; stateProvince: Piauí; municipality: Piracuruca; locality: Parque Nacional de Sete Cidades, Cachoeira do Riachão; maximumElevationInMeters: 171; verbatimCoordinates: 4°6'28"S, 41°40'13"W; **Identification:** identifiedBy: Julianna Freires Barbosa; **Event:** samplingProtocol: Manual; verbatimEventDate: 9.ii.13; **Record Level:** institutionCode: DZRJ; basisOfRecord: PreservedSpecimen
- **Type status:** Other material. **Occurrence:** recordedBy: Santos, A.P.M. | Takiya, D.M.; individualCount: 1; sex: female; lifeStage: adult; **Location:** country: Brazil; stateProvince: Piauí; municipality: Piracuruca; locality: Parque Nacional de Sete Cidades, Cachoeira do Riachão; maximumElevationInMeters: 171; verbatimCoordinates: 4°6'28"S, 41°40'13"W; **Identification:** identifiedBy: Julianna Freires Barbosa; **Event:** samplingProtocol: Manual; verbatimEventDate: 9.ii.13; **Record Level:** institutionCode: DZRJ; basisOfRecord: PreservedSpecimen
- **Type status:** Other material. **Occurrence:** recordedBy: Santos, A.P.M. | Takiya, D.M.; individualCount: 1; lifeStage: immature; **Location:** country: Brazil; stateProvince: Piauí; municipality: Piracuruca; locality: Parque Nacional de Sete Cidades, Cachoeira do Riachão; maximumElevationInMeters: 171; verbatimCoordinates: 4°6'28"S, 41°40'13"W; **Identification:** identifiedBy: Julianna Freires Barbosa; **Event:** samplingProtocol: Manual; verbatimEventDate: 9.ii.13; **Record Level:** institutionCode: DZRJ; basisOfRecord: PreservedSpecimen

##### Distribution

Brazil: PA, PI!, TO, MT, GO, MG, SP, RJ, RS. Paraguay. Argentina.

##### Notes

Species firstly recorded from Northeastern Brazil in Barbosa and Nessimian 2013.

#### Ochteridae

##### Notes

Family firstly recorded from PI in Cordeiro et al. 2014.

#### Ochterus

Latreille, 1807

##### Notes

Genus firstly recorded from PI in Cordeiro et al. 2014.

#### Ochterus santosi

Cordeiro & Moreira, 2014

##### Materials

- **Type status:** Other material. **Occurrence:** recordedBy: Santos, A.P.M. | Takiya, D.M.; individualCount: 1; sex: male; lifeStage: adult; **Location:** country: Brazil; stateProvince: Piauí; municipality: Piracuruca; locality: Parque Nacional de Sete Cidades, Olha d'água dos Milagres; maximumElevationInMeters: 180; verbatimCoordinates: 4°5'31.8"S, 41°40'48.2"W; **Identification:** identifiedBy: Isabelle da R. S. Cordeiro; **Event:** samplingProtocol: Manual; verbatimEventDate: 8.ii.13; **Record Level:** institutionCode: CZMA; basisOfRecord: PreservedSpecimen
- **Type status:** Other material. **Occurrence:** recordedBy: Santos, A.P.M. | Takiya, D.M.; individualCount: 1; sex: female; lifeStage: adult; **Location:** country: Brazil; stateProvince: Piauí; municipality: Piracuruca; locality: Parque Nacional de Sete Cidades, Cachoeira do Riachão; maximumElevationInMeters: 171; verbatimCoordinates: 4°6'28"S, 41°40'13"W; **Identification:** identifiedBy: Isabelle da R. S. Cordeiro; **Event:** samplingProtocol: Manual; verbatimEventDate: 9.ii.13; **Record Level:** institutionCode: CZMA; basisOfRecord: PreservedSpecimen

##### Distribution

Brazil: PI.

##### Notes

Species described in Cordeiro et al. 2014.

#### Odonata

#### Anisoptera

#### Aeshnidae

#### Coryphaeschna

##### Notes

New genus record for PI.

#### Coryphaeschna viriditas

Calvert, 1952

##### Materials

- **Type status:** Other material. **Occurrence:** recordedBy: Santos, A.P.M. | Takiya, D.M.; individualCount: 1; sex: male; lifeStage: adult; **Location:** country: Brazil; stateProvince: Piauí; municipality: Piracuruca; locality: Parque Nacional de Sete Cidades, Olho d'água Piscina do Bacuri; maximumElevationInMeters: 171; verbatimCoordinates: 4°6'1.2"S, 41°42'38.8"W; **Identification:** identifiedBy: Ângelo Parise Pinto; **Event:** samplingProtocol: Manual; verbatimEventDate: 9.ii.13; **Record Level:** institutionCode: DZRJ; basisOfRecord: PreservedSpecimen

##### Distribution

USA south to Panama. Trinidad and Tobago. Colombia. Venezuela. Guyana. Suriname. French Guiana. Brazil: AM/PA, PI!, PE, MT, MG, ES, RJ. Ecuador. Peru. Bolivia. Paraguay.

##### Notes

New species record for PI. See Fig. 27.

#### Gynacantha

Rambur, 1842

##### Notes

New genus record for PI.

#### Gynacantha nervosa

Rambur, 1842

##### Materials

- **Type status:** Other material. **Occurrence:** recordedBy: Rafael, J.A. | Limeira-de-Oliveira, F. | et al.; individualCount: 1; sex: female; lifeStage: adult; **Location:** country: Brazil; stateProvince: Piauí; municipality: Piracuruca; locality: Parque Nacional de Sete Cidades, Alojamento; maximumElevationInMeters: 193; verbatimCoordinates: 4°5'57"S, 41°42'34"W; **Identification:** identifiedBy: Ângelo Parise Pinto; **Event:** samplingProtocol: Suspended intercept trap; verbatimEventDate: 19.iv.12; **Record Level:** institutionCode: CZMA; basisOfRecord: PreservedSpecimen
- **Type status:** Other material. **Occurrence:** recordedBy: Santos, A.P.M. | Takiya, D.M.; individualCount: 1; sex: female; lifeStage: adult; **Location:** country: Brazil; stateProvince: Piauí; municipality: Piracuruca; locality: Parque Nacional de Sete Cidades, Olha d'água dos Milagres; maximumElevationInMeters: 180; verbatimCoordinates: 4°5'31.8"S, 41°40'48.2"W; **Identification:** identifiedBy: Ângelo Parise Pinto; **Event:** samplingProtocol: Manual; verbatimEventDate: 8.ii.13; **Record Level:** institutionCode: DZRJ; basisOfRecord: PreservedSpecimen
- **Type status:** Other material. **Occurrence:** recordedBy: Santos, A.P.M. | Takiya, D.M.; individualCount: 1; sex: male; lifeStage: adult; **Location:** country: Brazil; stateProvince: Piauí; municipality: Piracuruca; locality: Parque Nacional de Sete Cidades, Alojamento; maximumElevationInMeters: 193; verbatimCoordinates: 4°5'57"S, 41°42'34"W; **Identification:** identifiedBy: Ângelo Parise Pinto; **Event:** samplingProtocol: Manual; verbatimEventDate: 9.ii.13; **Record Level:** institutionCode: DZRJ; basisOfRecord: PreservedSpecimen

##### Distribution

USA south to Panama. Trinidad and Tobago. Colombia. Venezuela. Guyana. Suriname. French Guiana. Brazil: RR, PI!, CE, PE, MT, MG, MS, ES, RJ. Ecuador. Peru. Bolivia.

##### Notes

New species record for PI.

#### Triacanthagyna

##### Notes

New genus record for PI.

#### Triacanthagyna caribbea

Williamson, 1923

##### Materials

- **Type status:** Other material. **Occurrence:** recordedBy: Santos, A.P.M. | Takiya, D.M.; individualCount: 1; sex: female; lifeStage: adult; **Location:** country: Brazil; stateProvince: Piauí; municipality: Piracuruca; locality: Parque Nacional de Sete Cidades, Olho d'água Piscina do Bacuri; maximumElevationInMeters: 171; verbatimCoordinates: 4°6'1.2"S, 41°42'38.8"W; **Identification:** identifiedBy: Ângelo Parise Pinto; **Event:** samplingProtocol: White sheet light trap; verbatimEventDate: 9.ii.13; **Record Level:** institutionCode: DZRJ; basisOfRecord: PreservedSpecimen

##### Distribution

Mexico. Belize. Guatemala. Honduras. Costa Rica. Dominican Republic. Puerto Rico. Trinidad and Tobago. Colombia. Venezuela. Suriname. French Guiana. Brazil: PA, PI!, MT, BA, ES, SP, RJ, PR. Ecuador. Peru. Bolivia.

##### Notes

New species record for PI.

#### Libellulidae

#### Brechmorhoga

Kirby, 1889

##### Notes

New genus record for PI.

#### Brechmorhoga sp. 1

##### Materials

- **Type status:** Other material. **Occurrence:** recordedBy: Rafael, J.A. | Limeira-de-Oliveira, F. | Takiya, D.M. | et al.; individualCount: 1; sex: female; lifeStage: adult; **Location:** country: Brazil; stateProvince: Piauí; municipality: Piracuruca; locality: Parque Nacional de Sete Cidades, Alojamento; maximumElevationInMeters: 193; verbatimCoordinates: 4°5'57"S, 41°42'34"W; **Identification:** identifiedBy: Ângelo Parise Pinto; **Event:** samplingProtocol: Malaise intercept trap; verbatimEventDate: 19.iv.12; **Record Level:** institutionCode: DZRJ; basisOfRecord: PreservedSpecimen

#### Diastatops

Rambur, 1842

##### Notes

New genus record for PI.

#### Diastatops obscura

(Fabricius, 1775)

##### Materials

- **Type status:** Other material. **Occurrence:** recordedBy: Rafael, J.A. | Limeira-de-Oliveira, F. | Takiya, D.M. | et al.; individualCount: 1; sex: male; lifeStage: adult; **Location:** country: Brazil; stateProvince: Piauí; municipality: Piracuruca; locality: Parque Nacional de Sete Cidades, Riacho da Bananeira; maximumElevationInMeters: 189; verbatimCoordinates: 4°5'59"S, 41°40'48"W; **Identification:** identifiedBy: Alcimar Carvalho | Ângelo Parise Pinto; **Event:** samplingProtocol: Malaise intercept trap; verbatimEventDate: 19.iv.12; **Record Level:** institutionCode: DZRJ; basisOfRecord: PreservedSpecimen
- **Type status:** Other material. **Occurrence:** recordedBy: Rafael, J.A. | Limeira-de-Oliveira, F. | Takiya, D.M. | et al.; individualCount: 1; sex: female; lifeStage: adult; **Location:** country: Brazil; stateProvince: Piauí; municipality: Piracuruca; locality: Parque Nacional de Sete Cidades, Riacho da Piedade; maximumElevationInMeters: 169; verbatimCoordinates: 4°6'34"S, 41°43'39"W; **Identification:** identifiedBy: Alcimar Carvalho | Ângelo Parise Pinto; **Event:** samplingProtocol: Malaise intercept trap; verbatimEventDate: 21.iv.12; **Record Level:** institutionCode: INPA; basisOfRecord: PreservedSpecimen

##### Distribution

Colombia. Venezuela. Guyana. Suriname. French Guiana. Brazil: RR, PA, AM, MA, PI!, CE, RN, TO, PE, MT, BA, MG, MS, ES, SP, RJ. Peru. Bolivia. Paraguay. Argentina.

##### Notes

New species record for PI.

#### Erythrodiplax

Brauer, 1868

##### Notes

New genus record for PI.

#### Erythrodiplax basalis

(Kirby, 1897)

##### Materials

- **Type status:** Other material. **Occurrence:** recordedBy: Santos, A.P.M. | Takiya, D.M.; individualCount: 1; sex: male; lifeStage: adult; **Location:** country: Brazil; stateProvince: Piauí; municipality: Piracuruca; locality: Parque Nacional de Sete Cidades, Poço do Bananeira; maximumElevationInMeters: 158; verbatimCoordinates: 4°5'55.8"S, 41°40'33.8"W; **Identification:** identifiedBy: Ângelo Parise Pinto; **Event:** samplingProtocol: Manual; verbatimEventDate: 11.ii.13; **Record Level:** institutionCode: DZRJ; basisOfRecord: PreservedSpecimen
- **Type status:** Other material. **Occurrence:** recordedBy: Rafael, J.A. | Limeira-de-Oliveira, F. | Takiya, D.M. | et al.; individualCount: 1; sex: female; lifeStage: adult; **Location:** country: Brazil; stateProvince: Piauí; municipality: Piracuruca; locality: Parque Nacional de Sete Cidades, Alojamento; maximumElevationInMeters: 193; verbatimCoordinates: 4°5'57"S, 41°42'34"W; **Identification:** identifiedBy: Ângelo Parise Pinto; **Event:** samplingProtocol: Malaise intercept trap; verbatimEventDate: 19.iv.12; **Record Level:** institutionCode: DZRJ; basisOfRecord: PreservedSpecimen

##### Distribution

Trinidad and Tobago. Colombia. Venezuela. Guyana. Suriname. French Guiana. Brazil: RR, PA, AM, MA, PI!, CE, PE, RO, BA, MT, MG, MS, ES, SP, RJ, RS. Ecuador. Peru. Bolivia. Paraguay. Argentina. Uruguay.

##### Notes

New species record for PI.

#### Erythrodiplax famula

(Erichson in Schomburgk, 1848)

##### Materials

- **Type status:** Other material. **Occurrence:** recordedBy: Takiya, D.M. | Cavichioli, R.R.; individualCount: 1; sex: male; lifeStage: adult; **Location:** country: Brazil; stateProvince: Piauí; municipality: Piracuruca; locality: Parque Nacional de Sete Cidades, Riacho da Piedade; maximumElevationInMeters: 169; verbatimCoordinates: 4°6'34"S, 41°43'39"W; **Identification:** identifiedBy: Ângelo Parise Pinto; **Event:** samplingProtocol: Manual; verbatimEventDate: 19.iv.12; **Record Level:** institutionCode: DZRJ; basisOfRecord: PreservedSpecimen

##### Distribution

Trinidad and Tobago. Colombia. Venezuela. Guyana. Suriname. French Guiana. Brazil: PA, AM, PI!, PE, GO, MG, ES, SP, RJ. Peru. Argentina.

##### Notes

New species record for PI.

#### Erythrodiplax fusca

(Rambur, 1842)

##### Materials

- **Type status:** Other material. **Occurrence:** recordedBy: Rafael, J.A. | Limeira-de-Oliveira, F. | Takiya, D.M. | et al.; individualCount: 1; sex: female; lifeStage: adult; **Location:** country: Brazil; stateProvince: Piauí; municipality: Piracuruca; locality: Parque Nacional de Sete Cidades, Alojamento; maximumElevationInMeters: 193; verbatimCoordinates: 4°5'57"S, 41°42'34"W; **Identification:** identifiedBy: Ângelo Parise Pinto; **Event:** samplingProtocol: Malaise intercept trap; verbatimEventDate: 19.iv.12; **Record Level:** institutionCode: DZRJ; basisOfRecord: PreservedSpecimen

##### Distribution

USA south to Panama. Trinidad and Tobago. Colombia. Venezuela. Guyana. Suriname. French Guiana. Brazil: PA, AM, MA, PI!, CE, RN, PE, MT, RO, BA, GO, MG, MS, ES, SP, RJ, SC, RS. Ecuador. Peru. Bolivia. Paraguay. Argentina. Uruguay.

##### Notes

New species record for PI.

#### Erythrodiplax umbrata

(Linnaeus, 1758)

##### Materials

- **Type status:** Other material. **Occurrence:** recordedBy: Santos, A.P.M. | Takiya, D.M.; individualCount: 1; sex: male; lifeStage: adult; **Location:** country: Brazil; stateProvince: Piauí; municipality: Piracuruca; locality: Parque Nacional de Sete Cidades, Riacho da Bananeira; maximumElevationInMeters: 189; verbatimCoordinates: 4°5'59"S, 41°40'48"W; **Identification:** identifiedBy: Ângelo Parise Pinto; **Event:** samplingProtocol: Manual; verbatimEventDate: 13.ii.13; **Record Level:** institutionCode: CZMA; basisOfRecord: PreservedSpecimen
- **Type status:** Other material. **Occurrence:** recordedBy: Santos, A.P.M. | Takiya, D.M.; individualCount: 1; sex: male; lifeStage: adult; **Location:** country: Brazil; stateProvince: Piauí; municipality: Piracuruca; locality: Parque Nacional de Sete Cidades, Riacho da Bananeira; maximumElevationInMeters: 189; verbatimCoordinates: 4°5'59"S, 41°40'48"W; **Identification:** identifiedBy: Ângelo Parise Pinto; **Event:** samplingProtocol: Manual; verbatimEventDate: 13.ii.13; **Record Level:** institutionCode: DZRJ; basisOfRecord: PreservedSpecimen
- **Type status:** Other material. **Occurrence:** recordedBy: Takiya, D.M. | Cavichioli, R.R.; individualCount: 4; sex: male; lifeStage: adult; **Location:** country: Brazil; stateProvince: Piauí; municipality: Piracuruca; locality: Parque Nacional de Sete Cidades, Riacho da Piedade; maximumElevationInMeters: 169; verbatimCoordinates: 4°6'34"S, 41°43'39"W; **Identification:** identifiedBy: Alcimar Carvalho | Ângelo Parise Pinto; **Event:** samplingProtocol: Manual; verbatimEventDate: 19.iv.12; **Record Level:** institutionCode: DZRJ; basisOfRecord: PreservedSpecimen
- **Type status:** Other material. **Occurrence:** recordedBy: Santos, A.P.M. | Takiya, D.M.; individualCount: 1; sex: female; lifeStage: adult; **Location:** country: Brazil; stateProvince: Piauí; municipality: Piracuruca; locality: Parque Nacional de Sete Cidades, Olha d'água dos Milagres; maximumElevationInMeters: 180; verbatimCoordinates: 4°5'31.8"S, 41°40'48.2"W; **Identification:** identifiedBy: Ângelo Parise Pinto; **Event:** samplingProtocol: White sheet light trap; verbatimEventDate: 9.ii.13; **Record Level:** institutionCode: DZRJ; basisOfRecord: PreservedSpecimen

##### Distribution

USA south to Panama. Trinidad and Tobago. Colombia. Venezuela. Guyana. Suriname. French Guyana. Brazil: RR, PA, AM, PI!, CE, PE, MT, RO, BA, MG, MS, ES, SP, RJ, RS. Ecuador. Peru. Bolivia. Paraguay. Argentina. Uruguay.

##### Notes

New species record for PI.

#### Orthemis

Hagen, 1861

##### Notes

New genus record for PI.

#### Orthemis aequilibris [red morph]f.[red morph]

Calvert, 1909

##### Materials

- **Type status:** Other material. **Occurrence:** recordedBy: Santos, A.P.M. | Takiya, D.M.; individualCount: 1; sex: male; lifeStage: adult; **Location:** country: Brazil; stateProvince: Piauí; municipality: Piracuruca; locality: Parque Nacional de Sete Cidades, Riacho da Bananeira; maximumElevationInMeters: 189; verbatimCoordinates: 4°5'59"S, 41°40'48"W; **Identification:** identifiedBy: Ângelo Parise Pinto; **Event:** samplingProtocol: Manual; verbatimEventDate: 13.ii.13; **Record Level:** institutionCode: DZRJ; basisOfRecord: PreservedSpecimen
- **Type status:** Other material. **Occurrence:** recordedBy: Santos, A.P.M. | Takiya, D.M.; individualCount: 1; sex: female; lifeStage: adult; **Location:** country: Brazil; stateProvince: Piauí; municipality: Piracuruca; locality: Parque Nacional de Sete Cidades, Riacho da Bananeira; maximumElevationInMeters: 189; verbatimCoordinates: 4°5'59"S, 41°40'48"W; **Identification:** identifiedBy: Ângelo Parise Pinto; **Event:** samplingProtocol: Manual; verbatimEventDate: 13.ii.13; **Record Level:** institutionCode: CZMA; basisOfRecord: PreservedSpecimen
- **Type status:** Other material. **Occurrence:** recordedBy: Santos, A.P.M. | Takiya, D.M.; individualCount: 1; sex: male; lifeStage: adult; **Location:** country: Brazil; stateProvince: Piauí; municipality: Piracuruca; locality: Parque Nacional de Sete Cidades, Riacho da Bananeira; maximumElevationInMeters: 189; verbatimCoordinates: 4°5'59"S, 41°40'48"W; **Identification:** identifiedBy: Ângelo Parise Pinto; **Event:** samplingProtocol: Manual; verbatimEventDate: 8.ii.13; **Record Level:** institutionCode: DZRJ; basisOfRecord: PreservedSpecimen

##### Distribution

Costa Rica. Panama. Colombia. Venezuela. Guyana. Suriname. Frech Guiana. Brazil: PA, AM, PI!, CE, BA, MG, MS, ES, RJ. Ecuador. Peru. Bolivia. Paraguay. Argentina.

##### Notes

New species record for PI.

#### Orthemis flavopicta

Kirby, 1889

##### Materials

- **Type status:** Other material. **Occurrence:** recordedBy: Santos, A.P.M. | Takiya, D.M.; individualCount: 1; sex: female; lifeStage: adult; **Location:** country: Brazil; stateProvince: Piauí; municipality: Piracuruca; locality: Parque Nacional de Sete Cidades, Olho d'água Piscina do Bacuri; maximumElevationInMeters: 171; verbatimCoordinates: 4°6'1.2"S, 41°42'38.8"W; **Identification:** identifiedBy: Ângelo Parise Pinto; **Event:** samplingProtocol: Pennsylvania light trap; verbatimEventDate: 8.ii.13; **Record Level:** institutionCode: DZRJ; basisOfRecord: PreservedSpecimen

##### Distribution

Venezuela. Brazil: PA, PI!, CE, RO, BA, GO. Bolivia.

##### Notes

New species record for PI. See Fig. 28.

#### Pantala

Hagen, 1861

##### Notes

New genus record for PI.

#### Pantala flavescens

(Fabricius, 1798)

##### Materials

- **Type status:** Other material. **Occurrence:** recordedBy: Santos, A.P.M. | Takiya, D.M.; individualCount: 1; sex: male; lifeStage: adult; **Location:** country: Brazil; stateProvince: Piauí; municipality: Piracuruca; locality: Parque Nacional de Sete Cidades, Olho d'água Piscina do Bacuri; maximumElevationInMeters: 171; verbatimCoordinates: 4°6'1.2"S, 41°42'38.8"W; **Identification:** identifiedBy: Ângelo Parise Pinto; **Event:** samplingProtocol: Manual; verbatimEventDate: 10.ii.13; **Record Level:** institutionCode: DZRJ; basisOfRecord: PreservedSpecimen
- **Type status:** Other material. **Occurrence:** recordedBy: Santos, A.P.M. | Takiya, D.M.; individualCount: 1; sex: female; lifeStage: adult; **Location:** country: Brazil; stateProvince: Piauí; municipality: Piracuruca; locality: Parque Nacional de Sete Cidades, Olho d'água Piscina do Bacuri; maximumElevationInMeters: 171; verbatimCoordinates: 4°6'1.2"S, 41°42'38.8"W; **Identification:** identifiedBy: Ângelo Parise Pinto; **Event:** samplingProtocol: Manual; verbatimEventDate: 10.ii.13; **Record Level:** institutionCode: CZMA; basisOfRecord: PreservedSpecimen
- **Type status:** Other material. **Occurrence:** recordedBy: Santos, A.P.M. | Takiya, D.M.; individualCount: 1; sex: male; lifeStage: adult; **Location:** country: Brazil; stateProvince: Piauí; municipality: Piracuruca; locality: Parque Nacional de Sete Cidades, Riacho da Bananeira; maximumElevationInMeters: 189; verbatimCoordinates: 4°5'59"S, 41°40'48"W; **Identification:** identifiedBy: Ângelo Parise Pinto; **Event:** samplingProtocol: Manual; verbatimEventDate: 13.ii.13; **Record Level:** institutionCode: CZMA; basisOfRecord: PreservedSpecimen
- **Type status:** Other material. **Occurrence:** recordedBy: Santos, A.P.M. | Takiya, D.M.; individualCount: 1; sex: male; lifeStage: adult; **Location:** country: Brazil; stateProvince: Piauí; municipality: Piracuruca; locality: Parque Nacional de Sete Cidades, Riacho da Bananeira; maximumElevationInMeters: 189; verbatimCoordinates: 4°5'59"S, 41°40'48"W; **Identification:** identifiedBy: Ângelo Parise Pinto; **Event:** samplingProtocol: Manual; verbatimEventDate: 13.ii.13; **Record Level:** institutionCode: CZMA; basisOfRecord: PreservedSpecimen
- **Type status:** Other material. **Occurrence:** recordedBy: Santos, A.P.M. | Takiya, D.M.; individualCount: 1; sex: male; lifeStage: adult; **Location:** country: Brazil; stateProvince: Piauí; municipality: Piracuruca; locality: Parque Nacional de Sete Cidades, Riacho da Bananeira; maximumElevationInMeters: 189; verbatimCoordinates: 4°5'59"S, 41°40'48"W; **Identification:** identifiedBy: Ângelo Parise Pinto; **Event:** samplingProtocol: Manual; verbatimEventDate: 13.ii.13; **Record Level:** institutionCode: DZRJ; basisOfRecord: PreservedSpecimen
- **Type status:** Other material. **Occurrence:** recordedBy: Santos, A.P.M. | Takiya, D.M.; individualCount: 1; sex: male; lifeStage: adult; **Location:** country: Brazil; stateProvince: Piauí; municipality: Piracuruca; locality: Parque Nacional de Sete Cidades, Riacho da Bananeira; maximumElevationInMeters: 189; verbatimCoordinates: 4°5'59"S, 41°40'48"W; **Identification:** identifiedBy: Ângelo Parise Pinto; **Event:** samplingProtocol: Manual; verbatimEventDate: 13.ii.13; **Record Level:** institutionCode: DZRJ; basisOfRecord: PreservedSpecimen
- **Type status:** Other material. **Occurrence:** recordedBy: Santos, A.P.M. | Takiya, D.M.; individualCount: 1; sex: male; lifeStage: adult; **Location:** country: Brazil; stateProvince: Piauí; municipality: Piracuruca; locality: Parque Nacional de Sete Cidades, Riacho da Bananeira; maximumElevationInMeters: 189; verbatimCoordinates: 4°5'59"S, 41°40'48"W; **Identification:** identifiedBy: Ângelo Parise Pinto; **Event:** samplingProtocol: Manual; verbatimEventDate: 13.ii.13; **Record Level:** institutionCode: DZRJ; basisOfRecord: PreservedSpecimen
- **Type status:** Other material. **Occurrence:** recordedBy: Santos, A.P.M. | Takiya, D.M.; individualCount: 1; sex: male; lifeStage: adult; **Location:** country: Brazil; stateProvince: Piauí; municipality: Piracuruca; locality: Parque Nacional de Sete Cidades, Riacho da Bananeira; maximumElevationInMeters: 189; verbatimCoordinates: 4°5'59"S, 41°40'48"W; **Identification:** identifiedBy: Ângelo Parise Pinto; **Event:** samplingProtocol: Manual; verbatimEventDate: 13.ii.13; **Record Level:** institutionCode: DZRJ; basisOfRecord: PreservedSpecimen
- **Type status:** Other material. **Occurrence:** recordedBy: Santos, A.P.M. | Takiya, D.M.; individualCount: 1; sex: male; lifeStage: adult; **Location:** country: Brazil; stateProvince: Piauí; municipality: Piracuruca; locality: Parque Nacional de Sete Cidades, Alojamento; maximumElevationInMeters: 193; verbatimCoordinates: 4°5'57"S, 41°42'34"W; **Identification:** identifiedBy: Ângelo Parise Pinto; **Event:** samplingProtocol: Manual; verbatimEventDate: 13.ii.13; **Record Level:** institutionCode: DZRJ; basisOfRecord: PreservedSpecimen
- **Type status:** Other material. **Occurrence:** recordedBy: Takiya, D.M. | Cavichioli, R.R.; individualCount: 1; sex: male; lifeStage: adult; **Location:** country: Brazil; stateProvince: Piauí; municipality: Piracuruca; locality: Parque Nacional de Sete Cidades, Riacho da Piedade; maximumElevationInMeters: 169; verbatimCoordinates: 4°6'34"S, 41°43'39"W; **Identification:** identifiedBy: Alcimar Carvalho | Ângelo Parise Pinto; **Event:** samplingProtocol: Manual; verbatimEventDate: 19.iv.12; **Record Level:** institutionCode: DZRJ; basisOfRecord: PreservedSpecimen
- **Type status:** Other material. **Occurrence:** recordedBy: Santos, A.P.M. | Takiya, D.M.; individualCount: 1; sex: male; lifeStage: adult; **Location:** country: Brazil; stateProvince: Piauí; municipality: Piracuruca; locality: Parque Nacional de Sete Cidades, Riacho da Bananeira; maximumElevationInMeters: 189; verbatimCoordinates: 4°5'59"S, 41°40'48"W; **Identification:** identifiedBy: Ângelo Parise Pinto; **Event:** samplingProtocol: Manual; verbatimEventDate: 8.ii.13; **Record Level:** institutionCode: DZRJ; basisOfRecord: PreservedSpecimen
- **Type status:** Other material. **Occurrence:** recordedBy: Santos, A.P.M. | Takiya, D.M.; individualCount: 1; sex: female; lifeStage: adult; **Location:** country: Brazil; stateProvince: Piauí; municipality: Piracuruca; locality: Parque Nacional de Sete Cidades, Olho d'água Piscina do Bacuri; maximumElevationInMeters: 171; verbatimCoordinates: 4°6'1.2"S, 41°42'38.8"W; **Identification:** identifiedBy: Ângelo Parise Pinto; **Event:** samplingProtocol: Manual; verbatimEventDate: 9.ii.13; **Record Level:** institutionCode: CZMA; basisOfRecord: PreservedSpecimen

##### Distribution

Pantropical. Widespread in the Americas. Brazil: RR, PA, AM, MA, PI!, CE, TO, PE, MT, RO, BA, MG, MS, ES, SP, RJ, PR, SC, RS, also in Fernando de Noronha and Trindade e Martim Vaz archipelagoes.

##### Notes

New species record for PI.

#### Perithemis

Hagen, 1861

#### Perithemis lais

(Perty, 1834)

##### Materials

- **Type status:** Other material. **Occurrence:** recordedBy: Rafael, J.A. | Limeira-de-Oliveira, F. | Takiya, D.M. | et al.; individualCount: 1; sex: male; lifeStage: adult; **Location:** country: Brazil; stateProvince: Piauí; municipality: Piracuruca; locality: Parque Nacional de Sete Cidades, Alojamento; maximumElevationInMeters: 193; verbatimCoordinates: 4°5'57"S, 41°42'34"W; **Identification:** identifiedBy: Ângelo Parise Pinto; **Event:** samplingProtocol: Malaise intercept trap; verbatimEventDate: 19.iv.12; **Record Level:** institutionCode: DZRJ; basisOfRecord: PreservedSpecimen
- **Type status:** Other material. **Occurrence:** recordedBy: Santos, A.P.M. | Takiya, D.M.; individualCount: 1; sex: male; lifeStage: adult; **Location:** country: Brazil; stateProvince: Piauí; municipality: Piracuruca; locality: Parque Nacional de Sete Cidades, Olha d'água dos Milagres; maximumElevationInMeters: 180; verbatimCoordinates: 4°5'31.8"S, 41°40'48.2"W; **Identification:** identifiedBy: Ângelo Parise Pinto; **Event:** samplingProtocol: White sheet light trap; verbatimEventDate: 9.ii.13; **Record Level:** institutionCode: DZRJ; basisOfRecord: PreservedSpecimen

##### Distribution

Trinidad and Tobago. Colombia. Venezuela. Guyana. Suriname. French Guiana. Brazil: RR, PA, AM, MA, PI!, PE, MT, RO, MS, ES, SP. Ecuador. Peru. Bolivia. Argentina.

##### Notes

New species record for PI.

#### Zygoptera

#### Calopterygidae

#### Hetaerina

Hagen in Selys, 1853

##### Notes

New genus record for PI.

#### Hetaerina sp. 1

##### Materials

- **Type status:** Other material. **Occurrence:** recordedBy: Rafael, J.A. | Limeira-de-Oliveira, F. | Takiya, D.M. | et al.; individualCount: 1; sex: female; lifeStage: adult; **Location:** country: Brazil; stateProvince: Piauí; municipality: Piracuruca; locality: Parque Nacional de Sete Cidades, Alojamento; maximumElevationInMeters: 193; verbatimCoordinates: 4°5'57"S, 41°42'34"W; **Identification:** identifiedBy: Ângelo Parise Pinto; **Event:** samplingProtocol: Malaise intercept trap; verbatimEventDate: 19.iv.12; **Record Level:** institutionCode: DZRJ; basisOfRecord: PreservedSpecimen

#### Coenagrionidae

#### Acanthagrion

Selys, 1876

##### Notes

New genus record for PI.

#### Acanthagrion jessei

Leonard, 1977

##### Materials

- **Type status:** Other material. **Occurrence:** recordedBy: Limeira-de-Oliveira, F. | Pinto Júnior, J.S.; individualCount: 1; sex: male; lifeStage: adult; **Location:** country: Brazil; stateProvince: Piauí; municipality: Piracuruca; locality: Parque Nacional de Sete Cidades, Alojamento; maximumElevationInMeters: 193; verbatimCoordinates: 4°5'57"S, 41°42'34"W; **Identification:** identifiedBy: Ângelo Parise Pinto; **Event:** samplingProtocol: Malaise intercept trap; verbatimEventDate: 16.xii.12; **Record Level:** institutionCode: DZRJ; basisOfRecord: PreservedSpecimen
- **Type status:** Other material. **Occurrence:** recordedBy: Rafael, J.A. | Limeira-de-Oliveira, F. | Takiya, D.M. | et al.; individualCount: 1; sex: female; lifeStage: adult; **Location:** country: Brazil; stateProvince: Piauí; municipality: Piracuruca; locality: Parque Nacional de Sete Cidades, Riacho da Bananeira; maximumElevationInMeters: 189; verbatimCoordinates: 4°5'59"S, 41°40'48"W; **Identification:** identifiedBy: Ângelo Parise Pinto; **Event:** samplingProtocol: Malaise intercept trap; verbatimEventDate: 19.iv.12; **Record Level:** institutionCode: DZRJ; basisOfRecord: PreservedSpecimen
- **Type status:** Other material. **Occurrence:** recordedBy: Rafael, J.A. | Limeira-de-Oliveira, F. | et al.; individualCount: 1; sex: male; lifeStage: adult; **Location:** country: Brazil; stateProvince: Piauí; municipality: Piracuruca; locality: Parque Nacional de Sete Cidades, Alojamento; maximumElevationInMeters: 193; verbatimCoordinates: 4°5'57"S, 41°42'34"W; **Identification:** identifiedBy: Ângelo Parise Pinto; **Event:** samplingProtocol: Suspended intercept trap; verbatimEventDate: 19.iv.12; **Record Level:** institutionCode: DZRJ; basisOfRecord: PreservedSpecimen
- **Type status:** Other material. **Occurrence:** recordedBy: Rafael, J.A. | Limeira-de-Oliveira, F. | Takiya, D.M. | et al.; individualCount: 2; sex: male; lifeStage: adult; **Location:** country: Brazil; stateProvince: Piauí; municipality: Piracuruca; locality: Parque Nacional de Sete Cidades, Riacho da Piedade; maximumElevationInMeters: 169; verbatimCoordinates: 4°6'34"S, 41°43'39"W; **Identification:** identifiedBy: Ângelo Parise Pinto; **Event:** samplingProtocol: Malaise intercept trap; verbatimEventDate: 21.iv.12; **Record Level:** institutionCode: DZRJ; basisOfRecord: PreservedSpecimen
- **Type status:** Other material. **Occurrence:** recordedBy: Rafael, J.A. | Limeira-de-Oliveira, F. | Takiya, D.M. | et al.; individualCount: 3; sex: female; lifeStage: adult; **Location:** country: Brazil; stateProvince: Piauí; municipality: Piracuruca; locality: Parque Nacional de Sete Cidades, Riacho da Piedade; maximumElevationInMeters: 169; verbatimCoordinates: 4°6'34"S, 41°43'39"W; **Identification:** identifiedBy: Ângelo Parise Pinto; **Event:** samplingProtocol: Malaise intercept trap; verbatimEventDate: 21.iv.12; **Record Level:** institutionCode: DZRJ; basisOfRecord: PreservedSpecimen

##### Distribution

Brazil: PI!, MT, RO, MS. Ecuador.

##### Notes

New species record for PI.

#### Acanthagrion truncatum

Selys, 1876

##### Materials

- **Type status:** Other material. **Occurrence:** recordedBy: Santos, A.P.M. | Takiya, D.M.; individualCount: 1; sex: male; lifeStage: adult; **Location:** country: Brazil; stateProvince: Piauí; municipality: Piracuruca; locality: Parque Nacional de Sete Cidades, Poço do Bananeira; maximumElevationInMeters: 158; verbatimCoordinates: 4°5'55.8"S, 41°40'33.8"W; **Identification:** identifiedBy: Ângelo Parise Pinto; **Event:** samplingProtocol: Manual; verbatimEventDate: 11.ii.13; **Record Level:** institutionCode: DZRJ; basisOfRecord: PreservedSpecimen
- **Type status:** Other material. **Occurrence:** recordedBy: Santos, A.P.M. | Takiya, D.M.; individualCount: 1; sex: male; lifeStage: adult; **Location:** country: Brazil; stateProvince: Piauí; municipality: Piracuruca; locality: Parque Nacional de Sete Cidades, Poço do Bananeira; maximumElevationInMeters: 158; verbatimCoordinates: 4°5'55.8"S, 41°40'33.8"W; **Identification:** identifiedBy: Ângelo Parise Pinto; **Event:** samplingProtocol: Manual; verbatimEventDate: 9.ii.13; **Record Level:** institutionCode: DZRJ; basisOfRecord: PreservedSpecimen

##### Distribution

Venezuela. Guyana. Brazil: PI!, TO, MT, GO, MG, MS, SP.

##### Notes

New species record for PI.

#### Argia

Rambur, 1842

##### Notes

New genus record for PI.

#### Argia tinctipennis

Selys, 1865

##### Materials

- **Type status:** Other material. **Occurrence:** recordedBy: Rafael, J.A. | Limeira-de-Oliveira, F. | et al.; individualCount: 1; sex: male; lifeStage: adult; **Location:** country: Brazil; stateProvince: Piauí; municipality: Piracuruca; locality: Parque Nacional de Sete Cidades, Cachoeira do Riachão; maximumElevationInMeters: 171; verbatimCoordinates: 4°6'28"S, 41°40'13"W; **Identification:** identifiedBy: Rosser W. Garrison; **Event:** samplingProtocol: Suspended intercept trap; verbatimEventDate: 17.ii.13; **Record Level:** institutionCode: DZRJ; basisOfRecord: PreservedSpecimen

##### Distribution

Brazil: AM, PI!, CE!, MT, GO, MS. Peru.

##### Notes

New species record for Northeastern Brazil.

#### Epipleoneura

Williamson, 1915

##### Notes

New genus record for PI.

#### Epipleoneura metallica

Rácenis, 1955

##### Materials

- **Type status:** Other material. **Occurrence:** recordedBy: Rafael, J.A. | Limeira-de-Oliveira, F. | Takiya, D.M. | et al.; individualCount: 1; sex: female; lifeStage: adult; **Location:** country: Brazil; stateProvince: Piauí; municipality: Piracuruca; locality: Parque Nacional de Sete Cidades, Alojamento; maximumElevationInMeters: 193; verbatimCoordinates: 4°5'57"S, 41°42'34"W; **Identification:** identifiedBy: Ângelo Parise Pinto; **Event:** samplingProtocol: Malaise intercept trap; verbatimEventDate: 19.iv.12; **Record Level:** institutionCode: DZRJ; basisOfRecord: PreservedSpecimen

##### Distribution

Colombia. Venezuela. Brazil: PA, AM, MA, PI!, CE, TO, PE, MT, RO, BA, GO, MG, DF, ES.

##### Notes

New species record for PI.

#### Ischnura

##### Notes

New genus record for PI.

#### Ischnura capreolus

(Hagen, 1861)

##### Materials

- **Type status:** Other material. **Occurrence:** recordedBy: Santos, A.P.M. | Takiya, D.M.; individualCount: 1; sex: male; lifeStage: adult; **Location:** country: Brazil; stateProvince: Piauí; municipality: Piracuruca; locality: Parque Nacional de Sete Cidades, Poço do Bananeira; maximumElevationInMeters: 158; verbatimCoordinates: 4°5'55.8"S, 41°40'33.8"W; **Identification:** identifiedBy: Ângelo Parise Pinto; **Event:** samplingProtocol: Manual; verbatimEventDate: 11.ii.13; **Record Level:** institutionCode: CZMA; basisOfRecord: PreservedSpecimen
- **Type status:** Other material. **Occurrence:** recordedBy: Santos, A.P.M. | Takiya, D.M.; individualCount: 1; sex: male; lifeStage: adult; **Location:** country: Brazil; stateProvince: Piauí; municipality: Piracuruca; locality: Parque Nacional de Sete Cidades, Poço do Bananeira; maximumElevationInMeters: 158; verbatimCoordinates: 4°5'55.8"S, 41°40'33.8"W; **Identification:** identifiedBy: Ângelo Parise Pinto; **Event:** samplingProtocol: Manual; verbatimEventDate: 11.ii.13; **Record Level:** institutionCode: DZRJ; basisOfRecord: PreservedSpecimen

##### Distribution

Mexico south to Panama. Trinidad and Tobago. Colombia. Venezuela. Guyana. Suriname. French Guiana. Brazil: PA, AM, PI!, CE, PB, PE, MT, RO, BA, MG, MS, ES, SP, RJ, RS. Ecuador. Peru. Bolivia. Paraguay. Argentina.

##### Notes

New species record for PI.

#### Neoneura

Selys, 1860

##### Notes

New genus record for PI.

#### Neoneura sylvatica

Hagen in Selys, 1886

##### Materials

- **Type status:** Other material. **Occurrence:** recordedBy: Limeira-de-Oliveira, F. | Pinto Júnior, J.S.; individualCount: 1; sex: female; lifeStage: adult; **Location:** country: Brazil; stateProvince: Piauí; municipality: Piracuruca; locality: Parque Nacional de Sete Cidades, Alojamento; maximumElevationInMeters: 193; verbatimCoordinates: 4°5'57"S, 41°42'34"W; **Identification:** identifiedBy: Ângelo Parise Pinto; **Event:** samplingProtocol: Malaise intercept trap; verbatimEventDate: 16.xii.12; **Record Level:** institutionCode: DZRJ; basisOfRecord: PreservedSpecimen

##### Distribution

Colombia. Venezuela. Suriname. French Guiana. Brazil: MA, PI!, CE, TO, PE, MT, RO, BA, GO, MG, MS, SP, RJ, PR. Bolivia. Argentina.

##### Notes

New species record for PI.

#### Oxyagrion

Selys, 1876

##### Notes

New genus record for PI.

#### Oxyagrion sp. 1 [O. basale group]

##### Materials

- **Type status:** Other material. **Occurrence:** recordedBy: Rafael, J.A. | Limeira-de-Oliveira, F. | et al.; individualCount: 1; sex: male; lifeStage: adult; **Location:** country: Brazil; stateProvince: Piauí; municipality: Piracuruca; locality: Parque Nacional de Sete Cidades, Alojamento; maximumElevationInMeters: 193; verbatimCoordinates: 4°5'57"S, 41°42'34"W; **Identification:** identifiedBy: Ângelo Parise Pinto; **Event:** samplingProtocol: Suspended intercept trap; verbatimEventDate: 19.iv.12; **Record Level:** institutionCode: DZRJ; basisOfRecord: PreservedSpecimen

##### Notes

Likely an undescribed species.

#### Plecoptera

##### Notes

New order record for PI.

#### Perlidae

##### Notes

New family record for PI.

#### Anacroneuria

Klapálek, 1909

##### Notes

New genus record for PI.

#### Anacroneuria calori

Duarte & Lecci, 2016

##### Materials

- **Type status:** Other material. **Occurrence:** recordedBy: Takiya, D.M.; individualCount: 1; sex: female; lifeStage: adult; **Location:** country: Brazil; stateProvince: Piauí; municipality: Piracuruca; locality: Parque Nacional de Sete Cidades, Cachoeira do Riachão; maximumElevationInMeters: 171; verbatimCoordinates: 4°6'28"S, 41°40'13"W; **Identification:** identifiedBy: Fernanda Avelino-Capistrano; **Event:** samplingProtocol: Pennsylvania light trap; verbatimEventDate: 19.iv.12; **Record Level:** institutionCode: DZRJ; basisOfRecord: PreservedSpecimen
- **Type status:** Other material. **Occurrence:** recordedBy: Takiya, D.M.; individualCount: 1; sex: male; lifeStage: adult; **Location:** country: Brazil; stateProvince: Piauí; municipality: Piracuruca; locality: Parque Nacional de Sete Cidades, Cachoeira do Riachão; maximumElevationInMeters: 171; verbatimCoordinates: 4°6'28"S, 41°40'13"W; **Identification:** identifiedBy: Fernanda Avelino-Capistrano; **Event:** samplingProtocol: Pennsylvania light trap; verbatimEventDate: 19.iv.12; **Record Level:** institutionCode: DZRJ; basisOfRecord: PreservedSpecimen
- **Type status:** Other material. **Occurrence:** recordedBy: Takiya, D.M. | Rafael, J.A.; individualCount: 1; sex: male; lifeStage: adult; **Location:** country: Brazil; stateProvince: Piauí; municipality: Piracuruca; locality: Parque Nacional de Sete Cidades, Cachoeira do Riachão; maximumElevationInMeters: 171; verbatimCoordinates: 4°6'28"S, 41°40'13"W; **Identification:** identifiedBy: Fernanda Avelino-Capistrano; **Event:** samplingProtocol: Pennsylvania light trap; verbatimEventDate: 20.iv.12; **Record Level:** institutionCode: DZRJ; basisOfRecord: PreservedSpecimen
- **Type status:** Other material. **Occurrence:** recordedBy: Takiya, D.M. | Rafael, J.A.; individualCount: 1; sex: male; lifeStage: adult; **Location:** country: Brazil; stateProvince: Piauí; municipality: Piracuruca; locality: Parque Nacional de Sete Cidades, Cachoeira do Riachão; maximumElevationInMeters: 171; verbatimCoordinates: 4°6'28"S, 41°40'13"W; **Identification:** identifiedBy: Fernanda Avelino-Capistrano; **Event:** samplingProtocol: Pennsylvania light trap; verbatimEventDate: 20.iv.12; **Record Level:** institutionCode: DZRJ; basisOfRecord: PreservedSpecimen

##### Distribution

Brazil: CE, PI!.

##### Notes

**Figure 25.**
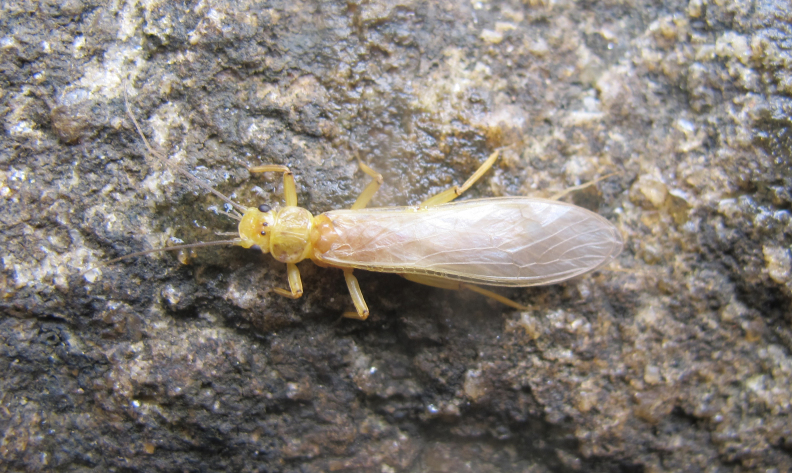
Teneral adult specimen of *Anacroneuria calori* collected at PNU-02. This species was also recorded from PNSC, representing the first record of Plecoptera for Piauí.

**Figure 26.**
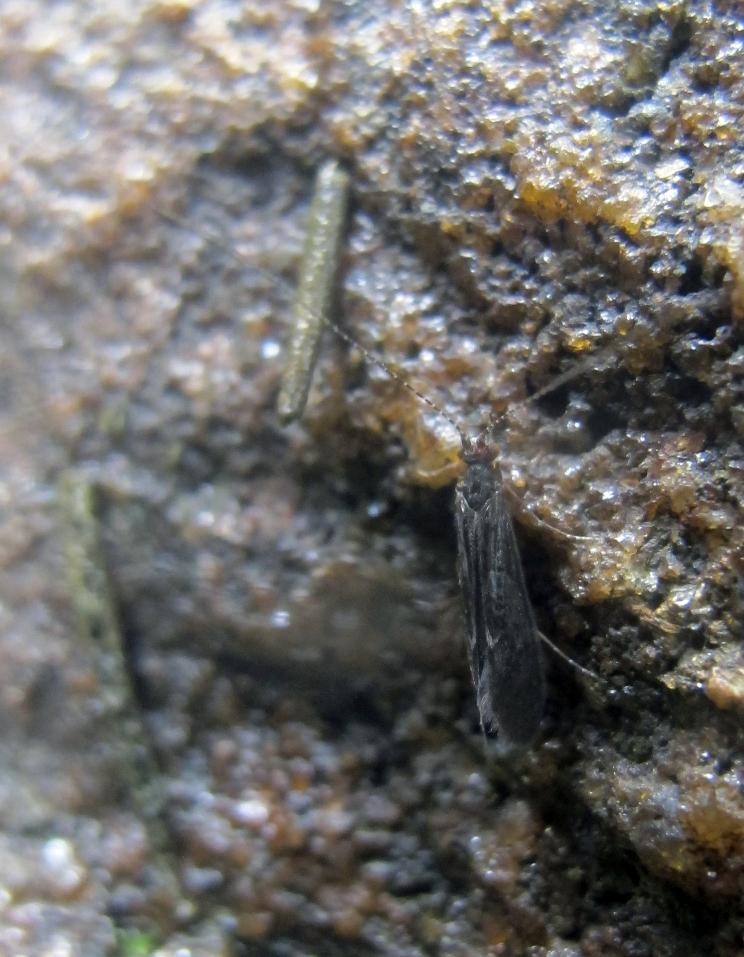
Adult and immature specimens of *Atanatolica nordestina* collected at PNU-04, species described based on material collected during this project.

**Figure 27.**
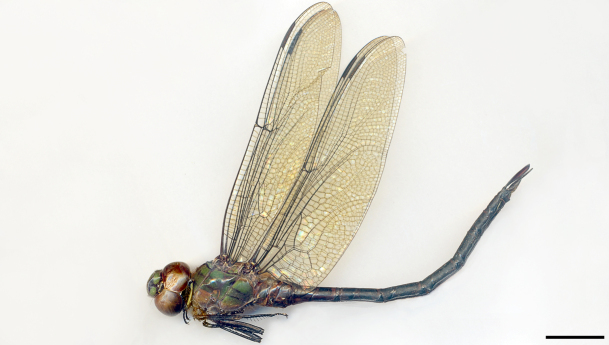
Lateral habitus of a male of *Coryphaeschna viriditas* collected at PNSC-06 (DZRJ), representing a new state record for Piauí. Scale bar = 10 mm.

**Figure 28.**
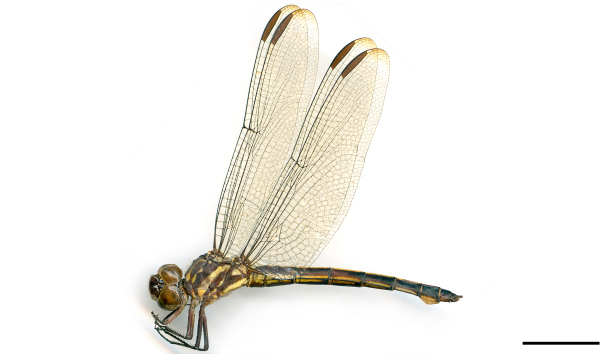
Lateral habitus of a female of *Orthemis flavopicta* collected at PNSC-06 (DZRJ), representing a new state record for Piauí. Scale bar = 10 mm.

New species record for PI. See Fig. 25.

#### Trichoptera

#### Ecnomidae

##### Notes

New family record for PI.

#### Austrotinodes

Schmid, 1955

##### Notes

New genus record for PI.

#### Austrotinodes paraguayensis

Flint, 1983

##### Materials

- **Type status:** Other material. **Occurrence:** recordedBy: Rafael, J.A. | Limeira-de-Oliveira, F. | Takiya, D.M. | et al.; individualCount: 1; sex: male; lifeStage: adult; **Location:** country: Brazil; stateProvince: Piauí; municipality: Piracuruca; locality: Parque Nacional de Sete Cidades, Riacho da Bananeira; maximumElevationInMeters: 189; verbatimCoordinates: 4°5'59"S, 41°40'48"W; **Identification:** identifiedBy: Wagner Rafael Maciel de Souza; **Event:** samplingProtocol: Malaise intercept trap; verbatimEventDate: 19.iv.12; **Record Level:** institutionCode: DZRJ; basisOfRecord: PreservedSpecimen

##### Distribution

Brazil: PI!, CE!, MG. Paraguay.

##### Notes

New species record for Northeastern Brazil.

#### Helicopsychidae

#### Helicopsyche

von Siebold, 1856

#### Helicopsyche (Feropsyche) vergelana

Ross, 1956

##### Materials

- **Type status:** Other material. **Occurrence:** recordedBy: Takiya, D.M.; individualCount: 1; sex: male; lifeStage: adult; **Location:** country: Brazil; stateProvince: Piauí; municipality: Piracuruca; locality: Parque Nacional de Sete Cidades, Cachoeira do Riachão; maximumElevationInMeters: 171; verbatimCoordinates: 4°6'28"S, 41°40'13"W; **Identification:** identifiedBy: Allan Paulo Moreira dos Santos; **Event:** samplingProtocol: Pennsylvania light trap; verbatimEventDate: 18.iv.12; **Record Level:** institutionCode: DZRJ; basisOfRecord: PreservedSpecimen
- **Type status:** Other material. **Occurrence:** recordedBy: Takiya, D.M.; individualCount: 1; sex: female; lifeStage: adult; **Location:** country: Brazil; stateProvince: Piauí; municipality: Piracuruca; locality: Parque Nacional de Sete Cidades, Cachoeira do Riachão; maximumElevationInMeters: 171; verbatimCoordinates: 4°6'28"S, 41°40'13"W; **Identification:** identifiedBy: Allan Paulo Moreira dos Santos; **Event:** samplingProtocol: Pennsylvania light trap; verbatimEventDate: 18.iv.12; **Record Level:** institutionCode: DZRJ; basisOfRecord: PreservedSpecimen
- **Type status:** Other material. **Occurrence:** recordedBy: Rafael, J.A. | Limeira-de-Oliveira, F. | Takiya, D.M. | et al.; individualCount: 1; sex: male; lifeStage: adult; **Location:** country: Brazil; stateProvince: Piauí; municipality: Piracuruca; locality: Parque Nacional de Sete Cidades, Riacho da Piedade; maximumElevationInMeters: 169; verbatimCoordinates: 4°6'34"S, 41°43'39"W; **Identification:** identifiedBy: Allan Paulo Moreira dos Santos; **Event:** samplingProtocol: Malaise intercept trap; verbatimEventDate: 18.iv.12; **Record Level:** institutionCode: DZRJ; basisOfRecord: PreservedSpecimen
- **Type status:** Other material. **Occurrence:** recordedBy: Rafael, J.A. | Limeira-de-Oliveira, F. | Takiya, D.M. | et al.; individualCount: 3; sex: male; lifeStage: adult; **Location:** country: Brazil; stateProvince: Piauí; municipality: Piracuruca; locality: Parque Nacional de Sete Cidades, Riacho da Piedade; maximumElevationInMeters: 169; verbatimCoordinates: 4°6'34"S, 41°43'39"W; **Identification:** identifiedBy: Allan Paulo Moreira dos Santos; **Event:** samplingProtocol: Malaise intercept trap; verbatimEventDate: 21.iv.12; **Record Level:** institutionCode: DZRJ; basisOfRecord: PreservedSpecimen
- **Type status:** Other material. **Occurrence:** recordedBy: Rafael, J.A. | Limeira-de-Oliveira, F. | Takiya, D.M. | et al.; individualCount: 13; sex: male; lifeStage: adult; **Location:** country: Brazil; stateProvince: Piauí; municipality: Piracuruca; locality: Parque Nacional de Sete Cidades, Riacho da Piedade; maximumElevationInMeters: 169; verbatimCoordinates: 4°6'34"S, 41°43'39"W; **Identification:** identifiedBy: Allan Paulo Moreira dos Santos; **Event:** samplingProtocol: Malaise intercept trap; verbatimEventDate: 21.iv.12; **Record Level:** institutionCode: DZRJ; basisOfRecord: PreservedSpecimen
- **Type status:** Other material. **Occurrence:** recordedBy: Rafael, J.A. | Limeira-de-Oliveira, F. | Takiya, D.M. | et al.; individualCount: 2; sex: female; lifeStage: adult; **Location:** country: Brazil; stateProvince: Piauí; municipality: Piracuruca; locality: Parque Nacional de Sete Cidades, Riacho da Piedade; maximumElevationInMeters: 169; verbatimCoordinates: 4°6'34"S, 41°43'39"W; **Identification:** identifiedBy: Allan Paulo Moreira dos Santos; **Event:** samplingProtocol: Malaise intercept trap; verbatimEventDate: 21.iv.12; **Record Level:** institutionCode: DZRJ; basisOfRecord: PreservedSpecimen

##### Distribution

Mexico. Belize. Guatemala. Honduras. Costa Rica. Panama. Trinidad and Tobago. Venezuela. Suriname. Brazil: PI!, CE!, PE. Peru.

##### Notes

New species record for PI.

#### Hydropsychidae

##### Notes

New family record for PI.

#### Leptonema

Guérin, 1843

##### Notes

New genus record for PI.

#### Leptonema viridianum

Navás, 1916

##### Materials

- **Type status:** Other material. **Occurrence:** recordedBy: Rafael, J.A. | Limeira-de-Oliveira, F. | Takiya, D.M. | et al.; individualCount: 2; sex: male; lifeStage: adult; **Location:** country: Brazil; stateProvince: Piauí; municipality: Piracuruca; locality: Parque Nacional de Sete Cidades, Riacho da Piedade; maximumElevationInMeters: 169; verbatimCoordinates: 4°6'34"S, 41°43'39"W; **Identification:** identifiedBy: Allan Paulo Moreira dos Santos; **Event:** samplingProtocol: Malaise intercept trap; verbatimEventDate: 18.iv.12; **Record Level:** institutionCode: DZRJ; basisOfRecord: PreservedSpecimen
- **Type status:** Other material. **Occurrence:** recordedBy: Rafael, J.A. | Limeira-de-Oliveira, F. | Takiya, D.M. | et al.; individualCount: 3; sex: male; lifeStage: adult; **Location:** country: Brazil; stateProvince: Piauí; municipality: Piracuruca; locality: Parque Nacional de Sete Cidades, Riacho da Piedade; maximumElevationInMeters: 169; verbatimCoordinates: 4°6'34"S, 41°43'39"W; **Identification:** identifiedBy: Allan Paulo Moreira dos Santos; **Event:** samplingProtocol: Malaise intercept trap; verbatimEventDate: 21.iv.12; **Record Level:** institutionCode: DZRJ; basisOfRecord: PreservedSpecimen
- **Type status:** Other material. **Occurrence:** recordedBy: Rafael, J.A. | Limeira-de-Oliveira, F. | Takiya, D.M. | et al.; individualCount: 4; sex: female; lifeStage: adult; **Location:** country: Brazil; stateProvince: Piauí; municipality: Piracuruca; locality: Parque Nacional de Sete Cidades, Riacho da Piedade; maximumElevationInMeters: 169; verbatimCoordinates: 4°6'34"S, 41°43'39"W; **Identification:** identifiedBy: Allan Paulo Moreira dos Santos; **Event:** samplingProtocol: Malaise intercept trap; verbatimEventDate: 21.iv.12; **Record Level:** institutionCode: DZRJ; basisOfRecord: PreservedSpecimen

##### Distribution

Colombia. Venezuela. Guyana. Brazil: PA, PI!, CE, GO, MG, DF, ES, RJ. Ecuador. Peru. Bolivia. Paraguay. Argentina.

##### Notes

New species record for PI.

#### Macronema

Pictet, 1836

##### Notes

New genus record for PI.

#### Macronema sp. 1

##### Materials

- **Type status:** Other material. **Occurrence:** recordedBy: Rafael, J.A. | Limeira-de-Oliveira, F. | Takiya, D.M. | et al.; individualCount: 0; sex: female; lifeStage: adult; **Location:** country: Brazil; stateProvince: Piauí; municipality: Piracuruca; locality: Parque Nacional de Sete Cidades, Riacho da Piedade; maximumElevationInMeters: 169; verbatimCoordinates: 4°6'34"S, 41°43'39"W; **Identification:** identifiedBy: Allan Paulo Moreira dos Santos; **Event:** samplingProtocol: Malaise intercept trap; verbatimEventDate: 18.iv.12; **Record Level:** institutionCode: DZRJ; basisOfRecord: PreservedSpecimen

#### Macrostemum

Kolenati, 1859

##### Notes

New genus record for PI.

#### Macrostemum ulmeri

(Banks, 1913)

##### Materials

- **Type status:** Other material. **Occurrence:** recordedBy: Rafael, J.A. | Limeira-de-Oliveira, F. | Takiya, D.M. | et al.; individualCount: 1; sex: male; lifeStage: adult; **Location:** country: Brazil; stateProvince: Piauí; municipality: Piracuruca; locality: Parque Nacional de Sete Cidades, Cachoeira do Riachão; maximumElevationInMeters: 171; verbatimCoordinates: 4°6'28"S, 41°40'13"W; **Identification:** identifiedBy: Allan Paulo Moreira dos Santos; **Event:** samplingProtocol: Malaise intercept trap; verbatimEventDate: 18.iv.12; **Record Level:** institutionCode: DZRJ; basisOfRecord: PreservedSpecimen
- **Type status:** Other material. **Occurrence:** recordedBy: Rafael, J.A. | Limeira-de-Oliveira, F. | Takiya, D.M. | et al.; individualCount: 1; sex: female; lifeStage: adult; **Location:** country: Brazil; stateProvince: Piauí; municipality: Piracuruca; locality: Parque Nacional de Sete Cidades, Cachoeira do Riachão; maximumElevationInMeters: 171; verbatimCoordinates: 4°6'28"S, 41°40'13"W; **Identification:** identifiedBy: Allan Paulo Moreira dos Santos; **Event:** samplingProtocol: Malaise intercept trap; verbatimEventDate: 18.iv.12; **Record Level:** institutionCode: DZRJ; basisOfRecord: PreservedSpecimen
- **Type status:** Other material. **Occurrence:** recordedBy: Rafael, J.A. | Limeira-de-Oliveira, F. | Takiya, D.M. | et al.; individualCount: 1; sex: male; lifeStage: adult; **Location:** country: Brazil; stateProvince: Piauí; municipality: Piracuruca; locality: Parque Nacional de Sete Cidades, Cachoeira do Riachão; maximumElevationInMeters: 171; verbatimCoordinates: 4°6'28"S, 41°40'13"W; **Identification:** identifiedBy: Allan Paulo Moreira dos Santos; **Event:** samplingProtocol: Malaise intercept trap; verbatimEventDate: 18.iv.12; **Record Level:** institutionCode: DZRJ; basisOfRecord: PreservedSpecimen
- **Type status:** Other material. **Occurrence:** recordedBy: Rafael, J.A. | Limeira-de-Oliveira, F. | Takiya, D.M. | et al.; individualCount: 4; sex: male; lifeStage: adult; **Location:** country: Brazil; stateProvince: Piauí; municipality: Piracuruca; locality: Parque Nacional de Sete Cidades, Riacho da Piedade; maximumElevationInMeters: 169; verbatimCoordinates: 4°6'34"S, 41°43'39"W; **Identification:** identifiedBy: Allan Paulo Moreira dos Santos; **Event:** samplingProtocol: Malaise intercept trap; verbatimEventDate: 18.iv.12; **Record Level:** institutionCode: DZRJ; basisOfRecord: PreservedSpecimen
- **Type status:** Other material. **Occurrence:** recordedBy: Rafael, J.A. | Limeira-de-Oliveira, F. | Takiya, D.M. | et al.; individualCount: 6; sex: female; lifeStage: adult; **Location:** country: Brazil; stateProvince: Piauí; municipality: Piracuruca; locality: Parque Nacional de Sete Cidades, Riacho da Piedade; maximumElevationInMeters: 169; verbatimCoordinates: 4°6'34"S, 41°43'39"W; **Identification:** identifiedBy: Allan Paulo Moreira dos Santos; **Event:** samplingProtocol: Malaise intercept trap; verbatimEventDate: 18.iv.12; **Record Level:** institutionCode: DZRJ; basisOfRecord: PreservedSpecimen
- **Type status:** Other material. **Occurrence:** recordedBy: Rafael, J.A. | Limeira-de-Oliveira, F. | Takiya, D.M. | et al.; individualCount: 22; sex: male; lifeStage: adult; **Location:** country: Brazil; stateProvince: Piauí; municipality: Piracuruca; locality: Parque Nacional de Sete Cidades, Riacho da Piedade; maximumElevationInMeters: 169; verbatimCoordinates: 4°6'34"S, 41°43'39"W; **Identification:** identifiedBy: Allan Paulo Moreira dos Santos; **Event:** samplingProtocol: Malaise intercept trap; verbatimEventDate: 18.iv.12; **Record Level:** institutionCode: DZRJ; basisOfRecord: PreservedSpecimen
- **Type status:** Other material. **Occurrence:** recordedBy: Rafael, J.A. | Limeira-de-Oliveira, F. | Takiya, D.M. | et al.; individualCount: 22; sex: female; lifeStage: adult; **Location:** country: Brazil; stateProvince: Piauí; municipality: Piracuruca; locality: Parque Nacional de Sete Cidades, Riacho da Piedade; maximumElevationInMeters: 169; verbatimCoordinates: 4°6'34"S, 41°43'39"W; **Identification:** identifiedBy: Allan Paulo Moreira dos Santos; **Event:** samplingProtocol: Malaise intercept trap; verbatimEventDate: 18.iv.12; **Record Level:** institutionCode: DZRJ; basisOfRecord: PreservedSpecimen
- **Type status:** Other material. **Occurrence:** recordedBy: Rafael, J.A. | Limeira-de-Oliveira, F. | Takiya, D.M. | et al.; individualCount: 22; sex: male; lifeStage: adult; **Location:** country: Brazil; stateProvince: Piauí; municipality: Piracuruca; locality: Parque Nacional de Sete Cidades, Riacho da Piedade; maximumElevationInMeters: 169; verbatimCoordinates: 4°6'34"S, 41°43'39"W; **Identification:** identifiedBy: Allan Paulo Moreira dos Santos; **Event:** samplingProtocol: Malaise intercept trap; verbatimEventDate: 18.iv.12; **Record Level:** institutionCode: DZRJ; basisOfRecord: PreservedSpecimen
- **Type status:** Other material. **Occurrence:** recordedBy: Rafael, J.A. | Limeira-de-Oliveira, F. | Takiya, D.M. | et al.; individualCount: 22; sex: female; lifeStage: adult; **Location:** country: Brazil; stateProvince: Piauí; municipality: Piracuruca; locality: Parque Nacional de Sete Cidades, Riacho da Piedade; maximumElevationInMeters: 169; verbatimCoordinates: 4°6'34"S, 41°43'39"W; **Identification:** identifiedBy: Allan Paulo Moreira dos Santos; **Event:** samplingProtocol: Malaise intercept trap; verbatimEventDate: 18.iv.12; **Record Level:** institutionCode: DZRJ; basisOfRecord: PreservedSpecimen
- **Type status:** Other material. **Occurrence:** recordedBy: Rafael, J.A. | Limeira-de-Oliveira, F. | Takiya, D.M. | et al.; individualCount: 2; sex: male; lifeStage: adult; **Location:** country: Brazil; stateProvince: Piauí; municipality: Piracuruca; locality: Parque Nacional de Sete Cidades, Riacho da Bananeira; maximumElevationInMeters: 189; verbatimCoordinates: 4°5'59"S, 41°40'48"W; **Identification:** identifiedBy: Allan Paulo Moreira dos Santos; **Event:** samplingProtocol: Malaise intercept trap; verbatimEventDate: 19.iv.12; **Record Level:** institutionCode: DZRJ; basisOfRecord: PreservedSpecimen
- **Type status:** Other material. **Occurrence:** recordedBy: Rafael, J.A. | Limeira-de-Oliveira, F. | Takiya, D.M. | et al.; individualCount: 6; sex: female; lifeStage: adult; **Location:** country: Brazil; stateProvince: Piauí; municipality: Piracuruca; locality: Parque Nacional de Sete Cidades, Riacho da Bananeira; maximumElevationInMeters: 189; verbatimCoordinates: 4°5'59"S, 41°40'48"W; **Identification:** identifiedBy: Allan Paulo Moreira dos Santos; **Event:** samplingProtocol: Malaise intercept trap; verbatimEventDate: 19.iv.12; **Record Level:** institutionCode: DZRJ; basisOfRecord: PreservedSpecimen
- **Type status:** Other material. **Occurrence:** recordedBy: Takiya, D.M.; individualCount: 1; sex: female; lifeStage: adult; **Location:** country: Brazil; stateProvince: Piauí; municipality: Piracuruca; locality: Parque Nacional de Sete Cidades, Riacho da Piedade; maximumElevationInMeters: 169; verbatimCoordinates: 4°6'34"S, 41°43'39"W; **Identification:** identifiedBy: Allan Paulo Moreira dos Santos; **Event:** samplingProtocol: Pennsylvania light trap; verbatimEventDate: 19.iv.12; **Record Level:** institutionCode: INPA; basisOfRecord: PreservedSpecimen
- **Type status:** Other material. **Occurrence:** recordedBy: Rafael, J.A. | Limeira-de-Oliveira, F. | Takiya, D.M. | et al.; individualCount: 50; sex: male; lifeStage: adult; **Location:** country: Brazil; stateProvince: Piauí; municipality: Piracuruca; locality: Parque Nacional de Sete Cidades, Riacho da Piedade; maximumElevationInMeters: 169; verbatimCoordinates: 4°6'34"S, 41°43'39"W; **Identification:** identifiedBy: Allan Paulo Moreira dos Santos; **Event:** samplingProtocol: Malaise intercept trap; verbatimEventDate: 21.iv.12; **Record Level:** institutionCode: DZRJ; basisOfRecord: PreservedSpecimen
- **Type status:** Other material. **Occurrence:** recordedBy: Rafael, J.A. | Limeira-de-Oliveira, F. | Takiya, D.M. | et al.; individualCount: 33; sex: female; lifeStage: adult; **Location:** country: Brazil; stateProvince: Piauí; municipality: Piracuruca; locality: Parque Nacional de Sete Cidades, Riacho da Piedade; maximumElevationInMeters: 169; verbatimCoordinates: 4°6'34"S, 41°43'39"W; **Identification:** identifiedBy: Allan Paulo Moreira dos Santos; **Event:** samplingProtocol: Malaise intercept trap; verbatimEventDate: 21.iv.12; **Record Level:** institutionCode: DZRJ; basisOfRecord: PreservedSpecimen
- **Type status:** Other material. **Occurrence:** recordedBy: Rafael, J.A. | Limeira-de-Oliveira, F. | Takiya, D.M. | et al.; individualCount: 1; sex: male; lifeStage: adult; **Location:** country: Brazil; stateProvince: Piauí; municipality: Piracuruca; locality: Parque Nacional de Sete Cidades, Riacho da Piedade; maximumElevationInMeters: 169; verbatimCoordinates: 4°6'34"S, 41°43'39"W; **Identification:** identifiedBy: Allan Paulo Moreira dos Santos; **Event:** samplingProtocol: Malaise intercept trap; verbatimEventDate: 21.iv.12; **Record Level:** institutionCode: DZRJ; basisOfRecord: PreservedSpecimen

##### Distribution

Honduras. Costa Rica. Panama. Colombia. Suriname. Brazil: PA, AM, PI!, CE!, MT, AC, RO, SP. Ecuador. Peru.

##### Notes

New species record for PI.

#### Smicridea

McLachlan, 1871

##### Notes

New genus record for PI.

#### Smicridea (Rhyacophylax) sp. 1

##### Materials

- **Type status:** Other material. **Occurrence:** recordedBy: Takiya, D.M.; individualCount: 3; sex: male; lifeStage: adult; **Location:** country: Brazil; stateProvince: Piauí; municipality: Piracuruca; locality: Parque Nacional de Sete Cidades, Riacho da Piedade; maximumElevationInMeters: 169; verbatimCoordinates: 4°6'34"S, 41°43'39"W; **Identification:** identifiedBy: Allan Paulo Moreira dos Santos; **Event:** samplingProtocol: Pennsylvania light trap; verbatimEventDate: 19.iv.12; **Record Level:** institutionCode: DZRJ; basisOfRecord: PreservedSpecimen
- **Type status:** Other material. **Occurrence:** recordedBy: Takiya, D.M.; individualCount: 3; sex: female; lifeStage: adult; **Location:** country: Brazil; stateProvince: Piauí; municipality: Piracuruca; locality: Parque Nacional de Sete Cidades, Riacho da Piedade; maximumElevationInMeters: 169; verbatimCoordinates: 4°6'34"S, 41°43'39"W; **Identification:** identifiedBy: Allan Paulo Moreira dos Santos; **Event:** samplingProtocol: Pennsylvania light trap; verbatimEventDate: 19.iv.12; **Record Level:** institutionCode: DZRJ; basisOfRecord: PreservedSpecimen
- **Type status:** Other material. **Occurrence:** recordedBy: Rafael, J.A. | Limeira-de-Oliveira, F. | Takiya, D.M. | et al.; individualCount: 1; sex: male; lifeStage: adult; **Location:** country: Brazil; stateProvince: Piauí; municipality: Piracuruca; locality: Parque Nacional de Sete Cidades, Riacho da Piedade; maximumElevationInMeters: 169; verbatimCoordinates: 4°6'34"S, 41°43'39"W; **Identification:** identifiedBy: Allan Paulo Moreira dos Santos; **Event:** samplingProtocol: Malaise intercept trap; verbatimEventDate: 21.iv.12; **Record Level:** institutionCode: DZRJ; basisOfRecord: PreservedSpecimen
- **Type status:** Other material. **Occurrence:** recordedBy: Rafael, J.A. | Limeira-de-Oliveira, F. | Takiya, D.M. | et al.; individualCount: 1; sex: female; lifeStage: adult; **Location:** country: Brazil; stateProvince: Piauí; municipality: Piracuruca; locality: Parque Nacional de Sete Cidades, Riacho da Piedade; maximumElevationInMeters: 169; verbatimCoordinates: 4°6'34"S, 41°43'39"W; **Identification:** identifiedBy: Allan Paulo Moreira dos Santos; **Event:** samplingProtocol: Malaise intercept trap; verbatimEventDate: 21.iv.12; **Record Level:** institutionCode: DZRJ; basisOfRecord: PreservedSpecimen

#### Smicridea (Smicridea) bivittata

(Hagen, 1861)

##### Materials

- **Type status:** Other material. **Occurrence:** recordedBy: Takiya, D.M.; individualCount: 1; sex: male; lifeStage: adult; **Location:** country: Brazil; stateProvince: Piauí; municipality: Piracuruca; locality: Parque Nacional de Sete Cidades, Cachoeira do Riachão; maximumElevationInMeters: 171; verbatimCoordinates: 4°6'28"S, 41°40'13"W; **Identification:** identifiedBy: Allan Paulo Moreira dos Santos; **Event:** samplingProtocol: Pennsylvania light trap; verbatimEventDate: 19.iv.12; **Record Level:** institutionCode: DZRJ; basisOfRecord: PreservedSpecimen

##### Distribution

Mexico. Guatemala. Honduras. El Salvador. Costa Rica. Trinidad and Tobago. Venezuela. Suriname. Brazil: PA, PI!, CE!, MG, SP. Ecuador.

##### Notes

New species record for Northeastern Brazil.

#### Smicridea (Smicridea) sp. 1

##### Materials

- **Type status:** Other material. **Occurrence:** recordedBy: Takiya, D.M.; individualCount: 2; sex: male; lifeStage: adult; **Location:** country: Brazil; stateProvince: Piauí; municipality: Piracuruca; locality: Parque Nacional de Sete Cidades, Cachoeira do Riachão; maximumElevationInMeters: 171; verbatimCoordinates: 4°6'28"S, 41°40'13"W; **Identification:** identifiedBy: Allan Paulo Moreira dos Santos; **Event:** samplingProtocol: Pennsylvania light trap; verbatimEventDate: 18.iv.12; **Record Level:** institutionCode: DZRJ; basisOfRecord: PreservedSpecimen
- **Type status:** Other material. **Occurrence:** recordedBy: Rafael, J.A. | Limeira-de-Oliveira, F. | Takiya, D.M. | et al.; individualCount: 1; sex: male; lifeStage: adult; **Location:** country: Brazil; stateProvince: Piauí; municipality: Piracuruca; locality: Parque Nacional de Sete Cidades, Cachoeira do Riachão; maximumElevationInMeters: 171; verbatimCoordinates: 4°6'28"S, 41°40'13"W; **Identification:** identifiedBy: Allan Paulo Moreira dos Santos; **Event:** samplingProtocol: Malaise intercept trap; verbatimEventDate: 18.iv.12; **Record Level:** institutionCode: DZRJ; basisOfRecord: PreservedSpecimen
- **Type status:** Other material. **Occurrence:** recordedBy: Rafael, J.A. | Limeira-de-Oliveira, F. | Takiya, D.M. | et al.; individualCount: 17; sex: male; lifeStage: adult; **Location:** country: Brazil; stateProvince: Piauí; municipality: Piracuruca; locality: Parque Nacional de Sete Cidades, Riacho da Piedade; maximumElevationInMeters: 169; verbatimCoordinates: 4°6'34"S, 41°43'39"W; **Identification:** identifiedBy: Allan Paulo Moreira dos Santos; **Event:** samplingProtocol: Malaise intercept trap; verbatimEventDate: 18.iv.12; **Record Level:** institutionCode: DZRJ; basisOfRecord: PreservedSpecimen
- **Type status:** Other material. **Occurrence:** recordedBy: Rafael, J.A. | Limeira-de-Oliveira, F. | Takiya, D.M. | et al.; individualCount: 17; sex: male; lifeStage: adult; **Location:** country: Brazil; stateProvince: Piauí; municipality: Piracuruca; locality: Parque Nacional de Sete Cidades, Riacho da Piedade; maximumElevationInMeters: 169; verbatimCoordinates: 4°6'34"S, 41°43'39"W; **Identification:** identifiedBy: Allan Paulo Moreira dos Santos; **Event:** samplingProtocol: Malaise intercept trap; verbatimEventDate: 18.iv.12; **Record Level:** institutionCode: DZRJ; basisOfRecord: PreservedSpecimen
- **Type status:** Other material. **Occurrence:** recordedBy: Takiya, D.M. | Rafael, J.A.; individualCount: 1; sex: male; lifeStage: adult; **Location:** country: Brazil; stateProvince: Piauí; municipality: Piracuruca; locality: Parque Nacional de Sete Cidades, Cachoeira do Riachão; maximumElevationInMeters: 171; verbatimCoordinates: 4°6'28"S, 41°40'13"W; **Identification:** identifiedBy: Allan Paulo Moreira dos Santos; **Event:** samplingProtocol: Pennsylvania light trap; verbatimEventDate: 20.iv.12; **Record Level:** institutionCode: DZRJ; basisOfRecord: PreservedSpecimen
- **Type status:** Other material. **Occurrence:** recordedBy: Takiya, D.M.; individualCount: 1; sex: male; lifeStage: adult; **Location:** country: Brazil; stateProvince: Piauí; municipality: Piracuruca; locality: Parque Nacional de Sete Cidades, Alojamento; maximumElevationInMeters: 193; verbatimCoordinates: 4°5'57"S, 41°42'34"W; **Identification:** identifiedBy: Allan Paulo Moreira dos Santos; **Event:** samplingProtocol: White sheet light trap; verbatimEventDate: 20.iv.12; **Record Level:** institutionCode: DZRJ; basisOfRecord: PreservedSpecimen
- **Type status:** Other material. **Occurrence:** recordedBy: Rafael, J.A. | Limeira-de-Oliveira, F. | Takiya, D.M. | et al.; individualCount: 26; sex: male; lifeStage: adult; **Location:** country: Brazil; stateProvince: Piauí; municipality: Piracuruca; locality: Parque Nacional de Sete Cidades, Riacho da Piedade; maximumElevationInMeters: 169; verbatimCoordinates: 4°6'34"S, 41°43'39"W; **Identification:** identifiedBy: Allan Paulo Moreira dos Santos; **Event:** samplingProtocol: Malaise intercept trap; verbatimEventDate: 21.iv.12; **Record Level:** institutionCode: DZRJ; basisOfRecord: PreservedSpecimen
- **Type status:** Other material. **Occurrence:** recordedBy: Rafael, J.A. | Limeira-de-Oliveira, F. | Takiya, D.M. | et al.; individualCount: 27; sex: male; lifeStage: adult; **Location:** country: Brazil; stateProvince: Piauí; municipality: Piracuruca; locality: Parque Nacional de Sete Cidades, Riacho da Piedade; maximumElevationInMeters: 169; verbatimCoordinates: 4°6'34"S, 41°43'39"W; **Identification:** identifiedBy: Allan Paulo Moreira dos Santos; **Event:** samplingProtocol: Malaise intercept trap; verbatimEventDate: 21.iv.12; **Record Level:** institutionCode: DZRJ; basisOfRecord: PreservedSpecimen
- **Type status:** Other material. **Occurrence:** recordedBy: Rafael, J.A. | Limeira-de-Oliveira, F. | Takiya, D.M. | et al.; individualCount: 28; sex: female; lifeStage: adult; **Location:** country: Brazil; stateProvince: Piauí; municipality: Piracuruca; locality: Parque Nacional de Sete Cidades, Riacho da Piedade; maximumElevationInMeters: 169; verbatimCoordinates: 4°6'34"S, 41°43'39"W; **Identification:** identifiedBy: Allan Paulo Moreira dos Santos; **Event:** samplingProtocol: Malaise intercept trap; verbatimEventDate: 21.iv.12; **Record Level:** institutionCode: DZRJ; basisOfRecord: PreservedSpecimen

##### Notes

Undescribed species.

#### Smicridea (Smicridea) sp. 2*

##### Materials

- **Type status:** Other material. **Occurrence:** recordedBy: Takiya, D.M.; individualCount: 2; sex: male; lifeStage: adult; **Location:** country: Brazil; stateProvince: Piauí; municipality: Piracuruca; locality: Parque Nacional de Sete Cidades, Cachoeira do Riachão; maximumElevationInMeters: 171; verbatimCoordinates: 4°6'28"S, 41°40'13"W; **Identification:** identifiedBy: Allan Paulo Moreira dos Santos; **Event:** samplingProtocol: Pennsylvania light trap; verbatimEventDate: 18.iv.12; **Record Level:** institutionCode: DZRJ; basisOfRecord: PreservedSpecimen
- **Type status:** Other material. **Occurrence:** recordedBy: Rafael, J.A. | Limeira-de-Oliveira, F. | Takiya, D.M. | et al.; individualCount: 3; sex: male; lifeStage: adult; **Location:** country: Brazil; stateProvince: Piauí; municipality: Piracuruca; locality: Parque Nacional de Sete Cidades, Cachoeira do Riachão; maximumElevationInMeters: 171; verbatimCoordinates: 4°6'28"S, 41°40'13"W; **Identification:** identifiedBy: Allan Paulo Moreira dos Santos; **Event:** samplingProtocol: Malaise intercept trap; verbatimEventDate: 18.iv.12; **Record Level:** institutionCode: DZRJ; basisOfRecord: PreservedSpecimen
- **Type status:** Other material. **Occurrence:** recordedBy: Rafael, J.A. | Limeira-de-Oliveira, F. | Takiya, D.M. | et al.; individualCount: 2; sex: male; lifeStage: adult; **Location:** country: Brazil; stateProvince: Piauí; municipality: Piracuruca; locality: Parque Nacional de Sete Cidades, Riacho da Piedade; maximumElevationInMeters: 169; verbatimCoordinates: 4°6'34"S, 41°43'39"W; **Identification:** identifiedBy: Allan Paulo Moreira dos Santos; **Event:** samplingProtocol: Malaise intercept trap; verbatimEventDate: 18.iv.12; **Record Level:** institutionCode: DZRJ; basisOfRecord: PreservedSpecimen
- **Type status:** Other material. **Occurrence:** recordedBy: Rafael, J.A. | Limeira-de-Oliveira, F. | Takiya, D.M. | et al.; individualCount: 3; sex: male; lifeStage: adult; **Location:** country: Brazil; stateProvince: Piauí; municipality: Piracuruca; locality: Parque Nacional de Sete Cidades, Riacho da Piedade; maximumElevationInMeters: 169; verbatimCoordinates: 4°6'34"S, 41°43'39"W; **Identification:** identifiedBy: Allan Paulo Moreira dos Santos; **Event:** samplingProtocol: Malaise intercept trap; verbatimEventDate: 18.iv.12; **Record Level:** institutionCode: DZRJ; basisOfRecord: PreservedSpecimen
- **Type status:** Other material. **Occurrence:** recordedBy: Rafael, J.A. | Limeira-de-Oliveira, F. | Takiya, D.M. | et al.; individualCount: 2; sex: male; lifeStage: adult; **Location:** country: Brazil; stateProvince: Piauí; municipality: Piracuruca; locality: Parque Nacional de Sete Cidades, Riacho da Bananeira; maximumElevationInMeters: 189; verbatimCoordinates: 4°5'59"S, 41°40'48"W; **Identification:** identifiedBy: Allan Paulo Moreira dos Santos; **Event:** samplingProtocol: Malaise intercept trap; verbatimEventDate: 19.iv.12; **Record Level:** institutionCode: DZRJ; basisOfRecord: PreservedSpecimen
- **Type status:** Other material. **Occurrence:** recordedBy: Rafael, J.A. | Limeira-de-Oliveira, F. | Takiya, D.M. | et al.; individualCount: 2; sex: male; lifeStage: adult; **Location:** country: Brazil; stateProvince: Piauí; municipality: Piracuruca; locality: Parque Nacional de Sete Cidades, Riacho da Piedade; maximumElevationInMeters: 169; verbatimCoordinates: 4°6'34"S, 41°43'39"W; **Identification:** identifiedBy: Allan Paulo Moreira dos Santos; **Event:** samplingProtocol: Malaise intercept trap; verbatimEventDate: 21.iv.12; **Record Level:** institutionCode: DZRJ; basisOfRecord: PreservedSpecimen

##### Notes

Undescribed species.

#### Hydroptilidae

##### Notes

Family firstly recorded from PI in Souza et al. 2014a​.

#### Betrichia

Mosely, 1939

##### Notes

Genus firstly recorded from PI in Souza et al. 2016b.

#### Betrichia nhundiaquara

Souza, Santos & Takiya, 2016

##### Materials

- **Type status:** Other material. **Occurrence:** recordedBy: Rafael, J.A. | Limeira-de-Oliveira, F. | Takiya, D.M. | et al.; individualCount: 1; sex: male; lifeStage: adult; **Location:** country: Brazil; stateProvince: Piauí; municipality: Piracuruca; locality: Parque Nacional de Sete Cidades, Riacho da Piedade; maximumElevationInMeters: 169; verbatimCoordinates: 4°6'34"S, 41°43'39"W; **Identification:** identifiedBy: Allan Paulo Moreira dos Santos; **Event:** samplingProtocol: Malaise intercept trap; verbatimEventDate: 21.iv.12; **Record Level:** institutionCode: DZRJ; basisOfRecord: PreservedSpecimen

##### Distribution

Brazil: PI, BA, MG, PR.

##### Notes

Species described in Souza et al. 2016b.

#### Flintiella

Angrisano, 1995

##### Notes

Genus firstly recorded from PI in Souza et al. 2016a.

#### Flintiella harrisi

Souza, Santos & Takiya, 2016

##### Materials

- **Type status:** Other material. **Occurrence:** recordedBy: Rafael, J.A. | Limeira-de-Oliveira, F. | Takiya, D.M. | et al.; individualCount: 1; sex: male; lifeStage: adult; **Location:** country: Brazil; stateProvince: Piauí; municipality: Piracuruca; locality: Parque Nacional de Sete Cidades, Riacho da Piedade; maximumElevationInMeters: 169; verbatimCoordinates: 4°6'34"S, 41°43'39"W; **Identification:** identifiedBy: Allan Paulo Moreira dos Santos; **Event:** samplingProtocol: Malaise intercept trap; verbatimEventDate: 21.iv.12; **Record Level:** institutionCode: DZRJ; basisOfRecord: PreservedSpecimen

##### Distribution

Brazil: PI.

##### Notes

Species described in Souza et al. 2016a.

#### Hydroptila

Dalman, 1918

##### Notes

Genus firstly recorded from PI in Souza et al. 2014a.

#### Hydroptila florestani

Souza, Santos & Takiya, 2014

##### Materials

- **Type status:** Other material. **Occurrence:** recordedBy: Takiya, D.M.; individualCount: 2; sex: male; lifeStage: adult; **Location:** country: Brazil; stateProvince: Piauí; municipality: Piracuruca; locality: Parque Nacional de Sete Cidades, Riacho da Piedade; maximumElevationInMeters: 169; verbatimCoordinates: 4°6'34"S, 41°43'39"W; **Identification:** identifiedBy: Allan Paulo Moreira dos Santos; **Event:** samplingProtocol: Pennsylvania light trap; verbatimEventDate: 19.iv.12; **Record Level:** institutionCode: DZRJ; basisOfRecord: PreservedSpecimen
- **Type status:** Other material. **Occurrence:** recordedBy: Takiya, D.M.; individualCount: 2; sex: female; lifeStage: adult; **Location:** country: Brazil; stateProvince: Piauí; municipality: Piracuruca; locality: Parque Nacional de Sete Cidades, Riacho da Piedade; maximumElevationInMeters: 169; verbatimCoordinates: 4°6'34"S, 41°43'39"W; **Identification:** identifiedBy: Allan Paulo Moreira dos Santos; **Event:** samplingProtocol: Pennsylvania light trap; verbatimEventDate: 19.iv.12; **Record Level:** institutionCode: DZRJ; basisOfRecord: PreservedSpecimen
- **Type status:** Other material. **Occurrence:** recordedBy: Rafael, J.A. | Limeira-de-Oliveira, F. | Takiya, D.M. | et al.; individualCount: 3; sex: male; lifeStage: adult; **Location:** country: Brazil; stateProvince: Piauí; municipality: Piracuruca; locality: Parque Nacional de Sete Cidades, Riacho da Piedade; maximumElevationInMeters: 169; verbatimCoordinates: 4°6'34"S, 41°43'39"W; **Identification:** identifiedBy: Allan Paulo Moreira dos Santos; **Event:** samplingProtocol: Malaise intercept trap; verbatimEventDate: 21.iv.12; **Record Level:** institutionCode: DZRJ; basisOfRecord: PreservedSpecimen
- **Type status:** Other material. **Occurrence:** recordedBy: Rafael, J.A. | Limeira-de-Oliveira, F. | Takiya, D.M. | et al.; individualCount: 1; sex: female; lifeStage: adult; **Location:** country: Brazil; stateProvince: Piauí; municipality: Piracuruca; locality: Parque Nacional de Sete Cidades, Riacho da Piedade; maximumElevationInMeters: 169; verbatimCoordinates: 4°6'34"S, 41°43'39"W; **Identification:** identifiedBy: Allan Paulo Moreira dos Santos; **Event:** samplingProtocol: Malaise intercept trap; verbatimEventDate: 21.iv.12; **Record Level:** institutionCode: DZRJ; basisOfRecord: PreservedSpecimen

##### Distribution

Brazil: PI.

##### Notes

Species described in Souza et al. 2014a.

#### Neotrichia

Morton, 1905

##### Notes

New genus record for PI.

#### Neotrichia sp. 1

##### Materials

- **Type status:** Other material. **Occurrence:** recordedBy: Takiya, D.M.; individualCount: 1; sex: male; lifeStage: adult; **Location:** country: Brazil; stateProvince: Piauí; municipality: Piracuruca; locality: Parque Nacional de Sete Cidades, Cachoeira do Riachão; maximumElevationInMeters: 171; verbatimCoordinates: 4°6'28"S, 41°40'13"W; **Identification:** identifiedBy: Allan Paulo Moreira dos Santos; **Event:** samplingProtocol: Pennsylvania light trap; verbatimEventDate: 19.iv.12; **Record Level:** institutionCode: DZRJ; basisOfRecord: PreservedSpecimen
- **Type status:** Other material. **Occurrence:** recordedBy: Takiya, D.M.; individualCount: 16; sex: male; lifeStage: adult; **Location:** country: Brazil; stateProvince: Piauí; municipality: Piracuruca; locality: Parque Nacional de Sete Cidades, Riacho da Piedade; maximumElevationInMeters: 169; verbatimCoordinates: 4°6'34"S, 41°43'39"W; **Identification:** identifiedBy: Allan Paulo Moreira dos Santos; **Event:** samplingProtocol: Pennsylvania light trap; verbatimEventDate: 19.iv.12; **Record Level:** institutionCode: DZRJ; basisOfRecord: PreservedSpecimen
- **Type status:** Other material. **Occurrence:** recordedBy: Takiya, D.M.; individualCount: 1; sex: male; lifeStage: adult; **Location:** country: Brazil; stateProvince: Piauí; municipality: Piracuruca; locality: Parque Nacional de Sete Cidades, Alojamento; maximumElevationInMeters: 193; verbatimCoordinates: 4°5'57"S, 41°42'34"W; **Identification:** identifiedBy: Allan Paulo Moreira dos Santos; **Event:** samplingProtocol: White sheet light trap; verbatimEventDate: 20.iv.12; **Record Level:** institutionCode: DZRJ; basisOfRecord: PreservedSpecimen
- **Type status:** Other material. **Occurrence:** recordedBy: Takiya, D.M.; individualCount: 1; sex: female; lifeStage: adult; **Location:** country: Brazil; stateProvince: Piauí; municipality: Piracuruca; locality: Parque Nacional de Sete Cidades, Alojamento; maximumElevationInMeters: 193; verbatimCoordinates: 4°5'57"S, 41°42'34"W; **Identification:** identifiedBy: Allan Paulo Moreira dos Santos; **Event:** samplingProtocol: White sheet light trap; verbatimEventDate: 20.iv.12; **Record Level:** institutionCode: DZRJ; basisOfRecord: PreservedSpecimen

#### Neotrichia sp. 2*

##### Materials

- **Type status:** Other material. **Occurrence:** recordedBy: Takiya, D.M.; individualCount: 1; sex: male; lifeStage: adult; **Location:** country: Brazil; stateProvince: Piauí; municipality: Piracuruca; locality: Parque Nacional de Sete Cidades, Cachoeira do Riachão; maximumElevationInMeters: 171; verbatimCoordinates: 4°6'28"S, 41°40'13"W; **Identification:** identifiedBy: Allan Paulo Moreira dos Santos; **Event:** samplingProtocol: Pennsylvania light trap; verbatimEventDate: 19.iv.12; **Record Level:** institutionCode: DZRJ; basisOfRecord: PreservedSpecimen

#### Neotrichia sp. 3

##### Materials

- **Type status:** Other material. **Occurrence:** recordedBy: Takiya, D.M.; individualCount: 1; sex: male; lifeStage: adult; **Location:** country: Brazil; stateProvince: Piauí; municipality: Piracuruca; locality: Parque Nacional de Sete Cidades, Cachoeira do Riachão; maximumElevationInMeters: 171; verbatimCoordinates: 4°6'28"S, 41°40'13"W; **Identification:** identifiedBy: Allan Paulo Moreira dos Santos; **Event:** samplingProtocol: Pennsylvania light trap; verbatimEventDate: 19.iv.12; **Record Level:** institutionCode: DZRJ; basisOfRecord: PreservedSpecimen

#### Neotrichia sp. 4

##### Materials

- **Type status:** Other material. **Occurrence:** recordedBy: Takiya, D.M.; individualCount: 1; sex: male; lifeStage: adult; **Location:** country: Brazil; stateProvince: Piauí; municipality: Piracuruca; locality: Parque Nacional de Sete Cidades, Cachoeira do Riachão; maximumElevationInMeters: 171; verbatimCoordinates: 4°6'28"S, 41°40'13"W; **Identification:** identifiedBy: Allan Paulo Moreira dos Santos; **Event:** samplingProtocol: Pennsylvania light trap; verbatimEventDate: 19.iv.12; **Record Level:** institutionCode: DZRJ; basisOfRecord: PreservedSpecimen
- **Type status:** Other material. **Occurrence:** recordedBy: Takiya, D.M.; individualCount: 4; sex: male; lifeStage: adult; **Location:** country: Brazil; stateProvince: Piauí; municipality: Piracuruca; locality: Parque Nacional de Sete Cidades, Riacho da Piedade; maximumElevationInMeters: 169; verbatimCoordinates: 4°6'34"S, 41°43'39"W; **Identification:** identifiedBy: Allan Paulo Moreira dos Santos; **Event:** samplingProtocol: Pennsylvania light trap; verbatimEventDate: 19.iv.12; **Record Level:** institutionCode: DZRJ; basisOfRecord: PreservedSpecimen

#### Ochrotrichia

Mosely, 1934

##### Notes

New genus record for PI.

#### Ochrotrichia sp. 1

##### Materials

- **Type status:** Other material. **Occurrence:** recordedBy: Takiya, D.M.; individualCount: 1; sex: male; lifeStage: adult; **Location:** country: Brazil; stateProvince: Piauí; municipality: Piracuruca; locality: Parque Nacional de Sete Cidades, Cachoeira do Riachão; maximumElevationInMeters: 171; verbatimCoordinates: 4°6'28"S, 41°40'13"W; **Identification:** identifiedBy: Allan Paulo Moreira dos Santos; **Event:** samplingProtocol: Pennsylvania light trap; verbatimEventDate: 19.iv.12; **Record Level:** institutionCode: DZRJ; basisOfRecord: PreservedSpecimen

#### Oxyethira

Eaton, 1873

##### Notes

New genus record for PI.

#### Oxyethira longissima

Flint, 1974

##### Materials

- **Type status:** Other material. **Occurrence:** recordedBy: Takiya, D.M.; individualCount: 1; sex: male; lifeStage: adult; **Location:** country: Brazil; stateProvince: Piauí; municipality: Piracuruca; locality: Parque Nacional de Sete Cidades, Cachoeira do Riachão; maximumElevationInMeters: 171; verbatimCoordinates: 4°6'28"S, 41°40'13"W; **Identification:** identifiedBy: Allan Paulo Moreira dos Santos; **Event:** samplingProtocol: Pennsylvania light trap; verbatimEventDate: 18.iv.12; **Record Level:** institutionCode: DZRJ; basisOfRecord: PreservedSpecimen
- **Type status:** Other material. **Occurrence:** recordedBy: Takiya, D.M.; individualCount: 1; sex: male; lifeStage: adult; **Location:** country: Brazil; stateProvince: Piauí; municipality: Piracuruca; locality: Parque Nacional de Sete Cidades, Cachoeira do Riachão; maximumElevationInMeters: 171; verbatimCoordinates: 4°6'28"S, 41°40'13"W; **Identification:** identifiedBy: Allan Paulo Moreira dos Santos; **Event:** samplingProtocol: Pennsylvania light trap; verbatimEventDate: 19.iv.12; **Record Level:** institutionCode: DZRJ; basisOfRecord: PreservedSpecimen

##### Distribution

Suriname. Brazil: AM, PI!.

##### Notes

New species record for Northeastern Brazil.

#### Oxyethira merga

Kelley, 1983

##### Materials

- **Type status:** Other material. **Occurrence:** recordedBy: Takiya, D.M.; individualCount: 1; sex: male; lifeStage: adult; **Location:** country: Brazil; stateProvince: Piauí; municipality: Piracuruca; locality: Parque Nacional de Sete Cidades, Cachoeira do Riachão; maximumElevationInMeters: 171; verbatimCoordinates: 4°6'28"S, 41°40'13"W; **Identification:** identifiedBy: Allan Paulo Moreira dos Santos; **Event:** samplingProtocol: Pennsylvania light trap; verbatimEventDate: 18.iv.12; **Record Level:** institutionCode: DZRJ; basisOfRecord: PreservedSpecimen

##### Distribution

Venezuela. Brazil: RR, PI!.

##### Notes

New species record for Northeastern Brazil.

#### Oxyethira spissa

Kelley, 1983

##### Materials

- **Type status:** Other material. **Occurrence:** recordedBy: Takiya, D.M.; individualCount: 1; sex: male; lifeStage: adult; **Location:** country: Brazil; stateProvince: Piauí; municipality: Piracuruca; locality: Parque Nacional de Sete Cidades, Cachoeira do Riachão; maximumElevationInMeters: 171; verbatimCoordinates: 4°6'28"S, 41°40'13"W; **Identification:** identifiedBy: Wagner Rafael Maciel de Souza; **Event:** samplingProtocol: Pennsylvania light trap; verbatimEventDate: 18.iv.12; **Record Level:** institutionCode: DZRJ; basisOfRecord: PreservedSpecimen

##### Distribution

Brazil: PA, PI!.

##### Notes

New species record for Northeastern Brazil.

#### Oxyethira tica

Harris & Holzenthal, 1992

##### Materials

- **Type status:** Other material. **Occurrence:** recordedBy: Takiya, D.M.; individualCount: 1; sex: male; lifeStage: adult; **Location:** country: Brazil; stateProvince: Piauí; municipality: Piracuruca; locality: Parque Nacional de Sete Cidades, Cachoeira do Riachão; maximumElevationInMeters: 171; verbatimCoordinates: 4°6'28"S, 41°40'13"W; **Identification:** identifiedBy: Allan Paulo Moreira dos Santos; **Event:** samplingProtocol: Pennsylvania light trap; verbatimEventDate: 18.iv.12; **Record Level:** institutionCode: DZRJ; basisOfRecord: PreservedSpecimen
- **Type status:** Other material. **Occurrence:** recordedBy: Takiya, D.M.; individualCount: 1; sex: male; lifeStage: adult; **Location:** country: Brazil; stateProvince: Piauí; municipality: Piracuruca; locality: Parque Nacional de Sete Cidades, Cachoeira do Riachão; maximumElevationInMeters: 171; verbatimCoordinates: 4°6'28"S, 41°40'13"W; **Identification:** identifiedBy: Allan Paulo Moreira dos Santos; **Event:** samplingProtocol: Pennsylvania light trap; verbatimEventDate: 19.iv.12; **Record Level:** institutionCode: DZRJ; basisOfRecord: PreservedSpecimen
- **Type status:** Other material. **Occurrence:** recordedBy: Takiya, D.M.; individualCount: 2; sex: male; lifeStage: adult; **Location:** country: Brazil; stateProvince: Piauí; municipality: Piracuruca; locality: Parque Nacional de Sete Cidades, Cachoeira do Riachão; maximumElevationInMeters: 171; verbatimCoordinates: 4°6'28"S, 41°40'13"W; **Identification:** identifiedBy: Allan Paulo Moreira dos Santos; **Event:** samplingProtocol: Pennsylvania light trap; verbatimEventDate: 19.iv.12; **Record Level:** institutionCode: DZRJ; basisOfRecord: PreservedSpecimen
- **Type status:** Other material. **Occurrence:** recordedBy: Takiya, D.M. | Rafael, J.A.; individualCount: 1; sex: male; lifeStage: adult; **Location:** country: Brazil; stateProvince: Piauí; municipality: Piracuruca; locality: Parque Nacional de Sete Cidades, Cachoeira do Riachão; maximumElevationInMeters: 171; verbatimCoordinates: 4°6'28"S, 41°40'13"W; **Identification:** identifiedBy: Allan Paulo Moreira dos Santos; **Event:** samplingProtocol: Pennsylvania light trap; verbatimEventDate: 20.iv.12; **Record Level:** institutionCode: DZRJ; basisOfRecord: PreservedSpecimen

##### Distribution

Mexico. Honduras. Costa Rica. Panama. Guadeloupe. Dominica. Santa Lucia. Saint Vicent and the Grenadines. Granada. Trinidad and Tobago. Venezuela. Brazil: AM, PI!, CE!, MG, RJ. Ecuador.

##### Notes

New species record for Northeastern Brazil.

#### Oxyethira sp. 1

##### Materials

- **Type status:** Other material. **Occurrence:** recordedBy: Takiya, D.M.; individualCount: 1; sex: male; lifeStage: adult; **Location:** country: Brazil; stateProvince: Piauí; municipality: Piracuruca; locality: Parque Nacional de Sete Cidades, Cachoeira do Riachão; maximumElevationInMeters: 171; verbatimCoordinates: 4°6'28"S, 41°40'13"W; **Identification:** identifiedBy: Allan Paulo Moreira dos Santos; **Event:** samplingProtocol: Pennsylvania light trap; verbatimEventDate: 18.iv.12; **Record Level:** institutionCode: DZRJ; basisOfRecord: PreservedSpecimen
- **Type status:** Other material. **Occurrence:** recordedBy: Takiya, D.M.; individualCount: 13; sex: male; lifeStage: adult; **Location:** country: Brazil; stateProvince: Piauí; municipality: Piracuruca; locality: Parque Nacional de Sete Cidades, Riacho da Piedade; maximumElevationInMeters: 169; verbatimCoordinates: 4°6'34"S, 41°43'39"W; **Identification:** identifiedBy: Allan Paulo Moreira dos Santos; **Event:** samplingProtocol: Pennsylvania light trap; verbatimEventDate: 19.iv.12; **Record Level:** institutionCode: DZRJ; basisOfRecord: PreservedSpecimen
- **Type status:** Other material. **Occurrence:** recordedBy: Takiya, D.M.; individualCount: 1; sex: male; lifeStage: adult; **Location:** country: Brazil; stateProvince: Piauí; municipality: Piracuruca; locality: Parque Nacional de Sete Cidades, Alojamento; maximumElevationInMeters: 193; verbatimCoordinates: 4°5'57"S, 41°42'34"W; **Identification:** identifiedBy: Allan Paulo Moreira dos Santos; **Event:** samplingProtocol: White sheet light trap; verbatimEventDate: 20.iv.12; **Record Level:** institutionCode: DZRJ; basisOfRecord: PreservedSpecimen
- **Type status:** Other material. **Occurrence:** recordedBy: Takiya, D.M.; individualCount: 1; sex: female; lifeStage: adult; **Location:** country: Brazil; stateProvince: Piauí; municipality: Piracuruca; locality: Parque Nacional de Sete Cidades, Alojamento; maximumElevationInMeters: 193; verbatimCoordinates: 4°5'57"S, 41°42'34"W; **Identification:** identifiedBy: Allan Paulo Moreira dos Santos; **Event:** samplingProtocol: White sheet light trap; verbatimEventDate: 20.iv.12; **Record Level:** institutionCode: DZRJ; basisOfRecord: PreservedSpecimen

##### Notes

Undescribed species.

#### Oxyethira sp. 2

##### Materials

- **Type status:** Other material. **Occurrence:** recordedBy: Santos, A.P.M. | Takiya, D.M.; individualCount: 3; sex: male; lifeStage: adult; **Location:** country: Brazil; stateProvince: Piauí; municipality: Piracuruca; locality: Parque Nacional de Sete Cidades, Poço do Bananeira; maximumElevationInMeters: 158; verbatimCoordinates: 4°5'55.8"S, 41°40'33.8"W; **Identification:** identifiedBy: Allan Paulo Moreira dos Santos; **Event:** samplingProtocol: Pennsylvania light trap; verbatimEventDate: 11.ii.13; **Record Level:** institutionCode: DZRJ; basisOfRecord: PreservedSpecimen
- **Type status:** Other material. **Occurrence:** recordedBy: Santos, A.P.M. | Takiya, D.M.; individualCount: 1; sex: female; lifeStage: adult; **Location:** country: Brazil; stateProvince: Piauí; municipality: Piracuruca; locality: Parque Nacional de Sete Cidades, Poço do Bananeira; maximumElevationInMeters: 158; verbatimCoordinates: 4°5'55.8"S, 41°40'33.8"W; **Identification:** identifiedBy: Allan Paulo Moreira dos Santos; **Event:** samplingProtocol: Pennsylvania light trap; verbatimEventDate: 11.ii.13; **Record Level:** institutionCode: DZRJ; basisOfRecord: PreservedSpecimen
- **Type status:** Other material. **Occurrence:** recordedBy: Rafael, J.A. | Limeira-de-Oliveira, F. | Takiya, D.M. | et al.; individualCount: 1; sex: male; lifeStage: adult; **Location:** country: Brazil; stateProvince: Piauí; municipality: Piracuruca; locality: Parque Nacional de Sete Cidades, Riacho da Bananeira; maximumElevationInMeters: 189; verbatimCoordinates: 4°5'59"S, 41°40'48"W; **Identification:** identifiedBy: Allan Paulo Moreira dos Santos; **Event:** samplingProtocol: Malaise intercept trap; verbatimEventDate: 19.iv.12; **Record Level:** institutionCode: DZRJ; basisOfRecord: PreservedSpecimen
- **Type status:** Other material. **Occurrence:** recordedBy: Takiya, D.M.; individualCount: 4; sex: male; lifeStage: adult; **Location:** country: Brazil; stateProvince: Piauí; municipality: Piracuruca; locality: Parque Nacional de Sete Cidades, Cachoeira do Riachão; maximumElevationInMeters: 171; verbatimCoordinates: 4°6'28"S, 41°40'13"W; **Identification:** identifiedBy: Allan Paulo Moreira dos Santos; **Event:** samplingProtocol: Pennsylvania light trap; verbatimEventDate: 19.iv.12; **Record Level:** institutionCode: DZRJ; basisOfRecord: PreservedSpecimen
- **Type status:** Other material. **Occurrence:** recordedBy: Takiya, D.M.; individualCount: 2; sex: male; lifeStage: adult; **Location:** country: Brazil; stateProvince: Piauí; municipality: Piracuruca; locality: Parque Nacional de Sete Cidades, Riacho da Piedade; maximumElevationInMeters: 169; verbatimCoordinates: 4°6'34"S, 41°43'39"W; **Identification:** identifiedBy: Allan Paulo Moreira dos Santos; **Event:** samplingProtocol: Pennsylvania light trap; verbatimEventDate: 19.iv.12; **Record Level:** institutionCode: DZRJ; basisOfRecord: PreservedSpecimen

##### Notes

Undescribed species.

#### Oxyethira sp. 3

##### Materials

- **Type status:** Other material. **Occurrence:** recordedBy: Takiya, D.M.; individualCount: 1; sex: male; lifeStage: adult; **Location:** country: Brazil; stateProvince: Piauí; municipality: Piracuruca; locality: Parque Nacional de Sete Cidades, Cachoeira do Riachão; maximumElevationInMeters: 171; verbatimCoordinates: 4°6'28"S, 41°40'13"W; **Identification:** identifiedBy: Allan Paulo Moreira dos Santos; **Event:** samplingProtocol: Pennsylvania light trap; verbatimEventDate: 19.iv.12; **Record Level:** institutionCode: DZRJ; basisOfRecord: PreservedSpecimen

##### Notes

Undescribed species.

#### Oxyethira sp. 4

##### Materials

- **Type status:** Other material. **Occurrence:** recordedBy: Santos, A.P.M. | Takiya, D.M.; individualCount: 1; sex: male; lifeStage: adult; **Location:** country: Brazil; stateProvince: Piauí; municipality: Piracuruca; locality: Parque Nacional de Sete Cidades, Poço do Bananeira; maximumElevationInMeters: 158; verbatimCoordinates: 4°5'55.8"S, 41°40'33.8"W; **Identification:** identifiedBy: Allan Paulo Moreira dos Santos; **Event:** samplingProtocol: Pennsylvania light trap; verbatimEventDate: 9.ii.13; **Record Level:** institutionCode: DZRJ; basisOfRecord: PreservedSpecimen

##### Notes

Undescribed species.

#### Oxyethira sp. 5

##### Materials

- **Type status:** Other material. **Occurrence:** recordedBy: Takiya, D.M.; individualCount: 3; sex: male; lifeStage: adult; **Location:** country: Brazil; stateProvince: Piauí; municipality: Piracuruca; locality: Parque Nacional de Sete Cidades, Cachoeira do Riachão; maximumElevationInMeters: 171; verbatimCoordinates: 4°6'28"S, 41°40'13"W; **Identification:** identifiedBy: Wagner Rafael Maciel de Souza; **Event:** samplingProtocol: Pennsylvania light trap; verbatimEventDate: 19.iv.12; **Record Level:** institutionCode: DZRJ; basisOfRecord: PreservedSpecimen
- **Type status:** Other material. **Occurrence:** recordedBy: Takiya, D.M.; individualCount: 1; sex: male; lifeStage: adult; **Location:** country: Brazil; stateProvince: Piauí; municipality: Piracuruca; locality: Parque Nacional de Sete Cidades, Riacho da Piedade; maximumElevationInMeters: 169; verbatimCoordinates: 4°6'34"S, 41°43'39"W; **Identification:** identifiedBy: Allan Paulo Moreira dos Santos; **Event:** samplingProtocol: Pennsylvania light trap; verbatimEventDate: 19.iv.12; **Record Level:** institutionCode: DZRJ; basisOfRecord: PreservedSpecimen

##### Notes

Undescribed species.

#### Leptoceridae

#### Nectopsyche

Müller, 1879

#### Nectopsyche multilineata

Flint, 1983

##### Materials

- **Type status:** Other material. **Occurrence:** recordedBy: Takiya, D.M.; individualCount: 2; sex: female; lifeStage: adult; **Location:** country: Brazil; stateProvince: Piauí; municipality: Piracuruca; locality: Parque Nacional de Sete Cidades, Centro de visitantes; maximumElevationInMeters: 202; verbatimCoordinates: 4°6'20"S, 41°41'52"W; **Identification:** identifiedBy: Ana Lucia Henriques Oliveira; **Event:** samplingProtocol: White sheet light trap; verbatimEventDate: 19.iv.12; **Record Level:** institutionCode: DZRJ; basisOfRecord: PreservedSpecimen

##### Distribution

Venezuela. Brazil: RR, PI!. Argentina.

##### Notes

if confirmed, new species record for Northeastern Brazil.

#### Nectopsyche muhni

Flint, 1983

##### Materials

- **Type status:** Other material. **Occurrence:** recordedBy: Takiya, D.M.; individualCount: 1; sex: male; lifeStage: adult; **Location:** country: Brazil; stateProvince: Piauí; municipality: Piracuruca; locality: Parque Nacional de Sete Cidades, Cachoeira do Riachão; maximumElevationInMeters: 171; verbatimCoordinates: 4°6'28"S, 41°40'13"W; **Identification:** identifiedBy: Ana Lucia Henriques Oliveira; **Event:** samplingProtocol: Pennsylvania light trap; verbatimEventDate: 18.iv.12; **Record Level:** institutionCode: DZRJ; basisOfRecord: PreservedSpecimen
- **Type status:** Other material. **Occurrence:** recordedBy: Takiya, D.M.; individualCount: 7; sex: male; lifeStage: adult; **Location:** country: Brazil; stateProvince: Piauí; municipality: Piracuruca; locality: Parque Nacional de Sete Cidades, Cachoeira do Riachão; maximumElevationInMeters: 171; verbatimCoordinates: 4°6'28"S, 41°40'13"W; **Identification:** identifiedBy: Allan Paulo Moreira dos Santos; **Event:** samplingProtocol: Pennsylvania light trap; verbatimEventDate: 18.iv.12; **Record Level:** institutionCode: DZRJ; basisOfRecord: PreservedSpecimen
- **Type status:** Other material. **Occurrence:** recordedBy: Takiya, D.M.; individualCount: 8; sex: male; lifeStage: adult; **Location:** country: Brazil; stateProvince: Piauí; municipality: Piracuruca; locality: Parque Nacional de Sete Cidades, Cachoeira do Riachão; maximumElevationInMeters: 171; verbatimCoordinates: 4°6'28"S, 41°40'13"W; **Identification:** identifiedBy: Ana Lucia Henriques Oliveira; **Event:** samplingProtocol: Pennsylvania light trap; verbatimEventDate: 18.iv.12; **Record Level:** institutionCode: DZRJ; basisOfRecord: PreservedSpecimen
- **Type status:** Other material. **Occurrence:** recordedBy: Takiya, D.M.; individualCount: 3; sex: male; lifeStage: adult; **Location:** country: Brazil; stateProvince: Piauí; municipality: Piracuruca; locality: Parque Nacional de Sete Cidades, Cachoeira do Riachão; maximumElevationInMeters: 171; verbatimCoordinates: 4°6'28"S, 41°40'13"W; **Identification:** identifiedBy: Ana Lucia Henriques Oliveira; **Event:** samplingProtocol: Pennsylvania light trap; verbatimEventDate: 18.iv.12; **Record Level:** institutionCode: DZRJ; basisOfRecord: PreservedSpecimen
- **Type status:** Other material. **Occurrence:** recordedBy: Takiya, D.M.; individualCount: 8; sex: male; lifeStage: adult; **Location:** country: Brazil; stateProvince: Piauí; municipality: Piracuruca; locality: Parque Nacional de Sete Cidades, Cachoeira do Riachão; maximumElevationInMeters: 171; verbatimCoordinates: 4°6'28"S, 41°40'13"W; **Identification:** identifiedBy: Ana Lucia Henriques Oliveira; **Event:** samplingProtocol: Pennsylvania light trap; verbatimEventDate: 18.iv.12; **Record Level:** institutionCode: DZRJ; basisOfRecord: PreservedSpecimen
- **Type status:** Other material. **Occurrence:** recordedBy: Takiya, D.M.; individualCount: 1; sex: male; lifeStage: adult; **Location:** country: Brazil; stateProvince: Piauí; municipality: Piracuruca; locality: Parque Nacional de Sete Cidades, Riacho da Piedade; maximumElevationInMeters: 169; verbatimCoordinates: 4°6'34"S, 41°43'39"W; **Identification:** identifiedBy: Ana Lucia Henriques Oliveira; **Event:** samplingProtocol: Pennsylvania light trap; verbatimEventDate: 19.iv.12; **Record Level:** institutionCode: DZRJ; basisOfRecord: PreservedSpecimen

##### Distribution

Venezuela. Guyana. Suriname. Brazil: RR, PI!, MG, SP, RJ. Ecuador. Peru. Bolivia. Paraguay. Argentina.

##### Notes

New species record for Northeastern Brazil.

#### Nectopsyche sp.

##### Materials

- **Type status:** Other material. **Occurrence:** recordedBy: Takiya, D.M.; individualCount: 1; sex: male; lifeStage: adult; **Location:** country: Brazil; stateProvince: Piauí; municipality: Piracuruca; locality: Parque Nacional de Sete Cidades, Alojamento; maximumElevationInMeters: 193; verbatimCoordinates: 4°5'57"S, 41°42'34"W; **Identification:** identifiedBy: Allan Paulo Moreira dos Santos; **Event:** samplingProtocol: White sheet light trap; verbatimEventDate: 20.iv.12; **Record Level:** institutionCode: DZRJ; basisOfRecord: PreservedSpecimen
- **Type status:** Other material. **Occurrence:** recordedBy: Takiya, D.M.; individualCount: 1; sex: male; lifeStage: adult; **Location:** country: Brazil; stateProvince: Piauí; municipality: Piracuruca; locality: Parque Nacional de Sete Cidades, Alojamento; maximumElevationInMeters: 193; verbatimCoordinates: 4°5'57"S, 41°42'34"W; **Identification:** identifiedBy: Ana Lucia Henriques Oliveira; **Event:** samplingProtocol: White sheet light trap; verbatimEventDate: 20.iv.12; **Record Level:** institutionCode: DZRJ; basisOfRecord: PreservedSpecimen

##### Notes

Undescribed species.

#### Oecetis

McLachlan, 1877

#### Oecetis sp. 1

##### Materials

- **Type status:** Other material. **Occurrence:** recordedBy: Takiya, D.M.; individualCount: 1; sex: male; lifeStage: adult; **Location:** country: Brazil; stateProvince: Piauí; municipality: Piracuruca; locality: Parque Nacional de Sete Cidades, Cachoeira do Riachão; maximumElevationInMeters: 171; verbatimCoordinates: 4°6'28"S, 41°40'13"W; **Identification:** identifiedBy: Ana Lucia Henriques Oliveira; **Event:** samplingProtocol: Pennsylvania light trap; verbatimEventDate: 18.iv.12; **Record Level:** institutionCode: DZRJ; basisOfRecord: PreservedSpecimen

##### Notes

Undescribed species.

#### Oecetis connata

Flint, 1974

##### Materials

- **Type status:** Other material. **Occurrence:** recordedBy: Takiya, D.M.; individualCount: 4; sex: male; lifeStage: adult; **Location:** country: Brazil; stateProvince: Piauí; municipality: Piracuruca; locality: Parque Nacional de Sete Cidades, Riacho da Bananeira; maximumElevationInMeters: 189; verbatimCoordinates: 4°5'59"S, 41°40'48"W; **Identification:** identifiedBy: Ana Lucia Henriques Oliveira; **Event:** samplingProtocol: Pennsylvania light trap; verbatimEventDate: 18.iv.12; **Record Level:** institutionCode: DZRJ; basisOfRecord: PreservedSpecimen
- **Type status:** Other material. **Occurrence:** recordedBy: Takiya, D.M.; individualCount: 1; sex: female; lifeStage: adult; **Location:** country: Brazil; stateProvince: Piauí; municipality: Piracuruca; locality: Parque Nacional de Sete Cidades, Riacho da Bananeira; maximumElevationInMeters: 189; verbatimCoordinates: 4°5'59"S, 41°40'48"W; **Identification:** identifiedBy: Ana Lucia Henriques Oliveira; **Event:** samplingProtocol: Pennsylvania light trap; verbatimEventDate: 18.iv.12; **Record Level:** institutionCode: DZRJ; basisOfRecord: PreservedSpecimen
- **Type status:** Other material. **Occurrence:** recordedBy: Rafael, J.A. | Limeira-de-Oliveira, F. | Takiya, D.M. | et al.; individualCount: 2; sex: male; lifeStage: adult; **Location:** country: Brazil; stateProvince: Piauí; municipality: Piracuruca; locality: Parque Nacional de Sete Cidades, Riacho da Bananeira; maximumElevationInMeters: 189; verbatimCoordinates: 4°5'59"S, 41°40'48"W; **Identification:** identifiedBy: Ana Lucia Henriques Oliveira; **Event:** samplingProtocol: Malaise intercept trap; verbatimEventDate: 18.iv.12; **Record Level:** institutionCode: DZRJ; basisOfRecord: PreservedSpecimen
- **Type status:** Other material. **Occurrence:** recordedBy: Takiya, D.M.; individualCount: 3; sex: male; lifeStage: adult; **Location:** country: Brazil; stateProvince: Piauí; municipality: Piracuruca; locality: Parque Nacional de Sete Cidades, Cachoeira do Riachão; maximumElevationInMeters: 171; verbatimCoordinates: 4°6'28"S, 41°40'13"W; **Identification:** identifiedBy: Ana Lucia Henriques Oliveira; **Event:** samplingProtocol: Pennsylvania light trap; verbatimEventDate: 18.iv.12; **Record Level:** institutionCode: DZRJ; basisOfRecord: PreservedSpecimen
- **Type status:** Other material. **Occurrence:** recordedBy: Takiya, D.M.; individualCount: 5; sex: female; lifeStage: adult; **Location:** country: Brazil; stateProvince: Piauí; municipality: Piracuruca; locality: Parque Nacional de Sete Cidades, Cachoeira do Riachão; maximumElevationInMeters: 171; verbatimCoordinates: 4°6'28"S, 41°40'13"W; **Identification:** identifiedBy: Ana Lucia Henriques Oliveira; **Event:** samplingProtocol: Pennsylvania light trap; verbatimEventDate: 18.iv.12; **Record Level:** institutionCode: DZRJ; basisOfRecord: PreservedSpecimen
- **Type status:** Other material. **Occurrence:** recordedBy: Takiya, D.M.; individualCount: 1; sex: male; lifeStage: adult; **Location:** country: Brazil; stateProvince: Piauí; municipality: Piracuruca; locality: Parque Nacional de Sete Cidades, Cachoeira do Riachão; maximumElevationInMeters: 171; verbatimCoordinates: 4°6'28"S, 41°40'13"W; **Identification:** identifiedBy: Ana Lucia Henriques Oliveira; **Event:** samplingProtocol: Pennsylvania light trap; verbatimEventDate: 18.iv.12; **Record Level:** institutionCode: DZRJ; basisOfRecord: PreservedSpecimen
- **Type status:** Other material. **Occurrence:** recordedBy: Takiya, D.M.; individualCount: 3; sex: female; lifeStage: adult; **Location:** country: Brazil; stateProvince: Piauí; municipality: Piracuruca; locality: Parque Nacional de Sete Cidades, Cachoeira do Riachão; maximumElevationInMeters: 171; verbatimCoordinates: 4°6'28"S, 41°40'13"W; **Identification:** identifiedBy: Ana Lucia Henriques Oliveira; **Event:** samplingProtocol: Pennsylvania light trap; verbatimEventDate: 18.iv.12; **Record Level:** institutionCode: DZRJ; basisOfRecord: PreservedSpecimen
- **Type status:** Other material. **Occurrence:** recordedBy: Takiya, D.M.; individualCount: 1; sex: female; lifeStage: adult; **Location:** country: Brazil; stateProvince: Piauí; municipality: Piracuruca; locality: Parque Nacional de Sete Cidades, Alojamento; maximumElevationInMeters: 193; verbatimCoordinates: 4°5'57"S, 41°42'34"W; **Identification:** identifiedBy: Ana Lucia Henriques Oliveira; **Event:** samplingProtocol: White sheet light trap; verbatimEventDate: 18.iv.12; **Record Level:** institutionCode: DZRJ; basisOfRecord: PreservedSpecimen
- **Type status:** Other material. **Occurrence:** recordedBy: Rafael, J.A. | Limeira-de-Oliveira, F. | Takiya, D.M. | et al.; individualCount: 1; sex: male; lifeStage: adult; **Location:** country: Brazil; stateProvince: Piauí; municipality: Piracuruca; locality: Parque Nacional de Sete Cidades, Riacho da Piedade; maximumElevationInMeters: 169; verbatimCoordinates: 4°6'34"S, 41°43'39"W; **Identification:** identifiedBy: Ana Lucia Henriques Oliveira; **Event:** samplingProtocol: Malaise intercept trap; verbatimEventDate: 18.iv.12; **Record Level:** institutionCode: DZRJ; basisOfRecord: PreservedSpecimen
- **Type status:** Other material. **Occurrence:** recordedBy: Rafael, J.A. | Limeira-de-Oliveira, F. | Takiya, D.M. | et al.; individualCount: 1; sex: female; lifeStage: adult; **Location:** country: Brazil; stateProvince: Piauí; municipality: Piracuruca; locality: Parque Nacional de Sete Cidades, Riacho da Piedade; maximumElevationInMeters: 169; verbatimCoordinates: 4°6'34"S, 41°43'39"W; **Identification:** identifiedBy: Ana Lucia Henriques Oliveira; **Event:** samplingProtocol: Malaise intercept trap; verbatimEventDate: 18.iv.12; **Record Level:** institutionCode: DZRJ; basisOfRecord: PreservedSpecimen
- **Type status:** Other material. **Occurrence:** recordedBy: Rafael, J.A. | Limeira-de-Oliveira, F. | Takiya, D.M. | et al.; individualCount: 1; sex: male; lifeStage: adult; **Location:** country: Brazil; stateProvince: Piauí; municipality: Piracuruca; locality: Parque Nacional de Sete Cidades, Riacho da Bananeira; maximumElevationInMeters: 189; verbatimCoordinates: 4°5'59"S, 41°40'48"W; **Identification:** identifiedBy: Ana Lucia Henriques Oliveira; **Event:** samplingProtocol: Malaise intercept trap; verbatimEventDate: 19.iv.12; **Record Level:** institutionCode: DZRJ; basisOfRecord: PreservedSpecimen
- **Type status:** Other material. **Occurrence:** recordedBy: Rafael, J.A. | Limeira-de-Oliveira, F. | Takiya, D.M. | et al.; individualCount: 1; sex: female; lifeStage: adult; **Location:** country: Brazil; stateProvince: Piauí; municipality: Piracuruca; locality: Parque Nacional de Sete Cidades, Riacho da Bananeira; maximumElevationInMeters: 189; verbatimCoordinates: 4°5'59"S, 41°40'48"W; **Identification:** identifiedBy: Ana Lucia Henriques Oliveira; **Event:** samplingProtocol: Malaise intercept trap; verbatimEventDate: 19.iv.12; **Record Level:** institutionCode: DZRJ; basisOfRecord: PreservedSpecimen
- **Type status:** Other material. **Occurrence:** recordedBy: Rafael, J.A. | Limeira-de-Oliveira, F. | Takiya, D.M. | et al.; individualCount: 2; sex: male; lifeStage: adult; **Location:** country: Brazil; stateProvince: Piauí; municipality: Piracuruca; locality: Parque Nacional de Sete Cidades, Riacho da Bananeira; maximumElevationInMeters: 189; verbatimCoordinates: 4°5'59"S, 41°40'48"W; **Identification:** identifiedBy: Ana Lucia Henriques Oliveira; **Event:** samplingProtocol: Malaise intercept trap; verbatimEventDate: 19.iv.12; **Record Level:** institutionCode: DZRJ; basisOfRecord: PreservedSpecimen
- **Type status:** Other material. **Occurrence:** recordedBy: Takiya, D.M.; individualCount: 4; sex: male; lifeStage: adult; **Location:** country: Brazil; stateProvince: Piauí; municipality: Piracuruca; locality: Parque Nacional de Sete Cidades, Riacho da Piedade; maximumElevationInMeters: 169; verbatimCoordinates: 4°6'34"S, 41°43'39"W; **Identification:** identifiedBy: Ana Lucia Henriques Oliveira; **Event:** samplingProtocol: Pennsylvania light trap; verbatimEventDate: 19.iv.12; **Record Level:** institutionCode: DZRJ; basisOfRecord: PreservedSpecimen
- **Type status:** Other material. **Occurrence:** recordedBy: Takiya, D.M.; individualCount: 1; sex: female; lifeStage: adult; **Location:** country: Brazil; stateProvince: Piauí; municipality: Piracuruca; locality: Parque Nacional de Sete Cidades, Riacho da Piedade; maximumElevationInMeters: 169; verbatimCoordinates: 4°6'34"S, 41°43'39"W; **Identification:** identifiedBy: Ana Lucia Henriques Oliveira; **Event:** samplingProtocol: Pennsylvania light trap; verbatimEventDate: 19.iv.12; **Record Level:** institutionCode: DZRJ; basisOfRecord: PreservedSpecimen
- **Type status:** Other material. **Occurrence:** recordedBy: Takiya, D.M.; individualCount: 38; sex: male; lifeStage: adult; **Location:** country: Brazil; stateProvince: Piauí; municipality: Piracuruca; locality: Parque Nacional de Sete Cidades, Alojamento; maximumElevationInMeters: 193; verbatimCoordinates: 4°5'57"S, 41°42'34"W; **Identification:** identifiedBy: Ana Lucia Henriques Oliveira; **Event:** samplingProtocol: White sheet light trap; verbatimEventDate: 20.iv.12; **Record Level:** institutionCode: DZRJ; basisOfRecord: PreservedSpecimen
- **Type status:** Other material. **Occurrence:** recordedBy: Takiya, D.M.; individualCount: 15; sex: female; lifeStage: adult; **Location:** country: Brazil; stateProvince: Piauí; municipality: Piracuruca; locality: Parque Nacional de Sete Cidades, Alojamento; maximumElevationInMeters: 193; verbatimCoordinates: 4°5'57"S, 41°42'34"W; **Identification:** identifiedBy: Ana Lucia Henriques Oliveira; **Event:** samplingProtocol: White sheet light trap; verbatimEventDate: 20.iv.12; **Record Level:** institutionCode: DZRJ; basisOfRecord: PreservedSpecimen
- **Type status:** Other material. **Occurrence:** recordedBy: Rafael, J.A. | Limeira-de-Oliveira, F. | Takiya, D.M. | et al.; individualCount: 2; sex: male; lifeStage: adult; **Location:** country: Brazil; stateProvince: Piauí; municipality: Piracuruca; locality: Parque Nacional de Sete Cidades, Riacho da Piedade; maximumElevationInMeters: 169; verbatimCoordinates: 4°6'34"S, 41°43'39"W; **Identification:** identifiedBy: Ana Lucia Henriques Oliveira; **Event:** samplingProtocol: Malaise intercept trap; verbatimEventDate: 21.iv.12; **Record Level:** institutionCode: DZRJ; basisOfRecord: PreservedSpecimen
- **Type status:** Other material. **Occurrence:** recordedBy: Rafael, J.A. | Limeira-de-Oliveira, F. | Takiya, D.M. | et al.; individualCount: 4; sex: female; lifeStage: adult; **Location:** country: Brazil; stateProvince: Piauí; municipality: Piracuruca; locality: Parque Nacional de Sete Cidades, Riacho da Piedade; maximumElevationInMeters: 169; verbatimCoordinates: 4°6'34"S, 41°43'39"W; **Identification:** identifiedBy: Ana Lucia Henriques Oliveira; **Event:** samplingProtocol: Malaise intercept trap; verbatimEventDate: 21.iv.12; **Record Level:** institutionCode: DZRJ; basisOfRecord: PreservedSpecimen

##### Distribution

Guyana. Suriname. Brazil: PA, AM, PI, MT, BA.

##### Notes

Species firstly recorded from PI in Henriques-Oliveira et al. 2014.

#### Philopotamidae

##### Notes

New family record for PI.

#### Chimarra

Stephens, 1829

##### Notes

New genus record for PI.

#### Chimarra (Chimarra) sp. 1

##### Materials

- **Type status:** Other material. **Occurrence:** recordedBy: Takiya, D.M.; individualCount: 2; sex: male; lifeStage: adult; **Location:** country: Brazil; stateProvince: Piauí; municipality: Piracuruca; locality: Parque Nacional de Sete Cidades, Cachoeira do Riachão; maximumElevationInMeters: 171; verbatimCoordinates: 4°6'28"S, 41°40'13"W; **Identification:** identifiedBy: Allan Paulo Moreira dos Santos; **Event:** samplingProtocol: Pennsylvania light trap; verbatimEventDate: 18.iv.12; **Record Level:** institutionCode: DZRJ; basisOfRecord: PreservedSpecimen
- **Type status:** Other material. **Occurrence:** recordedBy: Rafael, J.A. | Limeira-de-Oliveira, F. | Takiya, D.M. | et al.; individualCount: 1; sex: male; lifeStage: adult; **Location:** country: Brazil; stateProvince: Piauí; municipality: Piracuruca; locality: Parque Nacional de Sete Cidades, Cachoeira do Riachão; maximumElevationInMeters: 171; verbatimCoordinates: 4°6'28"S, 41°40'13"W; **Identification:** identifiedBy: Allan Paulo Moreira dos Santos; **Event:** samplingProtocol: Malaise intercept trap; verbatimEventDate: 18.iv.12; **Record Level:** institutionCode: DZRJ; basisOfRecord: PreservedSpecimen

##### Notes

Undescribed species.

#### Chimarra (Chimarra) sp. 2

##### Materials

- **Type status:** Other material. **Occurrence:** recordedBy: Rafael, J.A. | Limeira-de-Oliveira, F. | Takiya, D.M. | et al.; individualCount: 1; sex: male; lifeStage: adult; **Location:** country: Brazil; stateProvince: Piauí; municipality: Piracuruca; locality: Parque Nacional de Sete Cidades, Riacho da Piedade; maximumElevationInMeters: 169; verbatimCoordinates: 4°6'34"S, 41°43'39"W; **Identification:** identifiedBy: Allan Paulo Moreira dos Santos; **Event:** samplingProtocol: Malaise intercept trap; verbatimEventDate: 18.iv.12; **Record Level:** institutionCode: DZRJ; basisOfRecord: PreservedSpecimen
- **Type status:** Other material. **Occurrence:** recordedBy: Takiya, D.M.; individualCount: 1; sex: male; lifeStage: adult; **Location:** country: Brazil; stateProvince: Piauí; municipality: Piracuruca; locality: Parque Nacional de Sete Cidades, Cachoeira do Riachão; maximumElevationInMeters: 171; verbatimCoordinates: 4°6'28"S, 41°40'13"W; **Identification:** identifiedBy: Allan Paulo Moreira dos Santos; **Event:** samplingProtocol: Pennsylvania light trap; verbatimEventDate: 19.iv.12; **Record Level:** institutionCode: DZRJ; basisOfRecord: PreservedSpecimen

##### Notes

Undescribed species.

#### Chimarra (Chimarra) sp. 3

##### Materials

- **Type status:** Other material. **Occurrence:** recordedBy: Takiya, D.M.; individualCount: 1; sex: male; lifeStage: adult; **Location:** country: Brazil; stateProvince: Piauí; municipality: Piracuruca; locality: Parque Nacional de Sete Cidades, Olho d'água Piscina do Bacuri; maximumElevationInMeters: 171; verbatimCoordinates: 4°6'1.2"S, 41°42'38.8"W; **Identification:** identifiedBy: Allan Paulo Moreira dos Santos; **Event:** samplingProtocol: White sheet light trap; verbatimEventDate: 18.iv.12; **Record Level:** institutionCode: DZRJ; basisOfRecord: PreservedSpecimen
- **Type status:** Other material. **Occurrence:** recordedBy: Takiya, D.M.; individualCount: 1; sex: female; lifeStage: adult; **Location:** country: Brazil; stateProvince: Piauí; municipality: Piracuruca; locality: Parque Nacional de Sete Cidades, Olho d'água Piscina do Bacuri; maximumElevationInMeters: 171; verbatimCoordinates: 4°6'1.2"S, 41°42'38.8"W; **Identification:** identifiedBy: Allan Paulo Moreira dos Santos; **Event:** samplingProtocol: White sheet light trap; verbatimEventDate: 18.iv.12; **Record Level:** institutionCode: DZRJ; basisOfRecord: PreservedSpecimen
- **Type status:** Other material. **Occurrence:** recordedBy: Takiya, D.M.; individualCount: 2; sex: male; lifeStage: adult; **Location:** country: Brazil; stateProvince: Piauí; municipality: Piracuruca; locality: Parque Nacional de Sete Cidades, Cachoeira do Riachão; maximumElevationInMeters: 171; verbatimCoordinates: 4°6'28"S, 41°40'13"W; **Identification:** identifiedBy: Allan Paulo Moreira dos Santos; **Event:** samplingProtocol: Pennsylvania light trap; verbatimEventDate: 19.iv.12; **Record Level:** institutionCode: DZRJ; basisOfRecord: PreservedSpecimen
- **Type status:** Other material. **Occurrence:** recordedBy: Takiya, D.M.; individualCount: 2; sex: male; lifeStage: adult; **Location:** country: Brazil; stateProvince: Piauí; municipality: Piracuruca; locality: Parque Nacional de Sete Cidades, Centro de visitantes; maximumElevationInMeters: 202; verbatimCoordinates: 4°6'20"S, 41°41'52"W; **Identification:** identifiedBy: Allan Paulo Moreira dos Santos; **Event:** samplingProtocol: White sheet light trap; verbatimEventDate: 19.iv.12; **Record Level:** institutionCode: DZRJ; basisOfRecord: PreservedSpecimen

##### Notes

Undescribed species.

#### Chimarra (Curgia) jugescens

Flint, 1998

##### Materials

- **Type status:** Other material. **Occurrence:** recordedBy: Rafael, J.A. | Limeira-de-Oliveira, F. | Takiya, D.M. | et al.; individualCount: 1; sex: male; lifeStage: adult; **Location:** country: Brazil; stateProvince: Piauí; municipality: Piracuruca; locality: Parque Nacional de Sete Cidades, Riacho da Bananeira; maximumElevationInMeters: 189; verbatimCoordinates: 4°5'59"S, 41°40'48"W; **Identification:** identifiedBy: Allan Paulo Moreira dos Santos; **Event:** samplingProtocol: Malaise intercept trap; verbatimEventDate: 18.iv.12; **Record Level:** institutionCode: DZRJ; basisOfRecord: PreservedSpecimen
- **Type status:** Other material. **Occurrence:** recordedBy: Rafael, J.A. | Limeira-de-Oliveira, F. | Takiya, D.M. | et al.; individualCount: 2; sex: male; lifeStage: adult; **Location:** country: Brazil; stateProvince: Piauí; municipality: Piracuruca; locality: Parque Nacional de Sete Cidades, Cachoeira do Riachão; maximumElevationInMeters: 171; verbatimCoordinates: 4°6'28"S, 41°40'13"W; **Identification:** identifiedBy: Allan Paulo Moreira dos Santos; **Event:** samplingProtocol: Malaise intercept trap; verbatimEventDate: 18.iv.12; **Record Level:** institutionCode: DZRJ; basisOfRecord: PreservedSpecimen
- **Type status:** Other material. **Occurrence:** recordedBy: Rafael, J.A. | Limeira-de-Oliveira, F. | Takiya, D.M. | et al.; individualCount: 3; sex: male; lifeStage: adult; **Location:** country: Brazil; stateProvince: Piauí; municipality: Piracuruca; locality: Parque Nacional de Sete Cidades, Riacho da Bananeira; maximumElevationInMeters: 189; verbatimCoordinates: 4°5'59"S, 41°40'48"W; **Identification:** identifiedBy: Allan Paulo Moreira dos Santos; **Event:** samplingProtocol: Malaise intercept trap; verbatimEventDate: 19.iv.12; **Record Level:** institutionCode: DZRJ; basisOfRecord: PreservedSpecimen
- **Type status:** Other material. **Occurrence:** recordedBy: Rafael, J.A. | Limeira-de-Oliveira, F. | Takiya, D.M. | et al.; individualCount: 3; sex: female; lifeStage: adult; **Location:** country: Brazil; stateProvince: Piauí; municipality: Piracuruca; locality: Parque Nacional de Sete Cidades, Riacho da Bananeira; maximumElevationInMeters: 189; verbatimCoordinates: 4°5'59"S, 41°40'48"W; **Identification:** identifiedBy: Allan Paulo Moreira dos Santos; **Event:** samplingProtocol: Malaise intercept trap; verbatimEventDate: 19.iv.12; **Record Level:** institutionCode: DZRJ; basisOfRecord: PreservedSpecimen

##### Distribution

Brazil: PA, AM, PI!.

##### Notes

New species record for Northeastern Brazil.

#### Polycentropodidae

##### Notes

New family record for PI.

#### Cernotina

Ross, 1938

##### Notes

New genus record for PI.

#### Cernotina sp. 1

##### Materials

- **Type status:** Other material. **Occurrence:** recordedBy: Rafael, J.A. | Limeira-de-Oliveira, F. | Takiya, D.M. | et al.; individualCount: 1; sex: male; lifeStage: adult; **Location:** country: Brazil; stateProvince: Piauí; municipality: Piracuruca; locality: Parque Nacional de Sete Cidades, Riacho da Piedade; maximumElevationInMeters: 169; verbatimCoordinates: 4°6'34"S, 41°43'39"W; **Identification:** identifiedBy: Allan Paulo Moreira dos Santos; **Event:** samplingProtocol: Malaise intercept trap; verbatimEventDate: 18.iv.12; **Record Level:** institutionCode: DZRJ; basisOfRecord: PreservedSpecimen
- **Type status:** Other material. **Occurrence:** recordedBy: Takiya, D.M.; individualCount: 1; sex: male; lifeStage: adult; **Location:** country: Brazil; stateProvince: Piauí; municipality: Piracuruca; locality: Parque Nacional de Sete Cidades, Riacho da Piedade; maximumElevationInMeters: 169; verbatimCoordinates: 4°6'34"S, 41°43'39"W; **Identification:** identifiedBy: Allan Paulo Moreira dos Santos; **Event:** samplingProtocol: Pennsylvania light trap; verbatimEventDate: 19.iv.12; **Record Level:** institutionCode: DZRJ; basisOfRecord: PreservedSpecimen
- **Type status:** Other material. **Occurrence:** recordedBy: Takiya, D.M.; individualCount: 1; sex: female; lifeStage: adult; **Location:** country: Brazil; stateProvince: Piauí; municipality: Piracuruca; locality: Parque Nacional de Sete Cidades, Riacho da Piedade; maximumElevationInMeters: 169; verbatimCoordinates: 4°6'34"S, 41°43'39"W; **Identification:** identifiedBy: Allan Paulo Moreira dos Santos; **Event:** samplingProtocol: Pennsylvania light trap; verbatimEventDate: 19.iv.12; **Record Level:** institutionCode: DZRJ; basisOfRecord: PreservedSpecimen
- **Type status:** Other material. **Occurrence:** recordedBy: Rafael, J.A. | Limeira-de-Oliveira, F. | Takiya, D.M. | et al.; individualCount: 4; sex: male; lifeStage: adult; **Location:** country: Brazil; stateProvince: Piauí; municipality: Piracuruca; locality: Parque Nacional de Sete Cidades, Riacho da Piedade; maximumElevationInMeters: 169; verbatimCoordinates: 4°6'34"S, 41°43'39"W; **Identification:** identifiedBy: Allan Paulo Moreira dos Santos; **Event:** samplingProtocol: Malaise intercept trap; verbatimEventDate: 21.iv.12; **Record Level:** institutionCode: DZRJ; basisOfRecord: PreservedSpecimen

#### Cernotina sp. 2*

##### Materials

- **Type status:** Other material. **Occurrence:** recordedBy: Rafael, J.A. | Limeira-de-Oliveira, F. | Takiya, D.M. | et al.; individualCount: 1; sex: male; lifeStage: adult; **Location:** country: Brazil; stateProvince: Piauí; municipality: Piracuruca; locality: Parque Nacional de Sete Cidades, Riacho da Piedade; maximumElevationInMeters: 169; verbatimCoordinates: 4°6'34"S, 41°43'39"W; **Identification:** identifiedBy: Allan Paulo Moreira dos Santos; **Event:** samplingProtocol: Malaise intercept trap; verbatimEventDate: 18.iv.12; **Record Level:** institutionCode: DZRJ; basisOfRecord: PreservedSpecimen
- **Type status:** Other material. **Occurrence:** recordedBy: Rafael, J.A. | Limeira-de-Oliveira, F. | Takiya, D.M. | et al.; individualCount: 2; sex: male; lifeStage: adult; **Location:** country: Brazil; stateProvince: Piauí; municipality: Piracuruca; locality: Parque Nacional de Sete Cidades, Riacho da Piedade; maximumElevationInMeters: 169; verbatimCoordinates: 4°6'34"S, 41°43'39"W; **Identification:** identifiedBy: Allan Paulo Moreira dos Santos; **Event:** samplingProtocol: Malaise intercept trap; verbatimEventDate: 21.iv.12; **Record Level:** institutionCode: DZRJ; basisOfRecord: PreservedSpecimen
- **Type status:** Other material. **Occurrence:** recordedBy: Rafael, J.A. | Limeira-de-Oliveira, F. | Takiya, D.M. | et al.; individualCount: 2; sex: female; lifeStage: adult; **Location:** country: Brazil; stateProvince: Piauí; municipality: Piracuruca; locality: Parque Nacional de Sete Cidades, Riacho da Piedade; maximumElevationInMeters: 169; verbatimCoordinates: 4°6'34"S, 41°43'39"W; **Identification:** identifiedBy: Allan Paulo Moreira dos Santos; **Event:** samplingProtocol: Malaise intercept trap; verbatimEventDate: 21.iv.12; **Record Level:** institutionCode: DZRJ; basisOfRecord: PreservedSpecimen
- **Type status:** Other material. **Occurrence:** recordedBy: Rafael, J.A. | Limeira-de-Oliveira, F. | Takiya, D.M. | et al.; individualCount: 4; sex: male; lifeStage: adult; **Location:** country: Brazil; stateProvince: Piauí; municipality: Piracuruca; locality: Parque Nacional de Sete Cidades, Riacho da Piedade; maximumElevationInMeters: 169; verbatimCoordinates: 4°6'34"S, 41°43'39"W; **Identification:** identifiedBy: Allan Paulo Moreira dos Santos; **Event:** samplingProtocol: Malaise intercept trap; verbatimEventDate: 21.iv.12; **Record Level:** institutionCode: DZRJ; basisOfRecord: PreservedSpecimen
- **Type status:** Other material. **Occurrence:** recordedBy: Rafael, J.A. | Limeira-de-Oliveira, F. | Takiya, D.M. | et al.; individualCount: 3; sex: female; lifeStage: adult; **Location:** country: Brazil; stateProvince: Piauí; municipality: Piracuruca; locality: Parque Nacional de Sete Cidades, Riacho da Piedade; maximumElevationInMeters: 169; verbatimCoordinates: 4°6'34"S, 41°43'39"W; **Identification:** identifiedBy: Allan Paulo Moreira dos Santos; **Event:** samplingProtocol: Malaise intercept trap; verbatimEventDate: 21.iv.12; **Record Level:** institutionCode: DZRJ; basisOfRecord: PreservedSpecimen
- **Type status:** Other material. **Occurrence:** recordedBy: Rafael, J.A. | Limeira-de-Oliveira, F. | Takiya, D.M. | et al.; individualCount: 67; sex: male; lifeStage: adult; **Location:** country: Brazil; stateProvince: Piauí; municipality: Piracuruca; locality: Parque Nacional de Sete Cidades, Riacho da Piedade; maximumElevationInMeters: 169; verbatimCoordinates: 4°6'34"S, 41°43'39"W; **Identification:** identifiedBy: Allan Paulo Moreira dos Santos; **Event:** samplingProtocol: Malaise intercept trap; verbatimEventDate: 21.iv.12; **Record Level:** institutionCode: DZRJ; basisOfRecord: PreservedSpecimen

#### Cyrnellus

Banks, 1913

##### Notes

New genus record for PI.

#### Cyrnellus fraternus

(Banks, 1915)

##### Materials

- **Type status:** Other material. **Occurrence:** recordedBy: Santos, A.P.M. | Takiya, D.M.; individualCount: 1; sex: male; lifeStage: adult; **Location:** country: Brazil; stateProvince: Piauí; municipality: Piracuruca; locality: Parque Nacional de Sete Cidades, Cachoeira do Riachão; maximumElevationInMeters: 171; verbatimCoordinates: 4°6'28"S, 41°40'13"W; **Identification:** identifiedBy: Allan Paulo Moreira dos Santos; **Event:** samplingProtocol: Pennsylvania light trap; verbatimEventDate: 10.ii.13; **Record Level:** institutionCode: DZRJ; basisOfRecord: PreservedSpecimen
- **Type status:** Other material. **Occurrence:** recordedBy: Santos, A.P.M. | Takiya, D.M.; individualCount: 5; sex: female; lifeStage: adult; **Location:** country: Brazil; stateProvince: Piauí; municipality: Piracuruca; locality: Parque Nacional de Sete Cidades, Cachoeira do Riachão; maximumElevationInMeters: 171; verbatimCoordinates: 4°6'28"S, 41°40'13"W; **Identification:** identifiedBy: Allan Paulo Moreira dos Santos; **Event:** samplingProtocol: Pennsylvania light trap; verbatimEventDate: 10.ii.13; **Record Level:** institutionCode: DZRJ; basisOfRecord: PreservedSpecimen
- **Type status:** Other material. **Occurrence:** recordedBy: Santos, A.P.M. | Takiya, D.M.; individualCount: 8; sex: male; lifeStage: adult; **Location:** country: Brazil; stateProvince: Piauí; municipality: Piracuruca; locality: Parque Nacional de Sete Cidades, Poço do Bananeira; maximumElevationInMeters: 158; verbatimCoordinates: 4°5'55.8"S, 41°40'33.8"W; **Identification:** identifiedBy: Allan Paulo Moreira dos Santos; **Event:** samplingProtocol: Pennsylvania light trap; verbatimEventDate: 10.ii.13; **Record Level:** institutionCode: DZRJ; basisOfRecord: PreservedSpecimen
- **Type status:** Other material. **Occurrence:** recordedBy: Santos, A.P.M. | Takiya, D.M.; individualCount: 2; sex: female; lifeStage: adult; **Location:** country: Brazil; stateProvince: Piauí; municipality: Piracuruca; locality: Parque Nacional de Sete Cidades, Poço do Bananeira; maximumElevationInMeters: 158; verbatimCoordinates: 4°5'55.8"S, 41°40'33.8"W; **Identification:** identifiedBy: Allan Paulo Moreira dos Santos; **Event:** samplingProtocol: Pennsylvania light trap; verbatimEventDate: 10.ii.13; **Record Level:** institutionCode: DZRJ; basisOfRecord: PreservedSpecimen
- **Type status:** Other material. **Occurrence:** recordedBy: Takiya, D.M.; individualCount: 2; sex: male; lifeStage: adult; **Location:** country: Brazil; stateProvince: Piauí; municipality: Piracuruca; locality: Parque Nacional de Sete Cidades, Riacho da Bananeira; maximumElevationInMeters: 189; verbatimCoordinates: 4°5'59"S, 41°40'48"W; **Identification:** identifiedBy: Allan Paulo Moreira dos Santos; **Event:** samplingProtocol: Pennsylvania light trap; verbatimEventDate: 18.iv.12; **Record Level:** institutionCode: DZRJ; basisOfRecord: PreservedSpecimen
- **Type status:** Other material. **Occurrence:** recordedBy: Takiya, D.M.; individualCount: 2; sex: female; lifeStage: adult; **Location:** country: Brazil; stateProvince: Piauí; municipality: Piracuruca; locality: Parque Nacional de Sete Cidades, Riacho da Bananeira; maximumElevationInMeters: 189; verbatimCoordinates: 4°5'59"S, 41°40'48"W; **Identification:** identifiedBy: Allan Paulo Moreira dos Santos; **Event:** samplingProtocol: Pennsylvania light trap; verbatimEventDate: 18.iv.12; **Record Level:** institutionCode: DZRJ; basisOfRecord: PreservedSpecimen
- **Type status:** Other material. **Occurrence:** recordedBy: Rafael, J.A. | Limeira-de-Oliveira, F. | Takiya, D.M. | et al.; individualCount: 5; sex: male; lifeStage: adult; **Location:** country: Brazil; stateProvince: Piauí; municipality: Piracuruca; locality: Parque Nacional de Sete Cidades, Riacho da Bananeira; maximumElevationInMeters: 189; verbatimCoordinates: 4°5'59"S, 41°40'48"W; **Identification:** identifiedBy: Allan Paulo Moreira dos Santos; **Event:** samplingProtocol: Malaise intercept trap; verbatimEventDate: 19.iv.12; **Record Level:** institutionCode: DZRJ; basisOfRecord: PreservedSpecimen
- **Type status:** Other material. **Occurrence:** recordedBy: Rafael, J.A. | Limeira-de-Oliveira, F. | Takiya, D.M. | et al.; individualCount: 1; sex: female; lifeStage: adult; **Location:** country: Brazil; stateProvince: Piauí; municipality: Piracuruca; locality: Parque Nacional de Sete Cidades, Riacho da Bananeira; maximumElevationInMeters: 189; verbatimCoordinates: 4°5'59"S, 41°40'48"W; **Identification:** identifiedBy: Allan Paulo Moreira dos Santos; **Event:** samplingProtocol: Malaise intercept trap; verbatimEventDate: 19.iv.12; **Record Level:** institutionCode: DZRJ; basisOfRecord: PreservedSpecimen
- **Type status:** Other material. **Occurrence:** recordedBy: Santos, A.P.M. | Takiya, D.M.; individualCount: 1; sex: male; lifeStage: adult; **Location:** country: Brazil; stateProvince: Piauí; municipality: Piracuruca; locality: Parque Nacional de Sete Cidades, Cachoeira do Riachão; maximumElevationInMeters: 171; verbatimCoordinates: 4°6'28"S, 41°40'13"W; **Identification:** identifiedBy: Allan Paulo Moreira dos Santos; **Event:** samplingProtocol: Pennsylvania light trap; verbatimEventDate: 8.ii.13; **Record Level:** institutionCode: DZRJ; basisOfRecord: PreservedSpecimen
- **Type status:** Other material. **Occurrence:** recordedBy: Santos, A.P.M. | Takiya, D.M.; individualCount: 3; sex: male; lifeStage: adult; **Location:** country: Brazil; stateProvince: Piauí; municipality: Piracuruca; locality: Parque Nacional de Sete Cidades, Poço do Bananeira; maximumElevationInMeters: 158; verbatimCoordinates: 4°5'55.8"S, 41°40'33.8"W; **Identification:** identifiedBy: Allan Paulo Moreira dos Santos; **Event:** samplingProtocol: Pennsylvania light trap; verbatimEventDate: 9.ii.13; **Record Level:** institutionCode: DZRJ; basisOfRecord: PreservedSpecimen
- **Type status:** Other material. **Occurrence:** recordedBy: Santos, A.P.M. | Takiya, D.M.; individualCount: 1; sex: female; lifeStage: adult; **Location:** country: Brazil; stateProvince: Piauí; municipality: Piracuruca; locality: Parque Nacional de Sete Cidades, Poço do Bananeira; maximumElevationInMeters: 158; verbatimCoordinates: 4°5'55.8"S, 41°40'33.8"W; **Identification:** identifiedBy: Allan Paulo Moreira dos Santos; **Event:** samplingProtocol: Pennsylvania light trap; verbatimEventDate: 9.ii.13; **Record Level:** institutionCode: DZRJ; basisOfRecord: PreservedSpecimen

##### Distribution

USA. Mexico. El Salvador. Nicaragua. Costa Rica. Panama. Venezuela. Suriname. Brazil: PA, AM, PI!, BA, MG, MS, ES, RJ, PR, SC. Ecuador. Paraguay. Argentina. Uruguay.

##### Notes

New species record for PI.

#### Polyplectropus

Ulmer, 1905

##### Notes

New genus record for PI.

#### Polyplectropus rondoniensis

Chamorro & Holzenthal, 2010

##### Materials

- **Type status:** Other material. **Occurrence:** recordedBy: Rafael, J.A. | Limeira-de-Oliveira, F. | Takiya, D.M. | et al.; individualCount: 1; sex: male; lifeStage: adult; **Location:** country: Brazil; stateProvince: Piauí; municipality: Piracuruca; locality: Parque Nacional de Sete Cidades, Riacho da Piedade; maximumElevationInMeters: 169; verbatimCoordinates: 4°6'34"S, 41°43'39"W; **Identification:** identifiedBy: Allan Paulo Moreira dos Santos; **Event:** samplingProtocol: Malaise intercept trap; verbatimEventDate: 21.iv.12; **Record Level:** institutionCode: DZRJ; basisOfRecord: PreservedSpecimen

##### Distribution

Brazil: PI!, RO.

##### Notes

New species record for Northeastern Brazil.

#### Xiphocentronidae

##### Notes

New family record for PI.

#### Xiphocentron

Brauer, 1870

##### Notes

New genus record for PI.

#### Xiphocentron sp. 2

##### Materials

- **Type status:** Other material. **Occurrence:** recordedBy: Santos, A.P.M. | Takiya, D.M.; individualCount: 9; lifeStage: immature; **Location:** country: Brazil; stateProvince: Piauí; municipality: Piracuruca; locality: Parque Nacional de Sete Cidades, Cachoeira do Riachão; maximumElevationInMeters: 171; verbatimCoordinates: 4°6'28"S, 41°40'13"W; **Identification:** identifiedBy: Allan Paulo Moreira dos Santos; **Event:** samplingProtocol: Manual; verbatimEventDate: 11.ii.13; **Record Level:** institutionCode: DZRJ; basisOfRecord: PreservedSpecimen
- **Type status:** Other material. **Occurrence:** recordedBy: Santos, A.P.M. | Takiya, D.M.; individualCount: 23; lifeStage: immature; **Location:** country: Brazil; stateProvince: Piauí; municipality: Piracuruca; locality: Parque Nacional de Sete Cidades, Cachoeira do Riachão; maximumElevationInMeters: 171; verbatimCoordinates: 4°6'28"S, 41°40'13"W; **Identification:** identifiedBy: Allan Paulo Moreira dos Santos; **Event:** samplingProtocol: Manual; verbatimEventDate: 9.ii.13; **Record Level:** institutionCode: DZRJ; basisOfRecord: PreservedSpecimen

## Discussion

### Species shared with other phytogeographical domains

Although the Caatinga shares with Cerrado similar landscapes, in part due its savannah-like formation and their geographical proximity, under a spatial evolution standpoint historically the biota from Northeastern Brazil forested areas are largely linked to Amazonia and Atlantic Forest (see Santos et al. 2007) and even appears disjunct from other savannah-like South American formations such as the Bolivian Chaco (Werneck et al. 2012). Considering the better known Odonata, the composition of the dragonfly assemblage is remarkable by virtually lacking typical Cerrado species and mixing elements from forested biomes of Amazonia and Atlantic Forest domains. Sampling from the two National Parks comprises largely common species occurring in lentic environments of almost all American countries (*e.g.*, *Ischnura capreolus* at PNSC and *Erythrodiplax fusca* at PNU), while a smaller number of species were only previously known from Amazonia (*e.g.*, *Castoraeschna corbeti* at PNU; Carvalho et al. 2009) or from Atlantic Forest (*e.g.*, *Neocordulia setifera* at PNU; Pinto and Lamas 2010). Some of them are largely common and characteristic of these domains, such as *P. complicatus* in the Atlantic Forest (see distribution data in De Almeida et al. 2013).

Past connections between Amazonia and Atlantic Forest biotas are open to debate, hypotheses diverging if they occurred during the Miocene through South America's dry diagonal vegetation, including currently the Cerrado and Chaco formations, or during the Plio–Pleistocene through forested areas in the Caatinga Domain (see references in Batalha-Filho et al. 2013). Furthermore, wet period intervals every 20,000-200,000 years during at least 2 million years created intermittent corridors of between forested areas in Brazil (Wang et al. 2004) and can explain the mixing of fauna among Amazonia-Caatinga-Atlantic Forest. At least the PNU can be considered an island of humid forest surrounded by savannah-like formation and studies focusing the aquatic insect assemblages can strongly help understand the spatial evolution of these forested patches in the Northeastern Brazil and past connections between the two largest tropical forest domains in South America.

### New distributional records and notes on species records

Based on the collected material during this project, one caddisfly species was firstly recorded from Brazil, *Phylloicus pirapo* (Calamoceratidae) previously known from Argentina and Paraguay. Eighteen species collected at PNU and 21 at PNSC (5 species shared between parks) represent their first record for Northeastern Brazil.

Several new state records are made based on this material, especially for Piauí State. Thirty and 56 species are firstly recorded from Ceará and Piauí states, respectively. These exclude records previously published, but based on material collected during this project, which add other 10 new species records for these states (see Table 2). Herein, it is also recorded 25 and 47 new genera and 9 and 14 aquatic insect families from Ceará and Piauí states, respectively.

Some of these new records are for widespread species in South America, such as for many dragonflies (see below), however, others significantly expand species ranges in Brazil. For example, the caddisflies *Helicopsyche monda* (Helicopsychidae) and *Chimarra calori* (Philopotamidae) previously known from Southeastern and/or Southern Brazil have their ranges expanded for over 1,000 km.

The knowledge of stoneflies from Northeastern Brazil is still very scarce, when compared to the other aquatic insects studied. In the Neotropical Plecoptera catalogue, only two species, *Anacroneuria lacunosa* (Navás, 1926) and *A. parilobata* Klapálek, 1922, from this Brazilian Region was recorded (Froehlich 2010), both from Bahia State. In the last ten years, following an increase of local taxonomists and inventories, species number boosted from two to 27 stoneflies (Lecci and Froehlich 2011, Righi-Cavallaro et al. 2013, Duarte et al. 2014, Duarte and Lecci 2014, Lecci et al. 2014, Duarte and Lecci 2016). However, most sampling localities are still concentrated in some regions of Bahia State. Nevertheless, stonefly diversity still seems higher in the Atlantic forest, than in the Caatinga. A single *Anacroneuria* species has been recently described fom PNU (Duarte and Lecci 2016) and was recorded herein from both National parks, thus representing the first record of Plecoptera from Piauí State.

Except by few Amazonian and Atlantic Forest representatives, most dragonflies correspond to common species and are now firstly recorded mainly due to undersampling. Thus, field surveys must be one of the priority efforts for assessment of the diverse dragonfly fauna of tropical South America (*e.g.*, Pinto and Lamas 2010). Amongst the new records, the known distribution of *Acanthagrion jessei* (Coenagrionidae) was extended considerably from previous localities at Brazilian Amazonia and Pantanal. Another unexpected occurrence is of the South American emerald *Neocordulia setifera* (Anisoptera, *incertae sedis*), a species apparently confined to ombrophilous forested areas in the Atlantic Forest Domain with several misidentified specimens cited in the literature, including records from Brazil (MT, GO, MG), and Ecuador (APP, in prep.). Other specimens need further studies, such as, a possible new species of the *Oxyagrion basale*-group (Coenagrionidae) from PNSC; a single female with mesostigmal plate and mesepisternal fossae similar to that of the Central American *Acanthagrion quadratum* (not a well preserved specimen) from PNU; and specimens of *Argia*, a genus of difficult species identification, which are broken or smashed, thus were determined doubtfully (“cf.”). Specimens from PNU of the Amazonian *Castoraeschna corbeti* (Aeshnidae) and *Hetaerina indeprensa* (Calopterygidae), species previously only known from their type localities in Pará State (Brazil) are slightly distinct from typical specimens and need further studies to investigate if they only represent geographical variation.

Several published records of dragonfly species from the states of CE and PI are old, based on misidentifications or wrong localities, or for names currently under a different taxonomic status, and as a result, some of them should be ignored or are pending confirmation. Such cases are discussed below.

The Amazonian damselflies *Mnesarete cupraea* (Selys, 1853) and *Metaleptobasis bicornis* (Selys, 1877) were recorded from Ceará State (Garrison 2006: 21, von Ellenrieder 2013: 20; respectively) based on a misinterpretation of the locality of “Canindé” on the Rio Gurupi Basin at the border of Pará and Maranhão states (see Pinto and Lamas 2011). The occurrence of *M. cupraea* in Ceará was recently proven to be true for Serra da Ibiapaba (Table 2).

Belle 1983: 170-171), in his revision of *Zonophora*, provided a map where *Zonophora calippus calippus* Selys, 1869 and *Zonophora batesi* Selys, 1869 are apparently recorded from Piauí State, however it is clear that their location was based on the material from Maranhão cited by Belle 1972: 236-236). This mistake was reproduced by Garrison et al. 2006: 131) in their thumbnail map. Additionally, several other thumbnail maps for genera (Garrison et al. 2006) show ranges of distribution in both Ceará and Piauí States (*e.g.*, *Phyllogomphoides*, *Aeschnosoma*, and *Ypiranthemis*), however, we have not located references for these records and they should be considered potential distributions rather than actual occurrences. Even considering that species of some of these genera are common and widespread in South America, they are pending confirmation based on voucher specimens.

In the first published list of dragonflies from Ceará State, Navás (1924) cited currently considered dubious or questionable names due to changes in species concepts and very likely also based on misidentifications. These species were not included in Table 2. Navás (1924) cited *Erythrodiplax connata* (Burmeister, 1839), which likely refers to *E. fusca* since the former is restricted to Chile (Paulson 2003). *Erythrodiplax nigricans* was mentioned dubiously in the generic revision by Borror (1942), and based on its distribution in southern South America with the northernmost record from Rio de Janeiro State (Anjos-Santos and Costa 2006), it seems unlikely that it occurs in Northeastern Brazil. Three other species recorded by Navás (1924), *Micrathyria eximia* Kirby, 1897, *Orthemis ferruginea* (Fabricius, 1775), and *Perithemis domitia* (Drury, 1773), are very likely misidentifications. While reviewing *M. eximia*, Westfall (1992) observed that almost all specimens identified as this species were misidentified, including most Brazilian ones, as they indeed correspond to four species. *Orthemis ferruginea* is a largely North and Central American species with southernmost records to Costa Rica (Donnelly 1995), and its occurrence in South America is very unlikely. Material in which this record was based represent at least five species of the *O. ferruginea* group (see Pinto 2010). Finally, *P. domitia* is a Caribbean and Central America species with southernmost records in Venezuela and Colombia. Costa et al. (2006) considered all records of the latter species from Brazil as records of *P. mooma* and we agree with these authors.

### Increase of the known aquatic insect diversity of Ceará and Piauí states

Besides the high number of taxa recorded for the first time from Ceará and Piauí, effectively increasing the knowledge of aquatic insect diversity for these states, several undescribed species were detected. Twenty-six and 20 undescribed species from PNU and PNSC, respectively, were detected based on the material collected during this project, of those 12 from PNU (Câmara et al. 2015, Henriques-Oliveira and Santos 2014, Souza et al. 2014a, Souza et al. 2014b, Santos et al. 2016a) and four from PNSC (Cordeiro et al. 2014, Souza et al. 2014b, Souza et al. 2016a, Souza et al. 2016b) have already been described.

**Figure 29.**
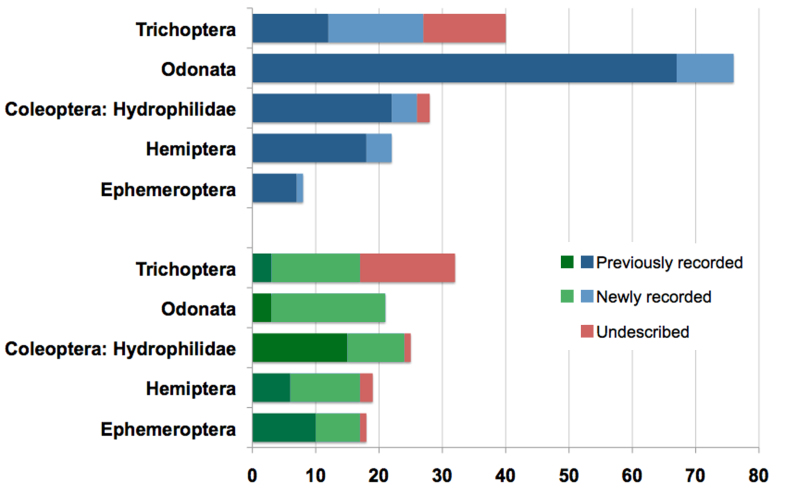
Number of previously recorded species of Trichoptera, Odonata, Hemiptera, Ephemeroptera, and Hydrophilidae (Coleoptera) for Ceará (blue) and Piauí (green). Dark blue or green represent the number of previously recorded species based on the literature. Light blue and green represent the number of species recorded for the first time in its respective state based on material collected at Ubajara (CE) and Sete Cidades (PI) National Parks. Number of undescribed species collected in these parks are given in red.

Many more aquatic insect species (except of Ephemeroptera) have been recorded from Ceará than Piauí (see Table 2). Our results represent an increase of approximately 16% in the number of known species for Ceará State, except for Trichoptera (Fig. 29). Caddisflies (the most diverse of groups sampled) seem to be understudied in both Brazilian states, with our sampling including a high proportion of species still to be described and results representing an increase of approximately 70% in the number of species known for Ceará and of 91% for Piauí. This increase for Piauí State is especially evident in other groups, such as, for Coleoptera, Ephemeroptera, and Hemiptera, which increased in approximately 51% of known species. This increase is even higher (86%) for dragonflies (Odonata) in Piauí, which is surprising given that they are possibly the most well-studied of focal groups.

### Species richness comparison between Parque Nacional de Ubajara (PNU) and Parque Nacional de Sete Cidades (PNSC)

There are no published lists of insect species for neither Parque Nacional de Ubajara (PNU) nor Parque Nacional de Sete Cidades (PNSC). In PNU’s management plan it is cited the occurrence of five orders and 14 insect families, with 6 genera and 9 species identified. However, these names were not made available. In another study, Silva and Ferreira (2009) list 26 families of 9 insect orders, including the identification solely of the genera *Conicera* (Phoridae), *Lutzomyia* (Psychodidae), and *Endecous* (Phalangopsidae), as part of a cave invertebrate inventory of PNU. The only insect faunal study published involving the two parks is a list of *Lutzomyia* (Diptera: Psychodidae) species by Martins et al. (1989), with five species occurring at PNU and seven species ate PNSC, while only Lutzomyia (L.) longipalpis (Lutz & Neiva, 1912) occurred in both parks. Thus, this preliminary list of aquatic insect taxa already represents a good sampling of insect diversity for these parks and a baseline for further monitoring and conservation strategies of freshwater macroinvertebrate fauna.

Considering all focal taxa, only 11% of species were found in both National Parks (Fig. 18), their species composition displaying very high complementarity (89% species). Although both parks are relatively close to eachother and within the Caatinga Domain, they drastically differ in phytophysiography. Specifically in the case of aquatic insects, the low complementary is more probably explained by the structural differences of water bodies sampled available in the different parks. At PNSC most part of aquatic insects (67 species) were collected at Cachoeira do Riachão (Fig. 3), a (supposedly) permanent stream with broad humid gallery forest and high diversity of microhabitats (sand, litter, and rocks), and in some temporary streams in open savannah vegetation (Fig. 4), which were partially dry during the second expedition and with lentic characteristics. The majority of water bodies sampled at PNU were very homogeneous, bedrock rapid streams and low diversity of microhabitats.

**Figure 30.**
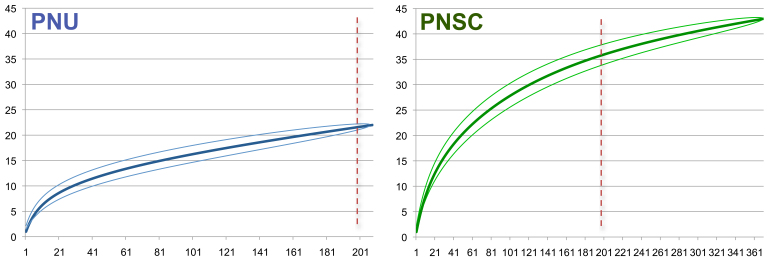
Rarefaction curves (thick lines) of aquatic insect richness from Ubajara (PNU, blue) and Sete Cidades (PNSC, green) National Parks with 95% confidence limits (fine lines). Dashed red line indicates the sample point of 200 individuals.

Although for some groups, *e.g.* Trichoptera, the number of species collected in each National Park was similar, rarefaction curves based on individuals sampled (Fig. 30) surprisingly suggest much higher expected richness of aquatic insect species at PNSC than at PNU, thus much more sampling is needed at PNSC to have a better estimate of total species richness. This is certainly the result of a higher range of aquatic microhabitats sampled at PNSC, as discussed above. However, it is still surprising that such a high diversity was found at PNSC in this study, given that the second expedition to these parks was conducted at the start of the rainy season, when some bodies of water sampled in the first expedition to PNSC (e.g., stream below Cachoeira do Riachão) were completely dry and resulted in the collection of a low number of individuals. Insect abundances in general seem to conspicuously peak during the rainy season and follow a very strong seasonality in Caatinga areas (Vasconcellos et al. 2010), which probably affected our effective sampling of a much higher richness from PNSC.

## Figures and Tables

**Figure 6.**
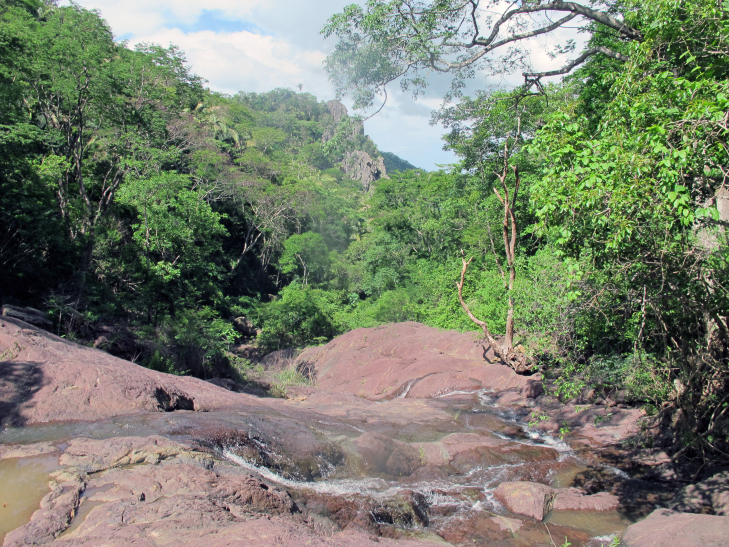
Rio das Minas crossing Trilha Araticum (PNU-02) at Parque Nacional de Ubajara, CE, Brazil.

**Figure 7.**
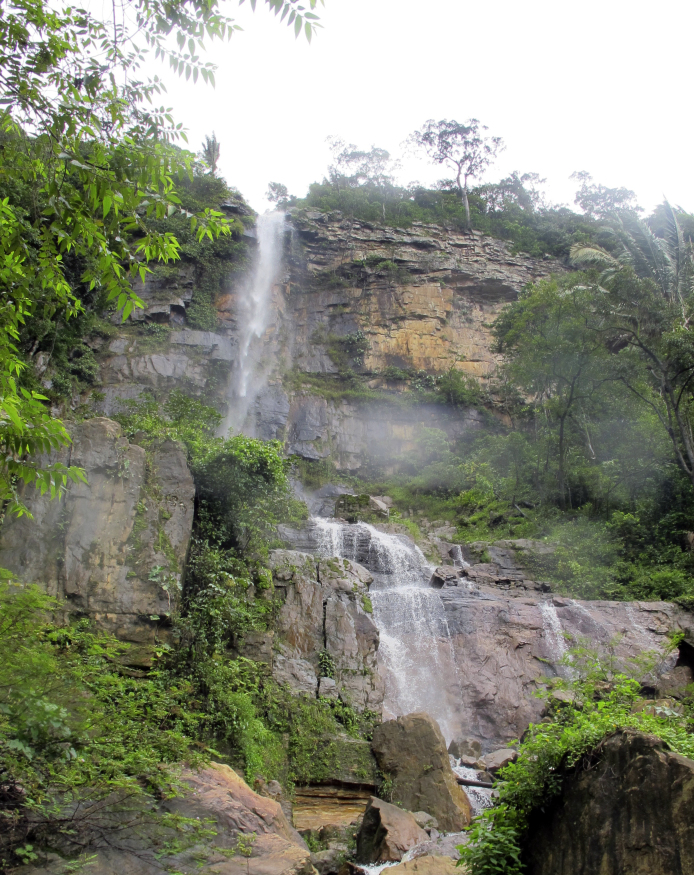
Rio Cafundó crossing Trilha Araticum (PNU-04) at Parque Nacional de Ubajara, CE, Brazil.

**Figure 8.**
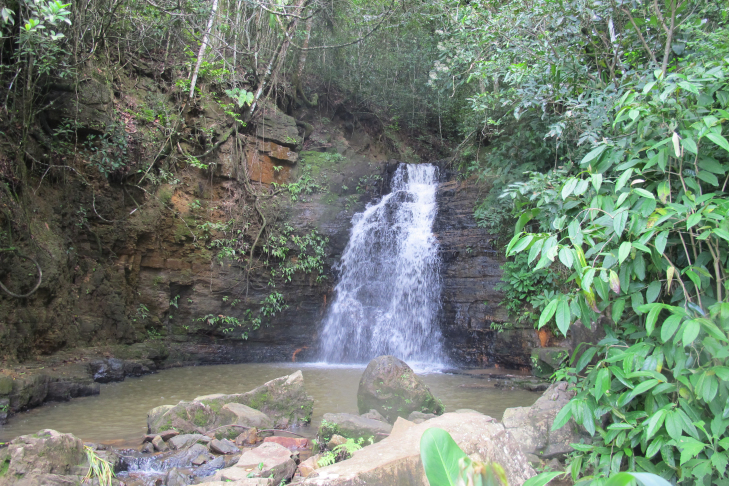
Cachoeira do Cafundó (PNU-07) at Parque Nacional de Ubajara, CE, Brazil.

**Figure 9.**
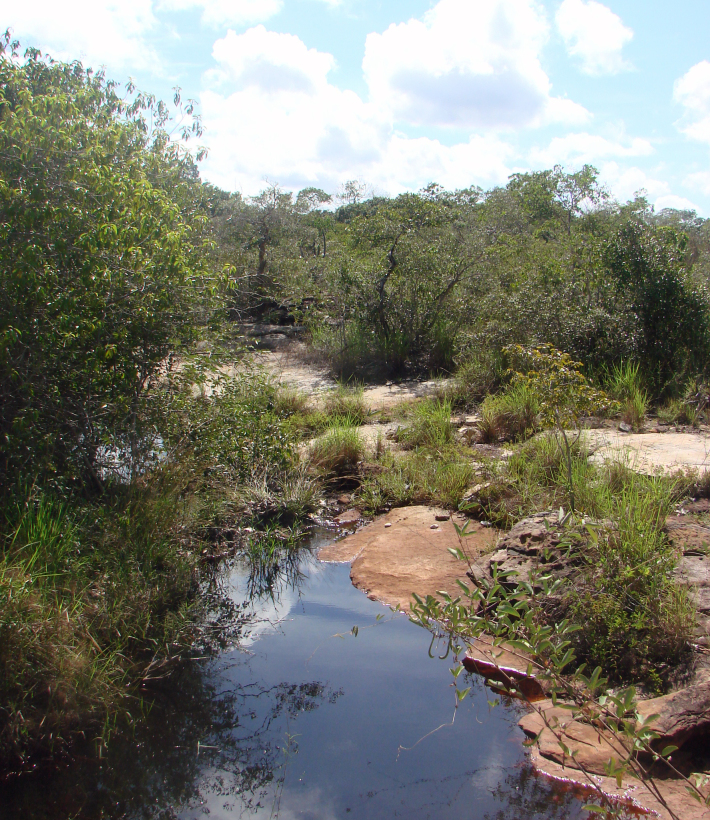
Riacho da Bananeira (PNSC-01) at Parque Nacional de Sete Cidades, PI, Brazil. Photo by A. Somavilla.

**Figure 13.**
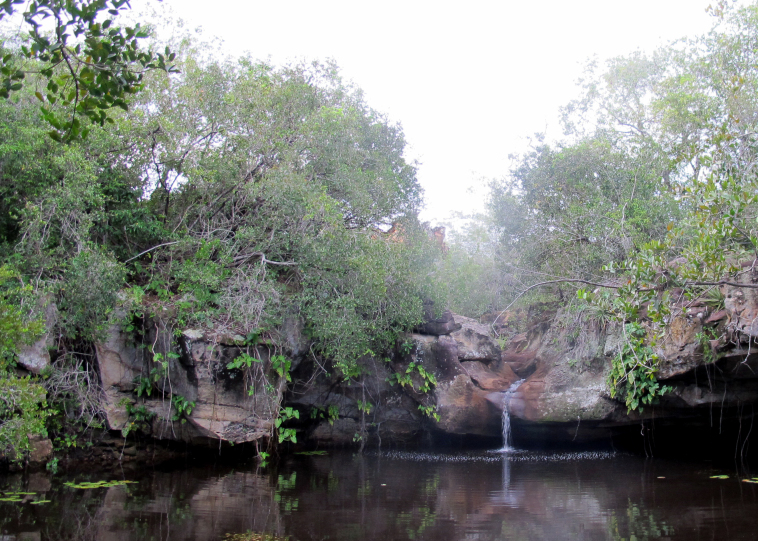
Poço do Bananeira (PNSC-08) at Parque Nacional de Sete Cidades, PI, Brazil.

**Figure 24.**
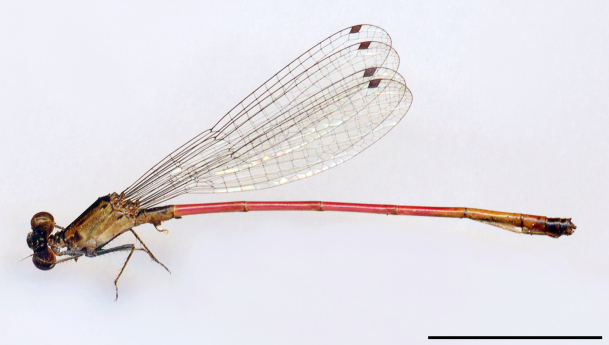
Lateral habitus of a male melanistic morph of *Oxyagrion chapadense* collected at PNU-02 (DZRJ). Scale bar = 10 mm.

**Table 1.** Number of described species in the major aquatic insect groups in the world and in Brazil, and percentage of Brazilian fauna in relation to the world’s biota.

**Taxon**	**World^a^**	**Brazil^b^**	**Brazilian fauna / World fauna**
Ephemeroptera	3,046	339	11.1%
Odonata	5,952	856	14.4%
Hemiptera: Nepomorpha	2,404	301	12.5%
Hemiptera: Gerromorpha*	2,021	228	11.3%
Plecoptera	3,497	164	4.7%
Diptera: Culicomorpha	19,618	1,719	8.8%
Diptera: Ephydridae	1,994	139	7.0%
Diptera: Psychodomorpha	3,412	519	15.2%
Diptera: Tipulomorpha	15,770	648	4.1%
Diptera: Tabanomorpha	5,373	498	9.3%
Trichoptera	14,291	687	4.8%
Megaloptera	328	20	6.1%
Coleoptera: Elmidae	1,300	148	11.4%
Coleoptera: Hydradephaga	5,126	529	10.3%
Coleoptera: Hydraenidae	1,380	27	2.0%
Coleoptera: Hydrophiloidea	2,205	280	12.7%
Coleoptera: Scirtidae	1,330	45	3.4%
**Total**	**89,047**	**7,147**	**8.0**%

^a^Numbers obtained from Dijkstra et al. (2014) (except Elmidae, from Jäch and Balke 2008).

^b^Numbers obtained from Boeger et al. (2016).

*Gerromorpha are semiaquatic.

**Table 2.** Species list of Coleoptera (Hydrophilidae), Ephemeroptera, Hemiptera (Gerromorpha and Nepomorpha), Odonata, and Trichoptera recorded from Ceará (CE) and Piauí (PI) states. Species marked with a "#" were described or firstly recorded to these states based on material sampled in this project.

**Insect family**	**Species**	**Previous record**	**Reference**
** COLEOPTERA **			
** Hydrophilidae **	*Berosus auriceps* Boheman, 1858	CE	Orchymont 1943
	*Berosus festivus* Berg, 1885	CE	Orchymont 1943
	*Berosus geayi* Orchymont, 1937	CE, PI	Orchymont 1943
	*Berosus novatus* Orchymont, 1940	PI	Orchymont 1943
	*Berosus patruelis* Berg, 1885	PI	Orchymont 1943
	*Berosus truncatipennis* Castelnau, 1840	CE, PI	Orchymont 1943
	*Crenitulus solstitialis* (Kirsch, 1873)	CE	Orchymont 1943
	*Crenitulus suturalis* (LeConte, 1866)	CE	Komarek 2005
	*Dactylosternum punctigerum* Knisch, 1924	CE	Knisch 1924
	*Derallus altus* (LeConte, 1855)	CE, PI	Orchymont 1943
	*Derallus ambitus* Orchymont, 1940	CE	Orchymont 1943
	*Derallus angustatus* Sharp, 1882	CE	Orchymont 1943
	*Derallus anicatus* Orchymont, 1940	PI	Orchymont 1943
	Enochrus (Methydrus) atlantis Orchymont, 1943	PI	Orchymont 1943
	*Hemiosus mornarius* Orchymont, 1940	CE	Orchymont 1943
	*Hemiosus mulvianus* Orchymont, 1940	CE, PI	Orchymont 1943
	*Hydrobiomorpha tricornis* Mouchamps, 1959	CE	Mouchamps 1959
	*Paracymus rufocinctus* Bruch, 1915	CE, PI	Orchymont 1943
	*Phaenonotum convexoides* Orchymont, 1943	CE	Orchymont 1943
	Tropisternus (Pleurhomus) sahlbergi (Sharp, 1883)	CE, PI	Orchymont 1943
	Tropisternus (Pristoternus) apicipalpis (Chevrolat,1834)	CE, PI	Orchymont 1943
	Tropisternus (Pristoternus) laevis (Sturm, 1826)	CE, PI	Orchymont 1943
	Tropisternus (Pristoternus) mutatus Orchymont, 1921	CE	Orchymont 1943
	Tropisternus (Pristoternus) ovalis (Castelnau, 1840)	CE, PI	Orchymont 1943
	Tropisternus (Pristoternus) regimbarti Orchymont, 1921	CE, PI	Orchymont 1943
	Tropisternus (Strepitornus) collaris (Fabricius, 1775)	CE, PI	Orchymont 1943
** DIPTERA **			
** Empididae **	#*Hemerodromia brevicercata* Câmara, Takiya, Plant & Rafael, 2015	CE	Câmara et al. 2015
	#*Hemerodromia membranosa* Câmara, Takiya, Plant & Rafael, 2015	CE	Câmara et al. 2015
	#*Hemerodromia mourai* Câmara, Takiya, Plant & Rafael, 2015	CE	Câmara et al. 2015
	#*Hemerodromia ubajaraensis* Câmara, Takiya, Plant & Rafael, 2015	CE	Câmara et al. 2015
** EPHEMEROPTERA **			
** Baetidae **	*Americabaetis alphus* Lugo-Ortiz & McCafferty, 1996	CE, PI	Boldrini et al. 2012
	*Callibaetis pollens* Needham and Murphy, 1924	CE, PI	Boldrini et al. 2012
	*Callibaetis guttatus* Navás, 1915	CE	Cruz et al. 2014
	*Camelobaetidius cayumba* (Traver & Edmunds, 1968)	CE, PI	Boldrini et al. 2012
	*Camelobaetidius janae* Dominique & Thomas, 2000	PI	Boldrini et al. 2012
	*Camelobaetidius tuberosus* Lugo-Ortiz & McCafferty, 1999	CE, PI	Boldrini et al. 2012
	*Cloeodes irvingi* Waltz & McCafferty, 1987	CE	Boldrini et al. 2012
	*Paracloeodes pacawara* Nieto & Salles, 2006	PI	Boldrini et al. 2012
	*Paracloeodes waimiri* Nieto & Salles, 2006	CE, PI	Boldrini et al. 2012
** Leptohyphidae **	Traverhyphes (Mocoihyphes) yuati Molineri, 2004	PI	Cruz et al. 2011
	*Tricorythodes mirca* Molineri, 2002	PI	Cruz et al. 2011
** Polymitarcyidae **	*Campsurus violaceus* Needham & Murphy, 1924	PI	Molineri et al. 2015
** HEMIPTERA **			
** Gerridae **	*Brachymetra albinervis albinervis* (Amyot & Serville, 1843)	CE	Dias da Rocha 1908
	*Brachymetra furva* Drake, 1957	PI	Rodrigues et al. 2012
	*Halobatopsis platensis* (Berg, 1879)	PI	Rodrigues et al. 2012
	#*Limnogonus profugus* Drake & Harris, 1930	CE	Cordeiro and Moreira 2015
	#*Neogerris lubricus* (White, 1879)	PI	Cordeiro and Moreira 2015
	*Rheumatobates crassifemur schroederi* Hungerford, 1954	CE	Hungerford 1954
	*Tachygerris adamsoni* (Drake, 1942)	PI	Rodrigues et al. 2012
** Mesoveliidae **	*Mesovelia amoena* Uhler, 1894	CE	Moreira and Campos 2012
	*Mesovelia mulsanti* White, 1879	CE	Moreira and Campos 2012
** Veliidae **	#*Microvelia ayacuchana* Drake & Maldonado Capriles, 1952	PI	Cordeiro and Moreira 2015
	*Microvelia mimula* White, 1879	CE	Moreira and Campos 2012
	#*Microvelia pulchella* Westwood, 1834	PI	Cordeiro and Moreira 2015
	#*Platyvelia brachialis* (Stål, 1860)	PI	Cordeiro and Moreira 2015
	#*Rhagovelia whitei* (Breddin, 1898)	CE	Cordeiro and Moreira 2015
** Belostomatidae **	*Belostoma anurum* (Herrich-Schäffer, 1848)	CE	Lauck 1962
	*Belostoma dallasi* De Carlo, 1930	CE	Ribeiro 2007
	*Belostoma dentatum* (Mayr, 1863)	PI	Ribeiro 2007
	*Belostoma elongatum* Montandon, 1908	PI	Ribeiro 2007
	*Belostoma foveolatum* (Mayr, 1863)	CE	Ribeiro 2000
	*Belostoma micantulum* (Stål, 1860)	CE	Dias da Rocha 1908
	*Lethocerus maximus* De Carlo, 1938	CE	Dias da Rocha 1936
** Corixidae **	*Heterocorixa wrighti wrigthi* Hungerford, 1948	CE	Hungerford 1948
	*Tenagobia incerta* Lundblad, 1929	CE	Nieser 1977
** Nepidae **	*Curicta montei* De Carlo, 1960	CE	Keffer 1997
** Notonectidae **	*Buenoa amnigenus* (White, 1879)	CE	Truxal 1953
	*Buenoa salutis* Kirkaldy, 1904	CE	Truxal 1953
	*Buenoa tarsalis* Truxal, 1953	CE	Truxal 1953
	*Buenoa unguis* Truxal, 1953	CE	Truxal 1953
	*Martarega brasiliensis* Truxal, 1949	CE	Truxal 1949
	*Martarega bentoi* Truxal, 1949	PI	Barbosa and Rodrigues 2013
	#*Notonecta disturbata* Hungerford, 1926	PI	Barbosa and Nessimian 2013
** Ochteridae **	#*Ochterus santosi* Cordeiro & Moreira, 2014	PI	Cordeiro et al. 2014
** ODONATA **			
** Aeshnidae **	*Anax amazili* (Burmeister, 1839)	CE	Navás 1924
	*Castoraeschna januaria* (Hagen, 1867)	CE	Navás 1924
	*Coryphaeschna viriditas* Calvert, 1952	CE	Navás 1924
	*Gynacantha nervosa* Rambur, 1842	CE	Navás 1924
** Gomphidae **	*Cacoides latro* (Erichson, 1848)	CE	Pinto 2010
	*Phyllocycla cf. gladiata* (Hagen in Selys, 1854)	CE	Nobre and Carvalho 2014
	*Progomphus dorsopallidus* Byers, 1934	CE	Nobre and Carvalho 2014
	#*Progomphus complicatus* Selys, 1854	CE	De Almeida et al. 2013
** Libellulidae **	*Brachymesia furcata* (Hagen, 1861)	CE	Navás 1924
	*Brachymesia herbida* (Gundlach, 1889)	CE	Pinto 2010
	*Brechmorhoga praecox praecox* (Hagen, 1861)	CE	Nobre and Carvalho 2014
	*Dasythemis esmeralda* Ris, 1910	CE	Nobre and Carvalho 2014
	*Diastatops obscura* (Fabricius, 1775)	CE	Navás 1924
	*Dythemis cf. nigra* Martin, 1897	CE	Nobre and Carvalho 2014
	*Dythemis velox* Hagen, 1861	CE	Navás 1924
	*Erythemis peruviana* (Rambur, 1842)	CE	Pinto 2010
	*Erythemis plebeja* (Burmeister, 1839)	CE	Navás 1924
	*Erythemis vesiculosa* (Fabricius, 1775)	CE	Pinto 2010
	*Erythrodiplax basalis* (Kirby, 1897)	CE	Navás 1924
	*Erythrodiplax fusca* (Rambur, 1842)	CE	Navás 1924
	*Erythrodiplax latimaculata* Ris, 1911	CE	Nobre and Carvalho 2014
	*Erythrodiplax leticia* Machado, 1996	CE, PI	Nobre and Carvalho 2014, Nobre 2016
	*Erythrodiplax ochracea* (Burmeister, 1839)	CE	Navás 1924
	*Erythrodiplax paraguayensis* (Förster, 1905)	CE	Pinto 2010
	*Erythrodiplax* sp.	CE	Nobre and Carvalho 2014
	*Erythrodiplax umbrata* (Linnaeus, 1758)	CE	Navás 1924
	*Idiataphe amazonica* (Kirby, 1889)	CE	Pinto 2010
	*Idiataphe cubensis* (Scudder, 1866)	CE	Navás 1924
	*Macrothemis griseofrons* (Calvert, 1909)	CE	Navás 1916
	*Macrothemis hemichlora* Burmeister, 1839	CE	Navás 1924
	*Macrothemis lutea* Calvert, 1909	CE	Nobre and Carvalho 2014
	*Miathyria marcella* (Selys in Sagra, 1857)	CE	Navás 1924
	*Micrathyria debilis* (Hagen, 1861)	CE	Navás 1924
	*Micrathyria hesperis* (Ris, 1911)	CE, PI	Navás 1924, Assis and Costa 1994
	*Micrathyria ocellata* Martin, 1897	CE	Navás 1924
	*Micrathyria tibialis* Kirby, 1897	CE	Navás 1924
	*Nephepeltia phryne* (Perty, 1834)	PI	Perty 1834
	*Oligoclada sylvia* (Kirby, 1899)	CE	Kirby 1889
	*Orthemis aequilibris* (Calvert, 1909)	CE	Nobre and Carvalho 2014
	*Orthemis aff. sulphurata* Hagen, 1868	CE	Pinto 2010
	*Orthemis ferruginea*-group	CE	Nobre and Carvalho 2014
	*Orthemis flavopicta* Kirby, 1889	CE	Nobre and Carvalho 2014
	*Pantala flavescens* (Fabricius, 1798)	CE	Nobre and Carvalho 2014
	*Perithemis bella* Kirby, 1889	CE	Navás 1924
	*Perithemis mooma* Kirby, 1889	CE, PI	Navás 1924, Costa et al. 2006
	*Tauriphila australis* Hagen, 1867	CE	Navás 1924
	*Tramea abdominalis* (Rambur, 1842)	CE	Pinto 2010
	*Tramea calverti* Muttkowski, 1910	CE	Nobre and Carvalho 2014
	*Tramea cophysa* Hagen, 1867	CE	Nobre and Carvalho 2014
	*Uracis imbuta* (Burmeister, 1839)	CE, PI	Costa and Santos 1997
** Calopterygidae **	*Hetaerina rosea* Selys, 1853	CE	Navás 1924
	*Mnesarete cupraea* (Selys, 1853)	CE	Carvalho and Bravo 2014
** Coenagrionidae **	*Acanthagrion gracile* (Rambur, 1842)	CE	Navás 1924
	*Argia cf. modesta* Selys, 1865	CE	Nobre and Carvalho 2014
	*Argia hasemani* Calvert, 1909	CE	Carvalho and Bravo 2014
	*Argia reclusa* Selys, 1865	CE	Navás 1924
	*Enallagma novaehispaniae* Calvert, 1907	CE	Nobre and Carvalho 2014
	*Ischnura capreolus* (Hagen, 1861)	CE	Navás 1924
	*Ischnura fluviatilis* Selys, 1876	CE	Pinto 2010
	*Leptagrion dardanoi* Santos, 1968	CE	Santos 1968
	*Neoneura sylvatica* Hagen in Selys, 1886	CE	Nobre and Carvalho 2014
	*Oxyagrion chapadense* Costa, 1978	CE	Carvalho and Bravo 2014
	*Telebasis corallina* (Selys, 1876)	CE	Nobre and Carvalho 2014
	*Telebasis filiola* (Perty, 1834)	CE	Nobre and Carvalho 2014
** Lestidae **	*Lestes forficula* Rambur, 1842	CE	Pinto 2010
** Perilestidae **	*Perilestes solutus* Williamson & Williamson, 1924	CE	Machado 2015
** PLECOPTERA **			
** Perlidae **	*Anacroneuria calori* Duarte & Lecci, 2016	CE	Duarte and Lecci 2016
** TRICHOPTERA **			
** Calamoceratidae **	*Phylloicus abdominalis* (Ulmer, 1905)	CE	Quinteiro et al. 2014​
	*Phylloicus bidigitatus* Prather, 2003	CE	Quinteiro et al. 2014
	*Phylloicus obliquus* Navás, 1931	CE	Quinteiro et al. 2014​
** Helicopsychidae **	*Helicopsyche* sp.	CE, PI	Quinteiro et al. 2014​
** Hydropsychidae **	*Leptonema pallidum* Guérin, 1834	CE	Costa et al. 2014
	*Leptonema viridianum* Navás, 1916	CE	Costa et al. 2014
	*Macrostemum hyalinum* (Pictet, 1836)	CE	Franca et al. 2013
	*Smicridea* sp.	CE	Quinteiro et al. 2014​
** Hydroptilidae **	#*Betrichia nhundiaquara* Souza, Santos & Takiya, 2016	PI	Souza et al. 2016b
	#*Flintiella harrisi* Souza, Santos & Takiya, 2016	PI	Souza et al. 2016a
	#*Hydroptila marighellai* Souza, Santos & Takiya, 2014	CE	Souza et al. 2014a
	#*Hydroptila florestani* Souza, Santos & Takiya, 2014	PI	Souza et al. 2014a
	#*Metrichia acuminata* Santos, Takiya & Nessimian, 2016	CE	Santos et al. 2016a
	#*Metrichia rafaeli* Santos, Takiya & Nessimian, 2016	CE	Santos et al. 2016a
	#*Metrichia ubajara* Santos, Takiya & Nessimian, 2016	CE	Santos et al. 2016a
	#*Metrichia vulgaris* Santos, Takiya & Nessimian, 2016	CE	Santos et al. 2016a
	#*Ochrotrichia caatinga* Souza, Santos & Takiya, 2014	CE	Souza et al. 2014b
	#*Ochrotrichia limeirai* Souza, Santos & Takiya, 2014	CE	Souza et al. 2014b
	#*Ochrotrichia patulosa* (Wasmund & Holzenthal, 2007)	CE	Souza et al. 2014b
** Leptoceridae **	#*Atanatolica nordestina* Henriques-Oliveira & Santos, 2014	CE	Henriques-Oliveira and Santos 2014
	*Nectopsyche splendida* (Navás, 1917)	PI	Quinteiro et al. 2014​
	#*Oecetis connata* Flint, 1974	PI	Henriques-Oliveira et al. 2014
	*Oecetis excisa* Ulmer, 1907	CE	Quinteiro et al. 2014​
	*Oecetis inconspicua* (Walker, 1852)	PI	Quinteiro et al. 2014​
	*Oecetis punctipennis* (Ulmer, 1905)	CE	Quinteiro et al. 2014​
** Odontoceridae **	*Marilia* sp.	CE	Quinteiro et al. 2014​
** Philopotamidae **	Chimarra (Curgia) conica Flint, 1983	CE	Flint 1998

**Table 3.** Sampling localities in Parque Nacional de Ubajara (PNU) and Parque Nacional de Sete Cidades (PNSC).

**Code**	**Locality**	**Coordinates**	**Altitude (m)**
PNU-01	PNU, Trilha Samambaia, Rio Gameleira (Fig. 5)	3°50'25"S, 40°54'19"W	874
PNU-02	PNU, Trilha Araticum, Rio das Minas (Fig. 6)	3°50'3"S, 40°54'18"W	524
PNU-03	PNU, Portão Neblina	3°50'18"S, 40°53'54"W	849
PNU-04	PNU, Trilha Araticum, Rio Cafundó (Fig. 7)	3°50'12"S, 40°54'31"W	753
PNU-05	PNU, Trilha Samambaia, Mirante da cachoeira do Gameleira	3°50'21"S, 40°54'23"W	880
PNU-06	PNU, Trilha Araticum, Rio das Minas na altura da trilha do teleférico	3°49'58"S, 40°53'53"W	420
PNU-07	PNU, Cachoeira do Cafundó (Fig. 8)	3°50'12"S, 40°54'35"W	783
PNU-08	PNU, Rio Cafundó, pouco acima da cachoeira	3°50'13"S, 40°54'35"W	795
PNU-09	PNU, Trilha Araticum, Rio da Minas abaixo do teleférico	3°49'43.3"S, 40°53'51.5"W	395
PNU-10	PNU, Ponte sobre Rio Miranda	3°50'7.4"S, 40°54'47.5"W	792
PNU-11	PNU, Rio das Minas, próximo ao Portão Araticum	3°49'32.6"S, 40°53'32.8"W	328
PNU-12	PNU, Trilha das Samambaias, brejo	3°50'25.8"S, 40°54'28.4"W	829
PNU-13	PNU, Mijo da Velha	3°50'16.7"S, 40°54'34.2"W	768
PNSC-01	PNSC, Riacho da Bananeira (Figs 9, 10)	4°5'59"S, 41°40'48"W	189
PNSC-02	PNSC, Cachoeira do Riachão (Figs 3, 11)	4°6'28"S, 41°40'13"W	171
PNSC-03	PNSC, Alojamento	4°5'57"S, 41°42'34"W	193
PNSC-04	PNSC, Riacho da Piedade (Fig. 4)	4°6'34"S, 41°43'39"W	169
PNSC-05	PNSC, Centro de visitantes	4°6'20"S, 41°41'52"W	202
PNSC-06	PNSC, Olho d'água Piscina do Bacuri	4°6'1.2"S, 41°42'38.8"W	171
PNSC-07	PNSC, Olho d'água dos Milagres (Fig. 12)	4°5'31.8"S, 41°40'48.2"W	180
PNSC-08	PNSC, Poço do Bananeira (Fig. 13)	4°5'55.8"S, 41°40'33.8"W	158
PNSC-09	PNSC, trilha para Poço do Bananeira	4°5'56.6"S, 41°40'32.3"W	162

## References

[B3005817] Anjos-Santos Danielle, Costa Janira M. (2006). A revised checklist of Odonata (Insecta) from Marambaia, Rio de Janeiro, Brazil with eight new records.. Zootaxa.

[B3366730] Assis C V, Costa J M (1994). Seis novas larvas do gênero *Micrathyria* Kirby e notas sobre a distribuição no Brasil (Odonata, Libellulidae). Revista Brasileira de Zoologia.

[B2851905] Barbosa Julianna F, Nessimian Jorge L (2013). New species and new records of *Notonecta* (Hemiptera: Heteroptera: Notonectidae) from Brazil. Zoologia (Curitiba).

[B2860571] Barbosa J F, Rodrigues H D D (2013). A new species of *Martarega* White, 1879, with new distributional records of Notonectidae (Hemiptera: Heteroptera: Nepomorpha) from Brazil. Zootaxa.

[B3005572] Batalha-Filho Henrique, Fjeldså Jon, Fabre Pierre-Henri, Miyaki Cristina Yumi (2013). Connections between the Atlantic and the Amazonian forest avifaunas represent distinct historical events. Journal of Ornithology.

[B3005777] Belle J (1972). Further studies on South American Gomphidae (Odonata). Tijdschrift voor Entomologie.

[B3005787] Belle J (1983). A review of the genus *Zonophora* Selys (Odonata, Gomphidae). Tijdschrift Voor Entomologie.

[B2851938] Boeger W A, Zaher H, Rafael J A, Valim M P Catálogo Taxonômico da Fauna do Brasil.. http://fauna.jbrj.gov.br/fauna/listaBrasil/ConsultaPublicaUC.

[B2851947] Boldrini R, Cruz P V, Salles F F, Belmont E L, Hamada N (2012). Baetidae (Insecta: Ephemeroptera) from northeastern Brazil. Checklist.

[B3005797] Borror D J (1942). A revision of Libellulinae genus *Erythrodiplax* (Odonata). Contributions in Zoology and Entomology.

[B2851961] Câmara J T, Takiya D M, Plant A R, Rafael J A (2015). Neotropical *Hemerodromia* Meigen (Diptera: Empididae), a world of discovery II: New species from Atlantic forest, Brazil. Zootaxa.

[B3005552] Carvalho Alcimar L., Pinto Ângelo P., Ferreira-Jr Nelson (2009). *Castoraeschna
corbeti* sp. nov. from Floresta Nacional de Carajás, Pará state, Brazil (Odonata: Aeshnidae). International Journal of Odonatology.

[B2916514] Carvalho J, Bravo F (2014). Odonata do semiárido. p. 83-89. In: Bravo F, Calor A (Orgs). Artrópodes do Semiárido: biodiversidade e conservação..

[B2917138] Castelletti C H M, Santos A M M, Tabarelli M, da Silva J M C., Leal I R, Tabarelli M, da Silva J M C (2003). Quanto ainda resta da Caatinga? Uma estimativa preliminar.. Ecologia e Conservação da Caatinga.

[B2851980] Chapman R F (1998). The Insects. Structure and Function.

[B2851989] Cheng L (1976). Marine insects.

[B2942084] Colwell RK, Coddington JA (1994). Estimating terrestrial biodiversity through extrapolation.. Philosophical Transactions of the Royal Society (Series B).

[B2980246] Cordeiro Isabelle R S, Moreira Felipe F F (2015). New distributional data on aquatic and semiaquatic bugs (Hemiptera: Heteroptera: Gerromorpha & Nepomorpha) from South America. Biodiversity Data Journal.

[B2852009] Cordeiro I R S, Moreira F F F, Silva F A C (2014). A new *Ochterus* (Hemiptera: Heteroptera: Ochteridae) from northeastern Brazil, with a key to the species recorded from the country. Zootaxa.

[B2852029] Costa A M, Quinteiro F B, Calor A R (2014). Trichoptera do Semiárido I: Annulipalpia. p. 225-228. In: Bravo F, Calor A (Orgs). Artrópodes do Semiárido: biodiversidade e conservação.

[B2916794] Costa J M, Santos T C (1997). Intra and interspecific variation in the genus *Uracis* Rambur, 1842, with a key to the known species (Anisoptera: Libellulidae). Odonatologica.

[B2916759] Costa J M, Souza L O I, Muzón J (2006). Descriptions of three new species of Odonata from Brazil. Zootaxa.

[B2852049] Coutinho L M (2006). O conceito de bioma. Acta Botanica Brasiliense.

[B2912091] Cruz P V, Salles F F, Hamada N (2014). *Callibaetis* Eaton (Ephemeroptera: Baetidae) from Brazil. Journal of Natural History.

[B2852059] Cruz P V, Belmont E L, Boldrini R, Hamada N (2011). Leptohyphidae (Insecta: Ephemeroptera) from northeastern Brazil. Neotropical Entomology.

[B2852069] De Almeida M V O, Pinto A P, Carvalho A L, Takiya D M (2013). When rare is just a matter of sampling: unexpected dominance of clubtail dragonflies (Odonata, Gomphidae) through different collecting methods at Parque Nacional da Serra do Cipó, Minas Gerais State, Brazil. Revista Brasileira de Entomologia.

[B2852524] Dias da Rocha F (1908). Insectos. Boletim do Museu Rocha.

[B2853177] Dias da Rocha F (1936). Subsídios para o estudo da fauna cearense. III. Insecta.. Nordeste Agrícola.

[B2853811] Dijkstra K B, Monaghan M T, Pauls S U (2014). Freshwater Biodiversity and Aquatic Insect Diversification. Annual Review of Entomology.

[B3005889] Donnelly T W (1995). *Orthemis
ferruginea* - An adventure in Caribbean biogeography. Argia.

[B3005717] Duarte T, Lecci L S (2014). Stoneflies (Insecta: Plecoptera) from Serra Bonita, Bahia, Brazil: New species and updated records. Zootaxa.

[B3276585] Duarte T., Lecci L. S. (2016). New species and records of *Anacroneuria* (Plecoptera: Perlidae) from the northeastern semi-arid region of Brazil. Zootaxa.

[B3005727] Duarte T, Bispo P. C., Calor A R (2014). A new species of *Tupiperla* Froehlich, 1969 (Plecoptera: Gripopterygidae) from Serra da Jibóia, Bahia, Brazil. Zootaxa.

[B2854102] Flint O S Jr (1998). Studies of Neotropical Caddisflies, LIII: A taxonomic revision of the subgenus *Curgia* of the genus *Chimarra* (Trichoptera: Philopotamidae). Smithsonian Contributions to Zoology.

[B2854112] Franca D, Paprocki H, Calor A R (2013). The genus *Macrostemum* Kolenati 1859 (Trichoptera: Hydropsychidae) in the Neotropical Region: Description of two new species, taxonomic notes, distributional records and key to males. Zootaxa.

[B3005757] Froehlich C G (2010). Catalogue of Neotropical Plecoptera. Illiesia.

[B2914421] Frost S. W. (1957). The Pennsylvania insect light trap. Journal of Economic Entomology.

[B3005866] Garrison R W (2006). A synopsis of the genera *Mnesarete* Cowley, *Bryoplathanon* gen. nov., and *Ormenoplebia* gen. nov. (Odonata: Calopterygidae). Contributions in Science.

[B3005847] Garrison R W, von Ellenrieder N, Louton J A (2006). Dragonfly genera of the New World: an illustrated and annotated key to the Anisoptera.

[B2914401] Gressitt J. L., Gressitt M. K. (1962). An improved malaise trap. Pacific Insects.

[B2854122] Grimaldi D, Engel M S (2005). Evolution of the Insects.

[B2854131] Hammer Ø, Harper D A T, Ryan P D (2001). PAST: Paleontological Statistics Software Package for Education and Data Analysis. Palaeontologia Electronica.

[B2854153] Henriques-Oliveira A L, Santos A P M (2014). Two new species of *Atanatolica* Mosely 1936 (Trichoptera: Leptoceridae) from Peru and Northeastern Brazil. Zootaxa.

[B2854143] Henriques-Oliveira A L, Dumas L L, Nessimian J L (2014). Three new species and new distributional records of *Oecetis* McLachlan 1877 (Trichoptera: Leptoceridae: Leptocerinae) from Brazil. Zootaxa.

[B2854163] Hungerford H B (1948). The Corixidae of the Western Hemisphere (Hemiptera). University of Kansas Science Bulletin.

[B2854203] Hungerford H B (1954). The genus *Rheumatobates* Bergroth (Hemiptera-Gerridae). University of Kansas Science Bulletin.

[B2854183] Jäch M A, Balke M (2008). Global diversity of water beetles (Coleoptera) in freshwater. Developments in Hydrobiology.

[B2854193] Keffer S L (1997). Systematics of the New World waterscorpion genus *Curicta* Stål (Heteroptera: Nepidae). Journal of the New York Entomological Society.

[B2916729] Kirby W. F. (1889). A revision of the subfamily Libellulinae, with descriptions of new genera and species. Transactions of the Zoological Society of London.

[B2982658] Knisch A. (1924). Neue neotropische Palpicornier (Col. Hydrophilidae. – Op. 16.). Wiener Entomologische Zeitung.

[B2982648] Komarek Albrecht (2005). Taxonomic revision of *Anacaena* Thomson, 1859 II. Neotropical species (Coleoptera: Hydrophilidae). Koleopterologische Rundschau.

[B2854213] Lauck D R (1962). A monograph of the genus *Belostoma* (Hemiptera) Part I. Introduction to *Belostoma
dentatum* and *subspinosum* groups. Bulletin of the Chicago Academy of Sciences.

[B2917275] Leal I R, Da Silva J M C, Tabarelli M, Lacher T E (2005). Changing the Course of Biodiversity Conservation in the Caatinga of Northeastern Brazil. Conservation Biology.

[B2877419] Lecci L, Froehlich C G Plecoptera in Catálogo Taxonômico da Fauna do Brasil. http://fauna.jbrj.gov.br/fauna/faunadobrasil/304.

[B3005747] Lecci L S, Froehlich C G (2011). Taxonomic revision of *Gripopteryx* (Pictet, 1841) (Plecoptera: Gripopterygidae). Zootaxa.

[B3004388] Lecci L S, Simões T V D, Calor A R (2014). Plecoptera do Semiárido: conhecimento atual e desafios. p. 91-98. In: Bravo F, Calor A (Orgs). Artrópodes do Semiárido: biodiversidade e conservação.

[B2916494] Machado Angelo B. M. (2015). *Perilestes
eustaquioi* sp. nov. and new distributional records of Perilestidae (Odonata) in Brazil. Zoologia.

[B2978867] Martins Amilcar Vianna, Dias Edelberto Santos, Falcão Alda Lima, da Silva João Evangelista (1989). Notas sobre os flebótomos dos estados do Ceará e Piauí, com a descrição da fêmea de *Lutzomyia
samueli* (Deane, 1955) (Diptera, Psychodidae, Phlebotominae). Memórias do Instituto Oswaldo Cruz.

[B2854223] Merritt R W, Cummins K W (1996). An Introduction to the Aquatic Insects of North America.

[B3004305] Mittermeier Russell A., Turner Will R., Larsen Frank W., Brooks Thomas M., Gascon Claude (2011). Global Biodiversity Conservation: The Critical Role of Hotspots. Biodiversity Hotspots.

[B2912101] Molineri C, Salles F F, Emmerich D (2015). Revision of *Campsurus
violaceus* species group (Ephemeroptera: Polymitarcyidae) with new synonymies and nomina dubia in *Campsurus* Eaton, 1868. Zootaxa.

[B2878467] Monné M L, Ferreira V S, Thomas M C, Costa C, Ferreira-Jr N, Aloquio S, Lopes-Andrade C, Sandoval-Gómez V E, Pollock D A, Sekerka L, Linzmeier A M, Bulirsch P, Flores G E, McHugh J, Moura L A, Gimmel M L, Segura M O, Lord N, Constantin R, Almeida L M, Grossi P C, Grzymala T L, Biffi G, Vaz-de-Mello F Z, Caron E, Spiessberger E L, Bicho C L, Chandler D S, Mermudes J R M, Vanin S A, Bená D C, Cline A, Monné M A, Souza D S, Hájek J, Anichtchenko A, Ribeiro-Costa C S, Agrain F, Chamorro M L, Cupello M, Smith T R, Botero J P, Sampaio B H L, Passos M I, Vaz S, Colpani D, Benetti C J, Leivas F W L, Degallier N, Gnaspini P, Nascimento E A, Quintino H Y S, McElrath T C, Powell G, Escalona H, Barbosa F F, Casari S, Silva A A S, Shockley F, Manfio D, Slipinski A, Leschen R A B, Cruz L S, Nascimento F E L, Tomaszewska W, Newton A F, Peck S B, Silveira L F L, Hamada N, Johnson C, Castro-Guedes C F, Santos P B, Aragão A C, Puker A, Morse G E, Corrêa R C, Rosa S P, Ivie M A Coleoptera in Catálogo Taxonômico da Fauna do Brasil. http://fauna.jbrj.gov.br/fauna/faunadobrasil/223.

[B2854242] Moreira F F F Water Bugs Distributional Database. http://sites.google.com/site/distributionaldatabase.

[B2854264] Moreira F F F, Campos G G F (2012). New distributional data concerning some Gerromorpha (Insecta: Hemiptera: Heteroptera) from Brazil. Check List.

[B2854251] Moreira F F F, Barbosa J F, Ribeiro J R I, Alecrim V P (2011). Checklist and distribution of semiaquatic and aquatic Heteroptera (Gerromorpha and Nepomorpha) occurring in Brazil. Zootaxa.

[B2982678] Mouchamps R. (1959). Remarques concernant les genres Hydriobiomorpha Blackburn et Neohydrophilus Orchymont (Coléoptères Hydrophilides). Bulletin et annales de la Société royale d’Entomologie de Belgique.

[B2854275] Myers N, Mittermeier R A, Mittermeier C G, de Fonseca G A B, Kent J (2000). Biodiversity hotspots for conservation priorities. Nature.

[B2916547] Navás L (1916). Neuroptera nova americana. II Series.. Memorie dell’ Accademia pontificia dei Nuovi Lincei, Ser. 2.

[B2916504] Navás L (1924). Odonatos nuevos o interesantes. Memorias de la Real Academia de Ciencias y Artes de Barcelona.

[B2854289] Nieser N (1977). A revision of the genus *Tenagobia* Bergroth (Heteroptera: Corixidae). Studies on Neotropical Fauna and Environment.

[B2854299] Nobre C E, Carvalho A L (2014). Odonata of Itatira, a Brazilian semi-arid area in the state of Ceará. International Journal of Odonatology.

[B3228493] Nobre Carlos Eduardo Beserra (2016). *Erythrodiplax
leticia*: Description of the female and updated geographic distribution (Odonata: Libellulidae). Zootaxa.

[B2982688] Orchymont A. d’. (1943). Faune du nord-est brésilien (récoltes du Dr O. Schubart): Palpicornia.. Musée royal d'histoire naturelle de Belgique.

[B3005807] Paulson Dennis R. (2003). Comments on the *Erythrodiplax
connata* (Burmeister, 1839) group, with the elevation of *Erythrodiplax
fusca* (Rambur, 1842), *Erythrodiplax
minuscula* (Rambur, 1842), and *Erythrodiplax
basifusca* (Calvert, 1895) to full species (Anisoptera: Libellulidae). Bulletin of American Odonatology.

[B2916699] Perty J A M (1834). Delectus animalium articulatorum, quae in itinere per Brasiliam, annis MDCCXVII-MDCCCXX jussu et auspiciis Maximiliani Josephi I.

[B2916474] Pinto Ângelo P (2010). A Sertanejo’s Trip: Occurrence of *Orthemis
sulphurata* Hagen in Northeastern Brazil?. Argia.

[B2874439] Pinto Ângelo P Odonata in Catálogo Taxonômico da Fauna do Brasil. http://fauna.jbrj.gov.br/fauna/faunadobrasil/171.

[B3005620] Pinto Ângelo Parise, Lamas Carlos José Einicker (2010). *Navicordulia
aemulatrix* sp. nov. (Odonata, Corduliidae) from northeastern Santa Catarina State, Brazil. Revista Brasileira de Entomologia.

[B3005562] Pinto Ângelo Parise, Lamas Carlos Jose Einicker (2011). *Oligoclada
mortis* sp. nov. from Rondônia State, Brazil, and distributional records of other species of the genus (Odonata: Libellulidae). International Journal of Odonatology.

[B2917090] Prado D E, Leal I R, Tabarelli M, da Silva J M C (2003). As Caatingas da América do Sul. Ecologia e Conservação da Caatinga.

[B2854309] Quinteiro F B, Costa A M, Calor A R (2014). Trichoptera do Semiárido II: Integripalpia. p. 229-244. In: Bravo F, Calor A (Orgs). Artrópodes do Semiárido: biodiversidade e conservação.

[B2873693] Rafael J A, Câmara J T Empididae in Catálogo Taxonômico da Fauna do Brasil. http://fauna.jbrj.gov.br/fauna/faunadobrasil/2379.

[B2914411] Rafael J A R, Gorayeb I S (1982). Tabanidae (Diptera) da Amazônia, I - Uma nova armadilha suspensa e primeiros registros de mutucas de copas de árvores. Acta Amazonica.

[B2854323] Ribeiro J R I (2000). Description of the male of *Belostoma
foveolatum* and new records of *Belostoma
costalimai* and *Belostoma
stollii* (Heteroptera: Belostomatidae). Entomological News.

[B2854333] Ribeiro J R I (2007). A review of the species of *Belostoma* Latreille, 1807 (Hemiptera: Heteroptera: Belostomatidae) from the four southeastern Brazilian states.. Zootaxa.

[B3005767] Righi-Cavallaro Karina O., Froehlich Claudio G., Lecci Lucas S. (2013). New species of *Anacroneuria* (Plecoptera: Perlidae) from northeast Brazil. Studies on Neotropical Fauna and Environment.

[B2854343] Rodrigues H D D, de Melo A L, Ferreira-Kepler R L (2012). New records of Gerromorpha (Insecta: Hemiptera: Heteroptera) from Brazil. Check List.

[B2854355] Rosenberg D M, Resh V H (1993). Freshwater Biomonitoring and Benthic Macroinvertebrates.

[B2875914] Salles F F, Boldrini R Ephemeroptera in Catálogo Taxonômico da Fauna do Brasil. http://fauna.jbrj.gov.br/fauna/faunadobrasil/122.

[B2854374] Salles F F, Massariol F C, Nascimento J M C, Boldrini R, Raimundi E A, Angeli K B, Souto P M Ephemeroptera do Brasil. http://ephemeroptera.com.br/.

[B3005530] Santos André M. Melo, Cavalcanti Deyvson Rodrigues, da Silva José Maria Cardoso, Tabarelli Marcelo (2007). Biogeographical relationships among tropical forests in north-eastern Brazil. Journal of Biogeography.

[B3276605] Santos Allan Paulo Moreira, Takiya Daniela M., Nessimian Jorge L. (2016). Integrative taxonomy of *Metrichia* Ross (Trichoptera: Hydroptilidae: Ochrotrichiinae) microcaddisflies from Brazil: descriptions of twenty new species. PeerJ.

[B2854386] Santos A P M, Calor A R, Dumas L L, Pes A M O, Souza W R M, Henriques-Oliveira A L, Camargos L M Trichoptera in Catálogo Taxonômico da Fauna do Brasil. http://fauna.jbrj.gov.br/fauna/faunadobrasil/278.

[B2916528] Santos N. D. (1968). Descrição de *Leptagrion
dardanoi* sp. n. (Odonata, Coenagrionidade). Atas da Sociedade de Biologia do Rio de Janeiro.

[B2854398] Silva M S, Ferreira R L (2009). Caracterização ecológica de algumas cavernas do Parque Nacional de Ubajara (Ceará) com considerações sobre o turismo nestas cavidades. Revista de Biologia e Ciências da Terra.

[B2854441] Souza W R M, Santos A P M, Takiya D M (2014). First records of *Ochrotrichia* Mosely, 1934 (Trichoptera: Hydroptilidae) in Northeastern Brazil: Five new species and two new geographical records. Zootaxa.

[B2854409] Souza W R M, Santos A P M, Takiya D M (2014). Three new species of *Hydroptila* (Trichoptera: Hydroptilidae) from Northeastern Brazil. Zoologia (Curitiba).

[B2854419] Souza W R M, Santos A P M, Takiya D M (2016). Description of a new species of *Betrichia* Mosely 1939 from Brazil and edescription of the type-species (Trichoptera: Hydroptilidae: Leucotrichiinae). Zootaxa.

[B2857087] Souza W R M, Santos A P M, Takiya D M (2016). Three new species of Stactobiinae (Trichoptera: Hydroptilidae) with the first record of the genus *Orinocotrichia* Harris, Flint & Holzenthal from Brazil. Zootaxa.

[B2917285] Tabarelli M, Vicente A, da Silva J M C, Tabarelli M, Fonseca M, Lins L (2004). Conhecimento sobre plantas lenhosas da Caatinga: lacunas geográficas e ecológicas. Biodiversidade da Caatinga: áreas e ações prioritárias para conservação.

[B2854477] Truxal F S (1949). A study of the genus *Martarega* (Hemiptera: Notonectidae). Journal of the Kansas Entomological Society.

[B2854487] Truxal F S (1953). A revision of the genus *Buenoa* (Hemiptera: Notonectidae). University of Kansas Science Bulletin.

[B2917504] Vasconcellos Alexandre, Andreazze Ricardo, Almeida Adriana M., Araujo Helder F. P., Oliveira Eduardo S., Oliveira Uirandé (2010). Seasonality of insects in the semi-arid Caatinga of northeastern Brazil. Revista Brasileira de Entomologia.

[B3004295] Vianna Dana Moiana, De Marco Júnior P (2012). Higher-Taxon and Cross-Taxon Surrogates for Odonate Biodiversity in Brazil. Natureza & Conservação.

[B3005856] von Ellenrieder Natalia (2013). A revision of *Metaleptobasis* Calvert (Odonata: Coenagrionidae) with seven synonymies and the description of eighteen new species from South America. Zootaxa.

[B3005606] Wang Xianfeng, Auler Augusto S., Edwards R. Lawrence, Cheng Hai, Cristalli Patricia S., Smart Peter L., Richards David A., Shen Chuan-Chou (2004). Wet periods in northeastern Brazil over the past 210 kyr linked to distant climate anomalies. Nature.

[B3005541] Werneck Fernanda P., Nogueira Cristiano, Colli Guarino R., Sites Jack W., Costa Gabriel C. (2012). Climatic stability in the Brazilian Cerrado: implications for biogeographical connections of South American savannas, species richness and conservation in a biodiversity hotspot. Journal of Biogeography.

[B3005827] Westfall M J (1992). Notes on *Micrathyria*, with descriptions of *Micrathyria
pseudeximia* sp. n., *Micrathyria
occipita* sp. n., *Micrathyria
dunklei* sp. n. and *Micrathyria
divergens* sp. n. (Anisoptera: Libellulidae).. Odonatologica.

